# Molecular characterization and descriptive analysis of carbapenemase-producing Gram-negative rod infections in Bogota, Colombia

**DOI:** 10.1128/spectrum.01714-23

**Published:** 2024-04-17

**Authors:** Elsa D. Ibáñez-Prada, Ingrid G. Bustos, Enrique Gamboa-Silva, Diego F. Josa, Lina Mendez, Yuli V. Fuentes, Cristian C. Serrano-Mayorga, Oscar Baron, Alejandra Ruiz-Cuartas, Edwin Silva, Louise M. Judd, Taylor Harshegyi, Hector F. Africano, Juan Urrego-Reyes, Claudia C. Beltran, Sebastian Medina, Rafael Leal, Andrew J. Stewardson, Kelly L. Wyres, Jane Hawkey, Luis Felipe Reyes

**Affiliations:** 1 Unisabana Center for Translational Science, School of Medicine, Universidad de la Sabana, Chía, Colombia; 2 Critical Care Department, Clínica Universidad de La Sabana, Chía, Colombia; 3 Microbiology Department, Fundación Clínica Shaio, Bogota, Colombia; 4 Department of Infectious Diseases, Central Clinical School, Monash University, Melbourne, Victoria, Australia; 5 Global Medical Scientific Affairs, MSD Colombia, Bogota, Colombia; 6 Pandemic Sciences Institute, University of Oxford, Oxford, United Kingdom; University of Pretoria, Pretoria, Gauteng, South Africa

**Keywords:** antimicrobial resistance, carbapenem resistance, carbapenemase-encoding genes, KPC, sequence analysis

## Abstract

In this study, the genetic differences and clinical impact of the carbapenemase-encoding genes among the community and healthcare-acquired infections were assessed. This retrospective, multicenter cohort study was conducted in Colombia and included patients infected with carbapenem-resistant Gram-negative rods between 2017 and 2021. Carbapenem resistance was identified by Vitek, and carbapenemase-encoding genes were identified by whole-genome sequencing (WGS) to classify the alleles and sequence types (STs). Descriptive statistics were used to determine the association of any pathogen or gene with clinical outcomes. A total of 248 patients were included, of which only 0.8% (2/248) had community-acquired infections. Regarding the identified bacteria, the most prevalent pathogens were *Pseudomonas aeruginosa* and *Klebsiella pneumoniae*. In the WGS analysis, 228 isolates passed all the quality criteria and were analyzed. The principal carbapenemase-encoding gene was *bla*KPC, specifically *bla*KPC-2 [38.6% (88/228)] and *bla*KPC-3 [36.4% (83/228)]. These were frequently detected in co-concurrence with *bla*VIM-2 and *bla*NDM-1 in healthcare-acquired infections. Notably, the only identified allele among community-acquired infections was *bla*KPC-3 [50.0% (1/2)]. In reference to the STs, 78 were identified, of which *Pseudomonas aeruginosa* ST111 was mainly related to blaKPC-3. *Klebsiella pneumoniae* ST512, ST258, ST14, and ST1082 were exclusively associated with *bla*KPC-3. Finally, no particular carbapenemase-encoding gene was associated with worse clinical outcomes. The most identified genes in carbapenemase-producing Gram-negative rods were *bla*KPC-2 and *bla*KPC-3, both related to gene co-occurrence and diverse STs in the healthcare environment. Patients had several systemic complications and poor clinical outcomes that were not associated with a particular gene.

**IMPORTANCE:**

Antimicrobial resistance is a pandemic and a worldwide public health problem, especially carbapenem resistance in low- and middle-income countries. Limited data regarding the molecular characteristics and clinical outcomes of patients infected with these bacteria are available. Thus, our study described the carbapenemase-encoding genes among community- and healthcare-acquired infections. Notably, the co-occurrence of carbapenemase-encoding genes was frequently identified. We also found 78 distinct sequence types, of which two were novel *Pseudomonas aeruginosa*, which could represent challenges in treating these infections. Our study shows that in low and middle-income countries, such as Colombia, the burden of carbapenem resistance in Gram-negative rods is a concern for public health, and regardless of the allele, these infections are associated with poor clinical outcomes. Thus, studies assessing local epidemiology, prevention strategies (including trials), and underpinning genetic mechanisms are urgently needed, especially in low and middle-income countries.

## INTRODUCTION

Antimicrobial resistance (AMR) is responsible for more than 4.95 million deaths globally (1), and it is forecasted that the mortality rate due to AMR will increase by up to 10 million annually by 2050. Moreover, the World Bank calculates an annual fall of the global gross domestic product by 1.1%–3.8%; this shortfall would exceed $1–3.4 trillion annually, secondary to AMR (2). This scenario worsens when Gram-negative rods become resistant to carbapenems used as part of the last-line antimicrobial treatments (3, 4). Notably, the frequency of carbapenem-resistant infections is three times higher in low and middle-income countries (LMIC) (5), especially in South America, due to the carriage of carbapenemase-encoding genes (6).

Previous worldwide studies have shown that the carbapenem resistance epidemiology of carbapenemase-encoding genes varies. *bla*KPC infections most commonly occur in the United States, metallo-β-lactamases in the Indian Subcontinent and some European countries, and the epicenter of *bla*OXA-48-like is in Turkey and its surroundings (7). Colombia has a high prevalence of infections caused by carbapenemase-producing Gram-negative rods (8); for instance, the country described the first *bla*KPC-producing *Pseudomonas aeruginosa* in 2006 (9, 10). Interestingly, the co-occurrence of carbapenemase-encoding genes has also been reported, such as *bla*KPC + *bla*VIM, *bla*NDM + *bla*OXA-48-like, and *bla*VIM + *bla*OXA-48-like (11, 12). Co-occurrence has become frequent in Latin America and represents a significant diagnostic and therapeutic challenge since it creates major difficulty for clinical treatment, leaving healthcare professionals without therapeutic alternatives for infected patients (13, 14).

Carbapenem-resistant infections are frequently acquired in healthcare-related environments (15). However, in recent years, the rate of community-acquired resistant infections has increased (16), and current evidence is scarce on this topic. Moreover, there is scarce data that describe these infections in Colombia, especially reports from community and healthcare-acquired infections and their impact on clinical outcomes. Thus, this project aims to bridge this gap in the literature. We used a comprehensive clinical and molecular approach to describe the genotypic characteristics of carbapenemase-producing Gram-negative rods in Colombia, an LMIC. We hypothesize that the description of the carbapenemase-encoding genes in these bacteria may vary, particularly in healthcare and community settings.

## RESULTS

**Fig 1:**
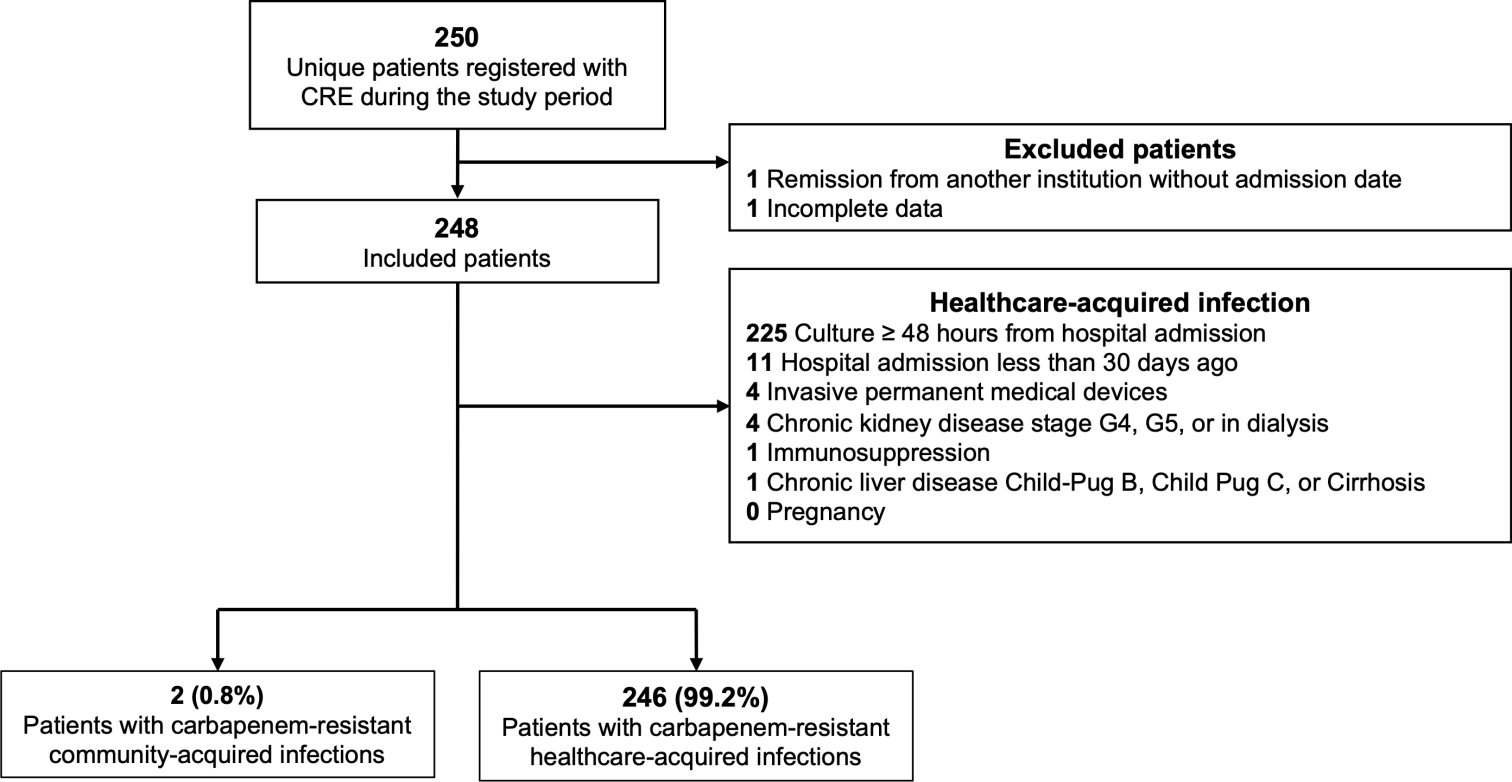
Study flowchart.

A total of 248 patients were included in the study, all with single bacterial isolation (Fig. 1). Most of the patients were male [71.4% (177/248)], and the median (interquartile range, IQR) age was 61.5 years old (47.0–72.3). The main identified comorbidities were arterial hypertension [50.0% (124/248)], diabetes mellitus [25.4% (63/248)], smoking history [26.2% (65/248)], and obesity [20.2% (50/248)]. Regarding treatments and interventions, more than half of the patients required invasive mechanical ventilation [61.3% (152/248)]. Also, two-thirds were admitted to the intensive care unit (ICU) [76.2% (189/248)] with a prolongated median (IQR) ICU length of stay (LOS) [25.0 days (14.0–36.0)]. Finally, mortality was reported in more than a third of the patients [33.9% (84/248)]. All patients’ baseline characteristics are shown in Table 1.

**TABLE 1:** Demographic characteristics of all patients and stratification between infection acquisition

	General cohort (*n* = 248)	Community acquired (*n* = 2)	Healthcare acquired (*n* = 246)
Demographic characteristics			
Age, median (IQR)	61.5 (47.0–72.3)	75.0 (68.5–81.5)	61.0 (47.0–72.0)
Male, *n* (%)	177 (71.4)	0 (0.0)	177 (72.0)
Charlson index, median (IQR)	3.0 (1.0–5.0)	3.5 (2.8–4.3)	3.0 (1.0–5.0)
Comorbid condition, *n* (%)			
Smoking history	65 (26.2)	0 (0.0)	65 (26.4)
Alcoholism	17 (6.9)	0 (0.0)	17 (6.9)
Chronic obstructive pulmonary disease (COPD)	45 (18.1)	1 (50.0)	44 (17.9)
Diabetes mellitus	63 (25.4)	0 (0.0)	63 (25.6)
Hypertension	124 (50.0)	0 (0.0)	124 (50.4)
Obesity	50 (20.2)	0 (0.0)	50 (20.3)
Congestive heart failure	35 (14.1)	0 (0.0)	35 (14.2)
Coronary disease	28 (11.3)	0 (0.0)	28 (11.4)
Atrial fibrillation	20 (8.1)	0 (0.0)	20 (8.1)
Dyslipidemia	32 (12.9)	0 (0.0)	32 (13.0)
Liver disease	5 (2.0)	0 (0.0)	5 (2.0)
Chronic kidney failure	47 (19.0)	0 (0.0)	47 (19.1)
Autoimmune disease	12 (4.8)	0 (0.0)	12 (4.9)
Cancer	11 (4.4)	0 (0.0)	11 (4.5)
HIV	3 (1.2)	0 (0.0)	3 (1.2)
Epilepsy	11 (4.4)	0 (0.0)	11 (4.5)
Previous hospital admission	54 (21.8)	0 (0.0)	54 (22.0)
Previous use of antibiotic	43 (17.3)	0 (0.0)	43 (17.5)
Vital signs at admission, median (IQR)			
Heart rate	85.0 (75.0–102.0)	87.5 (85.8–89.3)	86.0 (75.0–102.0)
Respiratory rate	19.0 (18.0–22.0)	17.5 (17.3–17.8)	19.0 (18.0–22.0)
Systolic blood pressure	120.0 (107.0–133.0)	143 (128.0–158.0)	120.0 (107.0–133.0)
Diastolic blood pressure	74.0 (65.0–80.0)	64.5 (55.3–73.8)	74.0 (52.0–80.0)
Mean arterial pressure	86.0 (75.0–96.0)	94 (81.0–107.0)	86.0 (75.0–96.0)
Temperature	36.4 (36.0–36.5)	36.3 (36.1–36.4)	36.4 (36.0–36.5)
Saturation	92.0 (87.0–94.0)	92.0 (91.0–93.0)	92.0 (87.0–94.0)
Glasgow	15.0 (13.0–15.0)	14.5 (14.3–14.8)	15.0 (13.0–15.0)
Laboratory testing at admission, median (IQR)			
Leucocytes	10.1 (7.3–14.5)	18.3 (14.3–22.3)	10.1 (7.2–14.4)
Hemoglobin	13.5 (10.4–15.5)	8.5 (7.8–9.1)	13.6 (10.5–15.5)
Blood urea nitrogen	22.0 (17.0–36.0)	106 (84.0–128.0)	22.0 (17.0–35.0)
Creatinine	1.0 (1.0–2.0)	6.7 (4.9–8.5)	1.0 (0.8–1.7)
Sodium	138.0 (135.0–141.0)	149 (143.0–155.0)	138.0 (135.0–141.0)
Potassium	4.3 (3.9–4.9)	6.5 (6.4–6.5)	4.3 (3.9–4.8)
Reactive C protein	100.0 (32.0–100.0)	NA^*a*^	100.0 (32.0–100.0)
Severity scores, median (IQR)			
Sequential organ failure assessment (SOFA) at admission	4 (3.0–6.0)	NA	4 (3.0–6.0)
Acute physiology and chronic health evaluation disease classification system (APACHE) at admission	12 (8.0–18.5)	NA	12 (8.0–18.5)
SOFA at isolation	6 (3.0–9.0)	NA	6 (3.0–9.0)
APACHE at isolation	16 (8.0–21.0)	NA	16 (8.0–21.0)
Treatments and interventions			
Invasive mechanical ventilation, *n* (%)	152 (61.3)	0 (0.0)	152 (61.8)
IMV days, median (IQR)	19.5 (11.0–28.0)	NA	19.5 (11.0–28.0)
Vasopressor, *n* (%)	154 (62.1)	0 (0.0)	154 (62.6)
Inotropic, *n* (%)	81 (32.7)	0 (0.0)	81 (32.9)
Antibiotics days, median (IQR)	14.0 (10.0–17.0)	6.0 (5.5–6.5)	14.0 (10.0–17.0)
Outcomes, *n* (%)			
Admission to the intensive care unit	189 (76.2)	1 (50.0)	188 (76.4)
Hospital LOS, median (IQR)	33.0 (19.0–57.0)	7.5 (6.3–8.8)	33.5 (19.0–57.0)
ICU LOS, median (IQR)	25.0 (14.0–36.0)	2.0 (2.0–2.0)	25.0 (14.0–36.0)
In-hospital mortality	84 (33.9)	1 (50.0)	83 (33.7)
	**General cohort (*****n*** **= 228)**	**Community acquired (*****n*** **= 2)**	**Healthcare acquired (*****n*** **= 226)**
Alleles according to WGS, *n* (%)			
*bla*KPC-2	88 (38.6)	0 (0.0)	88 (38.9)
*bla*KPC-3	83 (36.4)	1 (50.0)	82 (36.3)
*bla*OXA-152	1 (0.4)	0 (0.0)	1 (0.4)
*bla*OXA-244	1 (0.4)	0 (0.0)	1 (0.4)
*bla*VIM-2	29 (12.7)	0 (0.0)	29 (12.8)
*bla*VIM-24	3 (1.3)	0 (0.0)	3 (1.3)
*bla*GES-5	1 (0.4)	0 (0.0)	1 (0.4)
*bla*NDM-1	16 (7.0)	0 (0.0)	16 (7.1)
NA	31 (13.6)	1 (50.0)	30 (13.3)

^*a*^ Not applicable.

Most patients had healthcare-acquired infections [99.2% (246/248)]. Those with community-acquired infections were [median (IQR)] older [CA: 75.0 years (68.5–81.5); HA: 61.0 (47.0–72.0)] and had a higher [median (IQR)] Charlson index [CA: 3.5 (2.8–4.3); HA: 3.0 (1.0–5.0)]. At admission, although patients in both groups had similar vital signs and paraclinical data, those with community-acquired infections had higher leucocytes [CA: 18.3 (14.3–22.3); HA: 10.1 (7.2–14.4)] and creatinine levels [6.7 (4.9–8.5); 1.0 (0.8–1.7)]. Notably, ICU admission was notoriously higher among those with healthcare-acquired infections [CA: 50.0% (1/2); HA: 76.4% (188/246)], but not in-hospital mortality [CA: 50.0% (1/2); HA: 33.7% (83/246)] (Table 1).

**Fig 2:**
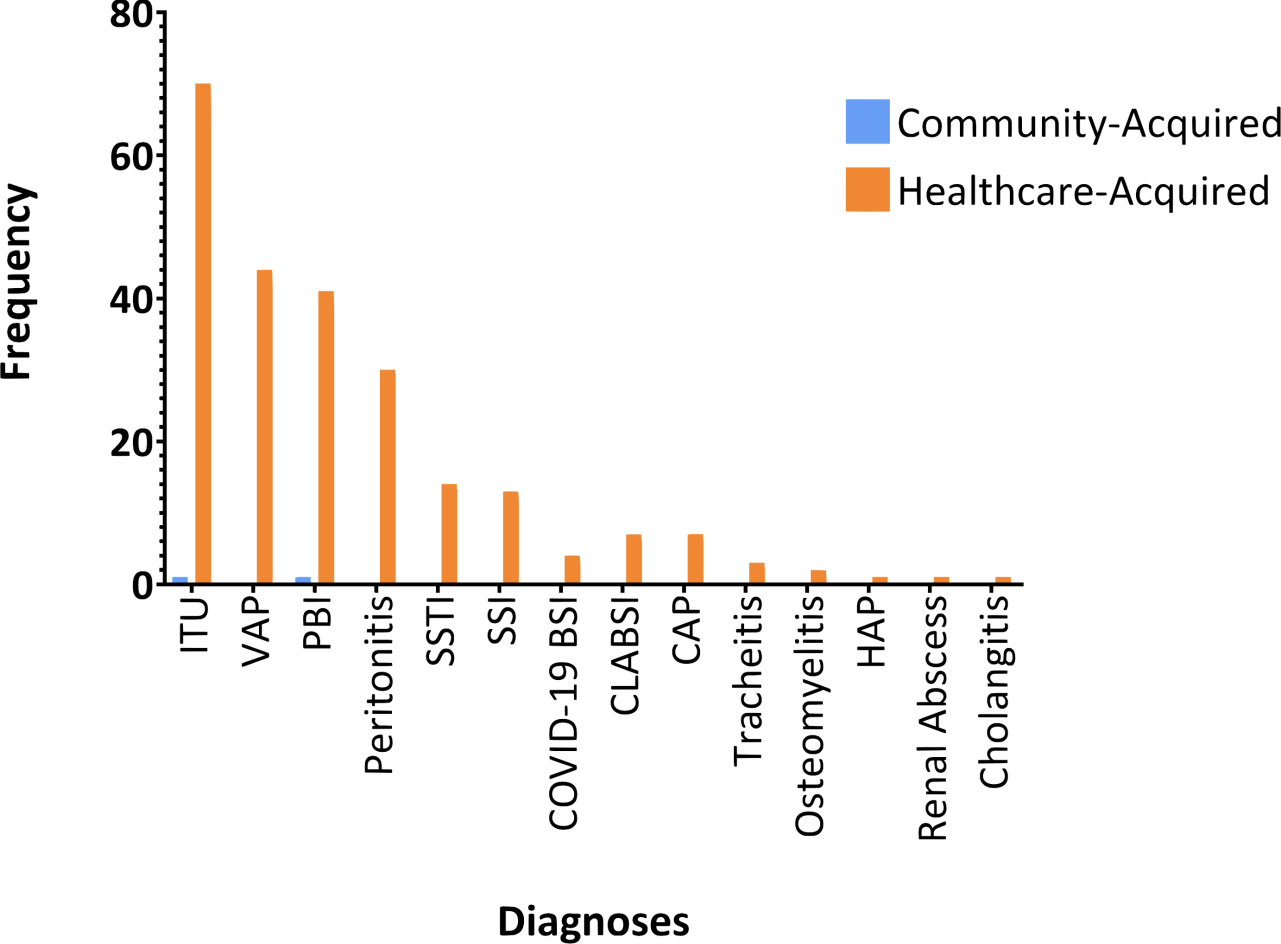
Patients’ diagnoses. Distribution of patients’ diagnoses is shown: those with community-acquired infections (blue) and healthcare-acquired infections (orange). UTI, urinary tract infection; VAP, ventilator-associated pneumonia; PBI, primary blood infection; SSTI, skin and soft tissue infection; SSI, surgical site infection; BSI, bacterial superinfection; CLABSI, central line-associated bloodstream infection; CAP, community-acquired pneumonia; and HAP, hospital-acquired infection.

In this study, the comprehensive analysis of clinical diagnoses revealed that the predominant infections were urinary tract infections [28.6% (71/248)], ventilator-associated pneumonia [17.7% (44/248)], primary bloodstream infection [16.9% (42/248)], and peritonitis [12.1% (30/248)] (Fig. 2). Regarding the community-acquired infections, these were either urinary tract infections [50.0% (1/2)] or primary bloodstream infections [50.0% (1/2)], while healthcare-acquired infections were dominated by urinary tract infections [28.5% (70/246)] and ventilator-associated pneumonia [17.9% (44/246)] (Fig. 2).

The initial bacterial identification was done at each institution by Vitek. Subsequently, these were recognized by WGS to determine the pathogen’s species accurately. Although all isolates were identified as carbapenem-resistant, during the genetic characterization using WGS, seven isolates did not pass quality control. In 13, a known carbapenemase gene was not identified. Consequently, 228 bacterial isolates were included in the WGS analysis. As expected, some discrepancies were observed among the identification strategies used (Vitek and WGS). For instance, 17 distinct species were represented by Vitek and 34 by WGS (Table S1). However, for both methods, the most prevalent species identified were *P. aeruginosa* [Vitek: 35.1% (87/248); WGS: 38.2% (87/228)] and *Klebsiella pneumoniae* [Vitek: 36.3% (90/248); WGS: 28.5% (65/228)] (Table S1).

### Genetic analysis by WGS

**Fig 3:**
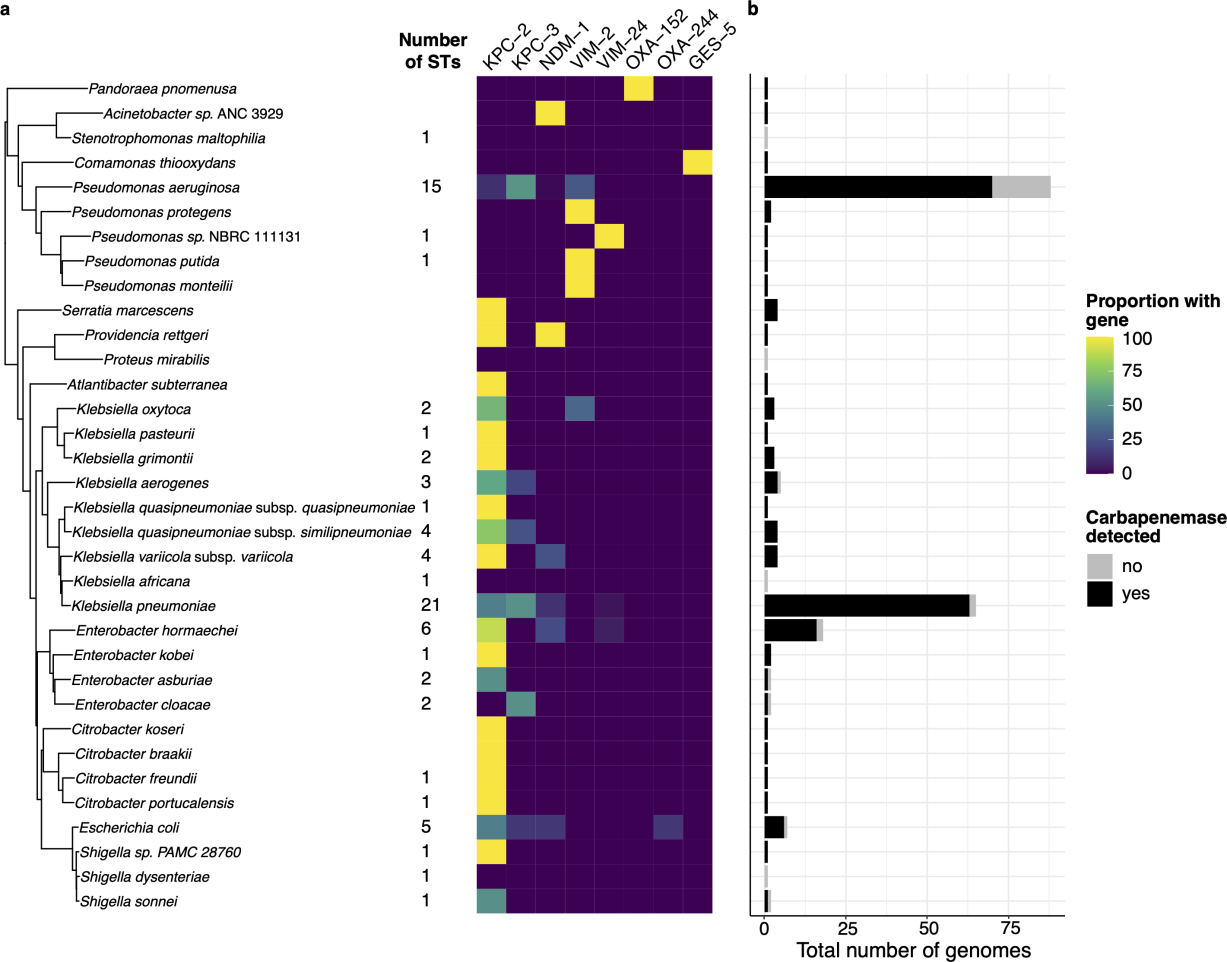
WGS identification. (a) Phylogenetic tree (midpoint rooted) showing the relationship of all species detected in WGS (one representative per species), the number of STs detected is shown next to the tree, and heatmap that shows the proportion of genomes carrying a particular carbapenemase gene identified by WGS. (b) Bar chart indicating the total number of genomes per species that either carried a carbapenemase gene (black) or not (gray).

A total of 228 isolates passed all quality checks for WGS analysis. Information regarding the susceptibility to aminoglycosides, colistin, fosfomycin, fluoroquinolones, rifampicin, sulfonamides, tetracyclines, trimethoprim, beta-lactamases, extended-spectrum beta-lactamases (ESBLs), SHV gene mutations, Omp gene mutations, colistin mutations, and fluoroquinolone mutations can be found in Table S2. Respecting the identified genes related to carbapenem resistance, the leading genotypic group was *bla*KPC, specifically, *bla*KPC-2 [38.6% (88/228)] and *bla*KPC-3 [36.4% (83/228)] as the predominant alleles. These results were followed by *bla*VIM-2 [12.7% (29/228)] and *bla*NDM-1 [7.0% (16/228)]. Carbapenemase-encoding genes *bla*OXA-152 [0.4% (1/228)], *bla*OXA-244 [0.4% (1/228)], and *bla*GES-5 [0.4% (1/228)] were the least prevalent identified. Notably, the only identified allele among community-acquired infections was *bla*KPC-3 [50.0% (1/2)]. All WGS results are shown in Fig. 3 and Table 1.

It is essential to clarify that in 31 confirmed carbapenemase-producing genomes, we could not detect carbapenemase-encoding genes. The majority of these were *P. aeruginosa* [58.0% (18/31)], representing 20.7% of all *P. aeruginosa* isolates. This phenomenon was also observed in smaller proportions with *K. pneumoniae*, *Enterobacter hormaechei*, *Escherichia coli*, and *Shigella sonnei* (Fig. 3b).

Isolates with co-occurring carbapenemase genes were detected in 9.6% (22/228) of genomes (Table S3). A total of 20 microorganisms had two carbapenem resistance genes, these being *P. aeruginosa* [55.0% (11/20)], *K. pneumoniae* [20.0% (4/20)], *E. hormaechei* [15.0% (3/20)], *Klebsiella variicola* [5.0% (1/20)], and *P. rettgeri* [5.0% (1/20)]. Contrastingly, only two isolates expressed three carbapenem resistance genes, both being *K. pneumoniae* [100.0% (2/2)]. Regarding co-occurring alleles, 10 were *bla*KPC-3 + *bla*VIM-2 [*P. aeruginosa*: 100.0% (10/10)], 9 were *bla*KPC-2 + *bla*NDM-1 [*K. pneumoniae*: 44.4% (4/9), *E. hormaechei*: 33.3% (3/9), *Klebsiella variicola* sub sp. *variicola*: 11.1% (1/9), and *Providencia rettgeri*: 11.1% (1/9)], 2 were *bla*KPC-2 + *bla*NDM-1 + *bla*VIM-24 [*K. pneumoniae*: 100.0% (2/2)], and 1 *bla*KPC-3 + *bla*NDM-1 [*P. aeruginosa*: 100.0% (1/1)] (Table S4).

Multi-locus sequence type (MLST) schemes were available for 23 of the 34 species identified in the WGS analysis, and 78 distinct STs were identified. A total of 56 were represented by only one isolate each, indicating a high diversity of carbapenemase-producing Gram-negative rods. However, eight STs appeared to be overrepresented and were identified among ≥5 genomes each. Regarding *P. aeruginosa,* these were ST111 [65.5% (57/87)] and ST235 [13.8% (12/87)]. For *K. pneumoniae* ST258 [15.4% (10/65)], ST14 [10.8% (7/65)], ST219 [10.8% (7/65)], ST236 [10.8% (7/65)], and ST1082 [7.7% (5/65)]. Finally, for *E. hormaechei,* only ST175 [52.9% (9/17)] (Table S2).

**Fig 4:**
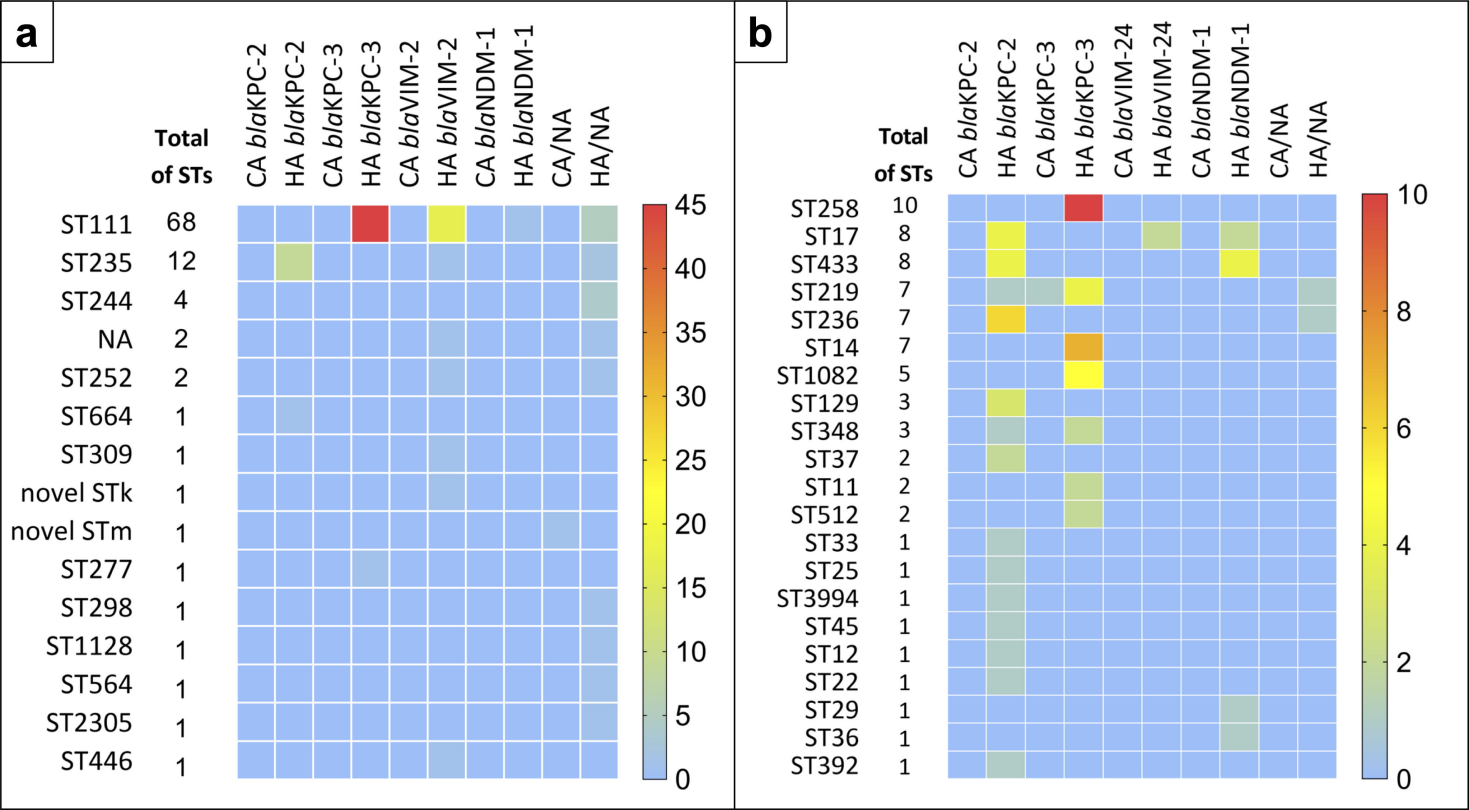
Gene correlation with STs of *Pseudomonas aeruginosa* and *Klebsiella pneumoniae*. (a) Heatmap shows the proportion of genes associated with the different STs of *Pseudomonas aeruginosa.* (b) Heatmap shows the proportion of genes associated with the different STs of *Klebsiella pneumoniae*. CA, community-acquired; HA, healthcare-acquired; and NA, non-detected.

The distribution of carbapenemase genes among STs was explored for the most frequently identified pathogens, *P. aeruginosa* and *K. pneumoniae*. Results were separated according to the source of infection (community-acquired and healthcare-acquired). Isolates carrying more than one carbapenemase gene were counted once for each gene. With respect to *P. aeruginosa*, ST111 was primarily associated with *bla*KPC-3 and *bla*VIM-2; both were related to healthcare-acquired infections. Also, two novel *P. aeruginosa* STs were identified; notably, one was recognized in a community-acquired infection (Fig. 4a). For *K. pneumoniae*, 14 STs were associated with *bla*KPC-2, all from healthcare-acquired infections. *bla*KPC-3 was associated exclusively with healthcare-acquired ST512 [100.0% (2/2)], ST258 [100.0% (10/10)], ST14 [100.0% (7/7)], and ST1082 [100.0% (5/5)] (Fig. 4b). Only ST219 was related to community-acquired infections [16.7% (1/6)].

We sought to understand the relationship between isolates in our collection and those from other geographies represented in public genome collections. We focused on the three STs for which ≥10 genomes were available in our collection (*P. aeruginosa* ST111 and ST235, *K. pneumoniae* ST258) and used maximum likelihood phylogenetic analyses to compare them to public genomes of the same ST identified via the Pathogenwatch genomic surveillance platform (https://pathogen.watch/; see Table S5 and Materials and Methods). The *P. aeruginosa* ST111 genomes from our collection formed three clusters in the phylogeny (Fig. S1), consistent with at least three independent introductions. All three clusters fell within a large sub-clade that was not well-resolved in the phylogeny and was dominated by genomes from Europe and North America carrying *bla*VIM carbapenemases; however, this was unsurprising given the overwhelming bias toward these regions among the public ST111 genomes. Nine of our *P. aeruginosa* ST235 genomes formed a cluster in the phylogeny, whereas the remaining three appeared unrelated to each other. The cluster of nine genomes was most closely related to a subclade comprising genomes from diverse geographic regions, but primarily Asia and North America, and mostly without any detectable carbapenemases (Fig. S2). All our *K. pneumoniae* ST258 genomes were clustered together in the phylogeny and sister clade to a group dominated by genomes from North America (Fig. S3). As is characteristic of ST258, most genomes carried *bla*KPC carbapenemase alleles. In all cases, it is difficult to draw conclusions about the potential origins of the isolates in our collection, due to the vast overrepresentation of public data from Europe and North America and the lack of representation of other sites in Colombia or, indeed, many South American countries.

### Clinical impact

Bivariate analysis between the different alleles identified by WGS. These showed no association or difference with hospital mortality (*P* = 0.83), ICU admission (*P* = 0.52), vasopressor requirement (*P* = 0.61), inotropic requirement (*P* = 0.65), or mechanical ventilation requirement (*P* = 0.25) (Table S5).

## DISCUSSION

This cohort of patients infected with carbapenemase-producing Gram-negative rods is novel in describing the molecular characteristics and epidemiological profile of a large cohort in an LMIC in South America. The most common isolated bacteria were *P. aeruginosa* and *K. pneumoniae*, and the primary detected carbapenemase-encoding gene was *bla*KPC, alleles *bla*KPC-2 and *bla*KPC-3. Also, co-occurrence was frequently detected. Moreover, a small number of ST-carbapenemase combinations were overrepresented among healthcare-acquired infections, which may indicate transmission within the hospital setting. These results are essential to generate recommendations to treat patients with these infections in LMICs.

The prevalence of AMR appears notably elevated in the context of hospital-acquired infections (17). This correlates with our results, where most isolates originated from a hospital setting (99.2%). In contrast, a 2022 population-based surveillance conducted in the United States revealed a higher proportion, with 10% of carbapenem-resistant Enterobacterales identified as community-acquired (18). These disparities in prevalence can be attributable to variations in the definitions employed or potential biases within the cohort selection process, which underscores the importance of standardizing definitions and ensuring accuracy in their application. Furthermore, the epidemiological profile of our cohort was similar to the worldwide description of carbapenem resistance, *P. aeruginosa* and *K. pneumoniae* being the most prevalent bacteria (19, 20). A 2022 global systematic review identified these pathogens as part of the six most resistant microorganisms associated with increased mortality (1, 21, 22). *Enterobacter* spp*.* was also a commonly isolated species in our study, which is considered an emerging global threat and has increased in Colombia during the last decade (23, 24). Unlike other countries, resistant *Acinetobacter baumannii* was not a frequently identified pathogen in our study. In the United States, it caused 22% of 29,742 reported infections between 2009 and 2013 (25). These advocate that LMICs’ epidemiology has differences that must be studied further.

The principal identified carbapenemase-encoding gene in our study was Class A *bla*KPC, which is in alignment with American and Latin American epidemiology (26, 27). In our cohort, *bla*KPC-2 and *bla*KPC-3 were the most frequently detected alleles in healthcare-acquired infections. *bla*KPC-2 is a commonly found resistance gene in Colombia. Latest studies have reported an increase in *P. aeruginosa* and *Enterobacter cloacae*, possibly mediated by plasmid transmission. Regarding *bla*KPC-3 infections, these have been described in Colombia since 2011 (28), and different cities still struggle with in-hospital outbreaks (29, 30). Notably, previous outbreaks associated with *bla*KPC-3 *K. pneumoniae* ST258 have been reported (29), our most common ST in this pathogen. Moreover, *P. aeruginosa* ST111 has been closely related to *bla*VIM-2 (31–33); however, in our study, its primary production was *bla*KPC-3, which is uncommon and has not been published before. These findings open a possibility of gene clonality in the healthcare environment either through an inter-species transfer of antimicrobial resistance plasmids in combination with local clonal expansion, which is the subject of ongoing analyses in our team, or patient-to-patient or from a reservoir within the hospital (e.g., contaminated equipment or sink drains), as have been implicated previously for these organisms (34–36).

Carbapenem resistance is known to have a negative effect on clinical outcomes, especially in LMICs (16, 37). Despite this, our results demonstrate that the different carbapenemase-producing Gram-negative rods and carbapenemase-encoding genes have similar effects on clinical endpoints. This could be in relation to the different treatment recommendations based on the etiological agent and/or its resistance mechanism (38). For instance, ceftolozane-tazobactam is a new antimicrobial against resistant *P. aeruginosa and K. pneumoniae;* however, it has no activity for class A serine carbapenemases or class B metallo-β-lactamases (39–41). Ceftazidime-avibactam and meropenem/vaborbactam are novel antibiotics active against OXA and AmpC and class A carbapenemases producing Enterobacterales, respectively (42–45). These have a higher reported effectivity than aminoglycosides and colistin (46–48). In a systematic review, Soriano et al*.* (47) also described that *bla*KPC resistance secondary to ceftazidime-avibactam use is infrequent. Since the principal carbapenemase gene in our population was *bla*KPC, we hypothesize that using these new treatments may reduce healthcare expenses and disease burden and improve clinical outcomes. Nevertheless, *bla*KPC co-occurrence with *bla*VIM and *bla*NDM was frequently identified in our study and could bring challenging implications regarding antimicrobial treatments.

Our study has limitations and strengths that are important to recognize. Our comprehensive approach has highlighted the burden of carbapenem-resistant infections in our setting and the extensive diversity of the underlying pathogens associated with community-acquired and healthcare-acquired infections. However, these results reflect the current state of carbapenemase-producing Gram-negative rods only in two Colombian centers, and, therefore, we cannot extrapolate the specific trends to other countries and/or regions. Second, we did not have data from the last 2 years or a sufficient sample size to detect statistical differences between the community-acquired and healthcare-acquired pathogen populations in terms of species and ST distributions. Third, most isolates in our study meet a definition for acquisition in the hospital environment, and the dissemination of carbapenem-resistant Gram-negative rods in the community is an aspect we could not elucidate. For future studies, different methodological approaches could be addressed, such as evaluating fecal carriage in well-selected samples of healthy subjects in the community (49) or evaluating an exclusive cohort of community-acquired isolates (50). Finally, we did not have access to information regarding the antimicrobial treatment of the patients or their prior contact with agriculture or livestock, which could have been valuable to provide results in the context of antimicrobial resistance. Nonetheless, our data highlight key aspects of carbapenem-resistant Gram-negative infections that should motivate similar works in other LMICs.

In conclusion, carbapenem resistance is a rising problem in LMICs such as Colombia. The most common carbapenemase-producing Gram-negative rods were *P. aeruginosa* and *K. pneumoniae*. Moreover, *bla*KPC-2 and *bla*KPC-3 were the most frequent carbapenemase-encoding genes related to gene co-occurrence and diverse ST in the healthcare environment. Notably, these patients had several systemic complications and poor clinical outcomes without being associated with a particular gene. These data highlight the need for better prevention, diagnosis, and management of multidrug-resistant infections in LMICs and globally.

## MATERIALS AND METHODS

This was a multicenter, retrospective cohort study conducted in Colombia between July 2017 and July 2021. It was performed in hospitalized patients infected with carbapenem-resistant Gram-negative rods, which could have been acquired in the community or the healthcare facility. Bacterial pathogens were identified using biochemical profiling during clinical practice. For this analysis, investigators collected all the clinical information by chart review. All the clinical data, laboratory results, and microbiological isolates were collected using the Research Electronic Data Capture (REDCap) electronic data collection form hosted by the Universidad de La Sabana. Informed consent was waived since this retrospective study used chart reviews to extract clinical data and bacteria kept for epidemiological vigilance in the microbiology labs.

### Setting

This study was carried out in two reference hospitals in Colombia. The Clínica Universidad de La Sabana, a trauma reference academic health center located in the surroundings of Bogotá, Colombia and the Fundación Clínica Shaio, a cardiovascular reference hospital in Bogotá, Colombia.

### Study population and data collection

All patients older than 18 years old hospitalized during the study period with a Gram-negative rod infection, with at least one carbapenem resistance in the antibiogram, were included. Isolates from patients without documented active infection (colonization) were excluded. The study’s definitions for infection and colonization can be found in the supplemental material. Patients who were transferred from another healthcare institution without available information regarding their previous hospitalization, those identified as duplicates, and individuals with more than one concomitant bacterial isolate were excluded from the study.

A community-acquired infection was defined as a direct admission from home, with a positive carbapenem-resistant culture documented within 48 h after admission and none of the following: (i) hospitalization within 30 days prior to admission, (ii) previous residency in a long-term care facility within 30 days prior to admission, (iii) chronic kidney disease stage G4, G5, or in dialysis, (iv) chronic liver disease Child-Pug B, Child-Pub C, or cirrhosis, (v) actively immunocompromised [i.e., chronic use of oral corticosteroids, chemotherapy, and acquired immunodeficiency syndrome), (vi) active cancer, (vii) pregnancy, or (viii) permanent use of a medical device (i.e., permanent bladder catheter, tracheostomy, nasogastric tube, and subclavian catheter) (Fig. 1).

The following variables were gathered in the data set: age, sex, past medical history (comorbidities), clinical diagnosis, treatments (medications), physiological and paraclinical variables, severity scores [Sequential Organ Failure Assessment (51) and Acute Physiology and Chronic Health disease Classification System (52)], LOS, ICU admission, days of antibiotic therapy, days of mechanical ventilation, ventilator support, vasopressor and inotropic support, and mortality. Physiological and paraclinical variables were collected during the first 24 h of admission and at the time of the bacterial isolation. All the study’s definitions are in the supplemental material.

### Sample size estimation

Taking a population size of 2,000 isolations, the proportions of carbapenem-resistant Gram-negative rods expected was 50% due to our country’s unknown prevalence of carbapenem resistance (not representative data). The confidence level assumed was 95%, with a design effect of 1 and a 6% precision. A minimum requirement of 236 bacteria was needed to have the mentioned confidence level that the real value is within ±6% of the isolates’ value.

### Bacterial isolates and procedures

Samples were taken according to the “Sample Collection Manual” of each institution. Both protocols can be found in the supplemental material. The identification of all the bacterial isolates and the antibiotic susceptibility testing were initially done by broth culture with Vitek identification according to the manufacturer’s instructions. Antibiotic resistance profiles were performed using a subculture of the identified strain in thioglycolate broth and incubated at 37°C for 24 h. Subsequently, the suspension was plated on a MacConkey agar plate and incubated at 37°C for 18–24 h. To detect carbapenem resistance, phenotypic tests and immunoassays were performed, such as the modified Hodge test, boronic acid sensi-discs synergy test, ethylenediaminetetraacetic acid discs synergy test, RESIST-4–O.K.N.V lateral flow immunoassays, and NG-Test CARBA 5 lateral flow immunoassay (53). All tests were performed consecutively by the same microbiology laboratory personnel, using the Clinical and Laboratory Standards Institute methods. The specific protocols for each test have been published elsewhere and can be found in the supplemental material (53).

The bacterial DNA extraction was done using the Qiagen DNeasy️ Blood & Tissue extraction kit, following the manufacturer’s instructions and subsequently tested in the NanoDrop to determine DNA quality; the cut-off point was 50 (ng/µL). Then, isolates were subjected to whole-genome sequencing. The sequencing libraries were generated using the Nextera DNA Flex kit (Illumina Inc.) and sequenced on the Illumina NovaSeq to create 150 bp paired-end reads. WGS data were used to confirm species identifications and identify 7-gene MLST and carbapenemase alleles (see supplemental material for full methodological details).

During the genetic characterization using WGS, 7 isolates did not pass quality control, and in 13, a known carbapenemase gene was not identified, and subsequent phenotypic re-testing revealed that the isolate received by the sequencing laboratory was not carbapenem-resistant (Table S6), suggesting that either (i) the original culture may have been mixed (one or more carbapenemase-producing strains plus one or more carbapenem-susceptible strains); (ii) the isolate had lost a resistance plasmid during culture; or (iii) a labeling error had occurred. Unfortunately, we could not determine which of these possibilities was most likely. Therefore, these 20 isolates were excluded from further analyses.

### Statistical analysis

Data with missing values of less than 30% were imputed. A descriptive analysis was performed based on qualitative variables presented with percentages and absolute frequencies and continuous variables with median (interquartile range) or mean (standard derivation) according to their distribution. Then, normally distributed continuous data were tested using Welch’s two-sample *t*-tests for data from two groups and ANOVA for three or more groups. When the data follow a non-parametric distribution, the Mann-Whitney *U* was used to compare differences between two groups or the Kruskal-Wallis tests for three or more groups. Qualitative variables were performed by chi-squared to assess the association. Statistical significance was chosen at 5%. Analyses were conducted in R Studio (R Foundation for Statistical Computing, Vienna, Austria) or SPSS 25 (StataCorp LLC, TX, USA).

## Data Availability

All the genomes are located in the following repository: PRJNA937403. All the data can be found in the supplemental material and Tables S1 to S6.

## SUPPLEMENTAL MATERIAL

Sample collection protocol spectrum.01714-23-s0001.pdf Sample collection manual for Shaio Clinic Foundation.

Sample collection protocol spectrum.01714-23-s0002.pdf Sample collection manual for Clinica Universidad de La Sabana.

Supplemental material spectrum.01714-23-s0003.docx Supplemental text, tables, and figures.

Supplemental table spectrum.01714-23-s0004.xlsx Table S2.

ASM does not own the copyrights to Supplemental Material that may be linked to, or accessed through, an article. The authors have granted ASM a non-exclusive, world-wide license to publish the Supplemental Material files. Please contact the corresponding author directly for reuse.
