# Supplementary material for: Molecular characterization and descriptive analysis of carbapenemase-producing Gram-negative rod infections in Bogota, Colombia
Source: Microbiol Spectr. 2024 Apr 17;12(6):e01714-23. doi: 10.1128/spectrum.01714-23 (PMC11237484; doi:10.1128/spectrum.01714-23)
Supplement: Sample collection protocol — Sample collection manual for Shaio Clinic Foundation. [file spectrum.01714-23-s0001.pdf]

ENGLISH VERSION
(TRANSLATED)

Machine Translated by Google

VERSION	DATE	DESCRIPTION OF THE MODIFICATION
7	02/24/2022	Phlebitis is included in the complications due to venipuncture.
6	05/29/2020	The marking of the blood sample tubes with the initials of the patient's full name is included, leaving them visible to be able to verify the data on the sticker generated by the laboratory system.<br>A sampling process is added when it is necessary to extract blood from the same extremity used for the intravenous administration of medications or liquids.<br><br>Information is added on patient position and proper use of the tourniquet during sample taking.<br>venous.<br>Information on the use of microtainer tubes is added.<br>Information about taking Glucometries was added to the *Accu-Chek Inform II system.* Reference values were updated.<br>Microbiology sampling protocol is updated.<br><br>Biosafety section is included in taking blood samples.<br><br>Covid-19 is included in section 15.0 Bases for interpretation of laboratory sample results and in section 16.6 General laboratory examinations and required samples.
5	MAY 1, 2016	The taking of blood cultures is modified due to the change of bottles due to the change in technology.
4	07/14/15	Update of activities, taking microbiology samples.
3	10-Mar-11	Update of activities, including reporting time table and patient preparation conditions
2	03/10/10	Update of activities related to taking samples in outpatient consultation

Machine Translated by Google

**Elaborated:** Isabel Cristina Torres	**Updated:** Paula Andrea Ramos	**Revised:** Isabel Cristina Torres	**Approval:** Gilberto Mejia Estrada, MD.
**Position:** Coordinator Clinical laboratory	**Position:** Bacteriology	**Post:** Clinical laboratory director	**Position:** Scientific director.
**Date:** December 14, 2009	**Date:** February 2022	**Date:** February 2022	**Date:** February 2022

1.0 GENERAL OBJECTIVE

Carry out sample collection in an effective and timely manner in the Emergency Services, hospitalization and outpatient consultation, complying with the requirements and guidelines established for this purpose in the laboratory department of the Shaio Clinic Foundation, ensuring the quality of the sample.

2.0 SCOPE

This procedure applies from the moment the request for examinations is generated in the clinical history information system, until the delivery of the samples to the laboratory, whether they are taken laboratory). in the , on the floor by the laboratory staff (bacteriological and/or auxiliary central established rounds, or by the nurses in charge of the patient.

3.0 DEFINITIONS

ASEPSY: A set of procedures that considerably reduce the proliferation and spread of pathogenic microorganisms.

STANDARDIZATION: Development and implementation in a uniform and agreed manner of technical specifications, criteria, methods, processes and practices that can increase quality, reproducibility and safety of care.

HEMOLYSIS: Phenomenon of the disintegration of erythrocytes.

CENTRAL **LABORATORY :** Clinical laboratory in charge of processing all laboratory tests of the Shaio Clinic Foundation.

ORDER: Request for laboratory tests completed by the treating physician on each floor and generated directly in the information system.

VENIPUNCTION: Venipuncture is the extraction of blood from a vein, performed by health personnel. It is also known as venipuncture.

Machine Translated by Google

4.0 RESPONSIBLE

Treating physician
Head Nurse
Assistant Billing.
Laboratory assistant
Bacteriologist

5.0 DESCRIPTION

The clinical laboratory is in charge of taking samples in the hospitalization service (except pediatric ICU and adult ICU roundabout), for which it has organized sample taking by rounds at the following times:

05:00 a.m. – 7:00 a.m.
12:00 p.m.
6:00 p.m.
24:00

For an order to be included in the round, it must be loaded into the Medical History system at least 1 **hour before** the round, otherwise it will be left for the next round.

Sample orders generated as urgent at times other than rounds must be taken and sent by the nurses on the floor.

Blood culture sampling is not governed by rounds, but at the request of the treating physician.

6.0. SPECIFIC OBJECTIVES:

Raise staff awareness of the importance of proper collection, handling, transportation and conservation of samples, to:

- Ensure a safe and patient-centered procedure.
- Standardize the sampling process.
- Prevent errors in sample collection so that clinical decisions based on the results of laboratory tests are correct.

7.0. GENERAL RECOMMENDATIONS

7.1. PROCEDURE FOR TAKING SAMPLES

Machine Translated by Google

ü Rigorously verify the patient's name and the examinations with the medical order. take.

ü Verify the type of tubes to be used before taking the sample. It is important to confirm with the medical order and the examination the type of tube, quantity of the sample (pediatric patients) and specific conditions for handling the samples.

ü Introduce yourself to the patient with name, position and explain your role in care.

ü Corroborate the patient's Identification, performing cross-verification, explaining the procedure and preparation if required.

ü Ask the patient about a history of allergies and medication intake, especially, anticoagulants.

ü Confirm that the patient meets the previous preparation conditions necessary for sample collection according to the requested examination.

ü Hands should be clean to minimize the risk of transmitting infections during extraction, a new pair of gloves should always be used to protect the patient and the staff taking the sample.

ü Preserve aseptic technique when obtaining samples through invasive procedures (peripheral venipuncture, central catheter, lumbar puncture, etc.) ü The patient must be in a comfortable position. ü If you have difficulty taking it, ask for help from another bacteriologist, nurse or doctor. nursing assistant.

ü Label the tubes or containers with the patient's data, in front of them corroborating the name and identification number.

ü For adequate identification, at least two identifiers must be used (full name and identification number, at the Shaio Clinic Foundation the admission number is used), the patient's identity must be compared with the data on the sticker.

ü The blood sample tubes, once taken and in the presence of the patient, must be marked with the initials of the patient's full name (written with a marker on the label that comes with the tube) and these must remain visible to be able to verify the barcode information. generated by the laboratory system.
This allows us to guarantee the traceability of the patient's identity

ü Send or take the sample to the laboratory in the shortest time possible.

ü Fill the vacuum tubes to the marked level; It is essential that they are full right up to the sign. ü Do not draw blood from the same extremity used for the intravenous administration of medications, fluids or transfusions. If no other site is available, ensure that the venipuncture is located below the catheter.

If the patient's condition allows it and under medical authorization, the nursing professional closes the flow for 10 minutes, takes a discarded yellow cap tube and then takes the tube necessary for the requested examination, on the sticker make the note with in order to make a correct correlation at the time of validating

Machine Translated by Google

the result.

ü Do not draw blood from hardened veins, arteriovenous shunts, places of hematoma, inflammation or swelling, from an arm with a vascular graft, paretic arms or arms with lymphatic drainage disorders.

Venous puncture can cause infection, circulatory disorders or delayed healing, due to inappropriate application of the technique.

At the Shaio Clinical Foundation, patients identified with a blue sticker are restricted from taking the sample because they have edematous, paralyzed areas, mastectomy or arterovenous fistula, and areas with infection or skin lesions. ü If the sample is taken from a line already installed in the patient (Catheter), it is essential to purge at least 20 cc in order to avoid chemical contamination.

ü Use the guards to discard the needles and use the bins according to the waste segregation in sampling.

ü Remember that a diagnosis or the evolution of a patient depend on a good procedure, the commitment of the person who takes the sample, processes it, validates it and reports it.

7. 2. PROCEDURE FOR TAKING SAMPLES IN CHILDREN

- In outpatients, verify that the parents or guardians have signed the consent for sample collection.
- Correctly follow the instructions proposed above.
- Perform the procedure requesting the help of another professional and/or a family member of the patient. minor.
- Hold the patient firmly without injuring him.

7.3. BIOSECURITY IN SAMPLING

Improper handling of samples can become a source of biological risk for people in contact or for the environment. All samples of biological specimens must be considered potentially infectious, so the Biosafety protocol established by the institution must be rigorously followed.

Use personal protection elements according to the biosafety standards of the clinical laboratory and the institution's infection committee, necessary to avoid exposure with biological risk according to the source of the sample: eye protection (goggles or face mask), gloves, coat.

Comply with the recommendations for handling sharps: Do not resheath needles, properly dispose and use the container for sharps, do not transport syringes with needles.

Machine Translated by Google

In the event of an accident with biological risk, notify immediately according to the recommendations of the institutional biological risk work accident protocol.

No food or drink should be ingested during the sampling procedures or in the places provided for this purpose.

7.4. PROCEDURE IN CASE OF A WORK ACCIDENT DUE TO PUNCTURE

- Remove gloves
- Constantly press the wound area allowing it to produce bleeding
- For ten minutes, wash the wound very well with soap and water.
- Follow the guidelines for reporting to the ARL according to institutional protocols; Notify the director of the area and the person in charge of occupational health of the institution, go to the emergency room, take the required samples and initiate post-exposure prophylaxis if indicated.

Related guidance documents are as follows:
Program PG-10.1-07 Work accident monitoring.
Instruction IN-10.1-02 Attention to work accidents.
Instructions IN-10.1-03 Instructions for the management, investigation and monitoring of work accidents.

8.0. PATIENT PREPARATION

- Proper patient preparation, sample collection and handling ensure optimal quality results while providing valid results.
- Patient-related factors that can affect results are divided in:
  ü Intrinsic or unchangeable: Age, sex, race, pregnancy, biological cycles.
  ü Extrinsic or Controllable: Diet, exercise, variable habits (smoking, alcohol, caffeine), sample collection (material, tourniquet), transportation and conservation.
- Mental or physical stress can affect the levels of many constituents of body fluids, therefore the patient should be and feel comfortable and comfortable.
- Anxiety and tension are stimulants of plasma concentration of somatotropin, prolactin, cortisol, catecholamines, aldosterone and renin.
- Periods of long stress affect metabolic tests such as glucose, proteins plasma and some coagulation factors.
- Exercise and vigorous muscle work affect CPK, LDH, K, Glucose, lactate, creatinine and some coagulation factors.
- Exercise stimulates the secretion and production of hormones such as blood hormone.

Machine Translated by Google

growth, prolactin, cortisol and renin.
- The blood sample should be drawn in the morning on an empty stomach, 8 to 12 hours after the last meal. Consumption of water in small quantities is allowed during the fasting period. Insulin, calcitonin, phosphates, triglycerides, alkaline phosphatase are altered after food intake.

- Give precise and clear instructions to the patient about the optimal preparation conditions for taking some tests. (see patient preparation annex)
- Verify, if applicable, that the patient has rigorously followed the instructions before taking the sample.
- Ethanol ingestion can alter samples of liver enzymes, glucose, triglycerides, urates, lactate and prothrombin.
- Record or take into account the medications that the patient is taking to carry out an adequate analysis and validation of the results. Medicine should be avoided in the morning unless it is vital for the patient.

8.1. SAMPLE COLLECTION TECHNIQUES

8.1.1. VENOUS SAMPLES

Venous blood collection should be performed in a clean, quiet, and private environment.
The patient should be reassured that the stress caused by phlebotomy (puncture) can affect laboratory results such as changes in catecholamine and blood gas concentrations.

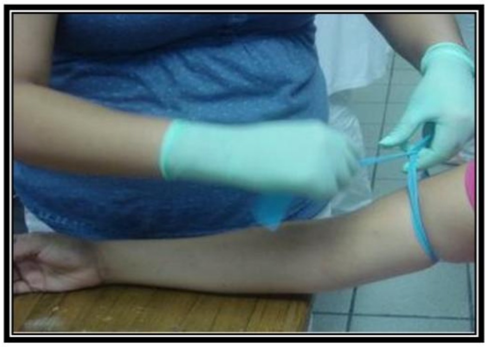

Place the tourniquet

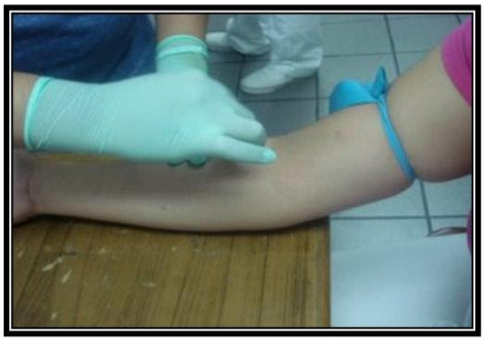

Asepsis of the area

Machine Translated by Google

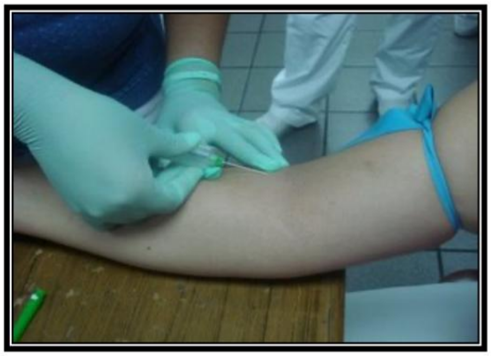

Perform the puncture

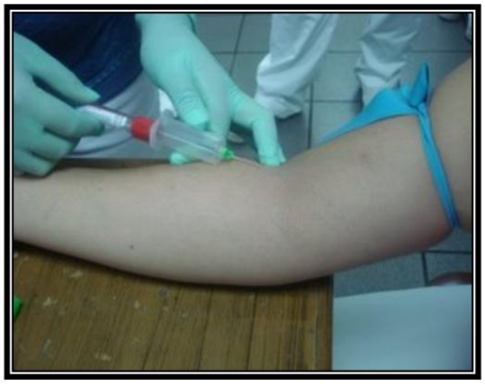

Take the sample

To select the venipuncture site, the patient's arm should be stretched downward. If available, the most prominent veins in the cubital seal should be the first choice.
Only if the main veins are not available, the dorsal hand veins can be used as an alternative. They can also be obtained from a central venous access established in the patient.

It is recommended to take the sample from a vein independent of the peripheral vein that is being used for intravenous therapy, using a closed vacuum tube technique, which improves its quality (appropriate quantity, decrease in hemolysis) and reduces the risk of contamination.

8.1.2. PROPER POSITION OF THE PATIENT:

Make sure the patient is in a safe and comfortable position.
Ideally, the patient should not change their position within 15 minutes prior to blood sampling. If the patient is lying down, the blood sample should be taken in a lying position (hospitalized patients). Outpatients should ideally rest in a sitting position for 15 minutes before blood sampling. Positional modifications affect the results of: Albumin, proteins, various enzymes, calcium, bilirubin, cholesterol, triglycerides, angiotensin, aldosterone and renin.

Lying down: There is an accommodation or distribution of hemodynamics and other body fluids

Sitting: The outflow of intravascular fluid into the interstitial space begins and, therefore, hemoconcentration occurs.

Machine Translated by Google

8.1.3. TOURNIQUET:

Blood collection should preferably be performed without a tourniquet (especially in patients with prominent veins) and this should be used only when necessary (patients with small or barely visible veins).
The tourniquet should be applied approximately one hand's width (7.5 cm) above the intended puncture site and should be tight enough to stop venous blood flow, but not arterial blood flow, too high does not exert pressure and too low increases the possibility of hematoma.

Its use should not be prolonged for more than 1 minute, it can produce localized venous ecstasy, the sample becomes hemoconcentrated, inducing erroneously high values.
The patient is asked to make a fist, which distends the veins. Excessive fist exercise should be avoided, since it can cause an increase in the concentration of potassium and LDH.

Samples to determine Lactate and Potassium should be taken without a tourniquet.

8.1.4. SELECTION OF THE PUNCTURE SITE

Ø Never perform a blood puncture on a patient who is standing (The standing position is unstable and if the patient faints, it will be more difficult to prevent injury). Ø Do not choose an extremity where
any type of infusion is placed. Ø Inspect the vein to be punctured.

Ø Place the tourniquet with the necessary tension level. Ø If
the vein is not very visible or palpable, gently massage the forearm with movements from the wrist to the elbow.

Ø Always observe the two upper extremities (arms) to choose the best place puncture.

Ø Remember that: Hematomas are prevented with proper technique by preventing the needle from passing through the vein, releasing the tourniquet before removing the needle, and applying sufficient pressure on the puncture site.

Ø At the end of the procedure, tell the patient to apply pressure to the punctured site for at least five (5) minutes. Finally place an adhesive band over the puncture site.

Ø If the bleeding does not stop, apply constant pressure on the puncture for 5 more minutes. If the problem is still not resolved, notify the nursing staff in charge.

Ø If the patient becomes dizzy or tends to faint, have him lie down and breathe deeply, monitor vital signs and immediately inform the treating physician and/or nursing staff.

Ø Leave the patient comfortable.

Machine Translated by Google

Ø Carry out the correct disposal of the waste generated from the procedure.

Be sure to label the sample tubes with complete identification in the presence of the patient.

Complications derived from venous extraction: Hematoma, Bleeding, Extravasation trauma, etc. These must be recorded in the Venipuncture Incident Report Book RE-4.5.4-78

8.1.5 SPECIAL RECOMMENDATIONS FOR SAMPLING

If blood does not flow into the tube or the flow decreases before collecting the appropriate sample, perform the following steps:

- Confirm the position of the needle in the vein
- The opening of the needle may be against the inner wall of the vein, slowly rotate the needle holder and blood should begin to flow.
- If the needle has passed through the vein, pull the guide slightly
- Confirm the correct position of the tube in the guide
- There may be loss of vacuum due to premature perforation or tube opening. You must change the tube.

Make sure to take the sample in the correct tube and in the following order if done by closed system:

Orden de toma para recolección de sangre venosa

Tapón	Contenido de tubo	Área de uso	Inversiones
	Hemocultivo	Microbiología	5 veces
	Citrato de sodio	Coagulación (Tiempos de coagulación fibrinógeno, y agregación plaquetaria)	3 a 4 veces
	Gel separador	Química clínica	5 veces
	Sin anticoagulante, con activador de coagulación, con silicón	Química clínica, banco de sangre serología	8 a 10 veces
	Gel separador y trombina	Obtención de suero rápido	5 a 6 veces
	Gel separador y heparina de litio	Química clínica en plasma	5 veces
	Heparina de sodio/litio	Química clínica (urgencias) hematología (fragalidad osmótica)	8 a 10 veces
	$EDTAK_2$	Hematología, banco de sangre	8 a 10 veces
	Gel separador y $EDTAK_2$	Determinaciones de carga viral	8 a 10 veces
	Oxalato de Potasio/NaF	Química clínica, pruebas de lactato y glucosa	8 veces

Fuente: Clinical and Laboratory Standards Institute (CLSI) – H3-A6 - Procedures for the Collection of Diagnostic Blood Specimens by Venipuncture; Approved Standard- Sixth Edition, recomendaciones para tubos de plástico.

1- In **blue tube:** PT, PTT, fibrinogen, antithrombin III, lupus anticoagulant, D-Dimer, Protein C and S for coagulation and platelet aggregation (5 tubes, must be sent immediately to the reference laboratory). Invest to guarantee anticoagulation. Mix by inversion 3 to 4 times. This tube is very sensitive to the blood/anticoagulant ratio, so it should be taken up to the indicated mark.

2- In **yellow and/or red tube:** blood chemistry, serology, infectious, immunology, serology, fibrinogen degradation products. The yellow apa tube should be mixed by inversion 5 times and the red cap 8 to 10 times.

3- In **green tube:** Immunogenetic tests, Troponin I. Mix by inversion from 8 to 10 times.

4- In **purple tube:** Blood count, erythrocyte sedimentation rate, platelets, hemoclassification, LE cells, direct and indirect Coombs and hemoparasites (FSP). Invert the tube to homogenize the sample with the anticoagulant 8 to 10 times.

5- In **gray tube:** Lactic acid and ethanol tests. Mix by inversion 8 times.

By taking samples through a closed system, risks are reduced, errors are reduced and processes are improved. If it is necessary to take drip samples, for example when referring the patient to the emergency room and resuscitation, it is suggested that the personnel taking the samples be accompanied by another professional or assistant who guarantees the adequate level of filling, the correct mixing of samples. by investment and correct identification.

BD Microtainer tubes are designed for collection, transportation and processing of samples obtained by capillary or venous puncture from pediatric patients,

Machine Translated by Google

geriatric, emergency, fragile veins, or any condition that requires small volumes of samples because they are difficult to access.

- ü Each tube has a filling volume mark to ensure the correct proportion of sample volume and anticoagulant.
- ü Maintains the color code of the cap in accordance with international regulations.
- ü It must be taken into account that the order, final volume and mixing during the taking process is very important to avoid the formation of clots in the lilac lid tubes.

Tapón	Contenido de tubo	Área de uso	Inversiones
Gases en sangre	Jeringas o capilares		
Muestras para microscopio			
	EDTA K_3	Hematología, banco de sangre	20 veces
	Heparina de sodio/litio	Química clínica (urgencias) hematología (fragilidad osmótica)	10 veces
	Heparina de litio y gel	Química clínica en plasma	10 veces
	NaFl / EDTA Na_2	Química clínica, pruebas de lactato y glucosa	10 veces
	Gel separador	Química clínica	5 veces
	Sin anticoagulante con silicón	Química clínica, banco de sangre, serología	10 veces

[illegible]

8.1.6 SERUM LEVELS OF MEDICATIONS

The blood collection time for therapeutic drug monitoring (MDT) will depend on the medication and the indication for the test (drug dose optimization, monitoring of drug adherence, adverse effects, drug intoxication, etc.). They should follow specific recommendations from the MDT physician for the exact timing of blood sampling.

Digoxin: The sample should be taken 8-12 hours after the last dose of the medication.

Theophylline: The sample should be taken 8-12 hours after the last dose of the medication.

Phenobarbital: The sample should be taken 8-12 hours after the last dose of the medication.

Phenytoin: The sample should be taken 8-12 hours after the last dose, it is recommended

Machine Translated by Google

Collect samples at least 2 hours after giving an intravenous dose of fosphenytoin and at least 4 hours after an intramuscular dose.l.
Ciclosporine: Taken two hours after the last dose, or as directed by the doctor.
Vancomycin: Monitoring of trough drug concentrations should begin once steady state is reached and samples should be drawn within 30 minutes before the next dose.

When measuring peak concentrations, samples should be drawn 0.5-2 hours after an infusion.

Protect from light.
Tacrolimus: It should be taken 15 minutes before the usual time of taking the medication.

NOTE: In all cases, the dose, date and time of the last dose of the medication must be listed. Date and time of sample collection, route of administration and patient diagnosis.

8.1.7. GLUCOSE TOLERANCE CURVE / POST LOAD GLUCOSE

Ø Obtain blood from the patient on an empty stomach
Ø Perform glucometry by obtaining the finger prick sample. If the result is greater than 128 mg/dl, do not give the load.
Ø The patient must take 75 g of dextrose diluted in 300 ml of water. For which the patient must sign the consent for the administration of glucose loads.
Obtain blood samples to quantify glucose at half an hour, one hour, two hours, three hours after ingesting the load for the curve, or two hours for post-load.

Ø The patient must remain at rest, not smoke, not eat, not drink during the time of the study.

8.1.8. POSTPRANDIAL GLUCOSE

Ø Obtain blood from the patient on an empty stomach
Ø Instruct the patient to have a normal breakfast and return to the laboratory exactly two hours after finishing breakfast to take the second blood sample. During those two hours you should not exercise or consume any different food.

8.1.9. O SULLIVAN TEST

Ø Test for post-load glucose control of 50 g during pregnancy.

Machine Translated by Google

Ø Ask the patient if she is fasting
Ø Perform glucometry. If the value is below 115 mg/dl, give it a load of 50g diluted in 200 ml of water.
Ø Take the second blood sample one hour after ingestion.

NOTE: According to the Clinical Practice Guide for the diagnosis, treatment and monitoring of Gestational diabetes of the Ministry of Health and social protection (2015 guide No GPC-2015-49), it is recommended to do the one-step test of the IADPSG (International Association of the Diabetes and Pregnancy Study Groups) to all pregnant patients from week 24, for the diagnosis of Gestational Diabetes with 75 g of glucose and two postprandial feedings at one hour and two hours. However, if the doctor orders it, the O Sullivan test is performed.

8.1.10 GLUCOMETRY

8.1.10.1. SUMMARY AND PRINCIPLE

The Accu-Chek® Inform II system is a device for *in vitro* diagnostic use designed for the quantitative determination of blood glucose levels in venous, capillary, arterial and neonatal blood samples.

The quantitative estimation of Glucose is based on an enzymatic reaction where the glucose in the blood sample is converted into Gluconolactone in the test strip. This reaction generates an electric current that the meter interprets (Amperometry) and converts into a Glycemic result. .

The procedure by which the measurement of glucose level in capillary blood is carried out can be:

Preprandial: before food and **Postprandial:** after food.

It can also be performed at times when the patient's symptoms make us think of an alteration in blood glucose levels.

8.1,10.2. STORAGE AND HANDLING

The test strips come in a container that must remain closed.

Machine Translated by Google

Store them at room temperature.

8.1.10.3. Glucometer: device that measures the amount of glucose in a blood sample generally taken by prick of the pad of a finger (capillary blood) that is applied to a test strip that is introduced into this device for reading.

8.1.10.4. Equipment:

- Accu-Chek meter
- Accu-Chek Performa or Accu-Chek Inform II test strips with the chip correspondent.
  - Store strips between 2 and 30°C
  - Keep the strips in their original container, covered.
  - The strips are stable for a maximum of 3 minutes after removing them from the packaging, so they should be used immediately.
  - When removing the strip from the container, hold it in the center, do not touch the ends.
- Accu-Chek Performa Control Solutions
  - Keep the Solutions at room temperature (between 2 and 30 °C)
  - Each bottle of Control Solution is stable for 90 days after opening. (Don't forget to close the bottle after use).
- Lancet device approved for professional use, adjusts according to depth required.
- Alcohol (if you use alcohol, do not forget to dry the skin very well). If possible, ask the patient to wash their hands.
- Waste containers

8.1.10.5. PROCEDURE

Once glucometry is requested by system, perform glucometry as follows:

Ø Press the on/off button to turn on the meter.
Ø When the Home screen appears, press the arrow to access the User ID screen or wait 5 seconds for the meter to access it directly
Ø Enter or scan the user ID and press to open the main menu screen.

Ø Press patient test to open the patient ID screen.
Ø Enter or scan the patient ID and press will ask you to confirm the batch of test strips. then The meter will
Ø Check the batch of test strips.
- If correct, press . The meter will ask you to insert the test strip.

Machine Translated by Google

- If not correct, press **X** to select a different lot number

Once the batch of test strips is confirmed, the system will prompt you to insert the strip.

- Ø Remove the test strip from the test strip vial and close the vial with the cap.
- Ø Hold the test strip in such a way that the inscription “ACCU-CHEK” faces above.
- Ø Insert the test strip into the test strip slot as far as it will go in the direction indicated by the arrows on the test strip. The meter will emit an acoustic signal.

When the meter detects the test strip, it requests that a blood sample be applied.

- Wait for the flashing drop symbol to appear on the screen to apply blood. The meter will beep again.
- It is recommended that the patient wash and dry their hands, if this is not possible, clean the finger with alcohol, let it dry very well, make the puncture and clean the first drop.
- Apply the drop of blood to the **front end** (yellow dosing area) of the test strip. **Do not** apply blood to the top of the strip. The test strip absorbs blood by capillary action. The blood at the top of the strip is not available for testing.

When the meter detects a sufficient amount of blood sample, it emits an acoustic signal and starts measurement. The hourglass icon indicates the completion of a test.

When the test is finished and the result is ready, the meter emits an acoustic signal again.

8.2. COLLECTION OF SAMPLES FOR SPECIAL TESTS

8.2.1. CORTISOL

- Ø The patient must be fasting
- Ø Two samples will be taken at 8:00 am and 4:00 pm

8.2.2. PROLACTIN POOL

- Ø The patient must be fasting
- Ø The sample must be taken at least two hours after the patient wakes up.
- Ø Three samples will be taken every twenty minutes.

Machine Translated by Google

8.3. PARTIAL URINE AND URINE CULTURE

In older pediatric patients who control sphincters, collecting samples by spontaneous urination is the ideal technique due to its simplicity and non-invasiveness. The situation is opposite in the cases of pediatric patients who do not control urination, an issue in which there are no generalizable validity estimators; However, despite the risk of contamination associated with the use of a collection bag with an adhesive strip, this is one of the most widely used methods worldwide to obtain urine samples in this segment of the population. Sample collection through transurethral catheter and suprapubic puncture have been considered the ideal collection methods in the pediatric population, but their invasiveness has restricted their use.

8.3.1 Care and Recommendations:

- All urine samples, except samples taken by catheterization or suprapubic puncture, must be obtained by the patient; For this reason, steps should be taken to avoid contamination of the sample with vaginal discharge, sperm, pubic hair, powders, oils, lotions, and other foreign materials. Samples should never be collected from diapers (CLSI, GP16A3, 2009)

- It should be the first urination of the day, random urine.
- Perform genital hygiene.

It is not indicated for the detection of anaerobes. It is an important sample for detection of infections caused by cytomegalovirus (CMV), enterovirus, and adenovirus. To search for Mycobacteria, the collection is carried out in the same way, culture is performed but smear microscopy is not performed due to the low specificity.

Instruct the patient how to collect the sample:

8.3.1.1. Man

- In the case of patients who have not been circumcised, the foreskin to expose the glans and urinary meatus
- Wash the glans.
- Eliminate the initial part of the jet.
- From the middle part of the jet, take the sample in a sterile container.
- Cover the container, identify it correctly , set the pickup time and send immediately to the laboratory.

Machine Translated by Google

8.3.1.2 Woman:

- Separate the labia majora and minora gently.
- Wash from front to back with a gauze soaked in soap and water.
- Clean excess soap with water.
- Keeping the lips separated eliminate the initial part of urination.
- From the middle part of the jet, take the sample in a sterile container.

If, due to clinical conditions, the patient must be assisted in taking the sample, after washing hands and putting on gloves, comply with the previous instructions and continue. with:

- Cover the container, identify it correctly , set the pickup time and send immediately to the laboratory.
- If the patient has a urinary catheter, **the catheter should never be untied.** It should be handled with sterile technique. The passage is blocked for ten to fifteen minutes, to take the sample, the distal end of the probe is cleaned with antiseptic solution and with a 10 cc syringe the probe is punctured, the sample is extracted and sent to the Clinical Laboratory, in the same syringe, properly identified.
- If a sample must be collected from a patient with vaginal bleeding, a probe must be used.

8.3.1.3 Sample collection for partial urine in children

In children, a sterile plastic urine collection bag is used. The bag must be changed after fifteen minutes if urination has not occurred.

- The bag will be placed after having washed the genitals, adhering it to the skin by means of an adhesive ring.
- In children, firmly adhere the bag to the base of the penis by pressing the bands on the patient's skin. • In girls,

stretch the skin of the perineal region to reduce folds. Press the adhesive bands firmly to the skin around the genitals. Start in the space between the anus and vagina to avoid contamination of the sample from the rectal area.

- Confirm that there are no folds or open spaces in the adhesive strip. • Once the

patient has urinated, remove the collection bag, avoiding contamination. Carefully transfer the sample to a sterile wide-mouth bottle.

- Close the bottle, verifying that no drips occur and label the sample with the name and identification number of the patient and the time of collection.

Machine Translated by Google

- The bag must be changed after fifteen minutes if it is not urination has occurred.

8.3.5. Suprapubic puncture

Occasionally, suprapubic aspiration of the bladder may be necessary and is performed by the physician. It consists of direct puncture of the bladder through the abdominal wall with a sterile needle and syringe. (You must ensure that the patient has a full bladder before starting the procedure.

8.3.6. UROCULTURE COLLECTION

8.3.6.1. Technique and Collection:

Ø Adults: Take into account the same indications for collecting partial urine. Instruct the patient to start urination, discard the first part of the urine, introduce the collection bottle, collect the middle part of the urine without stopping the urinary flow (5-10 cc) and finish eliminating in the toilet or duck. Cover the bottle without contaminating the sample.

Ø Pediatric: To perform Urine Culture of urine samples from children, it must be done through a probe. A urine collection bag cannot be used, due to the high risk of contamination it represents.

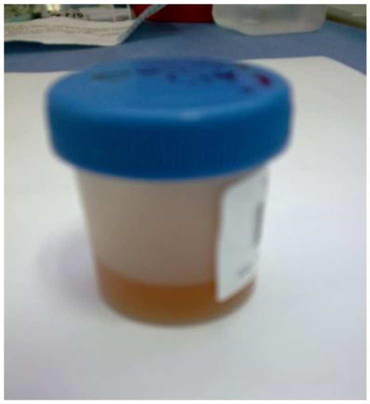

8.3.6.2. Transport:

All samples taken to the laboratory must comply with certain conditions:

- The collection jars should always have a wide mouth and a screw cap to ensure

Machine Translated by Google

Ensure the hermetic closure of the jars and avoid drips.
- Samples must be labeled with the name and identification number of the _ patient, as well as the date and time of collection .
- Ideally, samples should be transported immediately to the laboratory clinical.
- Record in the notebook found in the service and have the receipt received signed in The laboratory.

8.3.7. 3 HOUR URINE COLLECTION

- Urinate in the morning when you get up and write down exactly the time taking into account that this sample will not be used for the examination.
- Collect subsequent morning and afternoon urine samples in the container provided by the laboratory or in a bottle of clean mineral water for a period of 3 hours.
- Keep the bottle in the refrigerator during collection.
- Take the sample once finished to the laboratory.
- Recommend the patient **NOT** to drink alcoholic beverages.

8.3.8. SAMPLING FOR RESEARCH OF KOCH BACILLUS (BK culture)

- Urine from the first urination is collected. It is recommended to collect it in glass water bottles with a capacity of 250 ml.
- Take the collected sample and the medical order to the laboratory.
- Recommend the patient avoid the consumption of alcoholic beverages.

8.4. SAMPLE COLLECTION FOR COPROLOGICAL AND COPROSCOPIC

- Collect the sample in the special sample container
- Do not receive samples that come in inappropriate containers
- The sample should not be contaminated with urine or water as it may inhibit bacteriological growth or may contain free-living microorganisms.
- Some drugs and mineral oil can affect the test.

8.4.1. HIDDEN BLOOD

- There should be no evidence of dental bleeding.
- Do not collect the sample if you are on your period.

Machine Translated by Google

8.5. UPPER AND LOWER RESPIRATORY TRACT

Respiratory diseases can be divided into two types: those of the upper respiratory tract and those of the lower respiratory tract. The former usually involve the ears, the mucous membranes of the nasal cavity and the pharynx up to above the epiglottis. The main etiological agents of upper respiratory tract diseases are viruses, such as respiratory syncytial virus, influenza virus and adenovirus, among others; The main bacterial agents responsible for upper respiratory tract diseases will depend on the location of the infection, such as *Streptococcus pneumoniae and Haemophilus influenzae* in otitis media and acute sinusitis and *Streptococcus pyogenes* in bacterial pharyngitis.

Typically, the most frequently used specimens in upper respiratory tract infections include pharyngeal swabs, nasopharyngeal swabs or washes, and oral cavity swabs.

The specimens that are most frequently used to identify the microorganisms that cause lower respiratory tract diseases are sputum, induced sputum, tracheal aspirate, bronchial lavage and bronchoalveolar lavage.

8.5.1. Taking the sample.

Respiratory tract samples should be collected as soon as possible, before the start of antibiotic therapy. The possibility of recovering viruses and bacteria decreases significantly after 72 hours after the onset of symptoms of the disease and after starting antibiotic therapy.

8.5.2 Nasal swab

Mainly used for the detection of fungi and/or methicillin-resistant *Staphyloccocus aureus* by culture.

8.5.2.1 Care and recommendations

- •Avoid nasal drops and baths before taking the sample
- •It is not recommended to send cultures for anaerobes
- •Nasal smear and culture are not indicated for the diagnosis of sinusitis, otitis media or lower respiratory tract infections.
- •It is only recommended to take cultures from the anterior nasal passages to detect carriers of *Staphylococcus aureus* or in nasal lesions. The same technique is recommended for searching for mushrooms.

Machine Translated by Google

8.5.2.2 Collection Technique

- •Place the patient under a good light source
- •Lift the patient's head and with the other hand insert the moistened swab 1 to 2 cm inside the nostrils, rotate it against the nasal mucosa for a period of 10 to 15 seconds and then remove it and identify which nostril it comes from. I take the sample.
- •Label the sample with the patient's name and identification number and the time of collection.

8.5.2.3 Transport

ØThe transport of the samples to the laboratory should be carried out as soon as possible, ideally within the first two hours after taking the samples, keeping them at room temperature (L Raka, 2012). If this is not possible, the samples must be refrigerated immediately after being taken, at a temperature between 4ºC and 8ºC for a maximum of 48 hours.

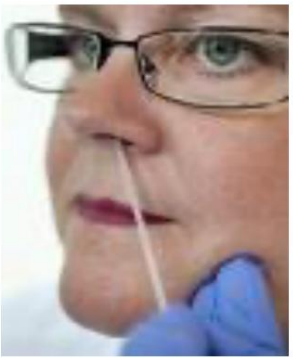

8.5.3. Nasopharyngeal swab

The nasopharyngeal swab is used for bacterial cultures, immunofluorescence studies and for real-time PCR studies (FILMARRAY), for detection of microorganisms that cause respiratory diseases including SARS-COV-2 (COVID-19).

• Moisten the swabs with saline solution. • With
your free hand, bring the patient's head back and with the other hand, insert the moistened swab through the nostrils, parallel to the palate (not up), until resistance is encountered or the equivalent distance from the nose. nostril to the ear. At this point the tip is located in the nasopharynx.

Machine Translated by Google

• Gently rotate the swab for 5 seconds and then slowly remove it, allowing The secretions on the swab are absorbed. •
Place the swab in the tube.
• Repeat the procedure in the contralateral nostril. • Once
the process is finished, remove the gloves and perform hand hygiene. • Label the
sample with the patient's name and identification number and the time of collection. harvest.

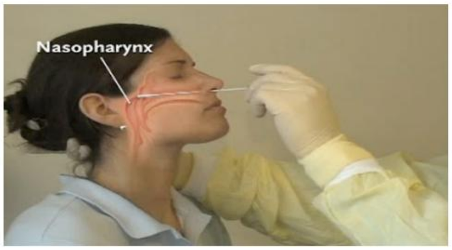

8.5.4. Nasopharyngeal aspirate

Nasopharyngeal aspirate is the method of choice to diagnose influenza virus and other respiratory viruses. Likewise, it is the ideal method for searching for *Bordetella pertussis* in the pediatric population.

8.5.4.1. Collection technique

• With your free hand, bring the patient's head back and introduce 1 to 2 mL of sterile saline solution (pH 7.0) into one of the nostrils using the syringe attached to the probe.

• Introduce the probe with 2 to 3 mL of saline solution through one nostril, parallel to the palate (not above) • Aspirate the
sample until obtaining the largest possible volume inside the syringe; There it should be evident that the saline solution is cloudy, which guarantees that the secretions have been collected correctly. • Carefully remove the
probe from the nostril. • Close the collection tube tightly
and clean its exterior thoroughly with
help of a gauze moistened with 70% isopropyl alcohol.

8.5.4.2 Transport

The transportation of the samples to the laboratory should be carried out as soon as possible, ideally within the first two hours after taking the samples, keeping them at room temperature (L Raka, 2012). If this is not possible, the samples

Machine Translated by Google

They must be refrigerated immediately after being taken, at a temperature between 4ºC and 8ºC for a maximum of 48 hours.

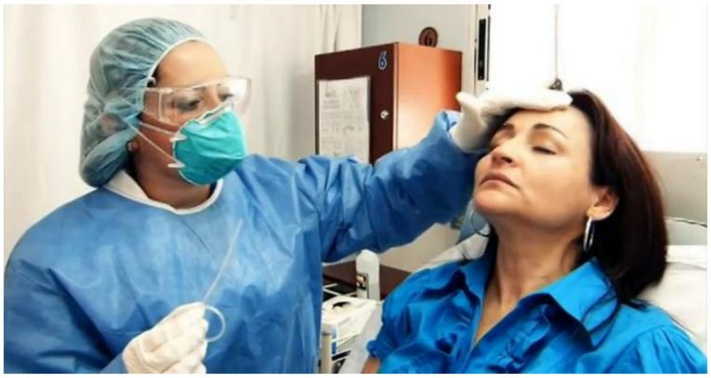

8.5.5. Pharyngeal swab

The throat swab is used for bacterial cultures essentially to look for *Streptococcus pyogenes.* However, it is also used in the search for *Corynebacterium diphteriae and Neisseria.* Detection of yeasts and fungi is generally restricted to microscopic evaluation of Gram stain. (P Murray, 2010)

Throat swab is contraindicated in patients diagnosed with epiglottitis.

8.5.5.1 Collection Technique

• Do not gargle or cleanse with any oropharyngeal solution.
• With your free hand, take the patient's head back and ask him to open his mouth; With the help of a tongue depressor, press the tongue down to facilitate visualization of the pharynx and avoid contamination of the swab. • Avoiding touching the tongue, teeth or gums, insert the swab into the posterior pharynx and tonsillar region. Rub the swab against the tonsillar walls and posterior oropharynx. • Remove the swab from your mouth and immediately place it in the tube.

8.5.5.2. STREPTO A TEST

8.5.6.2.1. Obtaining and preparing the sample:

Machine Translated by Google

•Collect throat swab samples with the sterile Dacron swab provided in the kit. Transport the swab containing Stuart's or Amies modified medium. Rub the posterior pharynx, tonsils and other inflamed areas. Avoid touching your tongue, inner cheekbones, and teeth with the swab.

•The test should be performed immediately after the samples have been collected. Swab samples can be stored at room temperature for up to 4 hours before testing. •If you want to perform a culture, lightly roll the tip of the
Dacron swab for pharyngeal sampling on Blood Agar, before using the swab in the rapid test.

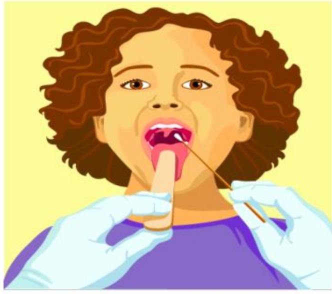

8.5.6 Sputum

Taking into account the ease of obtaining sputum samples, this technique is widely used in uncomplicated patients to adequately guide the clinical diagnosis.

8.5.6.1 Patient conditions

- Collect the sample in the morning.
- Instruct the patient that before taking the sample, they must first perform a
  Washing the oral cavity with water to reduce excess bacterial flora. (P Murray, 2010)
  - Preferably

fasting.

8.5.6.2 Collection technique

- Explain to the patient that he or she must cough deeply to mobilize secretions from the lower respiratory tract.
- Instruct the patient to expectorate and collect the sputum generated in the sterile bottle.
- If you are intubated, the respiratory therapist places the secretion trap between the

Machine Translated by Google

aspiration catheter and suction, obtains the sample, correctly identifies it and immediately sends it to the clinical laboratory.

- Close the bottle immediately, verifying that it is not contaminated with secretions on the external surface.

8.5.6.3 Recommendations

- For the study of mycobacteria, three (3) serial samples must be obtained.
- Samples can be obtained at any time during the clinical course of the procedure. disease, but it is recommended that they be taken before the start of antibacterial therapy.
- The presence of abundant epithelial cells is a strong indicator of contamination with oral bacterial flora. A contaminated specimen cannot be accepted for routine bacterial cultures, but can be taken into account for mycobacterial cultures.
- An adequate sample for culture should be representative of the lower airway (contain less than 10 epithelial cells and more than 25 polymorphonuclear cells per low-power field).

8.5.7. induced sputum

Taking samples of induced sputum with nebulizations with 0.9% saline solution is indicated in patients with difficulty obtaining the sample by expectoration.

8.5.7.1 Collection Technique

• Nebulize the patient with 3 mL of 0.9% normal saline solution. • Explain to the patient that he or she must cough deeply to mobilize secretions from the lower respiratory tract. • Instruct the patient to expectorate and collect the sputum generated in the sterile bottle.

8.5.7.2 Recommendations

• For the study of mycobacteria, three (3) serial samples must be obtained. • Samples can be obtained at any time during the clinical course of the disease, but it is recommended that they be taken before the start of antibacterial therapy.

- Unlike sputum obtained by expectoration, induced sputum may be contaminated by abundant epithelial cells and bacteria from the oral cavity. He

Machine Translated by Google

specimen should be processed for bacteria despite contamination of the oral cavity.

- Induced sputum should be performed in a single room with adequate aeration to avoid the risk generated by the aerosolization of mycobacteria.

8.5.8. Tracheal aspirate

Tracheal aspirate is used to determine the etiological agent in patients with pneumonia. Contamination of samples with microorganisms from the oral cavity can be very common. It should always be remembered that tracheal aspirate samples must be cultured despite the presence of epithelial cells in the smear (P Murray, 2010). The smear of the tracheal aspirate, in the absence of inflammatory cells and the negativity of the culture for common germs, has a high negative predictive value (E Baron, 2013; 57).

8.5.8.1. Collection Technique

- Introduce the Nelaton tube into the tracheal cavity through the endotracheal tube or of the tracheostomy orifice.
- Connect the suction device to the distal end of the probe and carefully aspirate the contents of the tracheal cavity.
- Slowly withdraw the probe, always keeping the distal hole of the probe occluded or connecting the suction device to the probe. Using sterile gauze, clean the external surface of the probe.
- Place the proximal end of the probe in the sterile bottle. With the scalpel blade, cut the distal end of the probe, ensuring emptying of the contents of the probe into the container.

8.5.9. Bronchoalveolar lavage, bronchial brushing and bronchial lavage.

Bronchoalveolar lavage, bronchial brushing and bronchial lavage are indicated in different pathologies according to clinical suspicion or in cases where the sputum has not been conclusive and there is still a suspicion of bacterial, fungal or viral infection (including *Pneumocystis jirovecii.)* of the lower respiratory tract or lung parenchyma. (L Raka, 2012).

Bronchoalveolar lavage samples are ideal for performing cultures, cytopathological studies and molecular biology tests. Using the fiberoptic bronchoscope, bronchoalveolar lavage of the desired lung segment can be obtained by instilling 300 to 350 mL of normal saline solution (0.9%) in 50 mL aliquots, which will subsequently be aspirated and collected in a sterile wide-mouth bottle. (L Raka, 2012)
The performance of Fibrobronchoscopy requires being performed by a specialist doctor with training in endoscopic procedures.

Machine Translated by Google

8.5.10. Recommendations for packaging and transporting samples to the laboratory

Swabs and probes should be collected in sterile tubes with antibiotic-free transport media. Samples of secretions, washings and expectorations should be collected in sterile wide-mouth bottles.
The transport of the samples to the laboratory should be carried out as soon as possible, ideally within the first two hours after taking the samples, keeping them at room temperature (L Raka, 2012). If this is not possible, the samples must be refrigerated immediately after being taken, at a temperature between 4ºC and 8ºC for a maximum of 48 hours. If the sample processing time will be longer than 48 hours, the sample can be frozen for 2 months at -20ºC or for 6 months at -70ºC.

8.5.11. MIDDLE EAR SECRETIONS AND EYE SECRETIONS

Acute otitis media is one of the most common infections in pediatrics, which is why treatment is essentially empirical, a clinical practice that may be responsible for the high rate of resistance to ÿ-lactams and macrolides of the etiological agents of otitis. mainly *Streptococcus pneumoniae* (Gené, 2004).
Conjunctival infections, although they generally do not produce a long-term risk, are responsible for 1% of consultations; Eight out of ten children have a conjunctival episode once a year and doctors usually order antibiotics to treat patients.

8.5.11.1 EYE SECRETION

8.5.11.1.1 Care and Recommendations

- •Do not use eye drops 18-24 hours before the sample
- •Absence of any cosmetic
- •Do not take antibiotics 24-48 hours before
- •Do not use anesthetics that have antimicrobial activity

8.5.11.1.2 Collection Technique

• With 0.9% saline solution, clean the external surface of the involved eye. • With your fingers, separate the lower eyelid and rotate the swab moistened with solution saline by rubbing the inner edge of the conjunctiva from the conjunctival fornix at the edge

Machine Translated by Google

nasal to the temporal border.

- Place the swab in a sterile tube with transport medium for the laboratory. • Repeat the procedure with the second swab and make a spread on the sheet slide
- Again repeat the procedure for the contralateral eye. • Cover the tubes with the transport medium, verifying that there are no leaks for the transport.
- **Corneal scraping:** This sample is collected by the specialist. Use a sterile spatula and scrape the lesions or ulcers and inoculate the sample in the transport medium or in a dry sterile tube without a screw cap or rubber stopper; spread on glass sheet.

- **Aspiration of vitreous fluid:** Use aseptic technique to perform puncture. aspiration

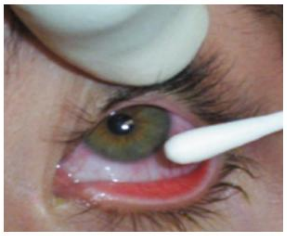

8.5.11.3. Transport

- Immediately after collection at room temperature.

8.5.12. EXTERNAL AUDITIVE CONDUCT

Sampling of otic secretions can be performed by tympanocentesis or by collecting spontaneous effusion from the middle ear after suppurative otitis media. The first is considered the method of choice because it has a lower risk of contamination and provides an immediate therapeutic solution for the patient (Pichichero, 2013). Swab collection of middle ear effusion is an option when it has occurred spontaneously (AS Adoga, 2010) .

Machine Translated by Google

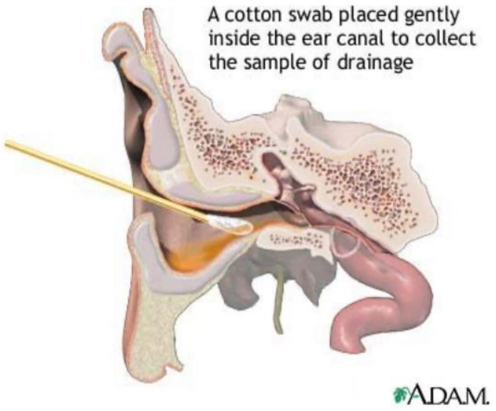

8.5.12.1 Care and Recommendations:

- Sampling of middle ear secretions and ocular secretions should be taken before starting local or systemic antibiotic treatments.
- Do not use ear drops 18 to 24 hours before taking the sample.
- Do not take or apply antibiotics 24-48 hours before

8.5.12.2. Transport

The transport of the samples to the laboratory should be carried out as soon as possible, maximum within the first two hours, after taking the samples, keeping them at room temperature. Refrigeration of the samples should be avoided until processing.

8.6 BLOOD CULTURES

Blood cultures have become the *gold standard* for the detection of bacteremia and fungemia. The recovery of circulating microorganisms in the blood of patients has great diagnostic and prognostic importance, since it indicates the failure of the patient's immune system to contain the infectious processes in their primary location. The presence of a positive blood culture allows establishing the etiological agent and the susceptibility of the microorganisms to antibiotics, facilitating their adequate treatment.

A main determining factor in the ability to obtain positive results and the isolation of the causative microorganism is related to the extraction of the adequate volume of sample, therefore the volume of blood that is cultured is crucial to achieve the detection of the microorganisms, since volumes Lower than optimal values can lead to false negative results.

Machine Translated by Google

8.6.1 Definitions

- **Antiseptic:** Substance that inhibits the growth and development of microorganisms.

- **Automated blood culture system:** Automated mechanical system that allows shaking, monitoring and incubating blood culture bottles to evaluate the growth of microorganisms.

- **Bacteremia:** It is the presence of bacteria in the bloodstream. Depending on the type of bacteria and growth time, it can be considered the cause of sepsis or a contaminating agent at the time of sample collection.

- **Blood culture:** Samples that are taken from a patient to evaluate the presence of bacteria or fungi in the bloodstream.

- **Blood culture set (sets):** This is the number of blood culture bottles (2 or 3 in adult patients and for the pediatric population it depends on the volume extracted) in which a sample of the patient's blood obtained from the same puncture site is planted. It usually consists of a bottle for the detection of anaerobic microorganisms and one for the detection of aerobic microorganisms.

- **Contaminant Agent:** Microorganism isolated from a blood culture, which was introduced into it during sample collection or processing of the blood culture, which is why it is not considered an etiological agent.

- **Culture Medium:** Substance enriched with factors necessary for the growth of microorganisms.

- **Disinfectant:** Substance that reduces the concentration of bacteria, fungi or viruses on a surface.

- **Fungemia:** Presence of fungi (hyphae or yeast) in the bloodstream.

- **Inadequate Blood Volume:** When the volume of blood inoculated into the blood culture bottle is less than 80% of the minimum volume required on the bottle label.

- **Sepsis:** Systemic inflammatory response syndrome resulting from an infection.

- **Venipuncture:** Puncture of a vein to obtain a blood sample.

Machine Translated by Google

• **Specificity:** Probability that the culture will not show growth if bacteremia is absent. That is, the specialty characterizes the ability of the test to detect the absence of the disease in healthy subjects. • **Sensitivity:** Probability that the culture will show growth if bacteremia is present: true positives. That is, sensitivity characterizes the ability of the test to detect the disease in sick subjects.

8.6.2 Conditions for taking the sample.

- Blood cultures must be obtained simultaneously in different punctures or with a short time difference between each one. Taking these at time intervals is indicated only when it is necessary to document continuous bacteremia in patients with suspected infective endocarditis or in endovascular infections.
- If possible, take blood cultures before starting antimicrobial therapy. The use of blood culture bottles with resins is recommended, which trap the antibiotic, facilitating the growth of microorganisms.
- In the case of blood cultures obtained from peripheral veins, it is recommended to use the median cubital vein or the cephalic veins of the upper limbs. It is not recommended to draw blood from already channeled peripheral lines; in the case of central lines, taking blood from them is indicated for the investigation of infections associated with it and always preceded by taking a blood sample from a vein. peripheral. • Blood culture bottles are not sterile on the outside,

which is why it is recommended to disinfect the rubber cap of the bottle using 70% isopropyl alcohol (CLSI, 2012). • The volume of blood obtained during blood culture collection is the factor

determinant in the recovery of the microorganism causing the infection. The isolation rate of pathogens obtained from blood cultures increases with the amount of blood taken.

- In adult patients, ideally a minimum volume of 10 mL per patient should be obtained. each set of blood cultures (volume extracted for each venipuncture). It is recommended to take two sets from two different anatomical sites (At the Shaio Clinic Foundation 2 sets are taken, each set includes an anaerobic and an aerobic blood culture)
- In neonates, some studies suggest that inoculating 1 mL of blood into the bottle may be sufficient volume to obtain adequate sensitivity when a single bottle is used.

- In neonates and premature babies, it is recommended to take two sets, each with a minimum volume of 0.5 mL.

Volume of blood to be extracted per set of blood cultures according to age and weight in

Machine Translated by Google

pediatric population

Población	Edad	Sitio	Volumen Minimo	Botellas
Neonatos	0-28 dias (o pacientes en URN)	vena periferica	<8 kg: 1 mL	Una botella pediatrica aerobica
Niños	1-3 meses	vena periferica	<8 kg: 1 mL	Una botella pediatrica aerobica
	3-36 meses	vena periferica	<8 kg: 1 mL<br>8-13 kg: 3 mL<br>13-27 kg: 5 mL	Botella pediatrica aerobica si el volumen es menor de 0,5 - 4 mL Bottella aerobica de adulto si el volumen es mayor de 4,0 mL
	4-11 años	vena periferica	8-13 kg: 3 mL<br>13-27 kg: 5 mL<br>27-40 kg: 10 mL >40 kg: 10 mL	Botella pediatrica aerobica si el volumen es menor de 0,5 - 4 mL Botella aerobica de adulto si el volumen es mayor de 4,0 mL
	12-17 años	vena periferica; considerar dos venas de sitios separados para 2 cultivos	27-40 kg: 10 mL >40 kg: 10 mL	Botella pediatrica aerobica si el volumen es menor de 0,5 - 4 mL Botella aerobica de adulto si el volumen es mayor de 4,0 mL

Tomado de: BLOOD CULTURES AND CENTRAL CATHETERS: IS THE "EASIEST WAY" BEST PRACTICE? Margo Halm, RN, PhD, ACNS-BC, Tracy Hickson, MLS (ASCP), CMSM, Deanna Stein, RN, Matthew Tanner, PharmD, BCPS, and Sheila VandeGraaf, PBT (ASCP)

8.6.3. Care and Recommendations

- Perform surgical hand washing
- Maintain aseptic technique throughout the procedure.
- Perform antisepsis on the area to be punctured; do not palpate the vein without sterile gloves once the skin is prepared.
- Use sterile gloves for each puncture

8.6.4 Collection technique

- Obtain each sample from different anatomical sites (NOT Arterial, NO limbs lower, generates greater complications and contamination).
- Before approaching the patient's environment to take this type of sample, you must perform hand hygiene. •

Disinfect the rubber cap of the blood culture bottle using sterile gauze. moistened with 70% isopropyl alcohol and let dry.

Machine Translated by Google

- Place the patient in the appropriate position, apply the tourniquet, select and locate the appropriate vein. To do this, palpate and follow the path of the vein in the arm with your finger. Verify that the venipuncture site is completely normal.

- Put on a hat, face mask, safety glasses or visual mask and gown. • Perform hand hygiene and immediately put on sterile gloves. • Perform asepsis and antisepsis of the venipuncture site with 3 pads soaked with 70% isopropyl alcohol, rubbing the area. Leave 30 seconds allowing the skin to dry for the first shot and 60 to 120 seconds for the second. In situations of hypersensitivity, the use of solutions with iodinated bases is recommended.

- Puncture the 10mL syringe immediately after removing the syringe, inoculate the anaerobic bottle and repeat the procedure of the other arm for the aerobic bottle(s).

- Mix the bottles by immersion.

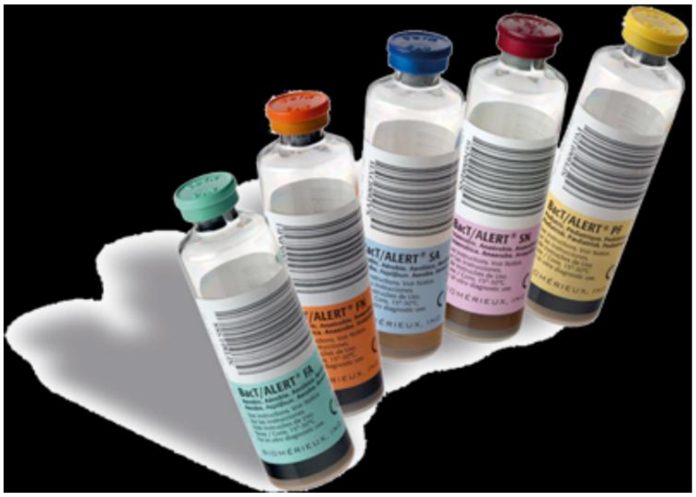

8.6.5. **Taking blood culture samples through a central catheter**

Machine Translated by Google

For sample collection via central catheter, in the case of suspected bloodstream infections associated with the device, a set of blood cultures should be taken simultaneously through a peripheral vein.

8.6.5.1 Collection Technique

• Before approaching the patient's environment and starting the procedure, you must perform hand hygiene. • Put
on sterile gloves. • Close the
intravenous infusion passage, for 3 – 5 minutes (depending on the patient's condition). • Choose a nearby port,
clean for 15 seconds using cloths impregnated with 70% isopropyl alcohol and allow it to dry.
• In adults, extract 20 mL of blood from the line and divide the contents of the syringe as follows: 10 mL for the anaerobic bottle and 10 mL for the aerobic bottle without changing the needle. Always inoculate the anaerobic bottle first and then the aerobic bottle. For the pediatric population, follow volume recommendations according to the age and weight of the patient. •
Mix the bottles by immersion.

8.7. CATHETER TIP

Quantitative culture (MAKI method) is the method that provides the greatest accuracy for making the diagnosis of a bloodstream infection directly related to the catheter.

Before taking the sample from the tip of the catheter, obtain the blood culture sets through peripheral venipuncture and the blood culture set through the central catheter.

8.7.1 Collection Technique

• Before approaching the patient's environment to take this type of sample, you must perform hand hygiene. • Put
on a hat, face mask, safety glasses or face mask. • Perform hand hygiene.
• Put on a surgical gown and
gloves. • Perform asepsis and antisepsis
of the catheter implantation site, leaving it for 30 seconds or 60 seconds if a solution with iodinated bases is used; allow it to dry.
• Remove the catheter and immediately cut the tip of the device 4 or 5 cm from the distal end using forceps and sterile scissors.
• Immediately place the cut segment in a sterile dry tube.

Machine Translated by Google

8.8. Transport

All blood culture bottles and catheter tip cultures should be sent to the laboratory as soon as possible, ideally within the first fifteen minutes and no more than two hours after the samples were taken. The delay in the entry of the bottles into the blood culture equipment can delay or prevent the detection of the growth of microorganisms.

After the blood culture bottles have been inoculated, it is recommended to keep them at room temperature for as little time as possible. Bottles should never be refrigerated or frozen due to the high risk of microorganism death (CLSI M47A,2012).

9.0 STERILE BODY FLUIDS

Sterile body fluids correspond to the liquids that are generated in the different body cavities which are completely isolated from the external environment and which can be reached by microorganisms through direct inoculation (trauma, iatrogenic), infection through the bloodstream. blood or infection of adjacent tissues.

Taking samples obtained through body cavities is an invasive procedure susceptible to complications. The procedure should be performed exclusively by trained medical personnel.

9.1 Definitions

- **Culture Medium:** Substance enriched with factors necessary for the growth of microorganisms.

- **Disinfectant:** Substance that reduces the concentration of bacteria, fungi or viruses on a surface.

- **Specificity:** Probability that the culture will not show growth if the infection is absent.

- **Sensitivity:** Probability that the culture will show growth if the infection is present.

- **Arthrocentesis:** Aseptic aspiration of fluid located in the joint cavity.

- **Thoracentesis:** Aseptic aspiration of fluid lodged in the pleural cavity.

Machine Translated by Google

• **Paracentesis:** Aseptic aspiration of fluid lodged in the abdominal cavity. •
Contaminant Agent: Microorganism isolated from a culture, which was introduced into the culture during sample collection or culture processing, which is why it is not considered an etiological agent.

• **Indeterminate Isolation:** Isolated clinical microorganism whose clinical importance is not established.

9.1.1 Conditions for taking the sample

• Samples from body cavities isolated from the external environment should be collected and taken to the laboratory for processing as soon as possible to guarantee the viability of difficult-to-grow microorganisms and avoid the overgrowth of contaminating bacteria.

• Fluids susceptible to clot formation should be collected in tubes with SPS (sodium polysulfonate) anticoagulants for special tests, except if searching for *Neisseria meningitidis, Neisseria gonorrhoeae, Peptostreptococcus and Gardenella vaginalis.* In this case, anticoagulant type heparin, sodium citrate and EDTA (ethylenediaminetetracetic acid) can be used.

9.2. Effusions from the pleural cavity (Thoracentesis)

Pleural effusions are often found accompanying bacterial pneumonias.
About 17% of community-acquired pneumonias are associated with pleural effusions, mainly in those patients who did not respond to early antimicrobial therapy. Thoracentesis is a diagnostic and sometimes therapeutic procedure.

9.3 Effusions from the peritoneal cavity (Paracentesis)

Abdominal paracentesis is the procedure by which ascitic fluid contained in the peritoneal cavity is removed. Microbiological studies are indicated when primary or secondary peritonitis is suspected; Positive cultures in the presence of a high PMN count could correspond to a diagnosis of peritonitis. (L Raka, 2012)

9.4. Effusions from the joint cavities (Arthrocentesis)

Arthrocentesis is one of the most frequently performed procedures in medical practice for the diagnosis and treatment of diseases of the joint cavity. Simple aspiration of excess synovial fluid from the joint can explain the causes of the disease and help in treatment by reducing intra-articular pressure.

Machine Translated by Google

9.5. Pericardial cavity (Pericardiocentesis)

Pericardiocentesis is a diagnostic procedure that can be used in clinical practice to diagnose the presence of fluid (blood, pericardial fluid, purulent material) in the pericardial cavity. In microbiological diagnosis, any growth of a pathogen in cultures has clinical significance.

9.6 Cerebrospinal Fluid - CSF (Lumbar Puncture)

The main diagnostic indications for CSF collection include infectious diseases, inflammatory diseases and neoplasms that compromise the central nervous system. The procedure is also indicated for the intrathecal administration of anesthetics, antibiotics, chemotherapy and antispastics.

9.7. Transport of samples

All samples, tubes and bottles collected must be labeled with the patient's name, identification number, type of specimen, date of collection and the type of study to be performed.

The transport of the samples to the laboratory should be carried out as soon as possible, ideally within the first 15 minutes and a maximum of two hours after taking the samples, keeping them at room temperature (L Raka, 2012). The medical order must include all of the tests requested for the procedure. Due to the difficulty in collecting these samples, actions must be taken to reduce the risk of pre-analytical errors in the laboratory.

10.0 SKIN AND SOFT TISSUES

Infections that involve the skin and soft tissues are those that include the entire skin, skin appendages, subcutaneous cellular tissue, fascia, and striated muscle.

The loss of skin integrity favors the colonization of the subcutaneous cellular tissue and becomes a nutritious and warm microenvironment for the colonization and proliferation of microorganisms. The characteristics of the wound (depth, location, skin quality, level of tissue perfusion) and the patient (quality of the immune response) will define the progression of the infection and the subsequent prognosis (PG Bowler, 2001).
The agents found within the normal microbial flora of the skin are *Corynebacterium spp,* coagulase-negative staphylococci, *Micrococcus spp* and *Aerococcus spp,* among others. On the other hand, beta-hemolytic streptococci, *Staphylococcus aureus, Enterococcus spp, Bacillus anthracis, Pseudomonas aeruginosa are considered pathogens.*
Anaerobic bacteria *(Bacterioides* spp, *Prevotella* spp, among others) are also

Machine Translated by Google

important etiological agents because they have been related to more than 38% of cases (Cercenado E, 2006).

10.1 Definitions

• **Microbial colonization:** Corresponds to the access and proliferation of microorganisms without causing infection.

• **Surgical wound infection:** It is defined as the infection that occurs from bacterial contamination caused by a surgical procedure.

• **Acute soft tissue infection:** Corresponds to infections that affect the skin, skin annexes, subcutaneous cellular tissue, fascia and skeletal muscle. U "fjf"
(Cercenado E, 2006). They include skin abscesses, traumatic wounds and necrotizing infections.

• **Bite infections:** Infection that is generated in a wound produced by the teeth of an animal or another person, through maceration, perforation or laceration of the superficial tissues of the patients. The most frequently isolated etiological agents are *S. aureus, Peptostreptococcus sp, Bacterioides spp* and *Pasteurella multocida.*

• **Burn infection:** It is diagnosed when changes appear in the appearance of the burn such as areas of local discoloration, edema at the wound margin, or rapid separation of the eschar.

• **Pressure ulcer:** Any ulcer or injury generated by sustained pressure against a bone surface or a firm plane, which can cause friction and shear responsible for ischemia of the superficial tissues.

• **Culture Medium:** Substance enriched with factors necessary for the growth of microorganisms.

• **Specificity:** Probability that the culture will not show growth if the Infection is absent.

• **Sensitivity:** Probability that the culture will show growth if infection is present.

10.2 Conditions for taking the sample.

Ideally, samples should be taken before starting empirical antibiotic therapy, from those lesions that present clinical signs of infection (change in appearance, poor healing) (Cercenado E, 2006). In cases of burn infections, samples

Machine Translated by Google

They should be obtained in the first days to weeks after the injury occurs.

10.3 Instructions for taking skin and soft tissue samples

It could be defined that skin and soft tissue samples can be of two types: wound tissues and wound fluids.

10.3.1 CLOSED WOUNDS

It is indicated when the presence of liquid collections in intact skin is clinically identified. It is also indicated in cases of surgical wounds or collections that are adjacent to open wounds covered with cellular debris.

10.3.2 OPEN WOUNDS

Taking samples for open wounds with swabs, although not the most recommended method, its ease and low invasiveness make it a convenient method for most open wounds. Any microorganism found deep in the wound will likely also be found on the surface, which explains the good correlation between quantitative cultures from biopsies and semiquantitative cultures obtained from swabs.

10.3.2.1 Care and Recommendations:

Clean the wound from the edge outward with gauze soaked with normal saline solution and 70% isopropyl alcohol, in order to avoid contamination of the sample with colonizing flora that is not really involved in the infectious process.
Wash the internal part of the wound with plenty of saline solution, without pressure. Do not use antiseptics.

10.3.3. Collection Technique:

ü Vacuum if possible or pass a swab inside the wound. Take the sample with two brushes.

ü If you use a means of transport, place one in said means and with the other make a spread on glass sheet.

ü If you do not have a means of transport, place the swabs in a sterile tube with a lid.

10.4 BIOPSIES AND CURETAGES

Acquisition of viable tissue through biopsy during initial debridement is the

Machine Translated by Google

most used method to determine the load and invasiveness of colonizing microorganisms in skin and soft tissue infections. The aseptically obtained tissue is homogenized, diluted and cultured in selective and non-selective media to provide qualitative and quantitative information. In suspected burn wound infection, samples obtained by biopsy have been the most used (Church D, 2006). The performance of this procedure must be carried out exclusively by trained medical personnel.

10.5 Transport of samples

Samples obtained in sterile syringes or bottles must be closed hermetically or ensuring that there are no drips or leaks that would allow the loss or contamination of the samples; Likewise, the swabs must be placed in sterile tubes.

The transport of the samples to the microbiology laboratory must be carried out as soon as possible at room temperature, a maximum of two hours after taking the samples (L Raka, 2012) (PG Bowler, 2001).

11.0 GASTROINTESTINAL TRACT

Gastrointestinal tract infections are the second most common type of infectious diseases, after respiratory tract infectious diseases. There are multiple presentations of gastrointestinal infections as well as the etiological agents that produce them (Spanish Society of Infectious Diseases and Clinical Microbiology, 2008).

For the diagnosis of diarrheal disease of the gastrointestinal tract, the type of sample of choice is stool with diarrheal characteristics. Compact stools or swabs should not be used for microbiological diagnosis.
In pediatric patients with suspected tuberculosis, it is indicated to take samples of gastric contents in order to perform cultures for *Mycobacterium tuberculosis.*

11.1 Definitions

• **Culture Medium:** Substance enriched with factors necessary for the growth of microorganisms.

• **Dysenteriform diarrhea:** type of diarrhea that is accompanied by abdominal pain, cramps, tenesmus and diarrheal stools with the presence of mucus and blood.

Machine Translated by Google

• **Choleriform diarrhea:** type of acute diarrhea that manifests itself with watery stools and presents as more than 3 liquid, yellowish stools, without evidence of blood, accompanied by vomiting, fever, decreased appetite and irritability.

11.2 Conditions for taking the sample.

In patients with suspected tuberculosis infection, the patient must be fasting and hospitalized to be able to perform the procedure.
In patients with acute diarrheal diseases, it is recommended to take the sample in the first days of the illness. *If parasites are suspected, take three samples on different days.* It is not recommended to use swabs to take the sample.

11.3 Gastric content for culture of Mycobacteria

This procedure is indicated in pediatric patients who do not have the ability to generate sputum. The gastric content obtained will be used to perform smear microscopy and to culture mycobacteria.

11.4 Fecal material

The microbiological study of fecal matter is indicated in patients with dysenteric disease, fever, bloody stools, nosocomial disease and persistent diarrheal disease. (E Baron, 2013; 57)

The use of fecal matter samples is also indicated in patients with suspected Clostrodium difficile infection .

11.4.1. Materials

ü Sterile wide-mouth container ü Non-sterile gloves

11.4.2. Collection Technique

ü Explain to the patient what the procedure consists of and the necessary amount of fecal matter needed for the analysis. ü
Perform hand hygiene. ü Collect
5 mL of diarrheal fecal matter or 2 to 4 grams of compact fecal matter in a sterile wide-mouth container.

Machine Translated by Google

ü In pediatric patients who use a diaper, do not place it in the absorbent position in order to obtain the sample more easily.

11.5 Transport of samples

Stool samples must be transported within the first two hours of being taken at room temperature and can be stored for up to 24 hours in refrigeration between 2°C and 8°C. In suspected C. difficile infection , samples for culture must be transported within the first hour of being taken and can be stored for up to 48 hours between 2 and 8°C; To search for cytotoxin, samples can be stored frozen for up to 72 hours at less than 60°C. To search for parasites, samples must be stored at room temperature. If Rotavirus is suspected, samples must be transported refrigerated between 2 and 8°C. (Spanish Society of Infectious Diseases and Clinical Microbiology, 2003)

In the case of gastric juice samples to search for mycobacteria, the samples must be transported in less than 15 minutes to the laboratory at room temperature.

12.0 GENITAL TRACT

Lesions of the genital tract can have multiple etiologies. The most frequently observed agents are *Chlamydia trachomatis, Neisseria gonorrhoeae, Treponema pallidum, human papillomavirus (HPV)* and Genital Herpes virus (HSV) among others. (J Aznar, 2007)

There are other genitourinary tract infections that do not originate from sexually transmitted agents; However, due to their characteristics, microbiological sampling of these lesions should be the same as that performed for sexually transmitted infections.

Genital tract infections can be divided topographically or syndromatically as (J Aznar, 2007):

ü Genital Ulcers: they can be painful or not painful. The most common agents are HSV, *Treponema pallidum* and *Haemophilus ducreyi.*

ü Urethritis and cervicitis: The most recognized corresponds to gonococcal urethritis caused by *Neisseria gonorrhoeae.* Non-gonococcal agents correspond to *Chlamydia trachomatis, Ureaplasma urealyticum, Mycoplasma genitalium* and *Candida sp.* among others.

Machine Translated by Google

ü Vulvovaginitis: In this case, the most frequent agents are *Candida sp., Trichomona vaginalis, Gardnerella vaginalis, Mycoplasma hominis, Staphylococcus aureus and Streptococcus agalactiae.*

The mixed bacterial flora found in the genital tract requires careful sampling, processing, and test interpretation with the sole objective of aiding in definitive diagnosis.

12.1 Conditions for taking the sample.

- Genital tract samples should be cultured as soon as possible to ensure the viability of difficult-to-grow microorganisms and to avoid overgrowth of contaminating bacteria.
- The patient should not apply suppositories or douche for 24 hours before the procedure. sampling
- Verify that adequate cleaning of the external genitalia has been carried out previously, to eliminate contaminating secretions.

12.2. Injuries or ulcers

For adequate sampling of this type of lesions, the possible etiological agents responsible for the infection must be taken into account; to ensure the viability of the sample.

Some of the infections that cause lesions or ulcers are:

1. Suspected Herpes Virus Infection *2.*
Suspected *Haemophilus ducreyi* infection *3.*
Suspected Syphilis infection
4. Suspected Donovanosis (inguinal granuloma)

12.3. Urethral exudate

Urethritis has similar signs and symptoms in men and women because the etiological agents are common in both. Urethritis are risk factors for developing infections of the upper genital tract such as Pelvic Inflammatory Disease, or in men orchidoepididymitis and prostatitis.

12.3.1. Care and Recommendations

•Verify that adequate cleaning of the external genitalia has been carried out previously, to eliminate contaminating secretions.

Machine Translated by Google

•The sample should be taken at least one hour after the patient has urinated.

12.3.2. Collection technique in men

• Put on personal protective equipment and non-sterile gloves. • If there is abundant secretion, with your free hand take the penis and with the other hand, collect the secretion using a swab.
• **If** no discharge is observed, with your free hand hold the penis and with the other hand, Carefully introduce the Dacron swab into the urethra about 2 cm and perform a rotation movement.
• Introduce the collected swabs into a means of transport that complies with growth conditions of the possible bacteria that may be found in the sample.

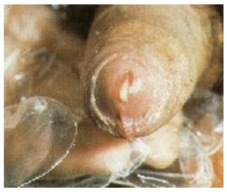

12.3.3, Collection technique in women

• Select a suitable light source that allows the procedure to be easily performed. • Place the patient in a gynecological position to take the sample. • Put on personal protective equipment and non-sterile gloves. • If there is abundant secretion, with your free hand separate the labia majora and with the other hand, collect the secretion using a Dacron swab. Take two or more swabs.

• **If** no discharge is observed, with your free hand separate the labia majora and with the other hand, carefully insert the swab about 2 cm and perform a rotating movement. Take two or more swabs. • On a slide, make a smear with one of the swabs to perform Gram stain.

• Introduce the collected swabs into a means of transport that complies with growth conditions of the possible bacteria that may be found in the sample.

Machine Translated by Google

12.4. Vaginal exudate

Vaginal exudate can be found in the presence of vaginitis, caused by *Candida sp* and *Trichomona vaginalis.* Likewise, taking a sample of vaginal exudate is essential for the microbiological diagnosis of bacterial vaginosis caused by *Gardnerella vaginalis.* If N. gonorrhoeae infections are suspected , cervical exudate sampling should be performed in conjunction with the vaginal smear. In the case of pregnant women, an active search for colonization by *Streptococcus agalactiae* must be carried out with the aim of providing prophylactic treatment during childbirth to avoid contamination of the neonate.

12.4.1. Collection Technique

- Put on personal protective equipment and non-sterile gloves. • With extreme care, insert a speculum through the vaginal introitus without using gel. lubricant. Open the speculum to visualize the vaginal cavity and the uterine cervix.
- Collect vaginal exudate deposited in the posterior vaginal fornix with Dacron swabs. Take two or more swabs. If the patient has had a hysterectomy, take samples from the posterior fornix.
- If N. gonorrhoeae infections are suspected , immediately take a blood sample. endocervical exudate.
- Insert the swabs immediately into the tubes with specific transport medium. (E Baron, 2013; 57)

12.5. Cervical exudate

The presence of cervical exudate can be found in the presence of cervicitis, endometritis, and chronic inflammatory pelvic disease. If gonococcal infection is suspected, a sample of cervical exudate should always be taken along with a sample of vaginal exudate. In pregnant patients, this type of sampling should be performed by experienced medical personnel. (Warren T, 2005) (J Aznar, 2007)

12.5. Collection Technique

- Put on personal protective equipment and non-sterile gloves. • With extreme care, insert a speculum through the vaginal introitus without using gel. lubricant. Open the speculum to visualize the vaginal cavity and the uterine cervix.
- Using a sterile Dacron swab, remove excess mucus from the surface of the cervix. Then discard the swab.

Machine Translated by Google

- Insert a new Dacron swab into the endocervical canal and rotate it 360° carefully. Repeat this procedure with a third Dacron swab.
- Insert the swabs immediately into the tubes with transport medium specific.

12.6. Bartholin's gland exudate

It is indicated in early stages of the disease.

12.6.1. Collection Technique

- Place the patient in a gynecological position to take the sample.
- Put on personal protective equipment and non-sterile gloves.
- With extreme care, insert a speculum through the vaginal introitus without using gel. lubricant. Open the speculum to visualize the vaginal cavity and the uterine cervix.
- Take a sterile Dacron swab and insert it into the Bartholin's gland, rotating it against the walls. Repeat this procedure three times to obtain samples for detection of *N. gonorrhoeae, C. trachomatis* and common germs.
- Insert the swabs immediately into the tubes with specific transport medium. (E Baron, 2013; 57)

12.7 Prostatic secretion

Taking samples of prostate secretion should be done after performing intrarectal prostate massage. It is indicated in patients with clinical suspicion of infectious prostatitis, epididymitis and/or orchitis. (L Raka, 2012) (E Baron, 2013; 57)

12.8. Semen

Semen sampling should be collected in a sterile wide-mouth bottle after local stimulation by the patient through masturbation.

12.8.1. Collection Technique

- Provide the patient with an intimate space to take the sample.
- Instruct the patient to perform hand hygiene, wash the penis and meatus. urinary before proceeding to take the sample.
- Instruct the patient to take the sample through personal stimulation through masturbation. The sample should be collected in a sterile wide-mouth bottle.

Machine Translated by Google

• Completely close the sample container bottle.

12.9. Transport of samples

The transport of the samples to the laboratory should be carried out as soon as possible, a maximum of two hours after taking the samples, keeping them at room temperature (L Raka, 2012). If cultures for *Chlamidia trachomatis* have been requested, the samples must be transported in the shortest possible time to the laboratory under refrigeration at 4°C (E Baron, 2013; 57).

13. RECTAL TRACT

13.1. Rectal swab for screening in pregnant patients

In pregnant women, rectal swabs are mainly used to search for *S. agalactiae* with the aim of administering prophylactic antibiotics during childbirth, to reduce the risk of colonization of the newborn by this microorganism.

- Take a culturette (swab) and insert it approximately 1 to 2 cm into the anal sphincter

- Rotate the swab
- Introduce the swab into the transport medium and take it to the laboratory for processing.

13.2. Rectal swab for men pre biopsy

In adult patients who are going to undergo surgical procedures, such as prostate biopsies, a rectal swab culture will be performed in order to evaluate the presence of Gram-negative bacilli that produce extended-spectrum beta-lactamases (ESBL).

-Take a culturette (swab) and insert it approximately 1 to 2 cm into the anal sphincter

- Rotate the swab
- Introduce the swab into the transport medium and take it to the laboratory for processing.

13.3. Rectal swab for screening for multidrug-resistant Gram-negative bacilli

Machine Translated by Google

In order to actively search for patients carrying or colonized by multidrug-resistant Gram-negative or carbapenamase-producing bacilli, it is established that a rectal swab will be performed on all patients admitted through the emergency department or patients who are going to enter the Care Unit. Intensive Care Units (ICU) to rapidly detect this type of microorganisms in order to carry out timely preventive isolation of these patients and avoid possible outbreaks within our institution.

-The laboratory will provide the respective sample collection kit, which consists of 2 swabs and 1 glass tube with 2 mL of saline solution.
-Introduce a swab and insert it approximately 1 to 2 cm into the anal sphincter
- Rotate the swab
- Insert the swab into the glass tube with saline solution and take it to the laboratory for processing.

14. COLLECTION OF SAMPLES FOR PATHOLOGY

• Surgical specimens taken from operating rooms should be sent to the clinical laboratory in an airtight container with 10% formalin and then processed by the histotechnologist.

• Liquids must be sent in a sterile screw-cap tube without including formaldehyde or any other type of solution to be processed in the area.

• Surgical ones such as kidney or skin biopsies that require processing of Electron microscopy the following procedure should be performed:

ü Notify the department one day in advance of taking the biopsy and communicate with the pathology secretary on the day and time of the procedure.
ü On the day of the procedure, notify one hour in advance and confirm the carrying out the procedure to the secretary.
ü When taking the biopsy, the specimen must be included in saline solution and sent immediately for its respective shipment to the reference laboratory.

NOTE: All waste from failed sample collection becomes pathological waste, so these tubes will be placed in a small red bag, identified or labeled as “Pathological Waste” with the date and time to be taken to the laboratory. in the sample transport container and carry out the corresponding segregation in the clinical laboratory department.

14.1 TRANSPORTATION OF SAMPLES TO THE LABORATORY

Once taken, they must be taken to the laboratory in the shortest possible time and be

Machine Translated by Google

delivered to the sample reception window in the clinical laboratory for processing.

- If the patient is an outpatient, the sample is brought by him or her or a family member, the respective order must be delivered with the patient's information, description of the sample, signature and seal of the doctor who performed the intervention.

The patient and/or family member bills and pays for the biopsy at the clinical laboratory cash register.

- Biopsies that arrive from surgery rooms are delivered to the laboratory by the responsible orderly. Each biopsy must come with its request, identified and labeled. They are received by the laboratory assistant, who checks the appearance of the sample and that the request for the corresponding exams appears in the system and signs the receipt in the service book.

NOTE: The orders are uploaded by the pathology secretary from Monday to Friday, during non-business hours they must be uploaded by billing, after which the samples are delivered to the laboratory.

15.0 BASES FOR INTERPRETATION OF THE RESULTS OF LABORATORY SAMPLES

PROOF	VALUE OF REFERENCE	CLINICAL INTERPRETATION
Amylase	Serum 30-110 U/L<br>Urine 32-641 U/L	It is a marker for acute pancreatic disease.<br>Serum amylase activity increases within hours (6 to 48 hours) in patients with acute pancreatitis; Values above 500 U/l have diagnostic value. Amylasuria rises rapidly, hours after elevation in serum, and remains elevated longer than in serum.
Ammonium	9-30 mmol/L	The most common cause of ammonia alteration is severe liver disease and chronic or acute kidney disease. Controlling ammonium levels in the blood is very useful in the treatment of hepatic coma
Total bilirubin and direct	Total: 0.2-1.3 mg/dL<br>Direct:0.0-0.3 mg/dL<br>Indirect: 0.0-0.1 mg/dL	Evaluation, classification and monitoring of both adult and neonatal jaundice. Hyperbilirubinemia is classified as conjugated and unconjugated. The conjugate is hepatic, cholestatic, primary biliary cirrhosis, hepatitis, familial jaundice and the post-hepatic one due to biliary obstruction due to stones, cancer or malformations of the bile duct. Unconjugated hyperbilirubinemia is classified as prehepatic (hemolytic states and extensive hematomas).

Machine Translated by Google

		and hepatic (Gilbert and Crigler-Najjar syndrome and neonatal jaundice).
**Ionic calcium**	Serum 1.15-1.32 mmol/L	There is hypercalcemia due to malignant tumors and primary hyperparathyroidism and there is hypocalcemia due to renal failure, hypoparathyroidism, vitamin D deficiency and secondary hyperparathyroidism.
Creatine Kinase Total CK-CPCreatin Kinase fraction MB	Men 55-170 U/L<br>Women 30-135 U/L<br>0-16 U/L	The CK MB/CPK offers a relative index that allows establishing the occurrence or not of acute myocardial infarction (MI). Vigorous exercise such as jogging or running can produce elevation of isoenzymes to levels similar to those of MI.
Chlorine	Serum<br>98-107 mmol/L	Hypochloremia due to chlorine loss occurs in gastrointestinal disorders, mineralocorticoid ketoacidosis, and kidney diseases, diabetic, under treatment excess with ethacrynic acid, ACTH, corticosteroids, mercurial diuretics and furosemide. Hyperchloremia occurs in metabolic acidosis due to loss of bicarbonate, in hyperalimentation of ammonium chloride salts.
Coproscopic	PH, Occult blood<br>Leukocytes (segmented cells): neutrophils, lymphocytes, eosinophils.<br>Reducing sugars<br>Glucose Sucrose Fats<br>Fungi Parasites<br>(troozoites)<br>Bacterial flora	Useful for the diagnosis of parasitic infestation, obstructive jaundice, diarrhea, malabsorption, rectosigmoid obstruction, dysentery, ulcerative colitis and gastrointestinal bleeding.
Creatinine	Serum<br>0-5 years 0.2-0.4 mg/dL<br>6-12 years 0.3-0.7 mg/dL<br>Men 0.66–1.25 mg/dL<br>Women 0.52-1.04 mg/dL	Determination of serum creatinine is mainly used to evaluate kidney function, it is elevated in kidney damage in musculoskeletal necrosis, trauma, progressive muscular dystrophy, amyotrophic lateral sclerosis, congenital amyotonia, dermatomyositis, myasthenia gravis,<br><br>prolonged fasting, hyperthyroidism and diabetic acidosis
Blood count complete	Leukocytes 4.5-11.0 (10*3 / mm3)<br>Erythrocytes 4.2-6.20 (10*6/ mm3)<br>Hemoglobin 14.0-17.0 g/dL<br>Hematocrit 36-54%<br>VCM 86-96 mm3<br>HCM 25-31 pg<br>MCHC 32-38 g/dL<br>Platelets 150-450 (10*3/mm3)<br>Erythrosedimentation:	Its greatest usefulness is in the differential diagnosis of anemias, in the evaluation and characterization of proliferative lesions of the hematopoietic system and in the evaluation of infectious processes.<br>The platelet count is determined in patients with suspected hemorrhagic disease, purpura or petechiae, prolonged bleeding time, leukemia, lymphoma, chemotherapy, and to determine the response of patients receiving platelet transfusions.

Machine Translated by Google

	0-20 mm/hour.	

PROOF	VALUE OF REFERENCE	CLINICAL INTERPRETATION
Nitrogen Ureaic (BUN)	Serum	BUN generally does not increase significantly until Men 9-20 mg/dL that glomerular filtration is not decreased at least in a Women 7-17 mg/dL 50%, therefore it is not an early indicator of kidney damage.
Culture and antibiogram bacteriological	Negative culture for isolation, identification and antibiogram in the different types of pathogenic bacteria that pathogenic germ	are involved in certain infections human.
Dehydrogenates sa lactic acid (LDH)	120-246 U/L	LDH is measured primarily to diagnose conditions in which there is tissue damage.
D-dimer	Less than 550	Indicator of activity of the fibrinolytic system in cases of disseminated intravascular coagulation. Screening of patients with suspected deep vein thrombosis or pulmonary thromboembolism. It is also elevated in hemorrhages, hematomas and in thrombolytic therapy. False positives occur due to rheumatoid factor or pregnancy.}
Fibrinogen (Factor I)	180-350 mg/dL	It is used to determine congenital or acquired fibrinogen deficiencies and to control the severity and treatment of disseminated intravascular coagulation and fibrinolysis phenomena. It is found at high levels in acute and chronic inflammatory diseases, nephrotic syndrome, chronic glomerulonephritis, hypernephron, hepatitis, hepatoma, cirrhosis, pregnancy, compensated intravascular coagulation and in estrogen therapy.
Alkaline phosphatase (FALK)	38-126 U/L<br>Children 0-600 U/L	It is elevated in intra- or extrahepatic cholestasis (hepatitis, biliary cirrhosis, hepatotoxic drugs, choledocholithiasis, carcinoma of the head of the pancreas, liver carcinoma), in bone diseases such as osteitis, rickets, osteomalacia, consolidation fractures, osteoblastic tumors and hyperparathyroidism. It can also be elevated in heart failure, infectious mononucleosis, MI, intestinal perforation and septicemia.
Spectrum Glutamyl Transferase (gGT	Men 15-73 U/L<br>Women 12-43 U/L	Microsomal enzyme found mainly in the liver, pancreas and kidney. It is indicated in the evaluation of cholestatic liver disease and liver injury induced by drug and alcohol abuse. It is elevated together with FAL in pancreatic and liver diseases.
Glucose	Fasting: 74-106 mg/dL<br>2 hours post charge: < 140 mg/dL<br>urine: < 30 mg/dL<br>CSF: 40-70 mg/dL	Fasting determination and glucose load tolerance testing serve to establish the diagnosis of Diabetes Mellitus and carbohydrate disorders. It is also used to monitor treatment in diabetics and patients with dehydration, coma, hypoglycemia, insulinoma, acidosis and ketoacidosis.

Machine Translated by Google

Lipase	8-78 U/L	Serum lipase rises rapidly in patients with pancreatic lesions such as acute and recurrent pancreatitis, abscess or pseudocyst, trauma, and carcinoma; It is also elevated in common bile duct obstruction, peritonitis, infarction and intestinal obstruction, abdominal abscesses, kidney failure and by the action of some drugs such as anticholinergics and opiates.
		Evaluation of alterations due to malabsorption, pancreatitis, renal clearance disorders and control of the treatment of pregnancy toxemia. Hypomagnesemia is associated with hypocalcemia, chronic alcoholism, malnutrition, malabsorption, Serum Magnesium: 1.6-2.3 mEq/L chronic hemodialysis, prolonged gastric drainage, acute pancreatitis, hypoparathyroidism, glomerulonephritis, hyperaldosteronism and pregnancy. Hypermagnesemia occurs in patients with kidney failure, dehydration, and Addison's disease.
Total proteins and A/G ratio	Totals: 6.3-8.2 g/dL<br>Albumin: 3.5-5.0 g/dL<br>Globulins: 2.8-3.2 g/dL	Serum protein concentration primarily reflects decreased hepatic synthesis or renal protein loss.<br>Elevation of total proteins is found in dehydration, multiple myeloma, hyperglobulinemia, granulomatous diseases, collagen diseases and certain tropical diseases.<br>There is a decrease due to a low protein diet, malnutrition, malabsorption, severe liver disease, extensive burns, chronic alcoholism, heart failure, neoplasms, overhydration, kidney disease.
C-reactive protein (PCR)	Adults: 0 - 5 mg/l<br>Newborn: <0.6 mg/l<br>Children from 4 days to 1 month: <1.6 mg/l	CRP is the most sensitive in the acute phase. It rises two hours after an acute injury, peaks and begins to decrease after 48 hours. It is a more sensitive indicator of inflammatory processes than erythrocyte sedimentation rate and leukogram.

PROOF	VALUE OF REFERENCE	CLINICAL INTERPRETATION
Potassium	Serum<br>3.5-5.1 mmol/L	Useful to detect metabolic states where variations in serum and/or urinary potassium concentration occur, such as hydroelectrolyte imbalance, arrhythmias, muscle weakness, hepatic encephalopathy, kidney failure.<br>There are medications that physiologically raise potassium such as amiloride, captopril, cyclosporine, danasol, enalapril, epinephrine, heparin, histamine, nifedipine, spirinolactone and terbutaline. There is a decrease in diuretics, amphotericin, cisplatin, corticosteroids, insulin, theophylline, sodium penicillin and sodium bicarbonate.
SARS-COV-2 (COVID-19) ANTIBODY<br>Yes	Negative	The results of this test cannot be used for diagnosis or exclusion of SARS COV-2.<br>A positive result requires confirmation. A negative result does not rule out the possibility of infection.<br>It is recommended to carry out an exhaustive analysis of the patient's clinical condition.

Machine Translated by Google

SARS-COV-2 (COVID-19) PCR-TIME REAL (PCR-RT)	Negative	Negative results do not exclude SARS-COV-2 infection and should not be used as the sole basis for patient management. Negative results should be combined with clinical observations, patient history, and epidemiological information.
SARS-COV-2 (COVID-19) Filmarray RT BIOFIRE PCR	Negative (Not detected)	Negative results do not exclude SARS-COV-2 infection and should not be used as the sole basis for patient management. Negative results should be combined with clinical observations, patient history, and epidemiological information.
Sodium	plasma: 137-145 mmol/L	It is elevated in acid-base imbalance in decompensated patients Serum or hemodynamically.
Transaminase Glutamicopyruvi AC GPT (ALT) Transaminase Glutamic oxalacetic GOT (AST)	ALT Men < 50 U/L Women <35 U/L AST Men 17-59 U/L Women 14-36 U/L	They are significantly elevated in hepatitis and hepatic necrosis of different etiologies and at a lower level in cirrhosis, obstructive jaundice, metastatic carcinoma, hepatic congestion and intrahepatic cholestasis. There may be slight elevation in AMI and acute pancreatitis, although the elevation of AST in AMI is more significant.
Time of Prothrombin (PT) and INR (Radio International Normalized)	PT 12.8-15.8 standard dose of of 2.5-3.5 for a dose of high of coumarins.	The PT evaluates coagulation disorders that compromise the second extrinsic system and the common coagulation pathway. INR2.0-3.0 for a of vitamin K, liver disease, hypofibrogenesis and coumarin coagulation. disseminated intravascular (DIC). Its greatest usefulness lies in the control oral anticoagulation with coumarins.
Part time Thromboplastin (PTT)		Test sensitive to all factors involved in the intrinsic coagulation system, especially functional deficiencies 24.6-31.2 seconds of factor VIII, IX, XI and XII. It is useful to monitor the effectiveness of heparin therapy.
Troponin I quantitative	and elevated cardiac Women: 0-15.5 pg/ml	Acute MI is usually diagnosed by chest pain, electrocardiographic changes , enzymes. In pg/ml In the last two decades, the most used has been the MB isoenzyme, without However, its specificity is not as good as that of TnI since CKMB is also elevated in acute and chronic disease.

Machine Translated by Google

Urinalysis	Aspect<br>Color: transparent yellow<br>pH: 5-7<br>Density: 1001-1030<br>Protein, bilirubin, ketones, nitrites, negative blood and glucose.<br>Urobilinogen: 0.2-1.0 Erlich U/dL<br>Leukocytes: 1-2 per field<br>Epithelial cells: few per field<br>Hyaline cylinders: occasional per field<br>Bacteria: rare<br>Erythrocytes: 0.2 per field. Crystals: occasional (phosphates) mucus	Urinalysis is helpful in the diagnosis, evolution and treatment of urinary infection and kidney diseases.
Opioids, amphetamines and cocaine	Negative	Detection of drugs of abuse.
**PROOF**	**VALUE OF REFERENCE**	**CLINICAL INTERPRETATION**
Benzodiazepine ace	benzodiazepines and	Screening or presumptive test to detect the presence of Negative < 12 ng/dL their metabolites in serum.
Negative < 60 ng/mL		Screening or presumptive test to detect the presence of barbiturates Barbiturates and its metabolites in urine; its presence indicates use for three days prior to determination
Antidepressants	Therapeutic ranges (ug/l):Amitriptyline 120-250<br>Nortriptyline 50-150<br>Desipramine 75-160<br>Inipramine 150-250	Monitoring of treatment to avoid toxic levels and in tricyclic cases where ingestion is suspected as a suicide attempt.
Ethyl alcohol (ethanol	< 10 mg/dL	Determines the concentration of ethyl alcohol in the blood to make the Negative correlation with the degree of intoxication or recent ingestion.
methyl alcohol (methanol)	Negative	Because it is a qualitative method, it is not possible to determine the degree of intoxication and for this it is necessary to clinically evaluate the patient.<br>This type of poisoning is a true toxicological emergency, therefore the result must be reported quickly.

Machine Translated by Google

Valproic acid	Therapeutic range: 50-100 ug/mL	Treatment monitoring due to large individual differences in the doses required to achieve effective therapy and in order to avoid toxic effects of the drug due to overdose.
Amikacin Carbamazepine to	Peak 20-25 ug/mg<br>Valley < 5 ug/mg<br>4-10 ug/mg	Control blood levels of the antibiotic for treatment efficiency and to avoid toxicity. Control of therapeutic levels
Lithium	Therapeutic range: 1.0-1.2 mmol/L<br>Potentially toxic range: >1.5 mmol/L<br>Severe toxicity: > 2.5 mmol/L	Control of therapeutic levels and to avoid toxicity due to overdose.
Digoxin	Therapeutic range: 0.6-2.0 ng/mL	Determine therapeutic levels and avoid toxicity due to excess medication; Ranges above 2.7 mg/mL should be a warning to reduce the dose.
Phenytoin	Therapeutic range:<br>Children >3 months and adults: 10-20 ug/mL<br>Children <03 months: 6-14 ug/mL<br>Free phenytoin: 1-2 ug/mL	Determination of therapeutic levels.
Phenobarbital	Therapeutic range: 10-30 ug/mL	Determination of therapeutic levels
Tacrolimus (FK-506)	Therapeutic range: 5-20 ng/mL	Treatment and control of post-transplant rejection organs.
Vancomycin	Therapeutic range:<br>Peak 30-40 mg/mL<br>Valley 5-10 mg/mL	Determination of therapeutic levels.

16.0. SAMPLING PROTOCOL

16.1 FLOOR SAMPLING PROTOCOL

ACTIVITY	DESCRIPTION	REGISTER YOU	RESPONSES ABLE
Application for exams	1- The doctor must request the exams directly in the system through medical history and indicate the time of sample collection	Clinic history	Floor treating doctor

Machine Translated by Google

laboratory hospitalization services n general	2- Make the exam request taking into account the following recommendations:<br>- Enter the CUPS code corresponding to each exam.<br>**- Clearly indicate the date and time when the exam is required.**<br>**- Independently record orders that are required for different days or times.**<br>- Make the observations corresponding to the type of sample (cultures) and number of blood cultures required so that they are also recorded.	stories Clinics	Treating physician
IDENTIFY R SAMPLES EARRING S BY TAKE	Once the order is entered, it remains on a preliminary list of patients pending to be taken.	Clinic history. Laboratory software	Biller Laboratory assistant
TRANSFER OF ORDERS	1- Transfer the orders that appear in the system, taking into account the date and time indicated for sample collection. If there is no indication in this regard, the order is transferred and the collection is left for the next round.	Software Laboratory	Bacteriologist to Laboratory assistant
	2- TRANSFER:<br>On the intermediate screen, review the pending orders to be taken<br>- Highlight the order that needs to be transferred and double click.<br>- The stickers are automatically printed.<br>- - Review the requested STUDIES in the system and confirm that you do not have pending exams, or those requested in duplicate. If you have pending tests, confirm in the corresponding section if it is necessary to take the sample, print the stickers so that they can be taken.<br>- Verify the room number with the patient list.<br>- Organize the orders by floors	Software Laboratory	Bacteriologist to Laboratory assistant

Machine Translated by Google

	correspond to that round.		
LOCATION OF THE PATIENT	3- Locate the patient on the corresponding floor, verify the names – surnames and ID of the patient.	Fingering voucher or stickers	Bacteriologist to
infection or SAMPLE	Before starting the process, wash your hands and use new gloves.<br><br>Perform cross identification with the patient, visual identifier in the patient room, handle or nursing staff.<br><br>Explain to the patient the procedure that is going to be performed.<br><br>Use personal protection elements, according to the type taken (preventive isolations, transplants, risk of airborne by contact, etc.) and according to the type of exam stickers (blood cultures).<br><br>Locate the arm and venipuncture site. Remember not to take samples on the arm that has fluids, fistulas, wounds or lesions.<br><br>Place the tourniquet so that the pressure is not excessive, as this interferes with laboratory tests.<br><br>Perform asepsis on the venipuncture site with a cotton ball moistened with alcohol, clean in a circular motion from the inside out and wait for it to dry, DO NOT BLOW.	of patient to be	Bacteriologist to
TAKING OF SAMPLES EARRING Yes	In case of difficult veins, patients undergoing procedures or room changes, do not release the tourniquet , take the necessary tubes according to the following order, you should ask a Bacteriologist for help. , take into account the order of intake and volume of the same shift.<br><br>required per tube, mix gently by inversion the number of times indicated for each In extreme cases where tube cannot be taken.<br>a sample for difficult vein, notify the floor manager		

Remove the needle from the vein , place a clean, dry cotton pad, apply gentle pressure with a straight arm and open hand. Place a tape

Machine Translated by Google

	looking for help.<br><br>**The recollection of samples to confirm a result must be carried out by the nursing staff in the shortest time, after the sample is requested by the laboratory.**		
IDENTIFY TION OF THE SAMPLES	Mark each tube in front of the patient with the initials of the patient's full name, leaving them visible so that the information can be verified with the corresponding stickers.	sticker	Bacteriologist to
	Samples that arrive taken at the laboratory must be recorded in the sample delivery notebook, noting the type of sample, full name of the patient, the person delivering and the person receiving, time and service.	Sample delivery notebook	Stretcher flats<br><br>Laboratory assistant
TRANSPORT I GIVE YOU SAMPLES TO THE LABORATORY RIVER	For safety, all samples should be handled as potentially infectious, even when the risk of infection is not evident.<br><br>Place the samples in the sample collection lunch box , and take them to the central laboratory in the shortest time possible. Keep in mind that the tubes should not lie down during the journey.<br><br>Samples must be taken to the laboratory once the round is finished.<br><br>Verify that all samples are delivered.<br>In case of a spill, clean immediately with the appropriate disinfectant. (Quaternary Ammonium)<br><br>Deliver the samples to the laboratory assistant.	Samples taken	Laboratory assistant<br><br>Bacteriologist to
RECORD OF ENTRY AND DISTRIBUTION ON DE SAMPLES	Once the samples are received, the assistant must record the samples that were received:<br>ORDERS- RECEIPT OF SAMPLES – PASS THE BARCODE AND CONFIRM ENTRY.<br><br>This process also applies to samples that arrive taken from the floor.<br>Distribute the samples in the different sections of the laboratory.	SAMPLE Yes	ASSISTANT OF LABORATORY RIVER

Machine Translated by Google

TAKING OF GLUCOMET RIAS	Request for exams by system.<br>Glucometry measurements are carried out by the nursing staff.	System exam request	Nurse Boss
TAKING OF CROPS	AEROBIC, ANAEROBIC, FUNGI CULTURES AND MYCOBACTERIA:<br><br>Skin or soft tissue wounds, samples from the respiratory and gastrointestinal tract, catheters, urine cultures, should be taken by nursing staff. And then be taken by the orderly to the laboratory in the shortest time possible.<br><br>CULTURE AND SPRAY OF VAGINAL FLOW: The collection of these samples depends on the clinical laboratory.<br>Patients who can be mobilized should be taken to the laboratory at the time requested.<br><br>Patients who cannot be transferred are taken directly to the floor by the nursing staff.<br><br>LIQUIDS: They must be taken by the treating doctor or specialist. Sufficient quantity must be sent according to the number of exams required. Pleural, synovial, joint, and peritoneal fluids must come in a sterile lilac tube to prevent them from clotting. CSF can come in a sterile screw-top tube.<br>Fluids that require ADA must come in a tube roundabout with citrate (blue)	Request for exams by<br><br>system.<br><br>Micro biology sampling manual	Nursing staff<br>stretcher bearer<br>Laboratory assistant<br>Doctor<br><br>clinical laboratory<br><br>Treating physician
TAKING OF HEMOCULTI YOU	Blood cultures are taken by the clinical laboratory. Except in the ICU where patient management is exclusive to nursing.<br><br>To take the sample, you must follow the protocol specified in the MICROBIOLOGY SAMPLE TAKING MANUAL. Verify the number of blood cultures in the system requested<br><br>Blood cultures should be taken as soon as possible, and<br>The delivery of the sample must be done as soon as possible.	Order by system.	Bacteriology to Personal of Nursing

should be recorded in the sample delivery notebook specifying the type of sample, full name of the patient, time of delivery, name of the person delivering, name

Machine Translated by Google

	PROTOCOL ANNEX No 2, by laboratory personnel<br><br>Blood cultures derived from catheters must be taken on the floor by the nursing staff.<br>Along with a catheter tip culture and a peripheral blood culture.		

16.2. SAMPLING PROTOCOL FOR INTENSIVE CARE UNITS

ACTIVITY	DESCRIPTION	RECORD<br>Yes	RESPONSE BLE
RESPONSIBILITY TAKE LITY OF THE SAMPLE	In the ICU, sample collection will be carried out as follows:<br><br>**SIDE:** Blood samples must be taken by clinical laboratory personnel.<br>When it is necessary to start an antibiotic regimen, the first blood culture must be taken by the nurse.<br>Secretions, smears and other cultures must be taken on the floor and the samples sent to the laboratory in the indicated time.<br><br>**ROTUNDA:** Due to the high risk of contamination that the patients on the side handle, it was agreed that these patients will be managed by the nursing staff in their entirety, including taking blood cultures and cultures in general.	Minutes of meeting with department nursing	Department head or nursing<br><br>Department head or clinical laboratory
TAKING OF SAMPLES BY CATHETER	When samples are taken through the arterial line, a 20 cc purge should be done.<br><br>In the roundabout when it is strictly necessary to take samples through the arterial line, it must be carried out by the nursing staff.<br><br>Filling the tubes must be carried out as follows:	Sample taking protocol ICU	Nursing

Machine Translated by Google

	1- the blue tube<br>2- the yellow tube<br>3- green tube<br>4- lilac tube<br>5- gray tube<br><br>Once the filling is done, mix by inverting and make sure to cover the tube very well.		
TAKING THE SAMPLE BY VENOPUNCI ON	Before starting the process, you must wash your hands and use new gloves.<br><br>Cross-identify patient...<br><br>Explain to the patient the procedure that is going to be performed whenever possible and the patient is consenting.<br><br>Use personal protection elements, depending on the type of patient to be taken (preventive isolation, transplants, risk of airborne or contact infection, etc.) and according to the type of examination (blood cultures).<br><br>Locate the arm and venipuncture site. Remember not to take samples on the arm that has fluids, fistulas, wounds or lesions.<br><br>Place the tourniquet so that the pressure is not excessive, as this interferes with laboratory tests.	System exam request	Bacteriologist Nursing
IDENTIFICATION ON OF THE SOURCE THE SAMPLE	Carry out asepsis of the venipuncture site. Once the sample is taken, mark it with a cotton pad moistened with alcohol, clean the origin of the sample of any stickers: circular shape from the inside out and wait for it to dry, DO NOT BLOW.<br>or sample taken by catheter<br>or sample taken by venipuncture<br>As soon as blood begins to flow, release the tourniquet,	Sample taking protocol ICU	Bacteriologist Nursing
IDENTIFICATION ON OF THE SAMPLES	take the necessary tubes according to the order taking into account the order of collection<br>from the intensive care unit must be identified with Sample sticker and patient.<br>required volume per tube, mix gently by inversion the number	Patient sticker	Bacteriologist Nursing
to the laboratory in a stretcher notebook.	of times indicated for TRANSPORT. Samples must be sent each tube.		

Remove the needle from the vein , release the tourniquet, place a clean, dry cotton pad, apply pressure

Machine Translated by Google

E DE SAMPLES TO THE LABORATORY EITHER	as little time as possible and verify that they are recorded in the sample entry notebook stipulating the person receiving and the time.	sample entry record	Laboratory assistant
TAKING OF HEMOCULTURE YOU	The collection of blood cultures on the lateral is carried out by the clinical laboratory. Except in the ICU roundabout where patient management is exclusive to nursing.<br><br>Blood cultures should be taken according to institutional protocol.<br><br>Blood cultures derived from catheters must be taken on the floor by the nursing staff. Along with a catheter tip culture and a peripheral blood culture.	Order by system.	Bacteriology Personal of Nursing

16.3. EMERGENCY SAMPLE TAKING PROTOCOL

ACTIVITY	DESCRIPTION	RECORD Yes	RESPONSIBILITY YOU
APPLICATION OF THE EXAMS OF LABORATORY EITHER	The treating doctor must request the examinations directly through the system in the medical history program	History Clinic	Emergency service doctor
IDENTIFY SAMPLES EARRINGS FOR TAKING	Once the order is entered, it remains on a preliminary list of patients pending to be taken.	Clinic history. Laboratory software	Laboratory assistant
TRANSFER OF ORDERS	Transfer the orders that appear in the system, taking into account the time indicated for sample collection.	Software Laboratory	Laboratory assistant
	2- TRANSFER:	Software	

Machine Translated by Google

	On the intermediate screen, review the pending orders to be taken<br>- Highlight the order that needs to be transferred and double click.<br>- The stickers are automatically printed.<br>- Review the requested STUDIES in the system and confirm that there are no pending exams, or those requested in duplicate. If you have pending exams - Check the room number with the patient list.<br>- Organize the orders that correspond to that round by floor.	Laboratory Assistant	Laboratory
TAKING THE SAMPLE	- Before starting the process, wash your hands and use new gloves.<br>- Perform cross identification with the patient, handle or nursing staff.<br>• - Reconfirm full names and phone number identification card,<br>- Explain to the patient the procedure that is going to be performed carry out.<br>• - Use personal protection elements.<br>• - Locate the arm and venipuncture site. Remember not to take samples on the arm that has fluids, fistulas, wounds or lesions.<br>• - Place the tourniquet so that the pressure is not excessive, as this interferes with laboratory tests.<br>- Carry out asepsis of the venipuncture site with a cotton ball moistened with alcohol, clean in a circular manner from the inside out and wait for it to dry, DO NOT BLOW.<br>• As soon as the blood starts to flow, release the tourniquet, take the necessary tubes according to the order, take into account the order of collection and volume required per tube, mix gently by inversion the number of times indicated for each tube.		Laboratory assistant

Machine Translated by Google

	• Remove the needle from the vein , release the tourniquet, place a clean, dry cotton ball, apply gentle pressure with a straight arm and open hand. Place an adhesive tape to hold the cotton. If you are an anticoagulated patient, make sure you apply pressure for 5 minutes.<br><br>• Take into account biosafety standards for the disposal of needles, cotton and waste in general.<br><br>• Tell the patient how long their result will be.		
TAKING OF SAMPLES EARRINGS	In case of difficult veins, pediatric patients, a Head Nurse should be asked for help.		
IDENTIFICATION ON OF THE SAMPLES	Mark each tube in front of the patient with the initials of the patient's full name, leaving them visible so that the information can be verified with the corresponding stickers. Use a highlighter to indicate that it is a sample that should be given priority.	sticker	Laboratory assistant
TRANSPORT E DE SAMPLES TO THE LABORATORY EITHER	For safety, all samples should be handled as potentially infectious, even when the risk of infection is not evident.<br><br>Place the samples in the transport container: shipping capsule by pneumatic tube or manual sample transport, and send or take them to the central laboratory in the shortest possible time. Keep in mind that the tubes should not lie down during the journey.<br><br>To send urine or fecal matter samples through the pneumatic tube, remember to double package them in a ziplot bag and an airtight bottle to place them in the capsule.<br><br>Verify that all samples are delivered.<br><br>In the event of a spill, clean immediately with the appropriate disinfectant. (Ammonium	Samples taken	Laboratory assistant

Machine Translated by Google

	Quaternary)<br>See instructions/cleaning and disinfection.<br><br>When transportation is personal, deliver the samples to the laboratory assistant.		
RECORD INPUT AND DISTRIBUTION N OF SAMPLES	Once the samples are received, the assistant must record the samples that were received:<br>ORDERS – RECEIPT OF SAMPLES –<br>PASS THE BARCODE AND CONFIRM ENTRY.<br><br>**In order to prioritize emergency samples, highlight the order number with any color.**<br><br>Any sample other than blood (urine, fecal matter, etc.) that is taken in the emergency room must be recorded in the emergency sample delivery book with the requested data.<br><br>Distribute the samples in the different sections of the laboratory.	SAMPLE<br>Yes	ASSISTANT LABORATORY OR CENTRAL

16.4. TAKING URINE AND COPROLOGICAL SAMPLES EMERGENCY SERVICE

ACTIVITY	DESCRIPTION	RESPONSIBLE	RECORDS
Container delivery	The nursing assistant must give the patient the indicated container, duly marked with names and surnames – Identification and admission number and the gauze to carry out the previous cleaning.	Invoiced order Sticker	Laboratory assistant
Taking samples by spontaneous urination	The assistant must explain to the outpatient patient the location and manner indicated for collecting the sample.		Laboratory assistant
Taking patient samples with a probe	The head nurse is in charge of collecting urine samples by tube and must notify the laboratory assistant.		Head Nurse Laboratory assistant

Machine Translated by Google

16.5. NON-CONFORMING SAMPLES

The clinical laboratory, in compliance with the pre-analytical requirements that the samples must meet, considers as non-compliant samples: hemolyzed, hemodiluted, clotted, insufficient, incorrectly taken samples (inadequate tube or container, the filling volume of the tube is not taken into account). , misidentified both by name and by sample type.

In reference to non-compliant samples, the laboratory mainly carries out three actions:

Rejection: The sample is reserved in custody and a new sample is requested
Concession: in case of samples that are difficult to collect (CSF, neonates).
Reprocessing: a new sample is requested to confirm the report.

16.6. GENERAL LABORATORY EXAMS AND REQUIRED SAMPLES

EXAM	SAMPLE	EXAM	SAMPLE
ANTICOAGULANT LUPICO	BLUE TUBE	FOLIC ACID	YELLOW TUBE
AGGREGATION PLALETTARY	5 TUBES BLUE	VALPROIC ACID YELLOW	TUBE
BLOOD COUNT	LILAC TUBE	ANTI-CHAGAS	YELLOW TUBE
CRYAGLUTININS	TUBE YELLOW	ANTIDNA	YELLOW TUBE
D-DIMER	BLUE TUBE	ANTI NUCLEAR T UBE	YELLOW
LILAC TUBE ERYTHROSEDIMENT	ATION	ANTI TOXO G	YELLOW TUBE
eosinophils in mucus NASAL	MUCOUS SWEATER NASAL	ANTI TOXO M	YELLOW TUBE
FACTORS OF COAGULATION	BLUE TUBE	ANTI THROMBIN III BLUE	TUBE
FIBRINOGEN	BLUE TUBE	ANTIGENS EXTRACTABLES	YELLOW TUBE

Machine Translated by Google

BLOOD SMEARS PERIPHERAL	EXT.<br><br>PERIPHERAL BLOOD	ANTIGEN PROSTATIC	YELLOW TUBE
LILAC TUBE HEMOCLASSIFICATION		ASTOS	YELLOW TUBE
HEMOPARASITES	THICK DROP + FSP	BETA HCG	YELLOW TUBE
PDF	TUBE YELLOW	BNP	LILAC TUBE
SYC PROTEIN COAGULATION	BLUE TUBE	CARBAMAZEPINE YELLOW TUBE	
P.T.	BLUE TUBE	CARDIOLIPIN IG G YELLOW TUBE	
PTT	BLUE TUBE	CARDIOLIPIN IG M YELLOW TUBE	
PLATELETS	LILAC TUBE	C 3	YELLOW TUBE
RESISTANCE TO PROTEIN C	BLUE TUBE	C4	YELLOW TUBE
RETICULOCYTES	LILAC TUBE	CYCLOSPORINE LILAC TUBE	
LE CELLS	LILAC TUBE	CYTOMEGALOVIRU S IG G	YELLOW TUBE
AMYLASE	TUBE YELLOW	CYTOMEGALOVIRU S IG M	YELLOW TUBE
LACTIC ACID	GRAY TUBE	COOMBS DIRECT LILAC TUBE	
URIC ACID	TUBE YELLOW	COOMBS INDIRECT	LILAC TUBE
BILIRUBIN T/D	TUBE YELLOW	DIGOXIN	YELLOW TUBE
CALCIUM	TUBE YELLOW	DRUGS ABUSE	PARTIAL URINE
CHLORINE	TUBE YELLOW	ETHANOL	GRAY TUBE
CHOLESTEROLS T, HDL, LDL	TUBE YELLOW	PHENYTOIN	YELLOW TUBE
CPK	TUBE YELLOW	PHENOBARBITAL YELLOW TUBE	
CPK MB	TUBE	FERRITIN	YELLOW TUBE

Machine Translated by Google

	YELLOW		
CREATININE	TUBE YELLOW	HEPATITIS (ALL)	YELLOW TUBE
ACID PHOSPHATASE	TUBE YELLOW	HIV	YELLOW TUBE
MATCH	TUBE YELLOW	IMMUNOGLOBULIN AS G, M, A, E	YELLOW TUBE
GGTP	TUBE YELLOW	PROLACTIN	YELLOW TUBE
GLYCEMIA	TUBE YELLOW	RUBEOLA IG G - IG M	YELLOW TUBE
HEMOGLOBIN GLYCOSYLATED	LILAC TUBE	SEROAGLUTINATION ONES	YELLOW TUBE
FREE HEMOGLOBIN BLUE TUBE		T3 – T4 – T3 AND T4 FREE	YELLOW TUBE
IRON	TUBE YELLOW	RA TEST	YELLOW TUBE
LDH	TUBE YELLOW	TRANSFERRIN YELLOW TUBE	
MAGNESIUM	TUBE YELLOW	TSH	YELLOW TUBE
UREIC NITROGEN	TUBE YELLOW	VDRL	YELLOW TUBE
ULTRASENSIVE PCR TUBE	YELLOW	B12 VITAMIN	YELLOW TUBE
POTASSIUM	TUBE YELLOW	ACID VANILMANDELIC	URINE 24 H + 10 CC HCL
PROCALCITONIN	TUBE YELLOW	5 HYDROXY ACID URINE 24 H + 10	CC HCL
PROTEINS	TUBE YELLOW	METANEPHRINES - CATECHOLAMINES	URINE 24 H + 10 CC HCL
SODIUM	TUBE YELLOW	ACTH	YELLOW TUBE
TGO	TUBE YELLOW	ADENOSIN OF AMINASE	SERUM OR LIQUID
TGP	TUBE	ALDOLASE	YELLOW TUBE

Machine Translated by Google

	YELLOW		
TRIGLYCERIDES	TUBE YELLOW	PHOSPHOLIPIDS IG M	YELLOW TUBE
ALPHAFETOPROTEINS TUBE	YELLOW	FSH	YELLOW TUBE
ALDOSTERONE	TUBE YELLOW	FTA	YELLOW TUBE
AMMONIUM	LILAC TUBE	GASTRINE	YELLOW TUBE
TROPONIN	GREEN TUBE HAPTOGLOBIN YELLOW TUBE		
ANTITHYROIDS	TUBE YELLOW	HELICOBACTER PILORY	YELLOW TUBE
ANTI MICROSOMAL TUBE	YELLOW	HERPES IY II IG G, IG M	YELLOW TUBE
ANTITHYROGLOBULIN TUBE	YELLOW	17 HYDROXYPROGEST ERONA	YELLOW TUBE
ANTIG CARCINOEMBRYONIC	TUBE YELLOW	HOMOCYSTEINE YELLOW TUBE	
ANTIG CANCER 125	TUBE YELLOW	INSULIN	YELLOW TUBE
ANTIG CANCER 15.3	TUBE YELLOW	MICROALBUMINURI TO	PARTIAL URINE
ANTIG CANCER 19.9	TUBE YELLOW	MITOCHONDRIA	YELLOW TUBE
PROSTATIC ANTIG FREE	TUBE YELLOW	YELLOW TUBE SMOOTH MUSCLE	
IONIC CALCIUM	TUBE YELLOW	OSTEOCALCIN GREEN TUBE	
CAPACITY COMB. IRON YELLOW	TUBE	P 65 FOR CYTOMEGALOVIRU Yes	LILAC TUBE
CAROTENE	TUBE YELLOW	**PARATHORMONE Yellow tube**	
CATECHOLAMINES	TUBE YELLOW	PIRINLIKS	URINE 3 HOURS

Machine Translated by Google

CYTOPLASMATIC	TUBE YELLOW	PROGESTERONE YELLOW TUBE	
CHOLINESTERASE	TUBE YELLOW	PROTEIN BENCE JONES	PARTIAL URINE
CORTISOL	TUBE YELLOW	RUBEOLA IG G	YELLOW TUBE
DEHSO4	TUBE YELLOW	RUBEOLA IG M	YELLOW TUBE
DENGUE IG G	TUBE YELLOW	TESTOSTERONE FREE	YELLOW TUBE
DENGUE IG M	TUBE YELLOW	TESTOSTERONE TOTAL	YELLOW TUBE
ELECTROPH. OF PROTEINS	TUBE YELLOW	THYROGLOBULIN YELLOW TUBE	
EPSTEIN BARR IG G	TUBE YELLOW	CHICKENPOX GYM YELLOW TUBE	
EPSTEIN BARR IG M	TUBE YELLOW	**VIRUS SECRETION PANEL**	**NASOPHARYNX**
ESTRADIOL	TUBE YELLOW	SUDAN	URINE OR FECAL MATTER
PHOSPHOLIPIDS IG G	TUBE YELLOW	CALCULATION URINARY	CALCULATION
COPROLOGIC	SUBJECT FECAL	DEPURATION CREATININE	URINE OF 24 HOURS
COPROCULTURE	SUBJECT FECAL	CREATINURIA	URINE OF 24 HOURS
COPROSCOPIC	SUBJECT FECAL	URICOSURIA	URINE OF 24 HOURS
HAMBURGER URINE COUNT 3 HOURS PROTEINURIA			URINE OF 24 HOURS
PARTIAL URINE	PARTIAL URINE CALCIURIA		URINE OF 24 HOURS
PREGNANCY TEST TUBE	YELLOW	PHOSPHATURY	URINE OF 24 HOURS
HIDDEN BLOOD	SUBJECT FECAL	UROCULTURE	PARTIAL URINE

Machine Translated by Google

SARS-CO-2 ANTIBODIES	TUBE YELLOW	SARS-COV-2 RT-PCR	SWAB NASOPHARYNGEAL
SARR-CO-2 FILM ARRAY HIPPOPATED	NASOPHARYNX EITHER		

16.7 MANAGEMENT OF ADVERSE REACTIONS IN SAMPLING IN THE CLINICAL LABORATORY

During the extraction of blood volumes corresponding to laboratory samples to perform paraclinical examinations, adverse reactions may occur that must be identified and managed promptly and effectively by clinical laboratory personnel. If necessary, the medical staff of the emergency service is able to collaborate with the management of the patient.

Adverse reactions can be of two categories: vasovagal reaction or venipuncture reaction. Both entities are not mutually exclusive.

16.7.1 VASOVAGALES

They are those triggered by a nervous stimulus or those associated with cerebral hypoperfusion, generally secondary to a decrease in cardiac output.
They can be caused by pain, seeing blood at the time of donation, seeing others donate blood, individual or group excitement, or appearing inexplicably. Depending on its severity (mild, moderate or severe), the most common findings are nausea, paleness and sweating. Additionally weakness, vomiting, drowsiness, vertigo, blurred vision, paresthesia, headache, cyanosis, chills, hypothermia, tachypnea, decrease in mean arterial pressure, rigidity, tremor, dyspnea, loss of consciousness, tachycardia, convulsions, relaxation of sphincters, pain precordial and trauma due to fall secondary to loss of consciousness.

General handling:

In all vasovagal reactions, the following actions must be carried out:

Machine Translated by Google

Ø Reassure the patient, avoid anxiety and take him, if possible, to a place suitable for handle your case in isolation.

Ø If the reaction occurs during the extraction, remove the tourniquet and the needle from the patient's arm.

Ø Loosen clothing and place the patient in a comfortable position (with the head level lower than the rest of the body to facilitate cerebral perfusion: Trendelenburg position).

Ø Control blood pressure, radial pulse, heart and respiratory rate, as well as the patient's attitude.

Specific management:

The following specific actions for each reaction are applied after having applied the measures described above.

a) **Syncope:** Syncope is a temporary loss of consciousness that leaves no sequelae. It is manifested by heaviness, sweating, vertigo, paleness, depression of consciousness, coldness of the skin, hypotension and bradycardia. It occurs rarely (0.08% to 0.34% of donations) and in these cases it is due:

Ø Verify that the airway is patent.

Ø Place cold compresses on the patient's forehead or neck.

Ø Gently inhale alcohol or ammonia.

Ø In the presence of hypotension, have the patient ingest a small portion of salt to stimulate an increase in blood pressure; if these measures do not help, refer to the emergency department.

Ø If there is no improvement, refer to the nearest emergency department for treatment. respective.

b) **Hyperventilation:** It is a frequency of pulmonary ventilation greater than that metabolically necessary for the adequate exchange of respiratory gases. It is the result of an increased respiratory rate, an increase in resting tidal volume, or a combination of both factors and produces excessive entry of oxygen with expiration of carbon dioxide. Hypocapnia and respiratory alkalosis appear, chest pain, vertigo, fainting, numbness of the fingers and toes, and psychomotor alteration occur.

• The patient's attention should be distracted by talking to him, to avoid hyperventilation.

• Ask the donor to breathe deeply through the nose and with the mouth closed, keep the air in the chest and after a few seconds slowly expel it through the mouth. This form of breathing should be done for at least five minutes.

Machine Translated by Google

• If the symptoms persist, the donor should be made to breathe into a paper or plastic bag and asked to breathe in through the nose and expel it with the mouth open (do not administer oxygen).

c) **Seizures:** Abrupt, violent and involuntary series of contractions of a group of muscles, which can be paroxysmal and episodic as in convulsive diseases, or transient and acute, as in the case of those that occur secondary to a blood donation. . A seizure can be clonic or tonic, focal or generalized (unilateral or bilateral). In tonic seizures, rigid muscles produce immobility of the corresponding segment, in which slight trembling occurs. In clonic seizures, the muscles experience a series of alternating contractions and relaxations that cause sudden jerks of the segment.

Ø Prevent the patient from injuring himself or another person.

Ø Lay the patient on the stretcher or on the floor.

Ø Prevent the patient from biting his tongue.

Ø Lateralize the head to the right or left, to avoid aspiration (safety position).

Ø Avoid crowding of personnel as this reduces oxygenation and obstructs the passage of personnel who must take immediate measures.

Ø Transfer the patient quickly to the emergency department.

d) **Nausea or vomiting:** Emesis can be accompanied by hydro-electrolyte imbalance due to loss of sodium, chlorine and hydrogen ions, with the consequent metabolic alkalosis.

• Instruct the donor to breathe slowly and deeply.

• Because this reaction is part of the vasovagal reflex and is accompanied by hypotension, the patient should be placed in the Trendelenburg position.

• Place the donor's head in a right or left lateral decubitus position. to avoid bronchoaspiration in case of vomiting.

• Provide a plasticized aluminum bag for vomit and provide towels paper for the donor to dry his mouth.

• Offer the donor water to rinse his or her mouth.

• If symptoms persist and according to medical criteria, administer 10 mg. of metoclopramide intravenously.

Machine Translated by Google

14.7.2. ADVERSE REACTIONS FROM VENIPUNCTION

They are caused by injury to the vessels located at the antecubital level, whether vein or artery, and in some cases by nerve injury to the region. In certain cases it is due to inadequate venipuncture technique and contamination of the area in the area in question.

General handling:

• Remove the tourniquet

• Stop bleeding

• Remove the needle

• Instruct the patient to apply digital pressure for ten minutes • Elevate the affected limb

• Apply ice in five minute intervals

• Recommend the patient to use anti-inflammatories and warm water cloths.

• Explain to the patient that generally the injuries caused by venipuncture are very slow to recover.

Specific management:

a) **Hematoma:**

EITHER Remove the tourniquet and needle.

EITHER Place a sterile gauze or dressing over the venipuncture site and apply digital pressure for 7 to 10 minutes with the affected arm elevated. The dressing should allow frequent inspection of the puncture site.

EITHER Apply ice to the area for five minutes to promote healing. local vasoconstriction (be careful to cover the ice in a gauze or compress to avoid burns).

b) **Arterial puncture**

Ø If an arterial puncture is suspected, remove the needle immediately and apply firm pressure for 10 minutes.

Ø Apply a compression bandage immediately.

Ø Check radial pulse. If the pulse is not palpable or weak, inform the doctor or donation coordinator.

Ø Late complications may arise from arterial puncture:

ÿ Pseudoaneurysm

Machine Translated by Google

ÿ Arteriovenous fistula

ÿ Compartment syndrome

These complications do not have specific management by the staff of the blood bank where the donor care was performed. In these cases, the treatment for these injuries must be done in a hospital center where definitive surgical treatment must be considered.

c) **Dermatitis**

Ø The donor should be suggested to use moisturizing creams with Vaseline.

Ø If the above does not improve, the donor will be recommended to consult with his or her doctor to begin managing the lesion with corticosteroids.

d) **Cellulite**

Ø Since the most common agent is *Staphylococcus aureus,* the antibiotic choice is a dicloxacillin-type beta lactam.

e) **Neuropathic pain**

Ø This injury is due to a nerve injury and is chronic in nature. It is recommended that the management of this injury be multidisciplinary; Due to the characteristics of the pain, medications are required that provide more effective analgesia than that provided by commonly used analgesics such as acetaminophen.

Ø Due to the above, initial management by neurology, physiatry and physiotherapy.

f) **Causalgia**

Ø Because the involvement in this type of neuropathic pain is much more severe and compromises the musculoskeletal system, specialized management is required.

Ø As a first therapeutic approach, oral medications such as gabapentin, calcitonin, bisphosphonates, calcium antagonists, GABA agonists and corticosteroids are started.

Ø The application of parenteral treatments such as cycles of local nervous anesthetics, intravenously or local anesthetic nerve blocks such as injections in the stellate ganglion or paralumbar sympathetic chain, spinal cord stimulation and intrathecal infiltrations, among others, is suggested.

g) Phlebitis

It can occur 12 to 48 hours after phlebotomy, it manifests itself with pain, redness and heat over the puncture site or along the course of the vein.

ØPerform asepsis of the puncture site, keeping hands and gloves clean.

ØUse strictly sterile equipment that must be uncovered at the time of phlebotomy.

ØDo not touch the puncture site after asepsis has been performed and covering the puncture site with a sterile band-aid.

Machine Translated by Google

ØDo not puncture near a skin lesion.

ØCarry out an assessment by the medical staff.

Ø According to the phlebitis scale, referenced in document OD-4.3.2-01, have it reviewed by medical personnel.

16.8 PRIOR PREPARATION FOR THE PATIENT

Exam	Preparation
Antithrombin III<br>Hemoglobin electrophoresis<br>Protein electrophoresis<br>Fibrinogen<br>Glycemia<br>Glucose-6-Phosphate<br>Globulins<br>Insulin<br>Total proteins<br>PT INR<br>PTT<br>Serology (RPR)<br>Thyroglobulin	**MAXIMUM FASTING 12 HOURS**
Lipid profile (total cholesterol, 14-hour	fast, do not consume HDL, LDL, triglycerides) alcoholic beverages 72 hours before, do not consume animal fats for 48 hours and in the 5 days prior to taking the drink you should follow your usual daily diet.
Glycemic and/or insulin curve	Fasting from 8 to 12 hours. Allow 3 ½ hours to complete the exam.<br>Do not smoke cigarettes during the test, do not expose yourself to stress or do any type of physical activity and stop 3 days before the test.
Glycemia and/or insulin pre and post	fasting of 8 – 12 hours. Have at least 2 ½ hours to complete the exam.
Thyroid profile	Do not take your thyroid medication before taking the sample, indicate what drug you take and in what dose.
Folic acid (Folate)	Fast for 12 hours, avoid alcoholic beverages, aminosalicylic acid, ampicillin, antimalarials, chloramphenicol, ampicillin, estrogens, methotrexane, penicillin, one week before taking.
ACTH (Hormone	Avoid being in stressful situations prior to taking

Machine Translated by Google

Adenocorticotropic)	of the sample.
Aldosterone	Maintain a normal sodium diet; the patient should not be exposed to radioactivity 4 hours before the test.
Prostate antigen (free PSA / It cannot be performed after the total physical examination) prostate (rectal examination). Verify that at least a week has passed. Sexual abstinence for 3 days.	
Alpha 1 Antitrypsin	It does not require fasting, avoiding stressful situations and exercise.
Uric acid<br>Alpha Feto Protein<br>Amylase<br>Hepatitis B Surface Antigen (HbsAg)<br><br>Antibody against hepatitis B surface antigen (aHBs)<br><br>Anti Chagas Antibodies<br>Anti-hepatitis B core antibodies (aHBC)<br>Anti Epstein Barr Antibodies (igM-IgG)<br>Anti Smooth Muscle Antibodies (ASTHMA)<br>Antibodies Antinuclear (ANAS)<br>Anti cytomegalovirus antibodies (IgM-IgG)<br>Anti Hepatitis A Antibodies (IgM-IgG)<br>Herpes Simplex I-II Antibodies (IgM-IgG)<br>Anti DNA Antibodies<br>Microsomal Antibodies<br>Antimitochondrial Antibodies (AMA)<br>Rubella antibodies (IgM-IgG)<br>Anti-HIV Antibodies<br>Total Anti Hepatitis Antibodies	**DOES NOT REQUIRE FASTING**

Machine Translated by Google

c<br>Thyroglobulin Antibodies<br>Thyroid Antibodies<br>Anti Toxoplasma Antibodies (IgM-IgG)<br>Antistreptolysin O (ASTOS)<br>Bacterial Antigens<br>Antigens Nuclear Extractables (ENAS)<br>Antigen Carcinoembryonic (ACE)<br>CA 15-3 Antigen<br>C-ANCA, P-ANCA Antibodies<br>Bilirubin (Total, direct, indirect)<br>Serum Calcium<br>Ionic Calcium<br>blood chlorine<br>Creatinine<br>Creatine Kinase (CK)<br>Creatine Kinase (CKMB)<br>cholinesterase<br>Blood count<br>C3 and C4<br>Dengue IgG – IgM – Antigen NS1<br>Lactic Dehydrogenase (LDH)<br>Gamma Glutamyl Transferase (GGT)<br>Iron (Fe)<br>Growth Hormone (GH)<br>Hemoclassification<br>Glycosylated hemoglobin<br>Immunoglobulins (IgA, IgD, IgE, IgG, IgM)<br>Magnesium<br>Urea Nitrogen (BUN)<br>Lead<br>Potassium<br>C Reactive Protein<br>Platelet count<br>Sodium<br>Somatomedin C	

Machine Translated by Google

Transaminases (TGO - TGP)<br>AR test<br>Free and total testosterone<br>Transferrin<br>Troponin I	
Ammonium	It does not require fasting, do not smoke 12 hours prior to taking the sample.
Allergens (IgE Antibodies)	Suppress the administration of isotopes for 24 hours.<br>Specify type of allergen to request, prior fasting.
Androsterone	Stop taking ACTH, gonadotropin or thyroid medications 48 hours before taking.
CA 125 Antigen	It does not require fasting, do not take during the menstrual period.
Apoliproteins A1 and B	Requires prior fasting of 14 hours, with the usual eating regimen.
Cervical Vaginal Cytology	• Do not have intercourse 3 days prior to the realization.<br>• Do it 5 days before or 5 days after the menstrual period.<br>• Not having used treatment with ovules 3 days prior to taking.
Stool culture, Clostridium difficile Diarrheal fecal matter.	
Coprological<br>Coproscopic	Fecal matter, two hours maximum for collection
serum cortisol	According to medical request, one or two doses are taken.<br>Arrive 20 minutes early for administrative procedures.<br>For a single dose: it must be taken at 8 am.<br>Two feedings: first feeding 8 am<br>second shot 4pm
Dehydroepiandrosterone Sulfate Avoid vigorous exercise for 48 hours (DHEAS)	
Therapeutic drug control Prior fasting, inform date of last intake (digoxin, carbamazepine, sample taken 12 hours after last phenytoin, phenobarbital, cyclosporine, tracolimus intake)	

Machine Translated by Google

Urine electrophoresis<br>Urine osmolarity	Urine sample
Creatinine clearance,<br>nitrogen, calcium, sodium, potassium,<br>chloride, cortisol in 24-hour urine,	Collect a 24-hour urine sample of the following proteinuria, urea manner:<br>ü Request a clean, dry bottle from the laboratory or purchase it. ü Start the urine collection<br>with the second urine of the morning, collect all the urine emitted during the course of the day and night and the first of the day that ends.<br>ü Once the collection is finished, take it to the laboratory as soon as possible, **avoid losing the volume of urine.**
Homovanillic acid, vanilmandelic acid acid, hydroxyindoleacetic acid, catecholamines,<br>metanephrines, porphobilinogen, oxalates in urine.	Collect urine for 24 hours out of the next 5 manner:<br>ü Claim a dark flask in the laboratory.<br>ü Start urine collection with the second urine in the morning, collect all the urine emitted during the course of the day, the night and the first urine of the day that ends.<br>ü Once the urine collection is finished, take it to the laboratory as soon as possible, **avoid losing the volume of urine.**<br>**Remember:** do not consume coffee, tea, bananas, vanilla and check with your doctor that the medications you take do not interfere with the exam.
Follicle Stimulating Hormone (FSH)<br>Luteinizing Hormone (LH)<br>Pregnancy test<br>Quantitative Beta HCG<br>Estradiol<br>Progesterone	Does not require fasting. Please indicate the date of the last menstruation.
CD3 – CD4 lymphocytes	Monday to Thursday from 6:30 am to 8:30 am, with a prior fast of 10 hours.
Lithium	Indicate the dose and the last time of the last dose.
Microalbuminuria	First urine of the morning.
Partial urine	Collect the first urine of the morning immediately after bathing, discarding the first stream. If the sample is collected in the laboratory, it must

Machine Translated by Google

	have a minimum of 3 hours of retention.
Pirilink count hamburguer,	Collect a 3-hour urine sample in a clean, dry bottle. Preferably it includes the 9 am
BK culture in urine	Collect the first urine of the morning, complete, in a clean, dry bottle.
Urine culture	Collect the first urine of the morning immediately after bathing, discarding the first stream. If the sample is collected in the laboratory, it must be retained for at least 4 hours and must be cleaned beforehand.
Hidden blood	There should be no evidence of dental bleeding, do not collect the sample if you are having your period.
Prolactin	It should be taken at least 2 hours after waking up.
BK staining and/or culture	Claim the indicated vial (red lid) at the laboratory, collect the sample and deliver it to the laboratory as soon as possible.
Platelet aggregation, HLA, Previous B27. Sampling by telephone (1) 271 probe.	fasting. Make an appointment from Monday to Thursday at 43 31.

14.0. BIBLIOGRAPHY

Ø Budassi S. Laboratory Specimens. In: Emergency Nursing. Principles and Practice. Mosby Yearbook. St.Louis, 1992.

Ø Campuzano-Maya G. The blood count. Medicine & Laboratory. 1998; 8:19-32. Cuervo P, Rico C. Guide for taking blood cultures. Current Sick 2001; 4:33-36. Ø From Pedro J, Llobera J, Bennassar M, et al. Efficacy of two compression methods in the appearance of hematomas after blood extractions. Clinical Nursing 2002; 12:1-5. De Merino N. Procedures Manual. Department of Pathology and Clinical Laboratory. Third edition. Santa Fe de Bogotá Foundation. Bogotá, 2001. Ø Tucker S, Canobbio M. Basic nursing diagnoses, special needs and equipment. In: Standards of Patient Care. Harcourt Oceano. Barcelona, 2002.

Ø Wilson S, Thompson J. Respiratory disorders. Doyma and Times Mirror Editions.

Machine Translated by Google

Barcelona, 1993.

- Ø Castro Rivera Sandra, Enf. Valle del Lili Foundation Clinic.
- Ø Jacques Wallach. Clinical interpretation of diagnostic tests. 8th edition. Lippincott Williams & Wilkins. Spain 2008.
- Ø Manual for taking samples for microbiological analysis, district health department-cic health foundation. Bogotá 2015.
- Ø Laboratory Dictionary Applied to the Clinic, 3rd edition, Angel Mejia Gilberto, 2004.
- Ø Clinical Interpretation of the Laboratory, 8th edition, Gómez Gutierrez – Casas Gomez, 2014.
- Ø Standard ISO 15189:2012 “Clinical Laboratories – Quality requirements and competence"
- Ø ISO/TS 20658:2017 “Medical laboratories – Requirements for the collection, transport, reception and handling of samples
- Ø Simundic et al.: Joint EFLM-COLABIOCLI Recommendation for venous blood sampling – v1.1, June 2018.

SPANISH VERSION (ORIGINAL)

VERSIÓN	FECHA	DESCRIPCIÓN DE LA MODIFICACIÓN
7	24/02/2022	Se incluye la flebitis dentro de las complicaciones por venopunción.
6	29/05/2020	Se incluye la marcación de los tubos de muestras sanguíneas con las iniciales del nombre completo del paciente, dejándolas visibles para poder verificar los datos del sticker generado por el sistema del laboratorio..<br>Se adiciona proceso para toma de muestras cuando es necesario extraer sangre de la misma extremidad utilizada para la administración intravenosa de medicamentos o líquidos.<br>Se adiciona información sobre posición del paciente y uso adecuado del torniquete durante la toma de muestras venosas.<br>Se adiciona información sobre el uso de tubos microtainer.<br>Se adiciono información sobre la toma de Glucometrias en el *sistema Accu-Chek* Inform II<br>Se actualizan valores de referencia.<br>Se actualiza protocolo de toma de muestras de microbiologia.<br>Se incluye numeral de Bioseguridad en toma de muestras sanguíneas.<br>Se incluye Covid-19 en el numeral 15.0 Bases de interpretación de los resultados de las muestras el laboratorio y en el numeral 16.6 Exámenes de laboratorio general y muestras requeridas.
5	1 MAY 2016	Se modifica la toma de hemocultivos por el cambio de botellas por el cambio de tecnología.
4	14/07/15	Actualización de actividades, toma de muestras de microbiología.
3	10-mar-11	Actualización de actividades, se incluye tabla de tiempo de reporte y condiciones de preparación del paciente
2	10/03/10	Actualización de actividades relacionadas a toma de muestras en consulta externa

**Elaboro:** Isabel Cristina Torres	**Actualizó:** Paula Andrea Ramos	**Revisó:** Isabel Cristina Torres	**Aprobo:** Gilberto Mejia Estrada, MD.
**Cargo:** Coordinadora Laboratorio Clínico	**Cargo:** Bacteriologa	**Cargo:** Directora laboratorio clínico	**Cargo:** Director científico.
**Fecha:** 14 dic 2009	**Fecha:** Febrero 2022	**Fecha**: Febrero 2022	**Fecha:** Febrero 2022

1.0 OBJETIVO GENERAL

Realizar de manera eficaz y oportuna la toma de muestras en los servicios de Urgencias,hospitalización y consulta externa cumpliendo los requisitos y pautas establecidas para tal fin en el departamento de laboratorio de la Fundación Clinica Shaio asegurando la calidad de la muestra.

2.0 ALCANCE

Este procedimiento aplica desde el momento en que se genera la solicitud de exámenes en el sistema de información de historia clínica, hasta la entrega de las muestras en el laboratorio central , sean éstas tomadas en piso por el personal del laboratorio (bacteriólogas y/o auxiliares) en las rondas establecidas, o por las enfermeras a cargo del paciente.

3.0 DEFINICIONES

ASEPSIA: Conjunto de procedimientos que disminuyen considerablemente la proliferación y propagación de microorganismos patógenos.

ESTANDARIZACIÓN: Desarrollo y la implementación de manera uniforme y acordada, de especificaciones técnicas, criterios, métodos, procesos y prácticas que pueden incrementar calidad, reproducibilidad y seguridad asistencial.

HEMOLISIS: Fenómeno de la desintegración de los eritrocitos.

LABORATORIO CENTRAL: Laboratorio clínico encargado del procesamiento de todos los exámenes de laboratorio de la Fundación Clinica Shaio.

ORDEN: Solicitud de exámenes de laboratorio diligenciada por el médico tratante en cada piso y generada directamente en el sistema de información.

VENOPUNCIÓN: La venopunción es la extracción de sangre de una vena, realizada por el personal de salud. También se conoce con el nombre de punción venosa.

4.0 RESPONSABLES

Medico tratante
Enfermera Jefe
Auxiliar de facturación.
Auxiliar de laboratorio
Bacterióloga

5.0 DESCRIPCIÓN

El laboratorio clínico está a cargo de la toma de muestras en el servicio de hospitalización (excepto UCI pediátrica y rotonda de UCI adultos), para lo cual ha organizado la toma de muestras por rondas en los siguientes horarios:

05:00 H – 7:00 H
12:00 H
18:00 H
24:00 H

Para que una orden sea incluida en la ronda, debe estar cargada en el sistema de Historia Clínica por lo menos 1 **hora antes** de la ronda, de lo contrario quedará para la ronda siguiente.

Las muestras de órdenes generadas como urgentes en horarios diferentes a las rondas deben ser tomadas y enviadas por las enfermeras en el piso.

La toma de muestras de Hemocultivos no se rige por las rondas, sino a solicitud del médico tratante.

6.0. OBJETIVOS ESPECÍFICOS:

Concientizar al personal de la importancia de una adecuada toma, manipulación, transporte y conservación de muestras, para:

- Garantizar un procedimiento seguro y centrado en el paciente.
- Estandarizar el proceso de toma de muestras.
- Prevenir errores en la toma de muestras de manera que las decisiones clínicas basadas en los resultados de los exámenes de laboratorio sean correctas

7.0. RECOMENDACIONES GENERALES

7.1. PROCEDIMIENTO PARA LA TOMA DE MUESTRAS

- ✓ Verificar rigurosamente con la orden médica el nombre del paciente y los exámenes a tomar.
- ✓ Verificar el tipo de tubos a utilizar antes de tomar la muestra, es importante confirmar con la orden médica y el examen el tipo de tubo, cantidad de la muestra (pacientes pediátricos) y condiciones específicas de manejo de las muestras.
- ✓ Presentarse ante el paciente con nombre, cargo y explicar su papel en la atención .
- ✓ Corroborar la Identificación del paciente, realizando verificación cruzada, explicarle el procedimiento y la preparación si se requiere.
- ✓ Interrogar al paciente sobre antecedentes de alergias e ingesta de medicamentos, especialmente, anticoagulantes.
- ✓ Confirmar que el paciente cumple con las condiciones previas de preparación necesarias para la toma de muestra según el examen solicitado.
- ✓ Las manos deben estar limpias para minimizar el riesgo de transmitir infecciones durante la extracción, siempre se debe usar un nuevo par de guantes para proteger al paciente y al personal que toma la muestra.
- ✓ Preservar la técnica aséptica en la obtención de muestras mediante procedimientos invasivos (venopunción periférica, catéter central, punción lumbar, etc.)
- ✓ El paciente debe estar en una posición cómoda.
- ✓ Si presenta dificultad en la toma solicite ayuda de otra bacterióloga, de enfermera o de auxiliar de enfermería.
- ✓ Rotular los tubos o recipientes con los datos del paciente, frente a él corroborando el nombre y número de identificación .
- ✓ Para una identificación adecuada, se deben usar al menos dos identificadores (Nombre completo y numero de identificación, en la Fundación Clínica Shaio se utiliza el numero de ingreso) , la identidad del paciente debe compararse con los datos del sticker.
- ✓ Los tubos de muestras sanguíneas una vez tomados y en presencia del paciente deben marcarse con las iniciales del nombre completo del paciente (escritas con marcador en el etiqueta que trae el tubo) y estas deben quedar visibles para poder verificar la información del código de barras generado por el sistema del laboratorio. Esto nos permite garantizar la trazabilidad de la identidad del paciente
- ✓ Enviar o llevar la muestra al laboratorio en el menor tiempo posible.
- ✓ Llenar los tubos al vacío hasta el nivel marcado; es imprescindible que estén llenos justo hasta la señal.
- ✓ No extraer sangre de la misma extremidad utilizada para la administración intravenosa de medicamentos, líquidos o transfusiones. Si no existe otro sitio disponible, asegúrese que la punción venosa se localiza por debajo del catéter .

  Si la condición del paciente lo permite y bajo autorización médica, el profesional de enfermería cierra el flujo por 10 minutos, se toma un tubo tapa amarilla de descarte y luego se tome el tubo necesario para el examen solicitado, en el sticker haga la anotación con el fin de realizar una correcta correlación en el momento de validar

el resultado.

- ✓ No extraiga sangre de venas endurecidas, derivaciones arteriovenosas, lugares de hematoma, inflamación o hinchazón, de un brazo con injerto vascular, brazos paréticos o brazos con transtornos de drenaje linfático.

  La punción venosa puede llegar a ser causa de infección, alteraciones circulatorias o retraso en la cicatrización, por aplicación inadecuada de la técnica.

  En la Fundacion Clínica Shaio los pacientes identificados con sticker azul tiene restricción para la toma de la muestra por tener zonas edematizadas, paralizadas, con mastectomía o fistula artero venosa y áreas con infección o lesiones cutáneas.
- ✓ En caso de que se tome la muestra de una vía ya instalada en el paciente (Catéter), es fundamental realizar una purga de mínimo 20 cc con el fin de evitar contaminación química.
- ✓ Utilizar los guardianes para descartar las agujas y utilizar las canecas de acuerdo a la segregación de desechos en la toma de muestras.
- ✓ Recordar que un diagnóstico o la evolución de un paciente dependen de un buen procedimiento, del compromiso del que toma la muestra, la procesa, valida y reporta.

7. 2. PROCEDIMIENTO PARA LA TOMA DE MUESTRAS EN NIÑOS

- En pacientes ambulatorios verifique que los padres o acudientes hayan firmado el consentimiento para la toma de la muestra.
- Seguir correctamente las indicaciones propuestas anteriormente.
- Realizar el procedimiento solicitando la ayuda de otro profesional y/o un familiar del menor.
- Sujetar al paciente firmemente sin lesionarlo.

7.3. BIOSEGURIDAD EN LA TOMA DE MUESTRAS

La manipulación inapropiada de las muestras puede convertirse en una fuente de riesgo biológico para las personas que están en contacto o para el medio ambiente. Todas las muestras de especímenes biológicos deben considerarse potencialmente infecciosas por lo que se debe seguir rigurosamente el protocolo de Bioseguridad establecido por la institución.

Utilizar los elementos de protección personal según las normas de bioseguridad del laboratorio clínico y del comité de infecciones de la institución, necesarios para evitar exposición con riesgo biológico de acuerdo con la fuente de la muestra: protección ocular (gafas o mascarilla facial), guantes, bata.

Cumplir con las recomendaciones de manejo de elementos cortopunzantes: No reenfundar agujas, disponer y utilizar adecuadamente el contenedor para cortopunzantes, no transportar jeringas con agujas.

En caso de accidente con riesgo biológico, avisar inmediatamente según las recomendaciones del protocolo de accidente de trabajo con riesgo biológico institucional.

No deben ingerirse comidas, bebidas durante los procedimientos de toma de muestras ni en los sitios dispuestos para tal fin.

7.4. PROCEDIMIENTO EN CASO DE ACCIDENTE DE TRABAJO POR PUNCIÓN

- Retirar los guantes
- Presionar constantemente el área de la herida permitiendo que la misma produzca sangrado
- Durante diez minutos lavar muy bien la herida con agua y jabón.
- Seguir los lineamientos para reporte a la ARL de acuerdo a los protocolos institucionales; avisar al director del área y al encargado de salud ocupacional de la institución, dirigirse a urgencias, tomar las muestras requeridas e iniciar profilaxis post exposición si esta indicada.

Los documentos guía relacionados son los siguientes:
Programa PG-10.1-07 Seguimiento accidentes de Trabajo.
Instructivo IN-10.1-02 Atención accidentes trabajo.
Instructivo IN-10.1-03 Instructivo de manejo , investigación y seguimiento de accidentes de trabajo.

8.0. PREPARACIÓN DEL PACIENTE

- La preparación del paciente, la toma y el manejo adecuados de las muestras garantizan resultados de óptima calidad y a la vez proporcionan resultados válidos.
- Los factores relacionados con el paciente que pueden afectar los resultados se dividen en:
  - ✓ Intrínsecos o inmodificables: Edad, sexo, raza, embarazo, ciclos biológicos.
  - ✓ Extrínsecos o Controlables: Dieta , ejercicio, hábitos variables (fumar, alcohol, cafeína), toma de muestra (material, torniquete), transporte y conservación.
- La tensión mental o física puede afectar los niveles de muchos constituyentes de los líquidos corporales, por lo tanto el paciente debe estar y sentirse cómodo y confortable
- La ansiedad y la tensión son estimulantes de concentración plasmática de la somatotropina, prolactina, cortisol, catecolaminas, aldosterona y renina.
- Períodos de larga tensión afectan pruebas metabólicas como la glucosa, las proteínas plasmáticas y algunos factores de coagulación.
- El ejercicio y el trabajo muscular vigoroso afectan la CPK, LDH, K, Glucosa, lactato, creatinina y algunos factores de la coagulación.
- El ejercicio estimula la secreción y producción de hormonas como la hormona de

crecimiento, prolactina, cortisol y renina.

- La muestra de sangre se debe extraer en la mañana en ayunas, 8 a12 horas después de la ultima comida. Se permite el consumo de agua en pequeñas cantidades durante el periodo de ayuno. La insulina, calcitonina, fosfatos, triglicéridos, fosfatasa alcalina se alteran después de la ingestas de alimentos.
- Dar instrucciones precisas y claras al paciente sobre las condiciones óptimas de preparación para la toma de algunas pruebas. (ver anexo preparación del paciente)
- Verificar si es el caso, que el paciente haya seguido rigurosamente las instrucciones antes de tomar la muestra.
- La ingesta de etanol puede alterar las muestras de enzimas hepáticas, glucosa, triglicéridos, uratos, lactato y protrombina.
- Registrar o tener en cuenta los medicamentos que está tomando el paciente para realizar un adecuado análisis y validación de los resultados. Se debe evitar la medicina en la mañana a menos que sea vital para el paciente.

8.1. TÉCNICAS DE RECOLECCIÓN DE MUESTRAS

8.1.1. MUESTRAS VENOSAS

La extracción de la sangre venosa debe realizarse en un entorno limpio, tranquilo y privado. Debe tranquilizarse al paciente ya que el estrés provocado por la flebotomía (punción) puede afectar los resultados del laboratorio como cambios en la concentración de catecoláminas y gases en sangre.

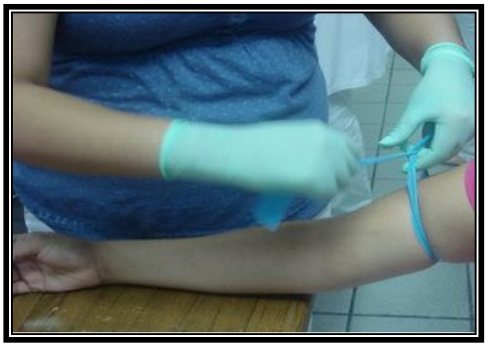

Colocar el torniquete

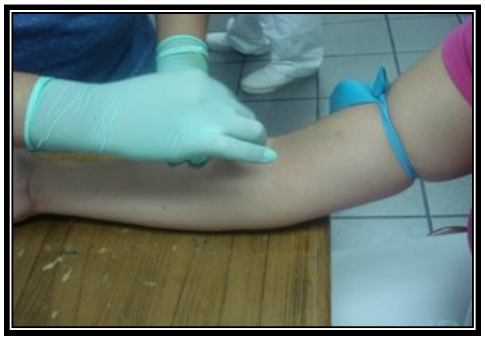

Asepsia del área

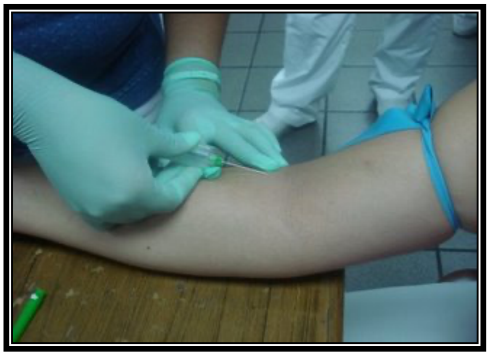

Realizar la punción

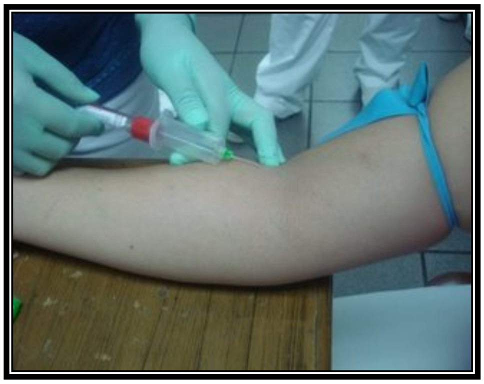

Tomar la muestra

Para seleccionar el sitio de venopunción, el brazo del paciente debe estirarse hacia abajo. Si están disponibles, las venas mas prominentes en la foca cubital deben ser la primera opción. Solo si las venas principales no están disponibles, las venas dorsales de la mano se pueden usar como alternativa. También se pueden obtener de un acceso venoso central instaurado en el paciente.
Se recomienda tomar la muestra, de una vena independiente de la vena periférica que se esté utilizando para terapia intravenosa, mediante técnica cerrada de tubos al vacío, lo cual mejora su calidad (cantidad apropiada, disminución de hemólisis) y disminuye el riesgo de contaminación.

8.1.2. POSICIÓN ADECUADA DEL PACIENTE:

Asegúrese que el paciente se ubique en una posición segura y cómoda.
Lo ideal es que el paciente no cambie su posición dentro de los 15 minutos previos al muestreo de sangre. Si el paciente esta acostado, la toma de la muestra de la sangre se debe hacer en posición acostada, (pacientes hospitalizados). Lo pacientes ambulatorios, idealmente, deben descansar en una posición sentada durante 15 minutos antes del muestreo de sangre. Las modificaciones posicionales afecta los resultados de: Albúmina, proteínas, diversas enzimas, calcio, bilirrubina, colesterol, triglicéridos, angiotensina, aldosterona y renina.

Acostado: Existe un acomodo o distribución hemodinámica y de otros líquidos corporales

Sentado: Empieza la salida de liquido intravascular al espacio intersticial y, por lo tanto, se produce hemoconcentración

8.1.3. TORNIQUETE:

La extracción de sangre se debe realizar preferiblemente sin torniquete (especialmente en pacientes con venas prominentes) y este se debe usar solo cuando sea necesario (pacientes con venas pequeñas o apenas visibles).
El torniquete debe aplicarse aproximadamente a un ancho de mano (7,5 cm) por encima del sitio de punción previsto y debe ser lo suficientemente apretado como para detener el flujo sanguíneo venoso, pero no el arterial, muy alto no ejerce presión y muy bajo aumenta la posibilidad de hematoma.
Su uso no se debe prolongar por mas de 1 minuto, puede producir éxtasis venoso localizado, la muestra se hace hemoconcentrada, induciendo valores erróneamente altos.
Se pide al paciente que cierre el puño, lo cual distiende las venas, el ejercicio excesivo del puño debe evitarse, dado que puede producir elevación en la concentración de potasio y LDH.
Las muestras para determinar Lactato y potasio deben ser tomadas sin torniquete.

8.1.4. SELECCIÓN DEL SITIO DE PUNCIÓN

- Nunca practique una punción sanguínea en un paciente que se encuentre de pie (La posición de pie es inestable y en caso que el paciente se desmaye, será más difícil evitar que se lesione).
- No elija una extremidad en donde esté colocada algún tipo de venoclisis.
- Inspeccione la vena que se va a puncionar.
- Coloque el torniquete con el nivel de tensión necesaria.
- Si la vena no es muy visible ni palpable, realice un suave masaje en el antebrazo con movimientos desde la muñeca hacia el codo.
- Observe siempre las dos extremidades superiores (brazos), para elegir el mejor sitio de punción.
- Recuerde que: Los hematomas se previenen con una técnica adecuada evitando que la aguja atraviese la vena, liberando el torniquete antes de extraer la aguja, aplicando suficiente presión sobre el sitio de la punción.
- Al finalizar el procedimiento, indíquele al paciente que debe hacer presión en el sitio punzado por lo menos durante cinco (5) minutos. Coloque finalmente una banda adhesiva sobre el sitio de la punción.
- Si el sangrado no se detiene, aplique presión constante sobre la punción durante 5 minutos más. Si el problema aún no se soluciona, avise al personal de enfermería encargado.
- Si el paciente se marea o tiende al desmayo, haga que se acueste y que respire profundamente, controle signos vitales e informe de inmediato al médico tratante y/o al personal de enfermería.
- Deje cómodo al paciente.

- Realice la correcta disposición de los residuos generados del procedimiento.

Asegúrese de rotular con identificación completa los tubos de las muestras, en presencia del paciente.

Complicaciones derivadas de una extracción venosa: Hematoma, Sangrado, Trauma extravasación, etc. Estas se deben dejar registradas en el Libro reporte de incidentes en venopunción RE-4.5.4-78

8.1.5 RECOMENDACIONES ESPECIALES EN LA TOMA DE MUESTRAS

Si la sangre no fluye en el interior del tubo o decrece el flujo antes de recolectar la muestra adecuada realice los siguientes pasos:

- Confirme la posición de la aguja en la vena
- La abertura de la aguja puede estar contra la pared interna de la vena, gire lentamente el soporte de la aguja y la sangre debe empezar a fluir.
- Si la aguja ha pasado a través de la vena, jale ligeramente la guía
- Confirmar la posición correcta del tubo en la guía
- Puede haber pérdida de vacío por perforación prematura o por abertura del tubo. Debe cambiar el tubo.

Asegurarse de tomar la muestra en el tubo correcto y en el siguiente orden si se realiza por sistema cerrado:

Orden de toma para recolección de sangre venosa

Tapón	Contenido de tubo	Área de uso	Inversiones
	Hemocultivo	Microbiología	5 veces
	Citrato de sodio	Coagulación (Tiempos de coagulación fibrinógeno, y agregación plaquetaria)	3 a 4 veces
	Gel separador	Química clínica	5 veces
	Sin anticoagulante, con activador de coagulación, con silicón	Química clínica, banco de sangre serología	8 a 10 veces
	Gel separador y trombina	Obtención de suero rápido	5 a 6 veces
	Gel separador y heparina de litio	Química clínica en plasma	5 veces
	Heparina de sodio/litio	Química clínica (urgencias) hematología (fragalidad osmótica)	8 a 10 veces
	$EDTAK_2$	Hematología, banco de sangre	8 a 10 veces
	Gel separador y $EDTAK_2$	Determinaciones de carga viral	8 a 10 veces
	Oxalato de Potasio/NaF	Química clínica, pruebas de lactato y glucosa	8 veces

Fuente: *Clinical and Laboratory Standards Institute (CLSI) – H3-A6 - Procedures for the Collection of Diagnostic Blood Specimens by Venipuncture; Approved Standard- Sixth Edition*, recomendaciones para tubos de plástico.

1- En tubo **azu**l: PT, PTT, fibrinógeno, , antitrombina **I**II, anticoagulante lúpico, Dimero D, Proteína C y S de coagulación y agregación plaquetaria (5 tubos, se deben enviar inmediatamente al laboratorio de referencia). Invertir para garantizar anticoagulación. Mezclar por inversiónde 3 a 4 veces. Este tubo es muy sensible a la relación sangre/anticoagulante por lo que se debe tomar hasta la marca indicada.

2- En tubo **amarillo y/o rojo:** química sanguínea, serología, infecciosas, inmunologia,serologia, productos de degradación de fibrinógeno. El tubo apa amarilla se debe mezclar por inversión 5 veces y el tapa roja de 8 a 10 veces.

3- En tubo **verde:** Pruebas de inmunogenética, Troponina I. Mezclar por inversión de 8 a 10 veces.

4- En tubo **lila:** Cuadro hemático, , eritrosedimentación, plaquetas, hemoclasificación, células LE, Coombs directo e indirecto y hemoparásitos (FSP) Invertir el tubo para homogenizar la muestra con el anticoagulante de 8 a 10 veces.

5- En tubo **gris:** Pruebas de ácido láctico y etanol. Mezclar por inversión de 8 veces.

Al tomar las muestras por sistema cerrado se reducen riesgos, se reducen errores y se mejoran los procesos. En caso de ser necesario tomar muestras por goteo, por ejemplo al canalizar al paciente en urgencias y reanimación, se sugiere que el personal que toma las muestras este acompañado de otro profesional o auxiliar que garantice el nivel adecuado de llenado, el correcto mezclado de muestras por inversión y la correcta identificación.

Los tubos BD Microtainer están diseñados para recolección, transporte y procesamiento de muestras obtenidas por punción capilar o venosa proveniente de pacientes pediátricos,

geriátricos, de urgencias, venas frágiles, o de cualquier condición que requiera volúmenes pequeños de muestras por ser de difícil acceso.

- ✓ Cada tubo tiene marca de volumen de llenado para asegurar la correcta proporción de volumen de muestra y anticoagulante.
- ✓ Mantiene el código de colores del tapón de acuerdo a la normatividad internacional.
- ✓ Se debe tener en cuenta que el orden, el volumen final y la mezcla durante el proceso de toma es muy importante para evitar la formación de coágulos en los tubos tapa lila.

Tapón	Contenido de tubo	Área de uso	Inversiones
Gases en sangre	Jeringas o capilares		
Muestras para microscopio			
	EDTA K_3	Hematología, banco de sangre	20 veces
	Heparina de sodio/litio	Química clínica (urgencias) hematología (fragilidad osmótica)	10 veces
	Heparina de litio y gel	Química clínica en plasma	10 veces
	NaFl / EDTA Na_2	Química clínica, pruebas de lactato y glucosa	10 veces
	Gel separador	Química clínica	5 veces
	Sin anticoagulante con silicón	Química clínica, banco de sangre, serología	10 veces

Información en base a extractos de los insertos técnicos e información de los productos BD Microtainer

8.1.6 NIVELES SÉRICOS DE MEDICAMENTOS

El tiempo de colecta de sangre para monitoreo de drogas terapéuticas (MDT) dependerá del medicamento y de la indicación para la prueba (optimización de la dosis del fármaco, monitoreo de la adherencia del fármaco, efectos adversos, intoxicación por drogas, etc.) Se deben seguir recomendaciones específicas del médico encargado del MDT para el momento exacto del muestreo de sangre.

Digoxina: Se debe tomar la muestra 8-12 horas despues de la ultima dosis del medicamento.
Teofilina: Se debe tomar la muestra 8-12 horas después de la ultima dosis del medicamento.
Fenobarbital: Se debe tomar la muestra 8-12 horas después de la ultima dosis del medicamento.
Fenitoina: Se debe tomar la muestra 8-12 horas después de la ultima dosis, se recomienda

recoger las muestras por lo menos 2 horas después de suministrar una dosis intravenosa de fosfenitoína y como minimo, 4 horas tras una dosis intramuscular.l.
Ciclosporina: Se toma dos horas después de la ultima dosis, o según indicación medica.
Vancomicina: El control de las concentraciones mínimas del fármaco deberá iniciarse una vez alcanzado el estado de equilibrio y las muestras deben extraerse en los 30 minutos anteriores a la siguiente dosis.
Cuando están midiendo las concentraciones máximas, las muestras deberán extraerse 0,5-2 horas después de una infusión.
Proteger de la luz.
T**acrolimus:** Se debe tomar 15 minutos antes de la hora habitual de toma del medicamento.

NOTA: En todos los casos se debe relacionar dosis, fecha y hora de la ultima dosis del medicamento. Fecha y hora de la toma de la muestra, vía de administración y diagnostico del paciente.

8.1.7. CURVA DE TOLERANCIA A LA GLUCOSA / GLUCOSA POST CARGA

- Obtener sangre del paciente en ayunas
- Realizar glucometría obteniendo la muestra de punción digital. Si el resultado es mayor de 128 mg/dl no darle la carga.
- El paciente deberá tomar 75 gr de dextrosa diluídos en 300 ml de agua. Para lo cual el paciente debe firmar el consentimiento para la administración de cargas de glucosa.
  Obtener muestras de sangre para cuantificar la glucosa a la media hora, una hora, dos horas, tres horas posteriores a la ingesta de la carga para la curva, o a las dos horas para post carga.
- El paciente debe mantenerse en reposo, no fumar, no comer, no beber durante el tiempo del estudio.

8.1.8. GLUCOSA POSTPRANDIAL

- Obtener sangre del paciente en ayunas
- Indicarle al paciente que debe tomar un desayuno normal y asistir nuevamente al laboratorio exactamente a las dos horas posteriores de haber terminado el desayuno para tomar la segunda muestra de sangre. Durante esas dos horas no se debe hacer ejercicio, ni consumir ningún alimento diferente.

8.1.9. TEST DE O SULLIVAN

- Test para el control de la glucosa post carga de 50 gr durante el embarazo.

- Preguntar a la paciente si se encuentra en ayunas
- Realice una glucometría. Si el valor se encuentra por debajo de 115 mg/dl darle carga de 50gr diluida en 200 ml de agua.
- Tomar la segunda muestra de sangre una hora después de la ingesta.

NOTA: Según la Guía de Practica Clinica para el diagnostico, tratamiento y seguimiento de la diabetes Gestacional del Ministerio de Salud y protección social (2015 guía No GPC-2015-49) se recomienda hacer la prueba de un paso de la IADPSG (International Association of the Diabetes and Pregnancy Study Groups) a todas las pacientes gestantes a partir de la semana 24, para el diagnóstico de la Diabetes Gestacional con 75 gr de glucosa y dos tomas postprandiales a la hora y dos horas. Sin embargo si el médico lo ordena se realiza en test de O Sullivan

8.1.10 GLUCOMETRIA

8.1.10.1. RESUMEN Y PRINCIPIO

El sistema Accu-Chek® Inform II es un dispositivo para uso diagnóstico *in vitro* diseñado en la determinación cuantitativa de los niveles de glicemia en muestras de sangre venosa, capilar, arterial y neonatal .

La estimación cuantitativa de la Glucosa se fundamenta en una reacción enzimática donde en la tira reactiva se convierte la glucosa de la muestra de la sangre en Gluconolactona, esta reaccción genera una corriente eléctrica que el medidor interpreta (Amperometria) y convierte en un resultado de Glicemia.

El procedimiento por el cual se realiza la medición del nivel de glucosa en sangre capilar puede ser:
Preprandial: antes de los alimentos y **Postprandial**: después de los alimentos.

También puede realizarse en los momentos en que la clínica del paciente nos haga pensar en una alteración de los niveles de glucosa en sangre.

8.1,10.2. ALMACENAMIENTO Y MANEJO

Las tiras de reactivas vienen dentro de un recipiente que debe permanecer cerrado.

Almacénelas a una temperatura ambiente.

8.1.10.3. Glucómetro: aparato que mide la cantidad de glucosa de una muestra de sangre tomada generalmente por punción del pulpejo de un dedo (sangre capilar) que es aplicada sobre una tirilla reactiva que es introducida a este aparato para su lectura.

8.1.10.4. Equipo:

- Medidor Accu-Chek
- Tiras reactivas Accu-Chek Performa o Accu-Chek Inform II con el chip correspondiente.
  - Almacene las tiras entre 2 y 30 °C
  - Mantenga las tiras en su envase original tapado
  - Las tiras son estables máximo 3 minutos después de sacarlas del envase por lo que se debe usar inmediatamente.
  - Al sacar la tira del envase tómela por el centro, no toque los extremos.
- Soluciones de control Accu-Chek Performa
  - Mantener las Soluciones a temperatura ambiente (entre 2 y 30 °C)
  - Cada botella de Solución control es estable 90 días Luego de abierta. (no olvide, cerrar la botella Luego de usarla).
- Dispositivo de lanceta aprobado para uso profesional, ajusta según la profundidad requerida.
- Alcohol (si usa alcohol, no olvide secar muy bien la piel). Si es posible solicite al paciente que se lave las manos.
- Recipientes de desechos

8.1.10.5. PROCEDIMIENTO

Una vez solicitada la glucometría por sistema realice la glucometría de la siguiente manera:

- ➢ Pulse el botón de encendido/apagado para encender el medidor.
- ➢ Cuando aparezca la pantalla Inicio, pulse la flecha para acceder a la pantalla ID de usuario o espere 5 segundos para que el medidor acceda a la misma directamente
- ➢ Introduzca o escanee el ID de usuario y pulse para abrir la pantalla del menú principal.
- ➢ Pulse test de paciente para abrir la pantalla ID de paciente.
- ➢ Introduzca o escanee el ID de paciente y pulse a continuación El medidor le pedirá que confirme el lote de tiras del test.
- ➢ Compruebe el lote de tiras reactivas.
  - Si es correcto, pulse . El medidor le pedirá que introduzca la tira para el test.

- Si no es correcto, pulse **X** para seleccionar un numero de lote distinto

Una vez confirmado el lote de tiras reactivas, el sistema le solicitará que introduzca la tira.

- Extraiga la tira reactiva del vial de tiras reactivas y cierre el vial con el tapón.
- Mantenga la tira reactiva de tal manera que la inscripción "ACCU-CHEK" mire hacia arriba.
- Introduzca la tira reactiva en la ranura para tiras reactivas hasta el tope máximo en el sentido que indican las flechas de la tira reactiva. El medidor emitirá una señal acústica.

Cuando el medidor detecta la tira reactiva, solicita que se aplique una muestra de sangre.

- Espere a que aparezca el símbolo de gota parpadeante en pantalla para aplicar la sangre. El medidor volverá a emitir una señal acústica.
- Se recomienda que el paciente se lave y seque las manos, si no es posible limpie el dedo con alcohol, deje secar muy bien , haga la punción y limpie la primera gota
- Aplique la gota de sangre en el **extremo frontal** (área amarilla para dosificación) de la tira reactiva. **No** aplique la sangre en la parte superior de la tira. La tira reactiva absorbe la sangre por acción capilar. La sangre en la parte superior de la tira no está disponible para el test.

Cuando el medidor detecta una cantidad suficiente de muestra de sangre, emite una señal acústica e inicia la medición. El icono de reloj de arena indica la realización de un test.

Cuando finaliza el test y el resultado está listo, el medidor emite de nuevo una señal acústica.

8.2. RECOLECCIÓN DE MUESTRAS PARA PRUEBAS ESPECIALES

8.2.1. CORTISOL

- El paciente debe estar en ayunas
- Se tomarán dos muestras a las 8:00 am y a las 4:00 pm

8.2.2. POOL DE PROLACTINA

- El paciente debe estar en ayunas
- La muestra se debe tomar mínimo dos horas después de levantada la paciente.
- Se tomarán tres muestras cada veinte minutos.

8.3. PARCIAL DE ORINA Y UROCULTIVO

En pacientes pediátricos mayores que controlan esfínteres, la recolección de las muestras por micción espontánea es la técnica ideal por su sencillez y nula invasividad. La situación es contraria en los casos de pacientes pediátricos que no controlan la micción, tema en el que no existen estimadores de validez generalizables; sin embargo, a pesar del riesgo de contaminación que tiene la utilización de una bolsa recolectora con banda adhesiva, éste es uno de los métodos más ampliamente utilizados a nivel mundial para obtener muestras de orina en este segmento de la población. La toma de muestra a través de catéter transuretral y la punción suprapúbica han sido consideradas como los métodos ideales de recolección en población pediátrica, pero su invasividad ha restringido su uso.

8.3.1 Cuidados y Recomendaciones:

- Todos las muestras de orina, a excepción de las muestras tomadas por cateterismo o por punción suprapúbica, deben ser obtenidas por el paciente; por esta razón, se deben tomar las medidas necesarias para evitar la contaminación de la muestra con secreción vaginal, esperma, vello púbico, polvos, aceites, lociones y otros materiales extraños. Las muestras nunca deben ser recogidas de pañales (CLSI, GP16A3, 2009)
- Debe ser la primera micción del día, orina aleatoria.
- Realizar higiene en genitales.

  No está indicada para la detección de anaerobios. Es una muestra importante para detección de infecciones causadas por citomegalovirus (CMV), enterovirus, adenovirus. Para búsqueda de Mycobacterias se realiza la recolección de la misma manera, se realiza cultivo pero no se realiza baciloscopia por la baja especificidad.

Indicar al paciente como recoger la muestra:

8.3.1.1. Hombre

- En el caso de pacientes que no se haya realizado la circuncisión, debe retraerse el prepucio para dejar expuesto el glande y el meato urinario
- Lavar el glande.
- Eliminar la parte inicial del chorro.
- De la parte media del chorro tomar la muestra en recipiente estéril.
- Tapar el recipiente, identificarlo correctamente , colocar la hora de recolección y enviar inmediatamente al laboratorio.

8.3.1.2 Mujer:

- Separar los labios mayores y menores suavemente.
- Efectuar el lavado de adelante hacia atrás con una gasa empapada en agua y jabón.
- Limpiar el exceso de jabón con agua.
- Manteniendo los labios separados eliminar la parte inicial de la micción.
- De la parte media del chorro tomar la muestra en recipiente estéril.

Si por las condiciones clínicas se debe asistir al paciente en la toma de la muestra, previo lavado de manos y colocación de guantes cumpla con las indicaciones anteriores y continúe con:

- Tapar el recipiente, identificarlo correctamente , colocar la hora de recolección y enviar inmediatamente al laboratorio.
- Si el paciente tiene sonda vesical **nunca se debe desempatar la sonda**. Se debe manejar con técnica estéril. Se bloquea el paso por diez a quince minutos, para tomar la muestra se realiza limpieza del extremo distal de la sonda con solución antiséptica y con una jeringa de 10 cc se punciona la sonda, se extrae la muestra y se envía al Laboratorio Clínico, en la misma jeringa, debidamente identificada.
- Si hay que recoger muestra a paciente con sangrado vaginal se debe utilizar sonda.

8.3.1.3 Recolección de muestras para parcial de orina en niños

En niños, se utiliza una bolsa de plástico estéril colectora de orina. Se debe realizar cambio de bolsa después de pasados quince minutos en caso que no se haya presentado micción.

- La bolsa se colocará después de haber lavado los genitales adheriéndola a la piel por medio de un anillo adhesivo.
- En los niños, adherir firmemente la bolsa en la base del pene presionando las bandas sobre la piel del paciente.
- En las niñas, estirar la piel de la región perineal para disminuir los pliegues. Presionar las bandas adhesivas firmemente a la piel alrededor de los genitales. Iniciar en el espacio que hay entre el ano y la vagina para evitar la contaminación de la muestra desde el área rectal.
- Confirmar que no queden pliegues o espacios abiertos en la banda adhesiva.
- Una vez se haya producido la micción del paciente, retirar la bolsa de recolección evitando contaminarla. Cuidadosamente, trasvasar la muestra a un frasco de boca ancha estéril.
- Cerrar el frasco verificando que no se produzcan goteos y rotular la muestra con el nombre y número de identificación del paciente y la hora de recolección

- Se debe realizar cambio de bolsa después de pasados quince minutos en caso que no se haya presentado micción.

8.3.5. Punción suprapúbica

Ocasionalmente, la aspiración por punción suprapúbica de la vejiga puede ser necesaria y está a cargo del médico. Consiste en la punción directa de la vejiga a través de la pared abdominal con aguja y jeringa estériles. (Debe asegurarse que el paciente tenga la vejiga llena antes de iniciar el procedimiento

8.3.6. RECOLECCION DE UROCULTIVO

8.3.6.1. Técnica y Recolección:

- Adultos: Tener en cuenta las mismas indicaciones para la recolección del parcial de orina. Instruir al paciente para que inicie la micción, desechar la primera parte de la orina, introducir el frasco recolector, recoger la parte media de la orina si detener el flujo urinario (5-10 cc) y terminar de eliminar en el sanitario o pato. Tapar el frasco sin contaminar la muestra.

- Pediátrico: Para realización de Urocultivo de muestras de orina de niños, debe realizarse por medio de sonda. No podrá utilizarse bolsa recolectora de orina, por la alto riesgo de contaminación que representa.

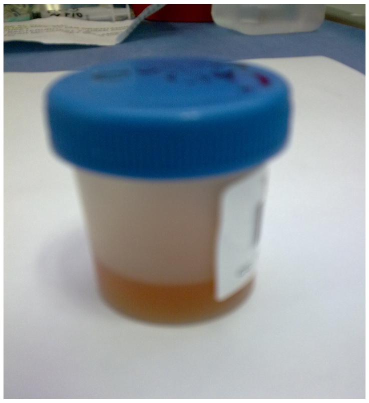

8.3.6.2. Transporte:

Todas las muestras que sean llevadas al laboratorio deben cumplir con ciertas condiciones:

- Siempre los frascos recolectores deben ser de boca ancha y de tapa rosca para

asegurar el cierre hermético de los frascos y evitar goteos.

- Las muestras deben ser rotuladas con el nombre y número de identificación del paciente, así como la fecha y hora de la recolección.
- Idealmente, las muestras deben ser transportadas inmediatamente al laboratorio clínico.
- Registro en el cuaderno que se encuentra en el servicio y hacer firmar el recibido en el Laboratorio.

8.3.7. RECOLECCIÓN DE ORINA DE 3 HORAS

- Orinar por la mañana al levantarse y anotar exactamente la hora teniendo en cuenta que esta muestra no se utilizará para el examen.
- Recolectar las muestras posteriores de orina de la mañana y tarde en el recipiente proporcionado por el laboratorio ó en un frasco de agua mineral limpio por un lapso de 3 horas
- Conserve el frasco en la nevera durante la recolección.
- Llevar la muestra una vez finalizada al laboratorio.
- Recomendar al paciente la **NO** ingesta de bebidas alcohólicas.

8.3.8. TOMA DE MUESTRAS PARA INVESTIGACIÓN DE BACILO DE KOCH (cultivo de BK)

- Se recolecta la orina de la primera micción. Se recomienda recogerla en frascos de agua cristal con capacidad de 250 ml.
- Llevar la muestra una recolectada y la orden médica al laboratorio.
- Recomendar al paciente evitar el consumo de bebidas alcohólicas.

8.4. RECOLECCIÓN DE MUESTRAS PARA COPROLÓGICO Y COPROSCOPICO

- Recolectar la muestra en el recipiente especial para la muestra
- No reciba muestras que vengan en recipientes inadecuados
- La muestra no debe estar contaminada con orina, ni con agua ya que puede inhibir el crecimiento bacteriólogico o puede contener microorganismos de vida libre.
- Algunos fármacos y el aceite mineral pueden afectar la prueba.

8.4.1. SANGRE OCULTA

- No debe haber evidencia de sangrados odontológicos.
- No recoja la muestra si está con el período.

8.5. TRACTO RESPIRATORIO SUPERIOR E INFERIOR

Las enfermedades respiratorias se pueden dividir en dos tipos: las del tracto respiratorio superior y las del tracto respiratorio inferior. Las primeras habitualmente comprometen los oídos, las mucosas de la cavidad nasal y la faringe hasta por encima de la epiglotis. Los principales agentes etiológicos de las enfermedades del tracto respiratorio superior son los virus, como el virus sincitial respiratorio, el virus de la influenza y adenovirus, entre otros; los principales agentes bacterianos responsables de las enfermedades del tracto respiratorio superior dependerán de la localización de la infección como por ejemplo *Streptococcus pneumoniae y Haemophilus influenzae* en otitis media y sinusitis aguda y *Streptococcus pyogenes* en faringitis bacteriana.
Habitualmente, los especímenes utilizados con mayor frecuencia en las infecciones del tracto respiratorio superior incluyen los hisopados faríngeos, los hisopados o lavados nasofaríngeos, los hisopados de la cavidad oral.
Los especímenes que con mayor frecuencia son utilizados para poder identificar los microorganismos que producen las enfermedades del tracto respiratorio inferior, son el esputo, el esputo inducido, el aspirado traqueal, el lavado bronquial y el lavado broncoalveolar.

8.5.1. Toma de la muestra.

Las muestras del tracto respiratorio deben ser recolectadas tan pronto sea posible, antes del inicio de la terapia con antibióticos. La posibilidad de recuperar los virus y las bacterias disminuye significativamente después de 72 horas de iniciados los síntomas de la enfermedad y después de iniciar la terapia con antibióticos.

8.5.2 Hisopado de fosas nasales

Utilizado principalmente para la detección de hongos y/o *Staphyloccocus aureus* meticilino resistente por medio de cultivo .

8.5.2.1 Cuidados y recomendaciones

- Evitar las gotas y los baños nasales antes de tomar la muestra
- No se recomienda enviar cultivos para anaerobios
- El frotis y cultivo nasal no esta indicado para el diagnóstico de sinusitis, otitis media o infecciones del tracto respiratorio inferior
- Solo se recomienda tomar cultivo de fosas nasales anteriores para la detección de portadores de *Staphylococcus aureus* o en lesiones nasales. Para búsqueda de hongos se recomienda la misma técnica.

8.5.2.2 Técnica de Recolección

- Colocar el paciente bajo una buena fuente de luz
- Levantar la cabeza del paciente y con la otra mano introducir el escobillón humedecido 1 a 2 cm en el interior de las fosas nasales, rotarlo contra la mucosa nasal por un lapso de 10 a 15 segundos y luego retirarlo e identificar de qué fosa nasal se tomo la muestra.
- Rotular la muestra con el nombre y número de identificación del paciente y la hora de recolección.

8.5.2.3 Transporte

➢ El transporte de las muestras hasta el laboratorio debe realizarse lo más pronto posible, idealmente dentro de las dos primeras horas después de tomadas las muestras manteniéndose a temperatura ambiente (L Raka, 2012). Sí esto no es posible, las muestras deben ser refrigeradas inmediatamente después de ser tomada, en una temperatura entre los 4ºC y los 8ºC por un máximo de 48 horas.

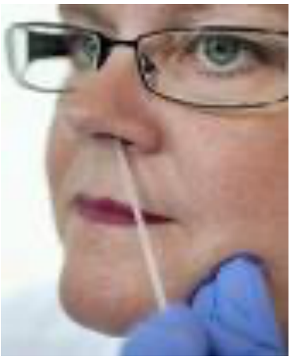

8.5.3. Hisopado nasofaríngeo

El hisopado nasofaríngeo se utiliza para cultivos bacterianos, estudios de inmunofluorescencia y para estudios de PCR en tiempo real (FILMARRAY), para detección de microorganismos causantes de enfermedades respiratorias incluyendo el SARS-COV-2 (COVID-19)

- Humedecer con solución salina los hisopos.
- Con la mano libre, llevar hacia atrás la cabeza del paciente y con la otra mano, introducir el hisopo humedecido a través de los orificios nasales, paralelo al paladar (no hacía arriba), hasta que se encuentra resistencia o la distancia equivalente desde la fosa nasal hasta la oreja. En este punto se encuentra la punta en la nasofaringe.

- Rotar suavemente el hisopo por 5 segundos y luego retirar lentamente, permitiendo que se absorban las secreciones en el hisopo.
- Colocar el hisopo en el tubo.
- Repetir el procedimiento en la fosa nasal contra lateral.
- Una vez terminado el proceso retirar los guantes y realizar higiene de manos.
- Rotular la muestra con el nombre y número de identificación del paciente y la hora de recolección.

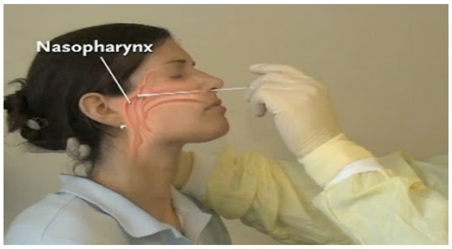

8.5.4. Aspirado nasofaríngeo

El aspirado nasofaríngeo es el método de elección para realizar el diagnóstico del virus de la influenza y otros virus respiratorios . De igual manera, es el método ideal para la búsqueda de *Bordetella pertussis* en población pediátrica.

8.5.4.1. Técnica de recolección

- Con la mano libre, llevar hacía atrás la cabeza del paciente e introducir 1 a 2 mL de solución salina estéril (pH 7,0) en una de las ventanas nasales utilizando la jeringa unida a la sonda.
- Introducir la sonda con 2 a 3 mL de solución salina a través de un orificio nasal, paralelo al paladar (no hacía arriba)
- Aspirar la muestra hasta obtener el mayor volumen posible en el interior de la jeringa; allí se debe evidenciar que la solución salina este turbia, lo cual garantiza que las secreciones han sido recolectadas correctamente.
- Retirar la sonda cuidadosamente de la fosa nasal.
- Tapar firmemente el tubo de recolección y limpiar su exterior exhaustivamente con ayuda de una gasa humedecida con alcohol isopropílico al 70%.

8.5.4.2 Transporte

El transporte de las muestras hasta el laboratorio debe realizarse lo más pronto posible, idealmente dentro de las dos primeras horas después de tomadas las muestras manteniéndose a temperatura ambiente (L Raka, 2012). Sí esto no es posible, las muestras

deben ser refrigeradas inmediatamente después de ser tomada, en una temperatura entre los 4ºC y los 8ºC por un máximo de 48 horas.

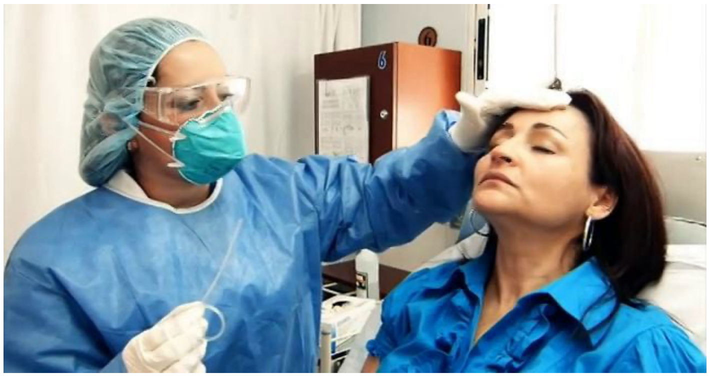

8.5.5. Hisopado faríngeo

El hisopado faríngeo se utiliza para cultivos bacterianos esencialmente para la búsqueda de *Streptococcus pyogenes*. Sin embargo, también es utilizado en la búsqueda de *Corynebacterium diphteriae y Neisseria.* La detección de levaduras y hongos es generalmente restringida a la evaluación microscópica de la coloración de Gram. (P Murray, 2010)
El hisopado de garganta está contraindicado en pacientes con diagnóstico de epiglotitis.

8.5.5.1 Técnica de Recolección

- No hacer gárgaras ni limpieza con ninguna solución bucofaríngea
- Con la mano libre, llevar hacia atrás la cabeza del paciente y pedirle que abra la boca; con ayuda de un bajalenguas, presionar la lengua hacia abajo para facilitar la visualización de la faringe y evitar la contaminación del hisopo.
- Evitando tocar la lengua, los dientes o las encías, ingresar el hisopo hasta la faringe posterior y la región amigdalina. Frotar el hisopo contra las paredes amigdalinas y la orofarínge posterior.
- Retirar el hisopo de la boca y colocarlo inmediatamente en el tubo.

8.5.5.2. STREPTO A TEST

8.5.6.2.1. Obtención y preparación de la muestra:

Recolecte las muestras de frotis de garganta con el hisopo de dacrón estéril que provee el estuche. Transporte el hisopo conteniendo medio modificado Stuart's o Amies. Frote la faringe posterior, amígdalas y otras áreas inflamadas. Evite tocar la lengua, la parte interna de los pómulos y los dientes con el hisopo.

La prueba debe ser realizada inmediatamente después de que las muestras han sido recolectadas. Las muestras de hisopo pueden almacenarse a temperatura ambiente hasta por 4 horas antes de tomar la prueba.

Si se desea realizar un cultivo, ruede ligeramente la punta del hisopo de dacrón para toma de muestra faríngea sobre un Agar Sangre, antes de ser utilizado el hisopo en la prueba rápida.

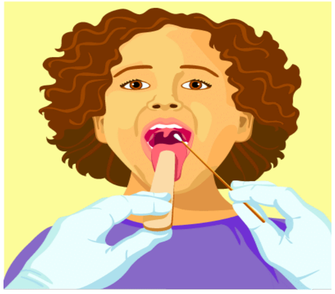

8.5.6 Esputo

Teniendo en cuenta la facilidad para la obtención de las muestra de esputo, esta técnica es ampliamente utilizada en los pacientes no complicados para orientar adecuadamente el diagnóstico clínico.

8.5.6.1 Condiciones del paciente

- Recolectar la muestra en la mañana.
- Indicar al paciente que antes de tomar la muestra, debe realizar previamente un lavado de la cavidad oral con agua para disminuir el exceso de flora bacteriana. (P Murray, 2010)
- Estar preferiblemente en ayunas.

8.5.6.2 Técnica de recolección

- Explicar al paciente que debe toser profundamente para movilizar las secreciones del tracto respiratorio inferior.
- Indicar al paciente que expectore y el esputo generado recogerlo en el frasco estéril.
- Si está intubado, la terapeuta respiratoria coloca la trampa para secreciones entre el

catéter de aspiración y la succión, obtiene la muestra, la identifica correctamente y la envía inmediatamente al Laboratorio clínico.

- Cerrar el frasco inmediatamente verificando que no se encuentre contaminado con secreciones en la superficie externa.

8.5.6.3 Recomendaciones

- Para el estudio de micobacterias se deben obtener tres (3) muestras seriadas
- Las muestras se pueden obtener en cualquier momento del curso clínico de la enfermedad, pero se recomienda que sean tomada antes del inicio de la terapia antibacteriana.
- La presencia de abundantes células epiteliales es un fuerte indicador de contaminación con flora bacteriana oral. Un espécimen contaminado no puede ser aceptado para cultivos bacterianos de rutina, pero si pueden ser tenidos en cuenta para cultivos de micobacterias.
- Una muestra adecuada para realizar el cultivo debe ser representativa de la vía aérea inferior (contener menos de 10 células epiteliales y más de 25 polimorfonucleares por campo de bajo poder).

8.5.7. Esputo inducido

La toma de muestras de esputo inducido con nebulizaciones con solución salina al 0,9% se encuentra indicada en pacientes con dificultad para obtener la muestra por expectoración.

8.5.7.1 Técnica de Recolección

- Realizar nebulización con 3 mL de solución salina normal al 0,9% al paciente.
- Explicar al paciente que debe toser profundamente para movilizar las secreciones del tracto respiratorio inferior.
- Indicar al paciente que expectore y el esputo generado recogerlo en el frasco estéril.

8.5.7.2 Recomendaciones

- Para el estudio de micobacterias se deben obtener tres (3) muestras seriadas.
- Las muestras se pueden obtener en cualquier momento del curso clínico de la enfermedad, pero se recomienda que sea tomada antes del inicio de la terapia antibacteriana.
- A diferencia del esputo obtenido por expectoración, el esputo inducido puede tener contaminación por abundantes células epiteliales y bacterias de la cavidad oral. El

espécimen debe ser procesado para bacterias a pesar de la contaminación de la cavidad oral.
- El esputo inducido debe realizarse en una habitación individual con adecuada aireación para evitar el riesgo generado por la aerolización de las micobacterias.

8.5.8. Aspirado traqueal

El aspirado traqueal es utilizado para determinar el agente etiológico en pacientes con neumonía. La contaminación de las muestras con microorganismos de la cavidad oral puede ser muy frecuente. Siempre se debe recordar que las muestras de aspirados traqueales, deben ser cultivados a pesar de la presencia de células epiteliales en el extendido (P Murray, 2010). El extendido del aspirado traqueal, en ausencia de células inflamatorias y la negatividad del cultivo para gérmenes comunes, tiene un alto valor predictivo negativo (E Baron, 2013; 57).

8.5.8.1. Técnica de Recolección

- Introducir la sonda Nelaton hasta la cavidad traqueal a través del tubo endotraqueal o del orificio de la traqueostomía.
- Conectar el succionador al extremo distal de la sonda y aspirar cuidadosamente el contenido de la cavidad traqueal.
- Retirar lentamente la sonda teniendo siempre ocluido el orificio distal de la sonda o conectando el succionador a la sonda. Con una gasa estéril, limpiar la superficie externa de la sonda.
- Colocar el extremo proximal de la sonda en el frasco estéril. Con la hoja de bisturí, cortar el extremo distal de la sonda, garantizando el vaciamiento del contenido de la sonda dentro del contenedor.

8.5.9. Lavado broncoalveolar, cepillado bronquial y lavado bronquial.

El lavado broncoalveolar, el cepillado bronquial y el lavado bronquial se encuentran indicados en diferentes patologías de acuerdo a la sospecha clínica o en casos donde el esputo no ha sido conclusivo y se continua con sospecha de infección bacteriana, fúngica o viral (incluido *Pneumocystis jirovecii.*) del tracto respiratorio inferior o del parénquima pulmonar. (L Raka, 2012).
Las muestras del lavado broncoalveolar son las ideales para la realización de cultivos, estudios citopatológicos y pruebas de biología molecular. Por medio del fibrobroncoscopio, se puede obtener lavado broncoalveolar del segmento pulmonar deseado, instilando 300 a 350 mL de solución salina normal (0,9%) en alicuotas de 50 mL, que posteriormente serán aspirados y recogidos en un frasco estéril de boca ancha. (L Raka, 2012)
La realización de la Fibrobroncoscopia requiere ser realizado por el médico especialista con entrenamiento en procedimientos endoscópicos.

8.5.10. Recomendaciones para el embalaje y el transporte de las muestras al laboratorio

Llos hisopos y las sondas deben ser recolectados en tubos estériles con medios de transporte sin antibióticos. Las muestras de secreciones, lavados y expectoraciones deben ser recogidas en frascos estériles de boca ancha.
El transporte de las muestras hasta el laboratorio debe realizarse lo más pronto posible, idealmente dentro de las dos primeras horas después de tomadas las muestras manteniéndose a temperatura ambiente (L Raka, 2012). Sí esto no es posible, las muestras deben ser refrigeradas inmediatamente después de ser tomada, en una temperatura entre los 4ºC y los 8ºC por un máximo de 48 horas. Si el tiempo de procesamiento de la muestra será mayor a 48 horas, la muestra puede ser congelada por 2 meses a -20ºC o por 6 meses a -70ºC.

8.5.11. SECRECIONES DEL OÍDO MEDIO Y SECRECIONES OCULARES

La otitis media aguda es una de las infecciones más frecuentes en pediatría, razón por la cual el tratamiento es esencialmente empírico, práctica clínica que puede ser responsable de la elevada tasa de resistencia a β-lactámicos y macrólidos de los agentes etiológicos de la otitis, principalmente el *Streptococcus pneumoniae* (Gené, 2004).
Las infecciones conjuntivales, aunque generalmente no producen un riesgo a largo plazo, son responsables del 1% de las consultas; ocho de cada diez niños tienen un episodio conjuntival una vez al año y habitualmente los médicos ordenan antibióticos para tratar a los pacientes.

8.5.11.1 SECRECIÓN OCULAR

8.5.11.1.1 Cuidados y Recomendaciones

- No usar gotas oftálmicas 18-24 horas antes de la muestra
- Ausencia de cualquier cosmético
- No tomar antibióticos 24-48 horas antes
- No usar anestésicos que poseen actividad antimicrobiana

8.5.11.1.2 Técnica de Recolección

- Con solución salina al 0,9%, limpiar la superficie externa del ojo comprometido.
- Con los dedos separar el párpado inferior y rotar el hisopo humedecido con solución salina frotando el borde interno de la conjuntiva desde el fórnix conjuntival en el borde

nasal hasta el borde temporal.

- Colocar el hisopo en un tubo estéril con medio de transporte para el laboratorio.
- Repetir con el segundo hisopo el procedimiento y realizar un extendido sobre la lámina portaobjeto
- Nuevamente repetir el procedimiento para el ojo contralateral.
- Tapar los tubos con el medio de transporte, verificando que no existan fugas para el transporte.
- **Raspado corneal:** Esta muestra es recolectada por el especialista. Use espátula estéril y raspe las lesiones o úlceras e inocule la muestra en el medio de transporte o en tubo estéril seco no tapa rosca o tapón de caucho; realice extendido en lámina de vidrio.
- **Aspirado de fluido vítreo:** Utilice la técnica aséptica para realizar punción por aspiración

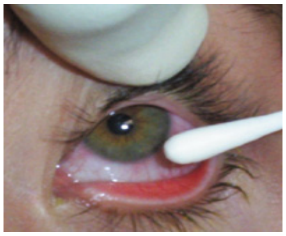

8.5.11.3. Transporte

- Inmediatamente luego de la recolección a temperatura ambiente.

8.5.12. CONDUCTO AUDITIVO EXTERNO

La toma de muestras de secreciones óticas puede ser realizada a partir de la timpanocentésis o de la recolección de la efusión espontanea del oído medio tras una otitis media supurativa. La primera se considera el método de elección por tener un menor riesgo de contaminación y dar una solución terapéutica inmediata para el paciente (Pichichero, 2013). La recolección con hisopo de la efusión del oído medio es una opción cuando ésta se ha presentado espontáneamente (AS Adoga, 2010) **.**

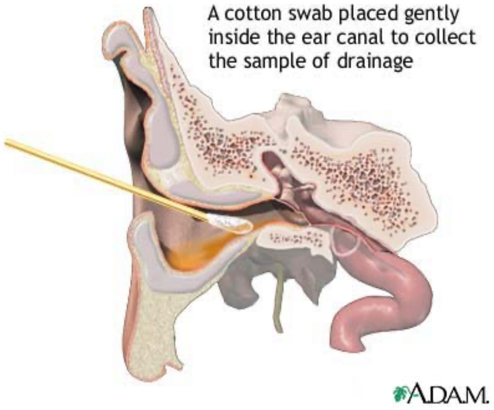

8.5.12.1 Cuidados y Recomendaciones:

- La toma de muestras de secreción del oído medio y de secreciones oculares debe ser tomada antes de iniciar tratamientos antibióticos locales o sistémicos.
- No usar gotas óticas de 18 a 24 horas antes de la toma de muestra
- No tomar o aplicar antibióticos de 24-48 horas antes

8.5.12.2. Transporte

El transporte de las muestras hasta el laboratorio debe realizarse lo más pronto posible, máximo dentro de las dos primeras horas, después de tomadas las muestras manteniéndose a temperatura ambiente Se debe evitar refrigerar las muestras hasta su procesamiento.

8.6 HEMOCULTIVOS

Los hemocultivos se han convertido en el *estándar de oro* para la detección de bacteremias y fungemias. La recuperación de microorganismos circulantes en la sangre de los pacientes tiene gran importancia diagnóstica y pronóstica, ya que indica la falla del sistema inmune del paciente para contener los procesos infecciosos en su localización primaria. La presencia de un hemocultivo positivo permite establecer el agente etiológico y la susceptibilidad de los microorganismos a los antibióticos, facilitando su adecuado tratamiento.
Un principal factor determinante de la capacidad de obtener resultados positivos y el aislamiento del microorganismo causante está relacionado con la extracción del volumen adecuado de muestra, por tanto el volumen de sangre que se cultiva es crucial para lograr la detección de los microorganismos, ya que volúmenes más bajos de los óptimos pueden llevar a obtener resultados falsos negativos.

8.6.1 Definiciones

- **Antiséptico:** Sustancia que inhibe el crecimiento y desarrollo de microorganismos.

- **Sistema automatizado de hemocultivos:** Sistema mecánico automatizado que permite agitar, monitorizar e incubar las botellas de hemocultivo para evaluar el crecimiento de microorganismos.

- **Bacteremia:** Es la presencia de bacterias en el torrente sanguíneo. Dependiendo del tipo de bacteria y tiempo de crecimiento puede ser considerada como causa de la sepsis o un agente contaminante al momento de la toma de la muestra.

- **Hemocultivo:** Muestras que son tomados de un paciente para evaluar la presencia de bacterias u hongos en el torrente sanguíneo.

- **Juego (sets) de hemocultivos:** Es el número de botellas de hemocultivos (2 ó 3 en pacientes adultos y para población pediátrica depende del volumen extraído) en los que se siembra una muestra de sangre del paciente obtenida de un mismo sitio de punción. Habitualmente está conformado por una botella para detección de microorganismos anaerobios y una para detección de microorganismos aerobios.

- **Agente Contaminante:** Microorganismo aislado de un hemocultivo, el cual fue introducido en éste durante la toma de la muestra o el procesamiento del hemocultivo, razón por la cual no se considera como agente etiológico.

- **Medio de Cultivo:** Sustancia enriquecida con factores necesarios para el crecimiento de los microorganismos.

- **Desinfectante:** Sustancia que reduce la concentración de bacterias, hongos o virus sobre una superficie.

- **Fungemia:** Presencia de hongos (hifas o levaduras) en el torrente sanguíneo.

- **Volumen Inadecuado de Sangre:** Cuando el volumen de sangre inoculado en la botella de hemocultivo es menor al 80% del volumen mínimo requerido en la etiqueta de la botella.

- **Sepsis:** Síndrome de respuesta inflamatoria sistémica producto de una infección.

- **Venopunción:** Punción de una vena para la obtención de la muestra de sangre.

● **Especificidad:** Probabilidad que el cultivo no muestre crecimiento si la bacteremia es ausente. Es decir, la especialidad caracteriza la capacidad de la prueba para detectar la ausencia de la enfermedad en sujetos sanos.
● **Sensibilidad:** Probabilidad que el cultivo muestre crecimiento si la bacteremia está presente: verdaderos positivos. Es decir, la sensibilidad caracteriza la capacidad de la prueba para detectar la enfermedad en sujetos enfermos.

8.6.2 Condiciones para la toma de la muestra.

- Los hemocultivos deben ser obtenidos simultáneamente en diferentes punciones o con poco tiempo de diferencia entre cada uno. La toma de estos en intervalos de tiempo está indicada solo cuando es necesario documentar bacteremias continuas en pacientes con sospecha de endocarditis infecciosa o en infecciones endovasculares.
- De ser posible, tomar los hemocultivos antes de iniciar terapia antimicrobiana, se recomienda el uso de botellas para hemocultivo con resinas, las cuales atrapan el antibiótico facilitando el crecimiento de los microorganismos.
- En el caso de hemocultivos obtenidos de venas periféricas, se recomienda utilizar la vena mediana cubital o las venas cefálicas de los miembros superiores. No se recomienda extraer la sangre de líneas periféricas ya canalizadas, en el caso de líneas centrales la toma de sangre a partir de ellas está indicada para la investigación de infecciones asociadas con la misma y siempre precedida de la toma de una muestra de sangre de vena periférica.
- Las botellas de hemocultivos en su parte externa no son estériles, razón por la cual se recomienda desinfectar la tapa de caucho de la botella utilizando alcohol isopropílico al 70% (CLSI, 2012).
- El volumen de sangre obtenido durante la toma de hemocultivos es el factor determinante en la recuperación del microorganismo causante de la infección. La tasa de aislamiento de patógenos obtenidos de hemocultivos aumenta con la cantidad de sangre tomada.
- En pacientes adultos idealmente se debe obtener un volumen mínimo de 10 mL por cada juego de hemocultivos (volumen extraído por cada venopunción). Se recomienda tomar dos juegos de dos sitios anatómicos diferentes (En la Fundación Clínica Shaio se toma 2 juegos, cada juego incluye un hemocultivo anaerobio y uno aerobio)
- En neonatos, algunos estudios sugieren que la inoculación de 1 mL de sangre en la botella puede ser el volumen suficiente para obtener una adecuada sensibilidad cuando se utiliza una botella única.
- En neonatos y prematuros se recomienda tomar dos juegos, cada uno con un volumen mínimo de 0.5 mL.

Volumen de sangre a extraer por juego de Hemocultivo según edad y peso en

población pediátrica

Población	Edad	Sitio	Volumen Minimo	Botellas
Neonatos	0-28 dias (o pacientes en URN)	vena periferica	<8 kg: 1 mL	Una botella pediatrica aerobica
Niños	1-3 meses	vena periferica	<8 kg: 1 mL	Una botella pediatrica aerobica
	3-36 meses	vena periferica	<8 kg: 1 mL<br>8-13 kg: 3 mL<br>13-27 kg: 5 mL	Botella pediatrica aerobica si el volumen es menor de 0,5 - 4 mL Bottella aerobica de adulto si el volumen es mayor de 4,0 mL
	4-11 años	vena periferica	8-13 kg: 3 mL<br>13-27 kg: 5 mL<br>27-40 kg: 10 mL >40 kg: 10 mL	Botella pediatrica aerobica si el volumen es menor de 0,5 - 4 mL Botella aerobica de adulto si el volumen es mayor de 4,0 mL
	12-17 años	vena periferica; considerar dos venas de sitios separados para 2 cultivos	27-40 kg: 10 mL >40 kg: 10 mL	Botella pediatrica aerobica si el volumen es menor de 0,5 - 4 mL Botella aerobica de adulto si el volumen es mayor de 4,0 mL

Tomado de: BLOOD CULTURES AND CENTRAL CATHETERS: IS THE "EASIEST WAY" BEST PRACTICE? Margo Halm, RN, PhD, ACNS-BC, Tracy Hickson, MLS (ASCP), CMSM, Deanna Stein, RN, Matthew Tanner, PharmD, BCPS, and Sheila VandeGraaf, PBT (ASCP)

8.6.3. Cuidados y Recomendaciones

- Realizar lavado de mano quirúrgico
- Mantener técnica aséptica durante todo el procedimiento.
- Realizar antisepsia de la zona a puncionar; no palpe la vena sin guantes estériles una vez preparada la piel.
- Utilizar guantes estériles para cada punción

8.6.4 Técnica de recolección

- Obtener cada muestra de sitios anatómicos diferentes (NO Arterial, NO miembros inferiores, genera mayores complicaciones y contaminación).
- Antes de acercarse al entorno del paciente para tomar este tipo de muestra debe realizar higiene de manos.
- Desinfectar la tapa de caucho de la botella de hemocultivo utilizando una gasa estéril humedecida con alcohol isopropílico al 70% y dejar secar.

- Ubicar al paciente en posición apropiada, poner el torniquete, seleccionar y localizar la vena adecuada. Para ello, palpar y hacer seguimiento del trayecto de la vena en el brazo con el dedo. Verificar que el sitio de la venopunción se encuentra completamente normal.
- Colocar gorro, tapabocas, gafas de seguridad o mascarilla visual y bata.
- Realizar higiene de manos e inmediatamente colocar guantes estériles.
- Realizar asepsia y antisepsia del sitio de venopunción con 3 pañines impregnados con alcohol isopropílico al 70%, frotando el área. Dejar 30 segundos permitiendo que la piel se seque para la primera toma y 60 a 120 segundos en la segunda. En situaciones de hipersensibilidad se recomienda el uso de soluciones con bases yodadas.
- Puncione con la jeringa de 10mL inmediatamente retirada la jeringa inocule la botella anaerobica y realice nuevamente el procedimieto de el otro brazo para la(s) botella(s) aerobica(s) .
- Mezclar por inmersión las botellas.

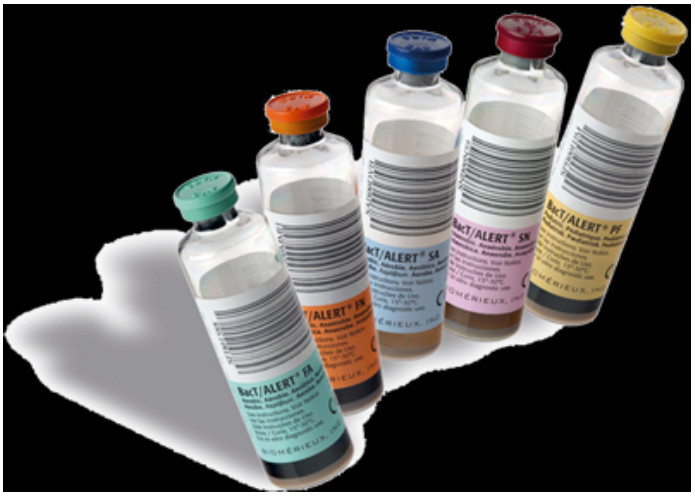

8.6.5. **Toma de muestra de hemocultivos a través de catéter central**

Para toma de muestra por vía de catéter central, en el caso de sospechar infecciones del torrente sanguíneo asociadas con el dispositivo se debe tomar simultáneamente un juego de hemocultivo por vena periférica.

8.6.5.1 Técnica de Recolección

- Antes de acercarse al entorno del paciente e iniciar el procedimiento debe realizar higiene de manos.
- Colocar guantes estériles.
- Cerrar el paso de infusión intravenosa, durante 3 – 5 minutos (dependiendo de la condición del paciente).
- Elegir un puerto próximo, realizar limpieza por 15 segundos utilizando pañines impregnados con alcohol isopropílico al 70% y permitir que seque.
- En adultos extraer 20 mL de sangre de la vía y dividir el contenido de la jeringa así: 10 mL para la botella anaeróbica y 10 mL para la botella aeróbica sin cambiar de aguja. Siempre inocular primero la botella anaeróbica y luego la aeróbica. Para población pediátrica seguir recomendaciones de volumen de acuerdo a la edad y peso del paciente.
- Mezclar por inmersión las botellas.

8.7. PUNTA DE CATETER

El cultivo cuantitativo (método de MAKI) es el método que provee mayor exactitud para hacer el diagnóstico de una infección del torrente sanguíneo relacionada directamente con el catéter.
Antes de tomar la muestra de la punta del catéter, obtener los juegos de hemocultivo por venopunción periférica y el juego de hemocultivo a través del catéter centra.

8.7.1 Técnica de Recolección

- Antes de acercarse al entorno del paciente para tomar este tipo de muestra debe realizar higiene de manos.
- Colocar gorro, tapabocas, gafas de seguridad o mascarilla facial
- Realizar higiene de manos.
- Colocar bata quirúrgica y guantes.
- Realizar asepsia y antisepsia del sitio de implantación del catéter, dejar 30 segundos o 60 segundos si se utiliza alguna solución con bases yodadas; permitir que se seque.
- Retirar el catéter e inmediatamente, cortar la punta del dispositivo a 4 ó 5 cm del extremo distal utilizando una pinza y una tijera estéril.
- Colocar inmediatamente el segmento cortado en un tubo seco estéril.

8.8. Transporte

Todas las botellas de hemocultivos y cultivos de punta de catéter, deben ser enviados al laboratorio lo más pronto posible, idealmente en los primeros quince minutos y no más de dos horas de haber sido tomadas las muestras. El retraso en el ingreso de las botellas a los equipos para hemocultivos, puede retrasar o impedir la detección del crecimiento de microorganismos.
Después de haber sido inoculadas las botellas de hemocultivos, se recomienda mantener el menor tiempo posible a temperatura ambiente. Nunca se deben refrigerar o congelar las botellas por el alto riesgo de muerte los microorganismos (CLSI M47A,2012).

9.0 LÍQUIDOS CORPORALES ESTÉRILES

Los líquidos corporales estériles corresponden a los líquidos que se generan en las diferentes cavidades corporales las cuales se encuentran completamente aisladas del medio externo y las cuales pueden ser alcanzadas por microorganismos por medio de la inoculación directa (trauma, iatrogenia), infección a través del torrente sanguíneo o infección de los tejidos adyacentes.
La toma de las muestras obtenidas a través de las cavidades corporales, es un procedimiento invasivo susceptible de complicaciones. El procedimiento debe ser realizado exclusivamente por personal médico entrenado.

9.1 Definiciones

- **Medio de Cultivo:** Sustancia enriquecida con factores necesarios para el crecimiento de los microorganismos.

- **Desinfectante:** Sustancia que reduce la concentración de bacterias, hongos o virus en una superficie.

- **Especificidad:** Probabilidad que el cultivo no muestre crecimiento si la infección está ausente.

- **Sensibilidad:** Probabilidad que el cultivo muestre crecimiento si la infección está presente.

- **Artrocentesis:** Aspiración aséptica de líquido ubicado en la cavidad articular.

- **Toracocentesis:** Aspiración aséptica de líquido alojado en la cavidad pleural.

- **Paracentesis:** Aspiración aséptica de líquido alojado en la cavidad abdominal.
- **Agente Contaminante:** Microorganismo aislado de un cultivo, el cual fue introducido dentro del cultivo durante la toma de la muestra o el procesamiento del cultivo, razón por la cual no se considera como agente etiológico.

- **Aislamiento Indeterminado:** Microorganismo clínico aislado cuya importancia clínica no se encuentra establecida.

9.1.1 Condiciones para la toma de la muestra

- Las muestras procedentes de cavidades corporales aisladas del medio externo, deben ser recolectadas y llevadas al laboratorio para su procesamiento tan pronto sea posible para garantizar la viabilidad de microorganismos de crecimiento difícil y evitar el sobrecrecimiento de bacterias contaminantes.
- Los líquidos susceptibles de formar coagulo deben ser colectadas en tubos con anticoagulantes SPS (polisulfonato de sodio) para pruebas especiales, excepto si se está haciendo búsqueda de *Neisseria meningitidis, Neisseria gonorrhoeae, Peptostreptococcus y Gardenella vaginalis*. En este caso se puede utilizar anticoagulante tipo heparina, citrato de sodio y EDTA (ácido etilendiaminotetracético).

9.2. Efusiones de la cavidad pleural (Toracocentesis)

Las efusiones pleurales a menudo se encuentran acompañando neumonías bacterianas. Cerca del 17% de las neumonías adquiridas en la comunidad se encuentran asociadas a efusiones pleurales, principalmente en aquellos pacientes que no respondieron a terapia antimicrobiana temprana. La toracocentesis es un procedimiento diagnóstico y en algunas ocasiones terapéutico.

9.3 Efusiones de la cavidad peritoneal (Paracentesis)

La paracentesis abdominal es el procedimiento mediante el cual se extrae líquido ascítico contenido en la cavidad peritoneal. Los estudios microbiológicos se encuentran indicados cuando se tiene sospecha de peritonitis primaria o secundaria; la positividad de los cultivos en presencia de un conteo elevado de PMN podría corresponder a un diagnóstico de peritonitis. (L Raka, 2012)

9.4. Efusiones de las cavidades articulares (Artrocentesis)

La artrocentesis es uno de los procedimientos que con mayor frecuencia se realiza en la práctica médica para el diagnóstico y el tratamiento de las enfermedades de la cavidad articular. La aspiración simple del exceso de líquido sinovial de la articulación puede explicar las causas de la enfermedad y ayudar en el tratamiento disminuyendo la presión intraarticular.

9.5. Cavidad pericárdica (Pericardiocentesis)

La pericardiocentesis es un procedimiento diagnóstico que puede ser utilizado en la práctica clínica para el diagnóstico de presencia de líquido (sangre, líquido pericárdico, material purulento) en la cavidad pericárdica. En el diagnóstico microbiológico, cualquier crecimiento de un patógeno en los cultivos tiene significancia clínica.

9.6 Líquido Cefalorraquídeo - LCR (Punción Lumbar)

Las principales indicaciones diagnósticas para la toma de LCR incluyen las enfermedades infecciosas, enfermedades inflamatorias y neoplasias que comprometen el sistema nervioso central. También se encuentra indicado el procedimiento en la administración intratecal de anestésicos, antibióticos, quimioterápicos y antiespásticos.

9.7. Transporte de las muestras

Todas las muestras, tubos y botellas recogidas deben ser rotulados con el nombre del paciente, el número de identificación, el tipo de espécimen, la fecha de recolección y el tipo de estudio a realizar.
El transporte de las muestras hasta el laboratorio debe realizarse lo más pronto posible, idealmente dentro de los primeros 15 minutos y máximo dos horas después de tomadas las muestras manteniéndose a temperatura ambiente (L Raka, 2012). La orden médica debe incluir la totalidad de las pruebas solicitadas para el procedimiento. Por la dificultad para la recolección de estas muestras, se deben tomar acciones encaminadas a disminuir el riesgo de errores preanalíticos en el laboratorio.

10.0 PIEL Y TEJIDOS BLANDOS

Las infecciones que comprometen la piel y los tejidos blandos, son aquellas que incluyen toda la piel, los anexos cutáneos, el tejido celular subcutáneo, las fascias y el músculo estriado.
La pérdida de la integridad de la piel favorece la colonización del tejido celular subcutáneo y se convierte en un microambiente nutritivo y caliente para la colonización y proliferación de microorganismos. Las características de la herida (profundidad, localización, calidad de la piel, nivel de perfusión tisular) y del paciente (calidad de la respuesta inmune), definirán la progresión de la infección y el posterior pronóstico (P. G. Bowler, 2001).
Los agentes que se encuentran dentro de la flora microbiana normal de la piel son el *Corynebacterium spp*, estafilococos coagulasa negativo, *Micrococcus spp* y *Aerococcus spp*, entre otros. Por otra parte, se consideran patógenos los estreptococos betahemolíticos, *Staphylococcus aureus, Enterococcus spp, Bacilos anthracis, Pseudomonas aeruginosa* . Las bacterias anaerobias (*Bacterioides* spp, *Prevotella* spp, entre otros), también son

agentes etiológicos importantes porque se han relacionado con más del 38% de los casos (Cercenado E, 2006).

10.1 Definiciones

● **Colonización microbiana:** Corresponde al acceso y proliferación de los microorganismos sin causar infección.

● **Infección de la herida quirúrgica:** Se define como la infección que ocurre a partir de la contaminación bacteriana causada por un procedimiento quirúrgico.

● **Infección aguda de tejidos blandos:** Corresponde a las infecciones que afectan la piel, los anexos cutáneos, el tejido celular subcutáneo, la fascia y el musculo esquelético. U " f j f " (Cercenado E, 2006). Incluyen los abscesos cutáneos, heridas traumáticas y las infecciones necrotizantes.

● **Infecciones por mordeduras:** Infección que se genera en una herida producida por los dientes de un animal o de otra persona, a través de la maceración, perforación o laceración de los tejidos superficiales de los pacientes. Los agentes etiológicos más frecuentemente aislados, son *S. aureus, Peptostreptococcus sp, Bacterioides spp* y *Pasteurella multocida.*

● **Infección de una quemadura:** Se diagnóstica cuando aparecen cambios en la apariencia de la quemadura como áreas de decoloración local, edema en el margen de la herida o separación rápida de la escara.

● **Úlcera por presión:** Toda úlcera o lesión generada por una presión sostenida contra una superficie ósea o un plano firme, la cual puede causar fricción y cizallamiento responsable de la isquemia de los tejidos superficiales.

● **Medio de Cultivo:** Sustancia enriquecida con factores necesarios para el crecimiento de los microorganismos.

● **Especificidad:** Probabilidad que el cultivo no muestre crecimiento si la Infección está ausente.

● **Sensibilidad:** Probabilidad que el cultivo muestre crecimiento si la infección está presente.

10.2 Condiciones para la toma de la muestra.

Idealmente, se deben tomar muestras antes de iniciar la terapia antibiótica empírica, de aquellas lesiones que presenten signos clínicos de infección (cambio de apariencia, pobre cicatrización) (Cercenado E, 2006). En caso de infecciones por quemaduras, las muestras

deben ser obtenidas en los primeros días a semanas después de presentarse la lesión.

10.3 Instrucciones para la toma de muestras de piel y tejidos blandos

Se podría definir que las muestras de piel y de tejidos blandos puede ser de dos tipos: tejidos de la herida y fluidos de la herida.

10.3.1 HERIDAS CERRADAS

Está indicada cuando clínicamente se identifica la presencia de colecciones líquidas en piel intacta. También se encuentra indicada en casos de heridas quirúrgicas o colecciones que se encuentra adyacente a heridas abiertas cubiertas con detritus celulares.

10.3.2 HERIDAS ABIERTAS

La toma de muestras para heridas abiertas con hisopos, sin ser el método más recomendado, su facilidad y baja invasividad, lo hacen un método conveniente para la mayoría de heridas abiertas. Cualquier microorganismo que se encuentra en la profundidad de la herida, posiblemente también se encontrará en la superficie, lo que explica la buena correlación entre los cultivos cuantitativos de biopsias y los cultivos semicuantitativos obtenidos con hisopos.

10.3.2.1 Cuidados y Recomendaciones:

Limpiar la herida del borde hacia afuera con gasa impregnada con solución salina normal y alcohol isopropílico al 70%, con el fin de evitar contaminación de la muestra con la flora colonizante que no está realmente implicada en el proceso infeccioso.
Lavar la parte interna de la herida con solución salina abundante, sin presión. No usar antisépticos.

10.3.3. Técnica de Recolección:

- ✓ Aspire si es posible o pase un escobillón dentro de la herida. Tome la muestra con dos escobillones.
- ✓ Si emplea medio de transporte coloque uno en dicho medio y con el otro haga un extendido en lámina de vidrio.
- ✓ Si no tiene medio de transporte coloque los escobillones en un tubo estéril con tapa.

10.4 BIOPSIAS Y CURETAJES

La adquisición de tejido viable a través de una biopsia durante el desbridamiento inicial es el

método más utilizado para poder determinar la carga e invasividad de los microorganismos colonizadores en las infecciones de piel y tejidos blandos. El tejido obtenido asépticamente, es homogenizado, diluido y cultivado en medios selectivos y no selectivos para proveer información cualitativa y cuantitativa. En sospecha de infección de heridas por quemaduras, las muestras obtenidas por biopsia han sido las más utilizadas (Church D, 2006). La realización de este procedimiento debe ser realizado exclusivamente por personal médico entrenado.

10.5 Transporte de las muestras

Las muestras obtenidas en jeringas o frascos estériles deben ser cerradas herméticamente o garantizando que no se presenten goteos o escapes que permitan la pérdida o contaminación de las muestras; de igual manera, los hisopos deben ser introducidos en tubos estériles.
El transporte de las muestras hasta el laboratorio de microbiología, debe realizarse lo más pronto posible a temperatura ambiente, máximo dos horas después de tomadas las muestras (L Raka, 2012) (P. G. Bowler, 2001).

11.0 TRACTO GASTROINTESTINAL

Las infecciones del tracto gastrointestinal son el segundo tipo de enfermedades infecciosas más frecuentes, después de las enfermedades infecciosas del tracto respiratorio. Son múltiples las presentaciones que tienen las infecciones gastrointestinales al igual que los agentes etiológicos que los producen (Sociedad Española de Enfermedades Infecciosas y Microbiología Clínica, 2008).
Para el diagnóstico de enfermedad diarreica del tracto gastrointestinal, el tipo de muestra de elección es la deposición con características diarreicas. Las deposiciones compactas o los hisopados no deben ser utilizadas para el diagnóstico microbiológico.
En pacientes pediátricos con sospecha de tuberculosis, se encuentra indicado tomar muestras de contenido gástrico con el objetivo de realizar cultivos para *Mycobacterium tuberculosis.*

11.1 Definiciones

- **Medio de Cultivo:** Sustancia enriquecida con factores necesarios para el crecimiento de los microorganismos.

- **Diarrea disenteríforme:** tipo de diarrea que se acompaña de dolor abdominal, calambres, tenesmo y deposiciones diarreicas con presencia de moco y sangre.

● **Diarrea coleriforme:** tipo de diarrea aguda que se manifiesta con deposiciones acuosas y que se presenta como más de 3 evacuaciones líquidas, amarillentas, sin evidencia de sangre, acompañadas de vómito, fiebre, disminución del apetito e irritabilidad.

11.2 Condiciones para la toma de la muestra.

En pacientes con sospecha de infección por tuberculosis el paciente debe estar en ayunas y hospitalizado para poder realizar el procedimiento.
En pacientes con enfermedades diarreicas aguda, se recomienda tomar la muestra en los primeros días de la enfermedad*. Ante sospecha de parásitos, tomar tres muestras en días diferentes*. No se recomienda utilizar escobillones para la toma de la muestra.

11.3 Contenido gástrico para cultivo de Micobacterias

Este procedimiento se encuentra indicado en pacientes pediátricos que no tienen la capacidad de generar esputo. El contenido gástrico obtenido será utilizado para realizar la baciloscopia y para el cultivo de micobacterias.

11.4 Material fecal

El estudio microbiológico de la materia fecal está indicado en pacientes con enfermedad disentérica, fiebre, deposición con sangre, enfermedad nosocomial y enfermedad diarreica persistente. (E Baron, 2013; 57)
Tambien está indicado la utilización de muestras de materia fecal en pacientes con sospecha de infección por *Clostrodium difficile.*

11.4.1. Materiales

- ✓ Recipiente estéril de boca ancha
- ✓ Guantes no estériles

11.4.2. Técnica de Recolección

- ✓ Explicar al paciente en qué consiste el procedimiento y la cantidad necesaria de materia fecal necesaria para el análisis.
- ✓ Realizar higiene de manos.
- ✓ Recoger en un recipiente estéril de boca ancha, 5 mL de materia fecal diarreica o 2 a 4 gramos de materia fecal compacta.

- ✓ En pacientes pediátricos que utilizan pañal, no colocar este por la posición absorbente para de esta manera poder obtener la muestra con mayor facilidad.

11.5 Transporte de las muestras

Las muestras de heces deben ser transportadas dentro de las dos primeras horas de ser tomadas a temperatura ambiente y pueden ser conservadas hasta por 24 horas en refrigeración entre 2°C y 8°C. En sospecha de infección por *C. difficile*, las muestras para cultivo deben ser transportadas dentro de la primera hora de ser tomada y puede ser conservada hasta por 48 horas entre 2 y 8°C; Para la búsqueda de la citotoxina, las muestras se pueden conservar hasta 72 horas congelada a menos de 60°C. Para la búsqueda de parásitos, las muestras deben ser conservadas a temperatura ambiente. Ante la sospecha de Rotavirus, las muestras deben ser transportadas refrigeradas entre 2 y 8°C. (Sociedad Española de Enfermedades Infecciosas y Microbiología Clínica, 2003)
En el caso de muestras de jugo gástrico para búsqueda de micobacterias, las muestras deben ser transportadas en menos de 15 minutos al laboratorio a temperatura ambiente.

12.0 TRACTO GENITAL

Las lesiones del tracto genital, pueden tener múltiples etiologías. Los agentes que con mayor frecuencia se observan son *Chlamydia trachomatis, Neisseria gonorrhoeae*, *Treponema pallidum, virus del papiloma humano (VPH)* y virus del Herpes Genital (VHS) entre otros. (J Aznar, 2007)
Existen otras infecciones del tracto genitourinario que no se originan a partir de agentes transmitidos sexualmente; sin embargo, por sus características la toma de muestras microbiológicas de estas lesiones debe ser igual a la que se realiza para infecciones de transmisión sexual.
Las infecciones del tracto genital, estas se pueden dividir topográfica o sindromáticamente como (J Aznar, 2007):

- ✓ Ulceras Genitales: pueden ser dolorosas o no dolorosas. Los agentes más frecuentes son el VHS, *Treponema pallidum* y *Haemophilus ducreyi.*

- ✓ Uretritis y cervicitis: La más reconocida corresponde a la uretritis gonocócica originada por la *Neisseria gonorrhoeae.* Los agentes no gonocócicos corresponden a la *Chlamydia trachomatis, Ureaplasma urealyticum, Mycoplasma genitalium* y *Candida sp.* entre otros.

- ✓ Vulvovaginitis: En este caso, los agentes más frecuentes son *la Candida sp., Trichomona vaginalis, Gardnerella vaginalis, Mycoplasma hominis, Staphylococcus aureus y Streptococcus agalactiae.*

La flora bacteriana mixta que se encuentra en el tracto genital, exige que la toma de muestras, el procesamiento y la interpretación de la prueba sean cuidadosos con el único objetivo de ayudar al diagnóstico definitivo.

12.1 Condiciones para la toma de la muestra.

- Las muestras del tracto genital deben ser cultivadas tan pronto sea posible para garantizar la viabilidad de microorganismos de crecimiento difícil y evitar el sobrecrecimiento de bacterias contaminantes.
- La paciente no debe aplicar óvulos o duchas vaginales durante 24 horas antes de la toma de muestra
- Verificar que se haya realizado una limpieza adecuada de los genitales externos previamente, para eliminar secreciones contaminantes.

12.2. Lesiones o ulceras

Para la adecuada toma de muestras de este tipo de lesiones se deben tener en cuenta los posibles agentes etiológicos responsables de la infección; para garantizar la viabilidad de la muestra.
Algunas de las infecciones que ocasionan lesiones o ulceras son:

1. Sospecha de Infección por Herpes Virus
2. Sospecha de infección por *Haemophilus ducreyi*
3. Sospecha de infección con Sífilis
4. Sospecha de Donovanosis (granuloma inguinal)

12.3. Exudado uretral

Las uretritis tienen signos y síntomas similares en los hombres y las mujeres debido a que los agentes etiológicos son comunes en los dos. Las uretritis son factores de riesgo para desarrollar infecciones del tracto genital superior como son la Enfermedad Pélvica Inflamatoria, o en los hombres orquidoepididimitis y prostatitis.

12.3.1. Cuidados y Recomendaciones

- Verificar que se haya realizado una limpieza adecuada de los genitales externos previamente, para eliminar secreciones contaminantes.

- La muestra se debe tomar al menos una hora después de que el paciente haya orinado.

12.3.2. Técnica de recolección en hombres

- Colocar los elementos de protección personal y guantes no estériles.
- Si existe secreción abundante, con la mano libre tomar el pene y con la otra mano, recoger la secreción utilizando un hisopo.
- Si no se observa secreción, con la mano libre tomar el pene y con la otra mano, introducir cuidadosamente el hisopo de dacrón en la uretra cerca de 2 cm y realizar un movimiento de rotación.
- Introducir los hisopos recogidos en un medio de transporte que cumpla con condiciones de crecimiento de las posibles bacterias que se puedan encontrar en la muestra.

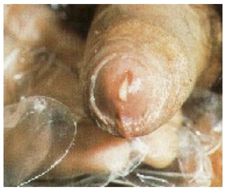

12.3.3, Técnica de recolección en mujeres

- Seleccionar una fuente de luz adecuada que permita realizar fácilmente el procedimiento.
- Ubicar a la paciente en posición ginecológica para la toma de la muestra.
- Colocar los elementos de protección personal y guantes no estériles.
- Si existe secreción abundante, con la mano libre separar los labios mayores y con la otra mano, recoger la secreción utilizando un hisopo de dacrón. Tomar dos o más hisopos.
- Si no se observa secreción, con la mano libre separar los labios mayores y con la otra mano, introducir cuidadosamente el hisopo cerca de 2 cm y realizar un movimiento de rotación. Tomar dos o más hisopos.
- En una lámina portaobjetos, realizar un extendido con uno de los hisopos para realizar coloración de Gram.
- Introducir los hisopos recogidos en un medio de transporte que cumpla con condiciones de crecimiento de las posibles bacterias que se puedan encontrar en la muestra.

12.4. Exudado vaginal

Exudado vaginal se puede encontrar en presencia de vaginitis, ocasionada por *Candida sp* y *Trichomona vaginalis.* De igual manera, la toma de muestra de exudado vaginal es fundamental para el diagnóstico microbiológico de la vaginosis bacteriana ocasionada por *Gardnerella vaginalis.* Ante sospecha de infecciones por *N. gonorrhoeae*, se debe realizar en conjunto con el frotis vaginal, la toma de muestra del exudado cervical. En el caso de mujeres gestantes, se debe realizar búsqueda activa de colonización por *Streptococcus agalactiae* con el objetivo de dar tratamiento profiláctico durante el parto para evitar la contaminación del neonato.

12.4.1. Técnica de Recolección

- Colocar los elementos de protección personal y guantes no estériles.
- Con extremo cuidado, introducir espéculo a través del introito vaginal sin utilizar gel lubricante. Abrir el espéculo para poder visualizar la cavidad vaginal y el cérvix uterino.
- Recoger con hisopos de dacrón el exudado vaginal depositado en el fondo de saco vaginal posterior. Tomar dos o más hisopos. Si la paciente se encuentra histerectomizada, tomar las muestras del fornix posterior.
- En sospecha de infecciones por *N. gonorrhoeae,* tomar inmediatamente muestra de exudado endocervical.
- Introducir los hisopos inmediatamente en los tubos con medio de transporte específico. (E Baron, 2013; 57)

12.5. Exudado cervical

La presencia de exudado cervical se puede encontrar en presencia de cervicitis, endometritis, y enfermedad pélvica Inflamatoria crónica. Ante sospecha de infección gonocócica, se debe tomar siempre muestra del exudado cervical en compañía de la muestra del exudado vaginal. En pacientes gestantes, este tipo de muestra debe ser realizado por personal médico experimentado. (Warren T, 2005) (J Aznar, 2007)

12.5. Técnica de Recolección

- Colocar los elementos de protección personal y guantes no estériles.
- Con extremo cuidado, introducir espéculo a través del introito vaginal sin utilizar gel lubricante. Abrir el espéculo para poder visualizar la cavidad vaginal y el cérvix uterino.
- Con un hisopo de dacrón estéril, retirar el exceso de moco de la superficie del cérvix. Posteriormente descartar el hisopo.

- Introducir un nuevo hisopo de dacrón en el canal endocervical y rotarlo 360° cuidadosamente. Repetir este procedimiento con un tercer hisopo de dacrón.
- Introducir los hisopos inmediatamente en los tubos con medio de transporte específico.

12.6. Exudado de la glándula de Bartolin

Se encuentra indicado en estadios tempranos de la enfermedad.

12.6.1. Técnica de Recolección

- Ubicar a la paciente en posición ginecológica para la toma de la muestra.
- Colocar los elementos de protección personal y guantes no estériles.
- Con extremo cuidado, introducir espéculo a través del introito vaginal sin utilizar gel lubricante. Abrir el espéculo para poder visualizar la cavidad vaginal y el cérvix uterino.
- Tomar un hisopo de dacrón estéril e introducirlo en la glándula de Bartolin, rotándolo contra las paredes. Repetir este procedimiento en tres ocasiones para obtener muestras para detección de *N. gonorrhoeae*, *C. trachomatis* y gérmenes comunes.
- Introducir los hisopos inmediatamente en los tubos con medio de transporte específico. (E Baron, 2013; 57)

12.7 Secreción prostática

La toma de muestras de secreción prostática se debe realizar posteriormente a realizar masaje prostático intrarrectal. Se encuentra indicada en pacientes con sospecha clínica de prostatitis infecciosa, epididimitis y/o orquitis. (L Raka, 2012) (E Baron, 2013; 57)

12.8. Semen

La toma de muestras de semen debe ser recogida en un frasco estéril de boca ancha después de estimulación local por parte del paciente por medio de la masturbación.

12.8.1. Técnica de Recolección

- Facilitar al paciente un espacio íntimo para la toma de la muestra.
- Indicar al paciente que se debe realizar higiene de manos, lavar el pene y el meato urinario antes de proceder a tomar la muestra.
- Indicar al paciente que debe tomar la muestra por medio de estimulación personal a través de la masturbación. La muestra se debe recoger en un frasco de boca amplia estéril.

- Cerrar completamente el frasco contenedor de la muestra.

12.9. Transporte de las muestras

El transporte de las muestras hasta el laboratorio debe realizarse lo más pronto posible, máximo hasta dos horas después de tomadas las muestras manteniéndolas a temperatura ambiente (L Raka, 2012). Si se han solicitado cultivos para *Chlamidia trachomatis* las muestras deben ser transportadas en el menor tiempo posible al laboratorio bajo refrigeración a 4°C (E Baron, 2013; 57).

13. TRACTO RECTAL

13.1. Hisopado rectal para tamizacion en paciente embarazada

En mujeres gestantes, el hisopado rectal se utiliza principalmente en la busqueda de *S. agalactiae* con el objetivo de administrar antibiótico profiláctico durante el parto, para disminuir el riesgo de colonización del recien nacido por este microorganismo.

- Tomar un culturette (hisopo) e introducirlo aproximadamente entre 1 a 2 cm en el esfinter anal
- Rotar el hisopo
- Introducir el hisopo en el medio de transporte y llevar al laboratorio para su procesamiento.

13.2. Hisopado rectal para hombres pre biopsia

En pacientes adultos quienes se vayan a realizar procedimientos quirúrgicos, como biopsias de próstata, se les realizará cultivo de hisopado rectal con el fin de evaluar la presencia de bacilos Gram negativos productores de Betalactamasas de espectro extendido (BLEE).

-Tomar un culturette (hisopo) e introducirlo aproximadamente entre 1 a 2 cm en el esfinter anal
- Rotar el hisopo
- Introducir el hisopo en el medio de transporte y llevar al laboratorio para su procesamiento.

13.3. Hisopado rectal para tamización de bacilos Gram negativos multirresistentes

Con el fin de realizar la búsqueda activa de pacientes portadores o colonizados por bacilos Gram negativos multirresistentes o productores de carbapenamasas, se tiene establecido realizar un hisopado rectal a todo paciente que ingrese por el servicio de urgencias o paciente que vaya a ingresar a Unidad de Cuidados Intensivos (UCI) para hacer la detección rápida de este tipo de microorganismos con el fin de realizar el aislamiento preventivo oportuno de estos pacientes y evitar posibles brotes dentro de nuestra institución.

-En el laboratorio se entregará el respectivo kit para la toma de muestra, que consta de 2 hisopos y 1 tubo de vidrio con 2 mL de solución salina.
-Introducir hisopo e introducirlo aproximadamente entre 1 a 2 cm en el esfinter anal
- Rotar el hisopo
- Introducir el hisopo en el tubo de vidrio con solucion salina y llevar al laboratorio para su procesamiento.

14. RECOLECCIÓN DE MUESTRAS PARA PATOLOGÍA

- Los especímenes quirúrgicos extraídos de salas de cirugía deben enviarse al laboratorio clínico en un recipiente hermético con formol al 10% para luego ser procesados por el histotecnólogo.
- Los líquidos deben enviarse en tubo estéril tapa rosca sin incluir formol ni otro tipo de solución para ser procesados en el área.
- Los quirúrgicos como biopsias renales o de piel que requieran procesamiento de microscopia electrónica debe realizarse el siguiente procedimiento:

  - ✓ Avisar con un día de anterioridad la toma de la biopsia al departamento y comunicarse con la secretaria de patología el día y hora del procedimiento.
  - ✓ El día del procedimiento avisar con una hora de anticipación y confirmar la realización del procedimiento a la secretaria.
  - ✓ Al tomar la biopsia se debe incluir el espécimen en solución salina y enviarlo de inmediato para su respectivo envío al laboratorio de referencia.

NOTA: Todos los residuos de toma de muestras fallidas se convierten en residuo anatomopatológico por lo cual, estos tubos se colocaran en un a bolsa pequeña de color rojo, identificada o rotulada como “Residuo anatomopatológico” con la fecha y hora para ser llevada al laboratorio en el recipiente de transporte de muestras y realizarle la segregación correspondiente en el departamento de laboratorio clínico.

14.1 TRANSPORTE DE LAS MUESTRAS AL LABORATORIO

Una vez tomadas deben ser llevadas en el menor tiempo posible al laboratorio y ser

entregadas en la ventanilla de recepción de muestras en el laboratorio clínico para su procesamiento.

- Si el paciente es ambulatorio la muestra es traída por el mismo o un familiar, debe entregar la respectiva orden con los datos del paciente, descripción de la muestra, firma y sello del medico que realizo la intervención.

El paciente y/o familiar factura y paga la biopsia en la caja del laboratorio clínico.

- Las biopsias que llegan de salas de cirugía son entregadas en el laboratorio por el camillero responsable, cada biopsia debe venir con su solicitud, identificada y rotulada. Son recibidas por la auxiliar del laboratorio, quien comprueba el aspecto de la muestra y que en el sistema aparezca la solicitud de los exámenes correspondientes y firma en constancia del recibido en el libro del servicio.

NOTA: Las ordenes son cargadas por la secretaria de patología de lunes a viernes, en horario no hábil las debe cargar facturación, posterior a esto las muestras son entregadas en el laboratorio .

15.0 BASES DE INTERPRETACIÓN DE LOS RESULTADOS DE LAS MUESTRAS DE LABORATORIO

PRUEBA	VALOR DE REFERENCIA	INTERPRETACIÓN CLÍNICA
Amilasa	Suero 30-110 U/L<br>Orina 32-641 U/L	Es un marcador para enfermedad pancreática aguda.<br>La actividad de la amilasa sérica aumenta en cuestión de horas (6 a 48 horas) en los pacientes con pancreatitis aguda; valores por encima de 500 U/l tienen valor diagnóstico. La amilasuria se eleva rápidamente, horas después de elevación en suero, y permanece elevada más tiempo que en el suero.
Amonio	9-30 mmol/L	La causa más frecuente de alteración de amonio es la enfermedad hepática severa y la enfermedad renal crónica o aguda. Controlar los niveles de amonio en sangre es de gran utilidad en el tratamiento del coma hepático
Bilirrubina total y directa	Total: 0,2-1,3 mg/dL<br>Directa:0,0-0,3 mg/dL<br>Indirecta: 0,0-0,1 mg/dL	Evaluación, clasificación y seguimiento de las ictericias tanto del adulto como neonatales. La hiperbilirrubinemia se clasifica en conjugada y no conjugada. La conjugada es hepática, colestásica, cirrosis biliar primaria, hepatitis, ictericia familiar y la pos hepática por obstrucción biliar por cálculos, cáncer o malformaciones de la vía biliar. La hiperbilirrubinemia no conjugada se clasifica en prehepática (estados hemolíticos y hematomas extensos)

		y hepática (síndrome de Gilbert y Crigler-Najjar e ictericia neonatal).
**Calcio iónico**	Suero 1,15-1,32 mmol/L	Hay hipercalcemia debida a tumores malignos e hiperparatirodismo primario y hay hipocalcemia por insuficiencia renal, hipoparatiroidismo, deficiencia de vitamina D e hiperparatiroidismo secundario.
Creatin Quinasa Total CK-CPCreatin [illegible]	Hombres 55-170 U/L<br>Mujeres 30-135 U/L<br>0-16 U/L	La CK MB/CPK ofrece un índice relativo que permite establecer la ocurrencia o no de infarto agudo del miocardio (IM). El ejercicio vigoroso como trotar o correr puede producir elevación de las isoenzimas a niveles similares a los del IM.
Cloro	Suero<br>98-107 mmol/L	Se presenta hipocloremia por pérdida de cloro en trastornos gastrointestinales, cetoacidosis diabética, exceso de mineralocorticoides y enfermedades renales; en tratamiento con ácido etacrínico, ACTH, corticoesteroides, diuréticos mercuriales y furosemida. Se presenta hipercloremia en acidosis metabólica por pérdida de bicarbonato, en hiperalimentación de sales de cloruro de amonio
Coproscópico	PH, Sangre oculta Leucocitos (células segmentadas): neutrófilos, linfocitos, eosinófilos. Azúcares reductores Glucosa Sacarosa Grasas Hongos Parásitos (troozoitos) Flora bacteriana	Útil para el diagnóstico de infestación parasitaria, ictericia obstructiva, diarrea, malabsorción, obstrucción rectosigmoidea, disentería, colitis ulcerativa y hemorragia gastrointestinal.
Creatinina	Suero<br>0-5 años 0,2-0,4 mg/dL<br>6-12 años 0,3-0,7 mg/dL<br>Hombres 0,66–1,25 mg/dL<br>Mujeres 0,52-1,04 mg/dL	La determinación de creatinina sérica se utiliza principalmente para evaluar la función renal, se eleva en daño renal en necrosis musculoesquelética, trauma, distrofia muscular progresiva, esclerosis lateral amiotrófica, amiotonía congénita, dermatomiositis, miastenia gravis,<br>ayuno prolongado, hipertiroidismo y acidosis diabética
Cuadro hemático completo	Leucocitos 4,5-11,0 (10*3 / mm3)<br>Eritrocitos 4,2-6,20 (10*6/ mm3)<br>Hemoglobina 14,0-17,0 g/dL<br>Hematocrito 36-54%<br>VCM 86-96 mm3<br>HCM 25-31 pg<br>CHCM 32-38 g/dL<br>Plaquetas 150-450 (10* 3 / mm3)<br>Eritrosedimentación:	Su mayor utilidad está en el diagnóstico diferencial de las anemias, en la evaluación y caracterización de lesiones proliferativas del sistema hematopoyético y en la evaluación de procesos infecciosos. El recuento plaquetario se determina en pacientes con sospecha de enfermedad hemorrágica, púrpura o petequias, prolongación del tiempo de sangría, leucemia, linfoma, quimioterapia, y para determinar la respuesta de los pacientes que están recibiendo transfusiones de plaquetas

	0-20 mm/hora.	

PRUEBA	VALOR DE REFERENCIA	INTERPRETACIÓN CLÍNICA
Nitrógeno Ureico (BUN)	Suero<br>Hombres 9-20 mg/dL<br>Mujeres 7-17 mg/dL	El BUN generalmente no se aumenta significativamente sino hasta que la filtración glomerular no se disminuye por lo menos en un 50%, por lo tanto no es un indicador precoz de daño renal.
Cultivo y antibiograma bacteriológico	Cultivo negativo para determinado germen patógeno	Aislamiento, identificación y antibiograma en los diferentes tipo de bacterias patógenas que están involucradas en infecciones humanas.
Deshidrogenasa láctica (LDH)	120-246 U/L	La LDH se mide principalmente para diagnosticar condiciones en las cuales hay daño tisular.
Dímero D	Menor de 550	Indicador de actividad del sistema fibrinolítico en casos de coagulación intravascular diseminada. Tamizaje de pacientes con sospecha de trombosis venosa profunda o tromboembolismo pulmonar. También se eleva en hemorragias, hematomas y en terapia trombolítica. Se presentan falsos positivos por factor reumatoideo o embarazo.}
Fibrinógeno (Factor I)	180-350 mg/dL	Sirve para determinar deficiencias congénitas o adquiridas de fibrinógenos y controlar severidad y tratamiento de la coagulación intravascular diseminada y de fenómenos de fibrinólisis. Se encuentra en niveles elevados en enfermedades inflamatorias agudas y crónicas, síndrome nefrótico, glomerulonefritis crónica, hipernefrona, hepatitis, hepatoma, cirrosis, embarazo, coagulación intravascular compensada y en terapia con estrógenos
Fosfatasa alcalina (FALK)	38-126 U/L<br>Niños 0-600 U/L	Se eleva en colestasis intra o extrahepática (hepatitis, cirrosis biliar, drogas hepatotóxicas, colédocolitiasis, carcinoma de la cabeza del páncreas, carcinoma hepático), en enfermedades óseas como osteitis, raquitismo, osteomalacia, fracturas en consolidación, tumores osteoblásticos e hiperparatiroidismo. También puede elevarse en Insuficiencia cardíaca, mononucleosis infecciosa, IM, perforación de intestino y septicemia.
Gama Glutamil Transferasa (gGT	Hombres 15-73 U/L<br>Mujeres 12-43 U/L	Enzima microsomal que se encuentra principalmente en hígado, páncreas y riñón. Está indicada en la evaluación de enfermedad hepática colestásica y lesión hepática inducida por abuso de drogas y alcohol. Se eleva junto con la FAL en enfermedades pancreáticas y hepáticas.
Glucosa	Ayunas:74-106 mg/dL<br>2 horas pos carga: < 140 mg/dL<br>orina: < 30 mg/dL<br>LCR: 40-70 mg/dL	La determinación en ayunas y la prueba de tolerancia a una carga de glucosa sirven para establecer el diagnóstico de Diabetes Mellitus y los trastornos de los carbohidratos. También sirve para controlar el tratamiento en los diabéticos y los pacientes con deshidratación, coma, hipoglicemia, insulinoma, acidosis y cetoacidosis

Lipasa	8-78 U/L	La lipasa sérica se eleva rápidamente en pacientes con lesiones en páncreas como pancreatitis aguda y recurrente, absceso o seudoquiste, trauma y carcinoma; también se eleva en obstrucción del colédoco, peritonitis, infarto y obstrucción intestinal, abscesos abdominales, falla renal y por acción de algunos fármacos como anticolinérgicos y opiáceos.
Magnesio	Suero: 1,6-2,3 mEq/L	Evaluación de alteraciones por malabsorción, pancreatitis, desórdenes de la depuración renal y control del tratamiento de la toxemia del embarazo. La hipomagnesemia se asocia con hipocalcemia, alcoholismo crónico, malnutrición, malabsorción, hemodiálisis crónica, drenaje gástrico prolongado, pancreatitis aguda, hipoparatiroidismo, glomérulonefritis hiperaldosteronismo y embarazo. La hipermagnesemia se presenta en pacientes con falla renal, deshidratación y enfermedad de Addison.
Proteínas totales y relación A/G	Totales: 6,3-8,2 g/dL<br>Albúmina: 3,5-5,0 g/dL<br>Globulinas: 2,8-3,2 g/dL	La concentración de proteínas séricas refleja principalmente la disminución de la síntesis hepática o la pérdida protéica por el riñón. La elevación de proteínas totales se encuentra en deshidratación, mieloma multiple, hiperglobulinemia, enfermedades granulomatosas, del colágeno y ciertas enfermedades tropicales. Hay disminución por dieta baja en proteínas, desnutrición, malabsorción, enfermedad hepática severa, quemaduras extensas, alcoholismo crónico, falla cardíaca, neoplasias, sobrehidratación, enfermedad renal.
Proteína C reactiva (PCR)	Adultos: 0 - 5 mg/l<br>Recien nacido: <0.6 mg/l<br>Niños de 4 dias a 1 mes: <1.6 mg/l	La PCR es la más sensible de fase aguda. Se eleva dos horas después de una lesión aguda, hace pico y empieza a disminuir a las 48 horas. Es un indicador de procesos inflamatorios más sensible que la sedimentación globular y el leucograma.

PRUEBA	VALOR DE REFERENCIA	INTERPRETACIÓN CLÍNICA
Potasio	Suero<br>3,5-5,1 mmol/L	Útil para detectar estados metabólicos donde se presentan variaciones en la concentración de potasio sérico y/o urinario tales como desbalance hidroelectrolítico, arritmias, debilidad muscular, encefalopatía hepática, falla renal. Hay medicamentos que elevan fisiológucamente el potasio tales como amiloride, captopril, ciclosporina, danasol, enalapril, epinefrina, heparina, histamina, nifedipina, espirinolactona y terbutalina. Se presenta disminución por diuréticos, anfotericina, cisplatino, corticoesteroides, insulina, teofilina, penicilina sódica y bicarbonato de sodio.
SARS-COV-2 (COVID-19) ANTICUERPO S	Negativo	Los resultados de esta prueba no pueden ser utilizados para diagnostico o exclusión de SARS COV-2.<br>Un resultado positivo requiere confirmación. Un resultado negativo no descarta la posibilidad de infección.<br>Es recomendado realizar un análisis exhaustivo de la condición clínica del paciente.

SARS-COV-2 (COVID-19) PCR-TIEMPO REAL (PCR-RT)	Negativo	Los resultados negativos no excluyen la infección por SARS-COV-2 y no deben usarse como la única base para el manejo del paciente. Los resultados negativos deben combinarse con observaciones clínicas, antecedentes del paciente e información epidemiológica.
SARS-COV-2<br>(COVID-19)<br>Filmarray RT<br>PCR BIOFIRE	Negativo (No detectado)	Los resultados negativos no excluyen la infección por SARS-COV-2 y no deben usarse como la única base para el manejo del paciente. Los resultados negativos deben combinarse con observaciones clínicas, antecedentes del paciente e información epidemiológica.
Sodio	Suero o plasma: 137-145 mmol/L	Se eleva en desequilibrio ácido-básico en pacientes descompensados hemodinámicamente.
Transaminasa Glutámicopirúvica<br>GPT (ALT)<br>Transaminasa Glutámico oxalacética<br>GOT (AST)	ALT<br>Hombres < 50 U/L<br>Mujeres <35 U/L<br><br>AST<br>Hombres 17-59 U/L<br>Mujeres 14-36 U/L	Se elevan significativamente en hepatitis y necrosis hepática de diferente etiología y en menor nivel en cirrosis, ictericia obstructiva, carcinoma metastásico, congestión hepática y colestasis intrahepática. Puede haber elevación ligera en IAM y pancreatitis aguda, aunque la elevación de la AST en el IAM es más significativa.
Tiempo de Protombina (PT) e INR (Radio Internacional Normalizado)	PT 12,8-15,8 segundos<br>INR2,0-3,0 para una dosis estándar de cumarínicos.<br>2,5-3,5 para una dosis alta de cumarínicos.	El PT evalúa trastornos de la coagulación que comprometen el sistema extrínseco y la vía común de la coagulación. También se prolonga durante el tratamiento a largo plazo con heparina, deficiencia de vitamina K, enfermedad hepática, hipofibronogenia y coagulación intravascular diseminada (CID). Su mayor utilidad radica en el control de la anticoagulación oral con cumarínicos.
Tiempo Parcial de Tromboplastina (PTT)	24,6-31,2 segundos	Prueba sensible a todos los factores que intervienen en el sistema intrínseco de la coagulación, especialmente a deficiencias funcionales de factor VIII, IX, XI y XII. Es de utilidad para controlar la efectividad de la terapia con heparina
Troponina I cuantitativa	Hombres: 0-34,1 pg/ml<br>Mujeres: 0-15,5 pg/ml	El IM agudo se diagnostica generalmente por dolor precordial, cambios electrocardiográficos y elevación de las enzimas cardíacas. En las últimas dos décadas la más usada ha sido la izoenzima MB, sin embargo, su especificidad no es tan buena como la de TnI puesto que la CKMB también se eleva en enfermedad aguda y crónica

Uroanálisis	Aspecto<br>Color : amarillo transparente<br>pH: 5-7<br>Densidad: 1001-1030<br>Proteína, bilirrubina, cetonas, nitritos, sangre y glucosa negativos.<br>Urobilinógeno: 0,2-1,0 U Erlich/dL<br>Leucocitos: 1-2 por campo<br>Células epiteliales: escasas por campo<br>Cilindros hialinos: ocasionales por campo<br>Bacterias: raras<br>Eritrocitos: 0,2 por campo. Cristales: ocasionales (fosfatos) moco	El uroanálisis es de ayuda en el diagnóstico, evolución y tratamiento de infección urinaria y enfermedades renales
Opiáceos, anfetaminas y cocaína	Negativo	Detección de drogas de abuso.
**PRUEBA**	**VALOR DE REFERENCIA**	**INTERPRETACIÓN CLÍNICA**
Benzodiacepinas	Negativo < 12 ng/dL	Prueba de tamizaje o presuntiva para detectar presencia de benzodiacepinas y sus metabolitos en suero.
Barbitúricos	Negativo < 60 ng/mL	Prueba de tamizaje o presuntiva para detectar presencia de barbitúricos y sus metabolitos en orina; su presencia indica uso durante tres días previos a la determinación
Antidepresivos tricíclicos	Rangos terapéuticos (ug/l):Amitriptilina 120-250<br>Nortriptilina 50-150<br>Desipramina 75-160<br>Inipramina 150-250	Seguimiento del tratamiento para evitar niveles tóxicos y en los casos donde se sospeche su ingestión como intento de suicidio.
Alcohol etílico (etanol	Negativo < 10 mg/dL	Determina la concentración de alcohol etílico en sangre para hacer la correlación con el grado de intoxicación o con ingestión reciente.
Alcohol metílico (metanol)	Negativo	Por ser un método cualitativo no es posible determinar el grado de intoxicación y para esto es necesario la evaluación clínica del paciente. Este tipo de intoxicación es una verdadera urgencia toxicológica por lo tanto el resultado debe ser informado rápidamente.

Ácido valproico	Rango terapéutico: 50-100 ug/mL	Seguimiento del tratamiento debido a grandes diferencias individuales en las dosis requeridas para alcanzar la terapia efectiva y con el fin de evitar efectos tóxicos de la droga por sobredosis.
Amikacina Carbamazepina	Pico 20-25 ug/mg Valle < 5 ug/mg 4-10 ug/mg	Controlar los niveles sanguíneos del antibiótico para eficiencia del tratamiento y evitar toxicidad. Control de los niveles terapéuticos
Litio	Rango terapéutico: 1,0-1,2 mmol/L Rango potencialmente tóxico: >1,5 mmol/L Toxicidad severa: > 2,5 mmol/L	Control de los niveles terapéuticos y para evitar toxicidad por sobredosis.
Digoxina	Rango terapéutico: 0,6-2,0 ng/mL	Determinar niveles terapéuticos y evitar toxicidad por exceso del medicamento; rangos por encima de 2,7 mg/mL deben ser de alerta para disminuir la dosis.
Fenitoína	Rango terapéutico: Niños >3 meses y adultos:10-20 ug/mL Niños <03 meses: 6-14 ug/mL Fenitoína libre: 1-2 ug/mL	Determinación de niveles terapéuticos.
Fenobarbital	Rango terapéutico: 10-30 ug/mL	Determinación de niveles terapéuticos
Tacrolimus (FK-506)	Rango terapéutico: 5-20 ng/mL	Tratamiento y control del rechazo pos trasplante de órganos.
Vancomicina	Rango terapéutico: Pico 30-40 mg/mL Valle 5-10 mg/mL	Determinación de niveles terapéuticos.

16.0. PROTOCOLO DE TOMA DE MUESTRAS

16.1 PROTOCOLO DE TOMA DE MUESTRAS PISOS

ACTIVIDAD	DESCRIPCIÓN	REGISTROS	RESPONSABLE
Solicitud de los exámenes de	1- El médico debe solicitar los exámenes directamente en el sistema por historia clínica e indicar la hora de la toma de la muestra	Historia clínica	Medico tratante de piso

laboratorio servicios de hospitalizació n general	2- Realizar la solicitud de exámenes teniendo en cuenta las siguientes recomendaciones:<br>- Digitar el código CUPS correspondiente a cada examen.<br>**- Indicar con claridad la fecha y hora en que se requiere el examen.**<br>**- Grabar de forma independiente órdenes que se requieren para días u horas diferentes.**<br>- Hacer las observaciones correspondientes al tipo de muestra (cultivos) y número de hemocultivos que requiere para que así mismo queden grabados.	Historias Clínicas	Medico tratante
IDENTIFICA R MUESTRAS PENDIENTE S POR TOMAR	Una vez ingresada la orden, queda en un listado preliminar de paciente pendientes por tomar.	Historia clínica. Software laboratorio	Facturador Auxiliar de laboratorio
TRASPASO DE ORDENES	1- Traspasar las órdenes que aparezcan en el sistema, teniendo en cuenta la fecha y hora indicada para la toma de muestra, en caso de no tener ninguna indicación al respecto se traspasa la orden y la toma queda para la ronda siguiente.	Software Laboratorio	Bacteriólog a Auxiliar de laboratorio
	2- TRASPASO:<br>En la pantalla de plano intermedio revisar las ordenes pendientes por tomar<br>- Resaltar la orden que necesita traspasar y dar doble click.<br>- Automáticamente se imprimen los sticker.<br>- - Revisar en el sistema los ESTUDIOS solicitados y confirmar que no tenga exámenes pendientes, o solicitados por duplicado. Si tiene exámenes pendientes confirmar en la sección correspondiente si es necesario la toma de la muestra, imprimir los sticker para que sean tomados.<br>- Verificar con el listado de pacientes, el número de habitación.<br>- Organizar por pisos las órdenes que	Software Laboratorio	Bacteriólog a Auxiliar de laboratorio

	correspondan a esa ronda.		
UBICACIÓN [illegible]	3- Ubicar al paciente en el piso correspondiente, verificar los nombres – apellidos y cédula del [illegible]	Vale de digitación o [illegible]	Bacterióloga
TOMA DE LA MUESTRA	Antes de iniciar el proceso, realizar el lavado de manos y utilizar guantes nuevos.<br><br>Realizar identificación cruzada con el paciente, identificador visual en la habitación del paciente, manilla o personal de enfermería.<br><br>Explicar al paciente el procedimiento que se va a realizar.<br><br>Utilizar los elementos de protección personal, según el tipo de paciente a tomar (Aislamientos preventivos, trasplantes, riesgo de infección aérea o por contacto, etc.) y según el tipo de examen (hemocultivos).<br><br>Ubicar el brazo y sitio de venopunción. Recuerde no tomar muestras en el brazo que tenga líquidos, fistulas, heridas o lesiones.<br><br>Poner el torniquete de manera que la presión no sea excesiva, pues esto interfiere en las pruebas de laboratorio.<br><br>Realizar la asepsia del sitio de la venopunción con un algodón húmedo con alcohol, limpiar de forma	Vale de digitación o stickers	Bacterióloga
TOMA DE MUESTRAS PENDIENTES	En caso de venas difíciles, pacientes en procedimientos o cambios de habitación, no pueden quedar pendientes por tomar hasta la ronda siguiente, se debe pedir ayuda a una Bacterióloga del mismo turno.<br><br>En casos extremos en los que no se pueda tomar una muestra por vena difícil, avisar a la jefe de piso		

	en busca de ayuda.<br><br>**La retoma de muestras para confirmar un resultado debe ser realizada por el personal de enfermería en el menor tiempo, luego de solicitada la muestra por el laboratorio.**		
IDENTIFICACIÓN DE LAS MUESTRAS	Marcar frente al paciente cada tubo con las iniciales del nombre completo del paciente, dejarlas visible para poder verificar la información con los sticker correspondientes	sticker	Bacterióloga
	Las muestras que llegan tomadas al laboratorio se deben registrar en el cuaderno de entrega de muestras, anotar el tipo de muestra, nombre completo del paciente, de la persona que entrega y de la persona que recibe, hora y servicio.	Cuaderno entrega de muestras	Camillero pisos<br><br>Auxiliar de laboratorio
TRANSPORTE DE MUESTRAS AL LABORATORIO	Por seguridad todas las muestras deben manejarse como potencialmente infecciosas, aún cuando no sean evidentes los riesgos de infección.<br><br>Colocar las muestras en la lonchera de toma de muestras, y llevarlas al laboratorio central en el menor tiempo posible. Tener en cuenta que los tubos no se deben acostar durante el trayecto.<br><br>Las muestras se deben llevar al laboratorio una vez terminada la ronda.<br><br>Verificar que sean entregadas todas las muestras. En caso de un derrame limpiar inmediatamente con el desinfectante adecuado.(Amonio Cuaternario)<br><br>Entregar las muestras a la auxiliar del laboratorio.	Muestras tomadas	Auxiliar de laboratorio<br><br>Bacterióloga
REGISTRO DE ENTRADA Y DISTRIBUCION DE MUESTRAS	Una vez recibidas las muestras la auxiliar debe hacer el registro de las muestras que ingresaron: ORDENES- RECEPCIÓN DE MUESTRAS – PASE EL CÓDIGO DE BARRAS Y CONFIRME EL INGRESO.<br>Este proceso también aplica para las muestras que llegan tomadas de piso.<br>Distribuir las muestras en las diferentes secciones del laboratorio.	MUESTRAS	AUXILIAR DE LABORATORIO

TOMA DE GLUCOMETRIAS	Solicitud de exámenes por sistema.<br>La toma de glucometría está a cargo del personal de enfermería.	Solicitud de examen por sistema	Enfermera Jefe
TOMA DE CULTIVOS	CULTIVOS AEROBIOS, ANAEROBIOS, HONGOS Y MICOBACTERIAS:<br><br>Heridas de piel o tejidos blandos, muestras del tracto respiratorio y gastrointestinal, catéteres, urocultivos, deben ser tomadas por el personal de enfermería. Y luego ser llevadas por el camillero al laboratorio en el menor tiempo posible.	Solicitud de exámenes por sistema.	Personal de enfermería<br>Camillero<br>Auxiliar de laboratorio<br>Médico
	CULTIVO Y FROTIS DE FLUJO VAGINAL: La toma de estas muestras depende del laboratorio clínico. Las pacientes que se puedan movilizar deben ser llevadas al laboratorio en el momento que se solicite.<br>A las pacientes que no puedan ser trasladadas se les toma directamente en piso por el personal de enfermeria.		laboratorio clínico
	LIQUIDOS: Deben ser tomados por el médico tratante o especialista. Se debe enviar cantidad suficiente según el número de exámenes que se requieran. Los líquidos pleurales, sinoviales, articulares, peritoneales deben venir en tubo lila estéril para evitar que se coagulen. Los LCR pueden venir en tubo estéril tapa de rosca.	Manual de toma de muestras micro biología	Medico [illegible]
TOMA DE HEMOCULTIVOS	La toma de hemocultivos está a cargo del laboratorio clínico. Excepto en la rotonda de la UCI donde el manejo de los pacientes es exclusivo de enfermería.<br><br>Verificar en el sistema el número de hemocultivos solicitados<br><br>Los hemocultivos deben ser tomados según	Orden por sistema.	Bacteriología<br>Personal de Enfermería

	PROTOCOLO ANEXO No 2, por personal del laboratorio<br>Los hemocultivos que deriven de catéter deben ser tomados en piso por el personal de enfermería. Junto con un cultivo de punta de catéter y un hemocultivo periférico.		

16.2. PROTOCOLO DE TOMA DE MUESTRAS UNIDADES DE CUIDADO INTENSIVO

ACTIVIDAD	DESCRIPCION	REGISTROS	RESPONSABLE
RESPONSABILIDAD TOMA DE LA MUESTRA	En la UCI la toma de las muestras se realizará de la siguiente manera:<br><br>**LATERAL:** La toma de muestras de sangre la debe hacer el personal de laboratorio clínico. Cuando se requiera iniciar un esquema antibiótico, el primer hemocultivo lo debe tomar enfermería. Las secreciones, frotis y demás cultivos deben ser tomados en piso y enviar las muestras al laboratorio en el tiempo indicado.<br><br>**ROTONDA:** Por el alto riesgo de contaminación que manejan los pacientes de la lateral se acordó que estos pacientes serán manejados por el personal de enfermería en su totalidad, incluyendo la toma de hemocultivos y cultivos en general.	Acta de reunión con departamento de enfermería	Jefe departamento de enfermería<br><br>Jefe departamento de laboratorio clínico
TOMA DE MUESTRAS POR CATETER	Cuando se tomen muestras por la línea arterial se debe hacer una purga de 20 c.c.<br><br>En la rotonda cuando sea estrictamente necesaria la toma de muestras por la línea arterial, lo debe realizar el personal de enfermería.<br><br>El llenado de los tubos se debe realizar de la siguiente manera:	Protocolo toma de muestras UCI	Enfermería

	1- el tubo azul<br>2- el tubo amarillo<br>3- tubo verde<br>4- tubo lila<br>5- tubo gris<br><br>Una vez se realice el llenado hay que mezclar por inversión y asegurarse de tapar muy bien el tubo.		
TOMA DE LA MUESTRA POR VENOPUNCIÓN	Antes de iniciar el proceso, se debe realizar lavado de manos y utilizar guantes nuevos.<br><br>Hacer identificación cruzada del paciente ...<br><br>Explicar al paciente el procedimiento que se va a realizar siempre que sea posible y el paciente este consiente.<br><br>Utilizar los elementos de protección personal, según el tipo de paciente a tomar (Aislamientos preventivos, trasplantes, riesgo de infección aérea o por contacto, etc.) y según el tipo de examen (hemocultivos).<br><br>Ubicar el brazo y sitio de venopunción. Recuerde no tomar muestras en el brazo que tenga líquidos, fistulas, heridas o lesiones.<br><br>Poner el torniquete de manera que la presión no sea excesiva, pues esto interfiere en las pruebas de laboratorio.<br><br>[illegible]	Solicitud del examen por sistema	Bacterióloga Enfermería
IDENTIFICACIÓN DEL ORIGEN DE LA MUESTRA	Una vez tomada la muestra se debe marcar en el sticker el origen de la muestra:<br><br>✓ muestra tomada por catéter<br>✓ muestra tomada por venopunción	Protocolo toma de muestras UCI	Bacterióloga Enfermería
IDENTIFICACIÓN DE LAS MUESTRAS	Las muestras de la unidad de cuidado intensivo deben ser identificadas con el sticker del paciente.	Sticker paciente	Bacterióloga Enfermería
TRANSPORT	Las muestras deben ser enviadas al laboratorio en	Cuaderno	Camillero

E DE MUESTRAS AL LABORATORIO	el menor tiempo posible y verificar que queden registradas en el cuaderno de ingreso de muestras estipulando la persona que recibe y la hora.	de registro de ingreso de muestras	Auxiliar de laboratorio
TOMA DE HEMOCULTIVOS	La toma de hemocultivos en la lateral está a cargo del laboratorio clínico. Excepto en larotonda de la UCI donde el manejo de los pacientes es exclusivo de enfermería.<br><br>Los hemocultivos deben ser tomados según el protocolo institucional.<br><br>Los hemocultivos que deriven de catéter deben ser tomados en piso por el personal de enfermería. Junto con un cultivo de punta de catéter y un hemocultívo periférico.	Orden por sistema.	Bacteriología Personal de Enfermería

16.3. PROTOCOLO TOMA DE MUESTRAS URGENCIAS

ACTIVIDAD	DESCRIPCIÓN	REGISTROS	RESPONSABLE
SOLICITUD DE LOS EXÁMENES DE LABORATORIO	El médico tratante debe solicitar los exámenes directamente por sistema en el programa de historia clínica	Historia Clínica	Medico servicio de urgencias
IDENTIFICAR MUESTRAS PENDIENTES POR TOMAR	Una vez ingresada la orden, queda en un listado preliminar de paciente pendientes por tomar.	Historia clínica. Software laboratorio	Auxiliar de laboratorio
TRASPASO DE ORDENES	Traspasar las órdenes que aparezcan en el sistema, teniendo en cuenta la hora indicada para la toma de muestra.	Software Laboratorio	Auxiliar de laboratorio
	2- TRASPASO:	Software	

	En la pantalla de plano intermedio revisar las ordenes pendientes por tomar<br>- Resaltar la orden que necesita traspasar y dar doble click.<br>- Automáticamente se imprimen los sticker.<br>- Revisar en el sistema los ESTUDIOS solicitados y confirmar que no tenga exámenes pendientes, o solicitados por duplicado. Si tiene exámenes pendientes<br>- Verificar con el listado de pacientes, el número de habitación.<br>- Organizar por pisos las órdenes que correspondan a esa ronda.	Laboratorio	Auxiliar de laboratorio
TOMA DE LA MUESTRA	- Antes de iniciar el proceso, realizar el lavado de manos y utilizar guantes nuevos.<br>- Realizar identificación cruzada con el paciente, manilla o personal de enfermería.<br>- Reconfirmar nombres completos y numero de cedula,<br>xplicar al paciente el procedimiento que se va a realizar.<br>- Utilizar los elementos de protección personal.<br>- Ubicar el brazo y sitio de venopunción. Recuerde no tomar muestras en el brazo que tenga líquidos, fistulas, heridas o lesiones.<br>- Poner el torniquete de manera que la presión no sea excesiva, pues esto interfiere en las pruebas de laboratorio.<br>ealizar la asepsia del sitio de la venopunción con un algodón húmedo con alcohol, limpiar de forma circular de adentro hacia afuera y esperar que se seque, NO SOPLE.<br><br>En cuanto empiece a fluir la sangre soltar el torniquete, tomar los tubos necesarios según la orden, tener en cuenta el orden de toma y volumen requerido por tubo, mezclar suavemente por inversión el número de veces indicado para cada tubo.		Auxiliar de laboratorio

	Retirar la aguja de la vena ,soltar el torniquete, colocar un algodón limpio y seco, hacer presión suave con el brazo estirado y la mano abierta. Colocar una cinta adhesiva para sostener el algodón. Si es un paciente anticoagulado asegúrese que se haga presión durante 5 minutos<br><br>Tener en cuenta las normas de bioseguridad para el descarte de agujas , algodones y residuos en general.<br>Decir al paciente en cuanto tiempo estará su resultado.		
TOMA DE MUESTRAS PENDIENTES	En caso de venas difíciles, pacientes pediatricos , se debe pedir ayuda a una Enfermera Jefe		
IDENTIFICACIÓN DE LAS MUESTRAS	Marcar frente al paciente cada tubo con las iniciales del nombre completo del paciente, dejarlas visible para poder verificar la información con los sticker correspondientes . Utilizar un resaltador para indicar que es una muestra a la que se le debe dar prioridad.	sticker	Auxiliar de laboratorio
TRANSPORTE DE MUESTRAS AL LABORATORIO	Por seguridad todas las muestras deben manejarse como potencialmente infecciosas, aún cuando no sean evidentes los riesgos de infección.<br><br>Colocar las muestras en el recipiente de transporte: capsula de envió por tubo neumático o transporte manual de muestras, y enviarlas o llevarlas al laboratorio central en el menor tiempo posible. Tener en cuenta que los tubos no se deben acostar durante el trayecto.<br><br>Para el envió de muestras de orina o materia fecal por el tubo neumático, recuerde hacer el doble embalaje en bolsa ziplot y frasco de cierre hermético para colocarlas en la capsula.<br><br>Verificar que sean entregadas todas las muestras.<br><br>En caso de un derrame limpiar inmediatamente con el desinfectante adecuado.(Amonio	Muestras tomadas	Auxiliar de laboratorio

	Cuaternario)<br>Ver instructivo /limpieza y desinfección.<br><br>Cuando el transporte sea personal entregar las muestras a la auxiliar del laboratorio.		
REGISTRO DE ENTRADA Y DISTRIBUCIO N DE MUESTRAS	Una vez recibidas las muestras la auxiliar debe hacer el registro de las muestras que ingresaron: ORDENES- RECEPCION DE MUESTRAS – PASE EL CODIGO DE BARRAS Y CONFIRME EL INGRESO.<br>**Con el fin de dar prioridad a las muestras de urgencias , resalte el numero de orden con cualquier color.**<br><br>Toda muestra diferente a sangre (orinas, materia fecal..) que se toma en urgencias se debe registrar en el libro de entrega de muestras de urgencias con los datos solicitados<br><br>Distribuir las muestras en las diferentes secciones del laboratorio.	MUESTRA S	AUXILIAR DE LABORATORI O CENTRAL

16.4. TOMA DE MUESTRAS ORINAS Y COPROLÓGICOS SERVICIO DE URGENCIAS

ACTIVIDAD	DESCRIPCIÓN	REGISTROS	RESPONSABLE
Entrega de recipientes	La auxiliar de enfermería debe entregar al paciente el recipiente indicado, debidamente marcado con nombres y apellidos – N° de identificación e ingreso y las gasas para realizar el aseo previo.	Orden facturada Sticker	Auxiliar de laboratorio
Toma de muestras por micción espontanea	La auxiliar debe explicar al paciente ambulatorio el sitio y la manera indicada para la recolección de la muestra		Auxiliar de laboratorio
Toma de muestras pacientes con sonda	La enfermera Jefe está a cargo de la toma de muestras de orina por sonda y debe avisar a la auxiliar de laboratorio.		Enfermera Jefe Auxiliar de laboratorio

16.5. MUESTRAS NO CONFORMES

El laboratorio clínico bajo el cumplimiento de los requisitos preanaliticos que deben cumplir la muestras considera como muestras no conformes: muestras hemolizadas, hemodiluidas, coaguladas, insuficientes, mal tomadas (tubo o recipiente inadecuado, no se tiene en cuenta el volumen de llenado del tubo), mal identificadas tanto por nombre como por tipo de muestra.

En referencia a las muestras no conformes el laboratorio realizá principalmente tres acciones:

Rechazo: La muestra se reserva en custodia y se solicita nueva muestra
Concesión: en caso de muestras de difícil retoma (LCR, neonatos).
Reproceso: se solicita nueva muestra para confirmar reporte.

16.6. EXÁMENES DE LABORATORIO GENERAL Y MUESTRAS REQUERIDAS

EXAMEN	MUESTRA	EXAMEN	MUESTRA
ANTICOAGULANTE LUPICO	TUBO AZUL	ACIDO FOLICO	TUBO AMARILLO
AGREGACION PLAQUETARIA	5 TUBOS AZULES	ACIDO VALPROICO	TUBO AMARILLO
CUADRO HEMATICO	TUBO LILA	ANTI CHAGAS	TUBO AMARILLO
CRIOAGLUTININAS	TUBO AMARILLO	ANTI DNA	TUBO AMARILLO
DIMERO D	TUBO AZUL	ANTI NUCLEARES	T UBO AMARILLO
ERITROSEDIMENTACION	TUBO LILA	ANTI TOXO G	TUBO AMARILLO
EOSINOFILOS EN MOCO NASAL	FROTIS MOCO NASAL	ANTI TOXO M	TUBO AMARILLO
FACTORES DE COAGULACION	TUBO AZUL	ANTI TROMBINA III	TUBO AZUL
FIBRINOGENO	TUBO AZUL	ANTIGENOS EXTRACTABLES	TUBO AMARILLO

FROTIS DE SANGRE PERIFERICA	EXT. DE SANGRE PERIFERICA	ANTIGENO PROSTATICO	TUBO AMARILLO
HEMOCLASIFICACION	TUBO LILA	ASTOS	TUBO AMARILLO
HEMOPARASITOS	GOTA GRUESA + FSP	BETA HCG	TUBO AMARILLO
P.D.F.	TUBO AMARILLO	BNP	TUBO LILA
PROTEINA S Y C COAGULACION	TUBO AZUL	CARBAMAZEPINA	TUBO AMARILLO
PT	TUBO AZUL	CARDIOLIPINA IG G	TUBO AMARILLO
PTT	TUBO AZUL	CARDIOLIPINA IG M	TUBO AMARILLO
PLAQUETAS	TUBO LILA	C 3	TUBO AMARILLO
RESISTENCIA A LA PROTEINA C	TUBO AZUL	C4	TUBO AMARILLO
RETICULOCITOS	TUBO LILA	CICLOSPORINA	TUBO LILA
CELULAS LE	TUBO LILA	CITOMEGALOVIRU S IG G	TUBO AMARILLO
AMILASA	TUBO AMARILLO	CITOMEGALOVIRU S IG M	TUBO AMARILLO
ACIDO LACTICO	TUBO GRIS	COOMBS DIRECTO	TUBO LILA
ACIDO URICO	TUBO AMARILLO	COOMBS INDIRECTO	TUBO LILA
BILIRRUBINAS T/D	TUBO AMARILLO	DIGOXINA	TUBO AMARILLO
CALCIO	TUBO AMARILLO	DROGAS DE ABUSO	ORINA PARCIAL
CLORO	TUBO AMARILLO	ETANOL	TUBO GRIS
COLESTEROLES T, HDL, LDL	TUBO AMARILLO	FENITOINA	TUBO AMARILLO
CPK	TUBO AMARILLO	FENOBARBITAL	TUBO AMARILLO
CPK MB	TUBO	FERRITINA	TUBO AMARILLO

	AMARILLO		
CREATININA	TUBO AMARILLO	HEPATITIS (TODAS)	TUBO AMARILLO
FOSFATASA ACIDA	TUBO AMARILLO	HIV	TUBO AMARILLO
FOSFORO	TUBO AMARILLO	INMUNOGLOBULINAS G, M, A, E	TUBO AMARILLO
GGTP	TUBO AMARILLO	PROLACTINA	TUBO AMARILLO
GLICEMIA	TUBO AMARILLO	RUBEOLA IG G - IG M	TUBO AMARILLO
HEMOGLOBINA GLICOSILADA	TUBO LILA	SEROAGLUTINACIONES	TUBO AMARILLO
HEMOGLOBINA LIBRE	TUBO AZUL	T3 – T4 – T3 Y T4 LIBRE	TUBO AMARILLO
HIERRO	TUBO AMARILLO	TEST RA	TUBO AMARILLO
LDH	TUBO AMARILLO	TRANSFERRINA	TUBO AMARILLO
MAGNESIO	TUBO AMARILLO	TSH	TUBO AMARILLO
NITROGENO UREICO	TUBO AMARILLO	VDRL	TUBO AMARILLO
PCR ULTRASENSIBLE	TUBO AMARILLO	VITAMINA B12	TUBO AMARILLO
POTASIO	TUBO AMARILLO	ACIDO VANILMANDELICO	ORINA 24 H + 10 CC HCL
PROCALCITONINA	TUBO AMARILLO	ACIDO 5 HIDROXI	ORINA 24 H + 10 CC HCL
PROTEINAS	TUBO AMARILLO	METANEFRINAS - CATECOLAMINAS	ORINA 24 H + 10 CC HCL
SODIO	TUBO AMARILLO	ACTH	TUBO AMARILLO
TGO	TUBO AMARILLO	ADENOSIN DE AMINASA	SUERO O LIQUIDO
TGP	TUBO	ALDOLASA	TUBO AMARILLO

	AMARILLO		
TRIGLICERIDOS	TUBO AMARILLO	FOSFOLIPIDOS IG M	TUBO AMARILLO
ALFAFETOPROTEINAS	TUBO AMARILLO	FSH	TUBO AMARILLO
ALDOSTERONA	TUBO AMARILLO	FTA	TUBO AMARILLO
AMONIO	TUBO LILA	GASTRINA	TUBO AMARILLO
TROPONINA	TUBO VERDE	HAPTOGLOBINA	TUBO AMARILLO
ANTITIROIDEOS	TUBO AMARILLO	HELICOBACTER PILORY	TUBO AMARILLO
ANTI MICROSOMALES	TUBO AMARILLO	HERPES I Y II IG G, IG M	TUBO AMARILLO
ANTITIROGLOBULINA	TUBO AMARILLO	17 HIDROXIPROGESTERONA	TUBO AMARILLO
ANTIG CARCINOEMBRIONARIO	TUBO AMARILLO	HOMOCISTEINA	TUBO AMARILLO
ANTIG CANCER 125	TUBO AMARILLO	INSULINA	TUBO AMARILLO
ANTIG CANCER 15,3	TUBO AMARILLO	MICROALBUMINURIA	ORINA PARCIAL
ANTIG CANCER 19,9	TUBO AMARILLO	MITOCONDRIA	TUBO AMARILLO
ANTIG PROSTATICO LIBRE	TUBO AMARILLO	MUSCULO LISO	TUBO AMARILLO
CALCIO IONICO	TUBO AMARILLO	OSTEOCALCINA	TUBO VERDE
CAPAC. COMB. HIERRO	TUBO AMARILLO	P 65 PARA CITOMEGALOVIRUS	TUBO LILA
CAROTENOS	TUBO AMARILLO	**PARATOHORMONA**	**Tubo amarillo**
CATECOLAMINAS	TUBO AMARILLO	PIRINLIKS	ORINA 3 HORAS

CITOPLASMATICOS	TUBO AMARILLO	PROGESTERONA	TUBO AMARILLO
COLINESTERASA	TUBO AMARILLO	PROTEINA DE BENCE JONES	ORINA PARCIAL
CORTISOL	TUBO AMARILLO	RUBEOLA IG G	TUBO AMARILLO
DEHSO4	TUBO AMARILLO	RUBEOLA IG M	TUBO AMARILLO
DENGUE IG G	TUBO AMARILLO	TESTOSTERONA LIBRE	TUBO AMARILLO
DENGUE IG M	TUBO AMARILLO	TESTOSTERONA TOTAL	TUBO AMARILLO
ELECTROF. DE PROTEINAS	TUBO AMARILLO	TIROGLOBULINA	TUBO AMARILLO
EPSTEIN BARR IG G	TUBO AMARILLO	VARICELA G Y M	TUBO AMARILLO
EPSTEIN BARR IG M	TUBO AMARILLO	**PANEL DE VIRUS**	**SECRECION NASOFARINGEA**
ESTRADIOL	TUBO AMARILLO	SUDAN	ORINA O MATERIA FECAL
FOSFOLIPIDOS IG G	TUBO AMARILLO	CALCULO URINARIO	CALCULO
COPROLOGICO	MATERIA FECAL	DEPURACION CREATININA	ORINA DE 24 HORAS
COPROCULTIVO	MATERIA FECAL	CREATINURIA	ORINA DE 24 HORAS
COPROSCOPICO	MATERIA FECAL	URICOSURIA	ORINA DE 24 HORAS
HAMBURGER RECUENTO	ORINA 3 HORAS	PROTEINURIA	ORINA DE 24 HORAS
PARCIAL DE ORINA	ORINA PARCIAL	CALCIURIA	ORINA DE 24 HORAS
PRUEBA DE EMBARAZO	TUBO AMARILLO	FOSFATURIA	ORINA DE 24 HORAS
SANGRE OCULTA	MATERIA FECAL	UROCULTIVO	ORINA PARCIAL

SARS-CO-2 ANTICUERPOS	TUBO AMARILLO	SARS-COV-2 RT-PCR	HISOPADO NASOFARINGEO
SARR-CO-2 FILM ARRAY	HIPOPADO NASOFARINGEO		

16.7 MANEJO DE REACCIONES ADVERSAS EN LA TOMA DE MUESTRAS EN EL LABORATORIO CLÍNICO

Durante la extracción de los volúmenes de sangre correspondientes a las muestras de laboratorio para la realización de los exámenes paraclínicos, se pueden presentar reacciones adversas que deben ser identificadas y manejadas con prontitud y eficacia por el personal del laboratorio clínico. En caso de ser necesario, el personal médico del servicio de urgencias está en capacidad de colaborar con el manejo del paciente.

Las reacciones adversas pueden ser de dos categorías: reacción vasovagal o reacción por venopunción. Ambas entidades no son mutuamente excluyentes.

16.7.1 VASOVAGALES

Son aquellas desencadenadas por un estimulo de índole nervioso o las asociadas a hipoperfusión cerebral, generalmente secundarias a una disminución del gasto cardiaco. Pueden ser causadas por dolor, visión de la sangre en el momento de la donación, por ver a otros donar sangre, por excitación individual o grupal, o aparecer inexplicablemente. De acuerdo con su gravedad (leve, moderada o severa) los hallazgos más comunes son náuseas, palidez y sudoración. Además debilidad, vómito, somnolencia, vértigo, visión borrosa, parestesias, cefalea, cianosis, escalofrío, hipotermia, taquipnea, descenso de la tensión arterial media, rigidez, temblor, disnea, pérdida de conciencia, taquicardia, convulsiones, relajación de esfínteres, dolor precordial y traumatismos por caída secundaria a pérdida de la conciencia.

Manejo general:

En todas las reacciones vasovagales se deben realizar las siguientes acciones:

- Tranquilizar al paciente, evitar la ansiedad y llevarlo, si es posible, a un sitio apto para manejar su caso en forma aislada.
- Si la reacción se presenta en el transcurso de la extracción, retirar el torniquete y la aguja del brazo del paciente.
- Aflojar las prendas de vestir y colocar al paciente en una posición cómoda (con el nivel de la cabeza inferior al resto del cuerpo para facilitar la perfusión cerebral: posición de Trendelemburg).
- Controlar presión arterial, pulso radial, frecuencia cardiaca y respiratoria, además de la actitud del paciente.

Manejo específico:

Las siguientes acciones específicas para cada reacción se aplican luego de haber aplicado las medidas anteriormente descritas.

a) **Síncope:** El síncope es una pérdida transitoria de la conciencia que no deja secuelas. Se manifiesta por pesadez, sudoración, vértigo, palidez, depresión del estado de conciencia, frialdad de la piel, hipotensión y bradicardia. Se presenta con rareza (0.08% a 0.34% de las donaciones) y en estos casos se debe:

- Verificar que la vía aérea sea permeable.
- Colocar compresas frías en la frente o nuca del paciente.
- Hacer inhalar suavemente alcohol o amoníaco.
- Ante la presencia de hipotensión, hacer ingerir al paciente una porción pequeña de sal para estimular el aumento de la presión arterial, si no ceden estas medidas remitir al servicio de urgencias.
- Si no hay mejoría remitir al servicio de urgencias más cercano para tratamiento respectivo.

b) **Hiperventilación**: Es una frecuencia de ventilación pulmonar mayor a la metabólicamente necesaria para el intercambio adecuado de los gases respiratorios. Es el resultado de una frecuencia respiratoria aumentada, de un aumento del volumen corriente en reposo o de una combinación de ambos factores y produce una entrada excesiva de oxígeno con espiración de dióxido de carbono. Aparecen hipocapnia y alcalosis respiratoria, se produce dolor torácico, vértigo, desfallecimiento, entumecimiento de los dedos de las manos y los pies y alteración psicomotora.

- Se debe distraer la atención del paciente conversando con él, para evitar la hiperventilación.

- Pedir al donante que respire profundo por la nariz y con la boca cerrada, que mantenga el aire en el tórax y después de unos segundos lo expulse lentamente por la boca, está forma de respiración la debe realizar por lo menos durante cinco minutos.

- Sí la sintomatología persiste, se debe hacer respirar al donante dentro de una bolsa de papel o plástico y se le solicita que tome el aire por la nariz y lo expulse con la boca abierta (no administrar oxígeno).

c) **Convulsiones:** Serie brusca, violenta e involuntaria de contracciones de un grupo de músculos, que puede ser paroxística y episódica como en las enfermedades convulsivantes, o transitoria y aguda, como en el caso de las que se presentan secundarias a una donación de sangre. Una convulsión puede ser clónica o tónica, focal o generalizada (unilateral o bilateral). En las convulsiones tónicas los músculos rígidos producen inmovilidad del segmento correspondiente, en el cual se produce ligero estremecimiento. En las convulsiones clónicas los músculos experimentan una serie de contracciones y relajaciones alternantes que ocasionan bruscas sacudidas del segmento.

- Evitar que al paciente se lesione o lesione a otra persona.
- Recostar al paciente en la camilla o en el suelo.
- Evitar que el paciente se muerda la lengua.
- Lateralizar la cabeza hacia la derecha o izquierda, para evitar broncoaspiración (posición de seguridad).
- Evitar la aglomeración de personal ya que se disminuye la oxigenación y se obstruye el paso del personal que debe tomar medidas inmediatas.
- Trasladar al paciente rápidamente al servicio de urgencias.

d) **Nauseas o vómito:** La emesis puede ir acompañada de desequilibrio hidro-electrolítico por pérdida de sodio, cloro e hidrogeniones, con la consecuente alcalosis metabólica.

- Indicar al donante que respire con lentitud y en forma profunda.
- Debido a que esta reacción hace parte del reflejo vasovagal y va acompañada de hipotensión, se debe colocar al paciente en posición de Trendelemburg.
- Colocar la cabeza del donante en posición de decúbito lateral derecho o izquierdo para evitar la broncoaspiración en caso de vómito.
- Suministrar una bolsa de aluminio plastificado para el vómito y proveer toallas de papel para que el donante se seque la boca.
- Ofrecer agua al donante para que se enjuague la boca.
- Si los síntomas persisten y según criterio médico, administrar 10 mg. de metoclopramida por vía endovenosa.

14.7.2. REACCIONES ADVERSAS POR VENOPUNCIÓN

Son causadas por lesión de los vasos ubicados a nivel antecubital, ya sea vena o arteria, y en algunos casos por lesión nerviosa de la región. En ciertos casos se debe a técnica inadecuada de venopunción y contaminación del área en el área en cuestión.

Manejo general:

- Retire el torniquete
- Detenga la sangría
- Retire la aguja
- Indique al paciente que realice presión digital por diez minutos
- Eleve el miembro afectado
- Aplique hielo en intervalos de cinco minutos
- Recomendar al paciente el uso de antiinflamatorios y paños de agua tibia
- Explicar al paciente que por lo general las lesiones causadas por la venopunción son de recuperación muy lenta.

Manejo específico:

a) **Hematoma:**

- Retirar el torniquete y la aguja.
- Colocar una gasa o apósito estéril sobre sitio de venopunción y aplicar presión digital durante 7 a 10 minutos con el brazo afectado elevado. El apósito debe permitir inspección frecuente del sitio de punción.
- Aplicar hielo en el área durante cinco minutos para favorecer la vasoconstricción local (tener la precaución de tener cubierto el hielo en una gasa o compresa para evitar quemaduras).

b) **Punción arterial**

- Si se sospecha una punción arterial retirar la aguja de inmediato y aplicar presión firme durante 10 minutos.
- Aplicar un vendaje compresivo enseguida.
- Controlar pulso radial. Si el pulso no es palpable o es débil, informar al médico o coordinador de la donación.
- A partir de la punción arterial pueden surgir complicaciones de tipo tardío:
    - Pseudoaneurisma

- Fístula arteriovenosa
- Síndrome compartimental

Estas complicaciones no tienen un manejo específico por parte del personal del banco de sangre donde se realizó la atención del donante. En estos casos el tratamiento para estas lesiones debe hacerse en un centro hospitalario donde se debe considerar un tratamiento quirúrgico definitivo.

c) **Dermatitis**

- Se debe sugerir al donante que utilice cremas hidratantes con vaselina.
- En caso de no mejorar con lo anterior se recomendará al donante que consulte con su médico para iniciar manejo de la lesión con corticoides.

d) **Celulitis**

- Dado que el agente más frecuente es *Staphylococcus aureus,* el antibiótico de elección es un beta lactámico de tipo dicloxacilina.

e) **Dolor neuropático**

- Esta lesión se debe a una lesión de nervio y es de carácter crónico. Se recomienda que el manejo de esta lesión sea multidisciplinario; debido a las características del dolor se requieren medicamentos que provean una analgesia más efectiva que la dada por los analgésicos de uso común como acetaminofén.
- Por lo anterior, se recomienda manejo inicial por parte de neurología, fisiatría y fisioterapia.

f) **Causalgia**

- Debido a que el compromiso en este tipo de dolor neuropático es mucho más severo y llega a comprometer sistema osteomuscular, se requiere manejo especializado.
- Como primer acercamiento terapéutico se inician medicamentos orales como gabapentín, calcitonina, bifosfonatos, calcioantagonistas, agonistas GABA y corticoides.
- Se sugiere la aplicación de tratamientos parenterales como ciclos de anestésicos nerviosos locales, por vía intravenosa o realización de bloqueos anestésicos nerviosos locales como inyecciones en el ganglio estrellado o en cadena simpática paralumbar, estimulación medular e infiltraciones intratecales, entre otros.

g) Flebitis

Se puede presentar de 12 a 48 horas después de la flebotomía, se manifiesta con dolor, rubor y calor sobre el sitio de la punción o en el recorrido de la vena.

- Realizar asepsia del sitio de la punción, manteniendo las manos y los guantes limpios.
- Usar equipo estrictamente estéril que se debe destapar al momento de la flebotomía.
- No tocar el sitio de punción después de realizada la asepsia y cubriendo el sitio de punción con una curita estéril.

- No realizar punciones cerca de una lesión en piel.
- Realizar valoración por parte del personal medico.
- De acuerdo a la escala de flebitis, referenciada en el documento OD-4.3.2-01 hacer revisar por parte del personal medico.

16.8 PREPARACIÓN PREVIA PARA EL PACIENTE

Examen	Preparación
Antitrombina III<br>Electroforesis de hemoglobina<br>Electroforesis de proteínas<br>Fibrinogeno<br>Glicemia<br>Glucosa-6-Fosfato<br>Globulinas<br>Insulina<br>Proteínas totales<br>PT INR<br>PTT<br>Serologia (RPR)<br>Tiroglobulina	**AYUNO MÁXIMO 12 HORAS**
Perfil lipídico (colesterol total, HDL, LDL, triglicéridos)	Ayuno previo de 14 horas, no consumir bebidas alcohólicas 72 horas antes, no consumir grasas animales por 48 horas y en 5 días anteriores a la toma debe seguir la alimentación diaria habitual.
Curva de glicemia y/o insulina	Ayuno de 8 a 12 horas. Disponer de 3 ½ horas para la realización del examen.<br>No fumar cigarrillo durante la prueba, no exponerse a estrés o realizar algún tipo de actividad física y suspender 3 días antes de la prueba.
Glicemia y/o insulina pre y post	Ayuno de 8 – 12 horas. Disponer de mínimo 2 ½ horas para la realización del examen.
Perfil tiroideo	No tome su medicamento para la tiroides antes de la toma de muestra, indique que droga toma y en qué dosis.
Ácido fólico (Folato)	Ayuno de 12 horas, evitar bebidas alcoholicas, ácido aminosalicílico, ampicilina, antipaludicos, cloramfenicol, ampicilina, estrogenos, metotrexano, penicilina, una semana antes de la toma.
ACTH (Hormona	Evitar estar en situaciones de estrés previo a la toma

Adenocorticotropica)	de la muestra.
Aldosterona	Mantener una dieta normal en sodio, el paciente no debe estar expuesto a radiactividad 4 horas antes de la prueba.
Antígeno prostático (PSA libre / total)	No se puede realizar luego del examen físico de próstata (tacto rectal). Verifique que hayan pasado mínimo una semana. Abstinecia sexual de 3 días.
Alfa 1 Antitripsina	No requiere ayuno, evitar situaciones de estrés y ejercicio.
Acido úrico<br>Alfa Feto Proteína<br>Amilasa<br>Antígeno de Superficie de la hepatitis B (HbsAg)<br>Anticuerpo contra el antígeno de superficie de la hepatitis B (aHBs)<br>Anticuerpos Anti Chagas<br>Anticuerpos anti-core de la hepatitis B (aHBC)<br>Anticuerpos Anti Epstein Barr (igM-IgG)<br>Anticuerpos Anti Músculo Liso (ASMA)<br>Anticuerpos Antinucleares (ANAS)<br>Anticuerpos Anti citomegalovirus (IgM-IgG)<br>Anticuerpos Anti Hepatitis A (IgM-IgG)<br>Anticuerpos de Herpes Simple I-II (IgM-IgG)<br>Anticuerpos Anti DNA<br>Anticuerpos Microsomales<br>Anticuerpos Antimitocondriales (AMA)<br>Anticuerpos de Rubeola (IgM-IgG)<br>Anticuerpos Anti-HIV<br>Anticuerpos Totales Anti Hepatitis	**NO REQUIERE AYUNO**

C<br>Anticuerpos Tiroglobulinicos<br>Anticuerpos Tiroideos<br>Anticuerpos Anti Toxoplasma (IgM-IgG)<br>Antiestreptolisina O (ASTOS)<br>Antígenos Bacterianos<br>Antígenos Nucleares Extractables (ENAS)<br>Antígeno Carcinoembrionario (ACE)<br>Antígeno CA 15-3<br>Anticuerpos C-ANCA, P-ANCA<br>Bilirrubinas (Totales, directa, indirecta)<br>Calcio Sérico<br>Calcio Iónico<br>Cloro sanguineo<br>Creatinina<br>Creatinkinasa (CK)<br>Creatinkinasa (CKMB)<br>Colinesterasa<br>Cuadro Hematico<br>C3 y C4<br>Dengue IgG – IgM – Antigeno NS1<br>Deshidrogenasa Lactica (LDH)<br>Gamma Glutamil Transferasa (GGT)<br>Hierro (Fe)<br>Hormona del Crecimiento (GH)<br>Hemoclasificación<br>Hemoglobina glicosilada<br>Inmunoglobulinas (IgA, IgD, IgE, IgG, IgM)<br>Magnesio<br>Nitrógeno Ureico (BUN)<br>Plomo<br>Potasio<br>Proteína C Reactiva<br>Recuento de plaquetas<br>Sodio<br>Somatomedina C	

Transaminasas (TGO - TGP)<br>Test de RA<br>Testosterona libre y total<br>Transferrina<br>Troponina I	
Amonio	No requiere ayuno, no fumar 12 horas previo a la toma de la muestra.
Alergenos (Anticuerpos IgE)	Suprimir por 24 horas la administración de isotopos. Especificar tipo de alérgeno a solicitar, ayuno previo.
Androsterona	Suspender la toma de ACTH, gonadotropina o medicamentos para tiroides 48 horas antes de la toma.
Antígeno CA 125	No requiere ayuno, no tomar durante el periodo menstrual.
Apoliproteínas A1y B	Requiere ayuno previo de 14 horas, con el régimen habitual de alimentación.
Citología Cervico Vaginal	- No tener relaciones 3 días previos a la realización.<br>- Realizarla 5 días antes o 5 días posteriores al periodo menstrual.<br>- No haber usado tratamiento con óvulos 3 días previos a la toma.
Coprocultivo, Clostridium difficile	Materia fecal diarreica.
Coprológico<br>Copróscopico	Materia fecal, dos horas màximas a su recolección
Cortisol en suero	De acuerdo a solicitud médica, se hacen una o dos tomas. Llegar 20 minutos antes para tramites administrativos.<br>Para única toma: se debe tomar a las 8 am.<br>Dos tomas: primera toma 8 am<br>segunda toma 4pm
Dehydroepiandrosterona Sulfato (DHEAS)	Evitar el ejercicio fuerte durante 48Horas
Contol de drogas terapéuticas (digoxina, carbamazepina, fenitoina, fenobarbital, ciclosporina, tracolimus)	Ayuno previo, informar fecha de la última toma, tomarse la muestra 12 horas después de la ultima toma.

Electroforesis en orina<br>Osmolaridad urinaria	Muestra de orina
Depuración de creatinina, proteinuria, nitrógeno ureico, calcio, sodio, potasio, cloro, cortisol en orina de 24 horas,	Recolectar una orina de 24 horas de la siguiente manera:<br>✓ Reclame en el laboratorio un frasco limpio y seco o adquiéralo<br>✓ Inicie la recolección de la orina con la segunda orina de la mañana, recoja toda la orina emitida durante el transcurso del día y la noche y la primera del día que termina.<br>✓ Una vez finalizada la recolección, llévela al laboratorio lo más pronto posible, **evite perder el volumen de la orina.**
Ácido homovanílico, ácido vanilmandélico, ácido 5 hidroxiindolacético, catecoláminas, metanefrinas, porfobilinógeno, oxalatos en orinas.	Recolectar una orina de 24 horas de las siguiente manera:<br>✓ Reclame en el laboratorio un frasco oscuro.<br>✓ Inicie la recolección de la orina con la segunda orina de la mañana, recoja toda la orina emitida durante el transcurso del día, la noche y la primera del día que termina.<br>✓ Una vez finalizada la recolección de la orina llévela al laboratorio lo más pronto posible, **evite perder el volumen de la orina.**<br>**Recuerde:** no consuma café, té, banano, vainilla y verifique con su médico que los medicamentos que toma no interfiera con el examen.
Hormona Foliculo Estimulante (FSH)<br>Hormona Luteinizante (LH)<br>Prueba de embarazo<br>Beta HCG cuantitativa<br>Estradiol<br>Progesterona	No requiere ayuno. Por favor indique la fecha de la última menstruación.
Linfocitos CD3 – CD4	Lunes a jueves de 6:30 a.m. a 8:30 a.m, con ayuno previo de 10 horas.
Litio	Indique la dosis y la última hora de la ultima dosis.
Microalbuminuria	Primera orina de la mañana.
Parcial de orina	Recoja la primera orina de la mañana inmediatamente después del baño, descartando el primer chorro. En caso de recoger la muestra en el laboratorio debe

	tener mínimo 3 horas de retención.
Recuento de hamburguer, pirilinks	Recoja una orina de 3 horas en un frasco limpio y seco. Preferiblemente que incluya la de las 9 a.m.
Cultivo de BK en orina	Recoja la primera orina de la mañana, completa, en un frasco limpio y seco.
Urocultivo	Recoja la primera orina de la mañana inmediatamente después del baño, descartando el primer chorro. En caso de recoger la muestra en el laboratorio debe tener mínimo 4 horas de retención y realizar aseo previo.
Sangre oculta	No debe haber evidencia de sangrados odontológicos, no recoja la muestra si está con el período.
Prolactina	Se debe tomar mínimo 2 horas después de haberse levantado.
Coloración y/o cultivo de BK	Reclame en el laboratorio el frasco indicado (tapa roja), recoja la muestra entréguela al laboratorio lo más pronto posible.
Agregación plaquetaria, HLA, B27. Toma de muestras con sonda.	Ayuno previo. Pedir cita previa de lunes a jueves al teléfono (1) 271 43 31.

14.0. BIBLIOGRAFÍA

- Budassi S. Laboratory Specimens. In: Emergency Nursing. Principles and Practice. Mosby Yearbook. St. Louis, 1992.
- Campuzano-Maya G. El hemograma. Medicina & Laboratorio. 1998; 8:19-32. Cuervo P, Rico C. Guía para la toma de hemocultivos. Actual Enferm 2001; 4:33-36.
- De Pedro J, Llobera J, Bennassar M, et al. Eficacia de dos métodos de compresión en la aparición de hematomas postextracciones sanguíneas. Enfermería Clínica 2002; 12:1-5. De Merino N. Manual de Procedimientos. Departamento de Patología y Laboratorio Clínico. Tercera edición. Fundación Santa Fe de Bogotá. Bogotá, 2001.
- Tucker S, Canobbio M. Diagnósticos básicos de enfermería, necesidades especiales y equipamiento. En: Normas de Cuidados del Paciente. Harcourt Oceano. Barcelona, 2002.
- Wilson S, Thompson J. Trastornos respiratorios. Ediciones Doyma y Times Mirror.

Barcelona, 1993.

- Castro Rivera Sandra, Enf. Clinica Fundación Valle del Lili.
- Jacques Wallach. Interpretación clínica de pruebas diagnosticas.8° edición.Lippincott Williams & Wilkins. España 2008.
- Manual para toma de muestras de análisis microbiologico, secretaría distrital de salud-fundación cic salud. Bogota 2015.
- Diccionario de Laboratorio Aplicado a la Clínica, 3ra edición, Angel Mejia Gilberto, 2004.
- Interpretación Clínica del Laboratorio, 8a edición, Gómez Gutierrez – Casas Gomez, 2014.
- Norma ISO 15189:2012 "Laboratorios Clínicos – Requisitos de la calidad y competencia"
- ISO/TS 20658:2017 "Laboratorios médicos-Requisitos para la recolección, transporte, recepción y manejo de muestras"
- Simundic et al.: Joint EFLM-COLABIOCLI Recommendation for venous blood sampling – v1.1, junio 2018.
