# Supplementary material for: Molecular characterization and descriptive analysis of carbapenemase-producing Gram-negative rod infections in Bogota, Colombia
Source: Microbiol Spectr. 2024 Apr 17;12(6):e01714-23. doi: 10.1128/spectrum.01714-23 (PMC11237484; doi:10.1128/spectrum.01714-23)
Supplement: Sample collection protocol — Sample collection manual for Clinica Universidad de La Sabana. [file spectrum.01714-23-s0002.pdf]

ENGLISH VERSION
(TRANSLATED)

Machine Translated by Google

1. INTRODUCTION

This manual describes the standardized procedures for taking Clinical Laboratory samples for hospitalized outpatients, surgery patients and emergency patients with the aim of guaranteeing optimal quality in the pre-analytical phase of the Process, which is of vital importance. to generate a safe and reliable result in the analytical and post-analytical phases.

Compliance with the procedures included in this Manual are the responsibility of the Laboratory Nursing Auxiliary collaborators in charge of the Sample Collection area for Outpatient Patients, of the care staff: Nursing Auxiliaries or Heads of Nursing of the University Clinic of La Sabana in the different services and of Professionals such as Doctors and Respiratory Therapists of the Clínica Universidad de La Sabana.

The Laboratory Bacteriologists will provide support to the Assistant in taking external consultation samples and will supervise the process with a report to the Administrator in charge of the headquarters.

This Manual will be updated every year by the collaborators assigned at headquarters and reviewed by the Administrator.

The main reasons for using Laboratory services are: collecting biological samples for therapeutic, diagnostic or prophylactic purposes, discovering diseases in sub-clinical stages, ratifying a questionable diagnosis, specifying risk reasons, which is why efficient collection is of utmost importance. of the samples so that in their subsequent analysis a result is obtained that contributes to competent health personnel and offers a diagnostic support solution for patients.

2. GENERAL OBJECTIVE

Provide a guide and consultation tool unifying the concepts and procedures for the collection of different clinical specimens, both to Laboratory collaborators and to the Clinic's healthcare personnel responsible for taking samples in the different services and who wish to consult.

3. SPECIFIC OBJECTIVES

1. Define this Manual as the learning and training base for the Laboratory Assistant collaborators of the Universidad de La Sabana Clinic headquarters regarding sample collection.

2. Standardize the sampling procedure in the different hospital services of the Clínica Universidad de La Sabana, consolidating teamwork.

3. Describe the preparation conditions that patients must meet to attend the Outpatient Sample Collection service.

4. Establish the criteria to act with the greatest skill, ensuring that the patient does not feel uncomfortable, transmitting confidence, security and professionalism during the procedure, demonstrating good Biosafety practices.

Machine Translated by Google

5. Guarantee optimal sample collection that minimizes risk to the patient.

6. Consider the patient as a human being who, in most cases, presents some illness and goes to the laboratory asking for help.

7. Describe the extramural sampling process, contemplating sampling in companies or institutions and taking samples at home.

4. SCOPE

This Manual applies to the Sampling procedures of the Compensar Clínica Universidad de La Sabana Clinical Laboratory Headquarters.

5. DEFINITIONS

ÿ **SAMPLING SERVICE:**

It is the one that has the appropriate technical and human resources destined exclusively for taking samples and/or biological products that will be processed in the clinical laboratory of the Clínica Universidad de la Sabana headquarters with their different degrees of complexity, on which they depend legally. , technically, scientifically and administratively in order to increase the accessibility and opportunity to the service by users who require clinical examinations, complying in a professional and ethical manner with the established standards and procedures.

ÿ **Biological Samples:** Any human or animal material including excreta, blood or its components, tissues and tissue fluids, collected for the purpose of making a diagnosis.

ÿ **Biosafety:** Standards of behavior and preventive management of health personnel against potentially pathogenic microorganisms.

ÿ **Primary Container:** Spill-proof container, made with materials that withstand changes in temperature, pressure or humidity to which it may be exposed during transport, they do not break easily or let their contents escape. If the contents of these containers are liquid, they must be wrapped in sufficient absorbent material to contain the liquid. The sample is contained in this container. (tube, urine bottles, blood culture bottles, etc.)

ÿ **Secondary Container:** Second, leak-proof container that encloses and protects the primary container(s, secondary bottle). It must have absorbent material to protect the primary containers and prevent collisions between them.

ÿ **Tertiary container:** Made of rigid material, it withstands changes in temperature, humidity and pressure to which it may be exposed during transport. It must be spill proof. (Transport refrigerator)

ÿ **Pathogen:** microorganism (bacteria, virus, fungus or parasite) or recombinant microorganism (hybrid or mutant), which is known or believed to cause an infectious disease in animals or humans.

ÿ **Sample stability:** capacity of a sample, when maintained under specified conditions, to maintain the values of its biological properties within pre-established limits.

ÿ **Infectious substances:** For the purposes of transport, infectious substances are understood to be substances for which it is known or reasonably believed that

Machine Translated by Google

They contain pathogenic agents. Pathogens are microorganisms (such as bacteria, viruses, rickettsiae, parasites and fungi) and other agents such as prions, which can cause diseases in animals or humans. The definition applies to all samples except those explicitly excluded (see below). Infectious substances are divided into two categories.

ÿ **Information Sheet:** The sheet contains and describes the conditions and requirements necessary for tests carried out in the Compensar Clinical Laboratory or support network. The healthcare staff of the Clínica Universidad de La Sabana can consult the Compensar Services Information Sheet through the folder shared for this purpose from the Clinic's Nursing Headquarters.

ÿ **Anticoagulants:** These are substances that prevent the formation of clots. There are different types of them in powder or liquid. The appropriate anticoagulant should always be selected according to the study that needs to be performed. The most commonly used anticoagulants are: EDTA, Sodium Citrate, Heparin, Oxalates.

ÿ **Sodium Citrate:** Anticoagulant that is generally used in concentrations of 3.8% in coagulation studies. It works by chelating calcium.

ÿ **EDTA: (ETHYLENE-DIAMINO-TETRA-ACETATE)** This type of anticoagulant is mainly used when studies are carried out where cells are counted. It works by chelating calcium.

ÿ **Heparin:** It is used both in some routine and specialized studies. Its presentation may include heparin with concentrations of sodium or lithium. In general, lithium heparin is used for chemistry studies and sodium heparin is used for lymphocyte studies. It acts by accelerating the inhibition of factor Xa by antithrombin.

ÿ **Exogenous:** Any factor or mechanism added to the sample in vivo (i.e., a drug) or in vitro (i.e., a contaminant).

ÿ **Influence Factor:** Biological influence (in vivo and in vitro) on the value of a biological quantity in a system (e.g. venous blood)

ÿ **Interference Factor:** Component of the matrix of a sample that differs from the analyte and interferes with the analytical procedure to give a false measurement signal.

ÿ **COVID-19:** Infectious disease caused by Coronavirus discovered in 2019. Coronavirus disease 2019 is the name assigned to the pathology caused by infection with the Acute, Severe or Severe Acute Respiratory Syndrome 2 coronavirus.

6. CHAPTERS

1. GENERALITIES

1. SAMPLE TAKING TIMES:

There are two types of times in exams. The first type (simple time), when it is an exam that only requires one puncture. The second type (multiple time), when the examination requires and includes different samples at different times. In any case, it must be taken into account:

to. The patient's preparation conditions must be correctly adopted.
b. Some patients take medications during the day, which can interfere with the results; This must be taken into account when analyzing the exams.
c. If the patient is ordered to return to the laboratory to take second samples, they must

Machine Translated by Google

The recommendations that the patient must follow will be provided, while the time comes for subsequent studies.

2. BIOSAFETY PRECAUTIONS IN SAMPLING

Improper handling can become a source of biological risk for people who are in contact with the samples or for the environment. Use the necessary personal protection elements to avoid exposure with biological risk, according to the source of the sample.

In the event of an accident with biological risk, notify immediately according to the recommendations of the institutional biological risk work accident protocol. See Biosafety Manual.
Auxiliary personnel and/or bacteriologists must follow all biosafety standards for patient care, See Biosafety Manual.

BIOSECURITY CONTAINERS

- Leak-proof, easy-to-seal specimen containers.
- Comply with the recommendations for handling sharps:
- Do not resheath needles.
- Properly arrange and use the sharps container.

PERSONAL PROTECTION ITEMS

- Eye protection: glasses or mask with visor.
- Mask and/or face covering.
- Disposable gloves.
- Anti-fluid or disposable gown.
- Cap.

3. COLLECTION OF BLOOD SAMPLES.

Venipuncture is one of the most common procedures that patients undergo in the Clinical Laboratory, carries a risk for the patient, since it is opening a dangerous Door to a totally aseptic area such as blood, that is why every venipuncture implies the risk of infection if all aseptic techniques are not taken into account. The infection can be caused by: contaminated equipment, contamination with microorganisms by air, due to poor aseptic technique during venipuncture. These risks can be minimized by performing the procedure with strict aseptic technique and maintaining care at the venipuncture site.

GENERALITIES ON VENIPUNCTION:

ÿ The staff in charge of taking and collecting Laboratory samples is made up of Nursing and/or Laboratory Assistants certified and trained in the area who assume responsibility for the correct collection of samples. For the sample taking process, there is advice and support from the Laboratory coordinator and the group of Bacteriology professionals. To take samples from hospital patients, the nursing staff is in charge of taking the samples in the services and their

Machine Translated by Google

delivery to the laboratory.

ÿ To take blood samples, special cubicles are assigned for this purpose in the clinical laboratory. The process is carried out by an exclusive assistant for this activity, guaranteeing confidentiality in care for all patients, including minors.

ÿ The assistant responsible for taking the sample will verify the risk of falls according to the use of the red button for adults, the blue handle for minors in outpatient areas, and the yellow button for the companion. In accordance with patient safety goals.

ÿ The exams are taken according to medical requirements from 6:00 am to 10:00 am by outpatient consultation and throughout the day they are received from the different services of the clinic. (Sample collection in the emergency department, ICU patients who do not have a central catheter and the morning hospitalization round is carried out by the laboratory staff).

ÿ It must be verified that patients meet the conditions required for sample collection before venipuncture.

ÿ It should be considered that all samples of biological specimens must be treated as potentially infectious.

ÿ Sufficient and adequate quantity and quality of blood must be obtained according to the type exam.

ÿ Ensure that the work elements are in perfect physical condition, dates of expiration, cleaning, disinfection and/or sterilization.

ÿ Keep the Laboratory clean and tidy, avoiding the presence of materials and equipment that are not related to work.

ELEMENTS FOR TAKING SAMPLES.

TUBES FOR BLOOD SAMPLES

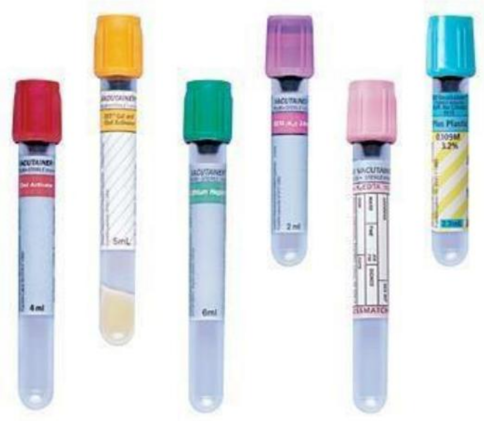

LILAC CAP: Contains EDTA as an anticoagulant, which is filled under vacuum according to the volume specified by the tube, taking into account that there are pediatric and adult tubes that require different volumes of blood. It is used for the determination of hematology tests and transfusion service.

When taking samples with this type of tube, it is vitally important to ensure the proper ratio between blood and anticoagulant. For this, the blood volume must always be checked in the tube.

Machine Translated by Google

which requires. If the sample is taken with a vacuum system, this helps maintain the relationship; if it is with a syringe, it must be filled up to the mark indicated by the manufacturer.

YELLOW/RED LID: It is a tube that contains a gel that allows the separation of the sample (serum – globular package), it must be filled according to the volume specified by the tube, taking into account that there are pediatric and adult tubes that require volumes. of different blood. This tube allows, after the centrifugation process, the globular package to be separated by the gel from the serum in which the determinations are made. It is used for blood chemistry and serological measurements, among others. Tube contains a procoagulant substance that accelerates the blood clotting process.

RED CAP TUBE WITHOUT GEL: Tube that does not contain any procoagulant substance or gel. This tube is used to perform tests that generate interference with procoagulants or the gel, such as methyl alcohol.

GRAY CAP: This tube has sodium fluoride as an anticoagulant. It is used for semi-automated measurement of ethyl alcohol and lactate by some techniques. In the laboratory offset is not routinely used.

BLUE CAP: This tube contains sodium citrate as an anticoagulant, which is used for coagulation tests. For coagulation tests, the preservation of the blood-anticoagulant ratio is of vital importance. It must be filled according to the volume specified by the tube, taking into account that there are pediatric and adult tubes that require different volumes of blood. It should be centrifuged as soon as possible to avoid the consumption of coagulation factors. It can be stored at room temperature or refrigerated until it is transported, preferably no more than 2 hours after taking.

GREEN LID: Contains sodium heparin as an anticoagulant. Used for determination of venous gases and cardiac markers.

ORDER IN FILLING THE TUBES:

In accordance with the manufacturer's recommendations, the order of filling tubes is determined:

Machine Translated by Google

Orden de Toma

Tapón	Contenido de tubo	Área de uso	Inversiones
	Hemocultivo	Microbiología	5 veces
	Citrato de sodio	Coagulación (Tiempos de coagulación fibrinógeno, agregación plaquetaria)	3 a 4 veces
	Gel separador	Química clínica	5 veces
	Sin anticoagulante, con activador de coagulación, con silicón	Química clínica, banco de sangre serología	8 a 10 veces
	Gel separador y trombina	Obtención de suero rápido	5 a 6 veces
	Gel separador y heparina de litio	Química clínica en plasma	5 veces
	Heparina de sodio/litio	Química clínica (urgencias) hematología (fragalidad osmótica)	8 a 10 veces
	EDTA K_2	Hematología, banco de sangre	8 a 10 veces
	Gel separador y EDTA K_2	Determinaciones de carga viral	8 a 10 veces
	Oxalato de Potasio/NaF	Química clínica, pruebas de lactato y glucosa	8 veces

1. Taking Order Table. BD Vacutainer Venous Blood Collection System TUBING MIXING: The adequate homogenization of the blood samples guarantees the optimal performance of the medical devices, contributing to obtaining reliable results during the analytical and post-analytical phase. Remember to homogenize these devices as illustrated below:

INSTRUCTIVO PARA MEZCLA DE TUBOS LABORATORIO CLINICO COMPENSAR compensar

DISPOSITIVO MEDICO	MEZCLA POR INVERSION (De techo a piso)		IDENTIFICACION TAPA DE TUBO POR COLOR
TUBO TAPA AMARILLA	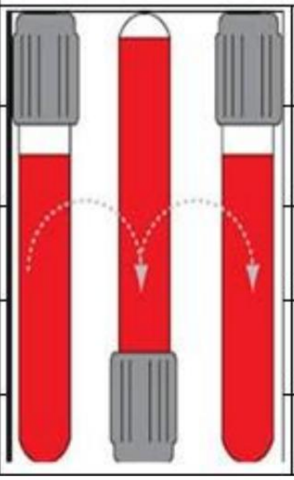	5 - 8 Veces	
TUBO TAPA AZUL		3-4 Veces	
TUBO TAPA VERDE (HEPARINA DE LITIO), LILA Y GRIS		8-10 Veces	
TUBO TAPA VERDE, LILA Y GRIS, PERLA		8-10 Veces	
TUBO TAPA VERDE (HEPARINA DE LITIO Y GEL SEPARADOR)		5 Veces	

Machine Translated by Google

- After sample extraction, the tubes should always be kept vertically in a rack, promoting the due process of retraction of the sample clot, which generally occurs 30 minutes after taking the sample.

- Storage and transportation: The samples must be centrifuged, preserved, stored and transported according to the requirements set forth in the information sheet which is available on the Compensar intranet.

VACUTAINER SYSTEM

- HOLDER OR SHIRT: Yellow or transparent plastic container where the needle is assembled and which in its lower part has a hood where the tubes are inserted for sample collection.

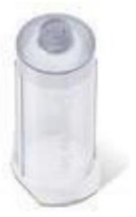

- NEEDLES: They are inside green capsules and have 2 parts, a rubber cover where the tubes are inserted, and the needle, which is used to puncture the patient.

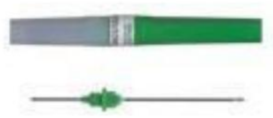

- BUTTERFLY ADAPTER: This allows the Vacutainer system to be adapted for use by butterflies, in patients with difficult venous access or in infants and children.

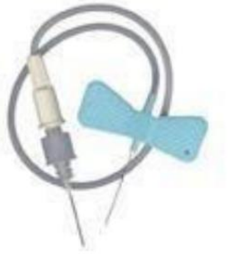

- **MICROTAINER SYSTEM:** Tubes used for pediatric sampling or for patients with difficult venous access. In the clinic, a lilac-cap microtainer tube is available for hematology and a blue cap for coagulation and a yellow cap for serum tests.

SYRINGES WITHOUT ADDITIVES

They should be used minimally and only in cases of very difficult venous access. According to the required volume of samples, there are 3ml, 5ml and 10ml syringes.

Machine Translated by Google

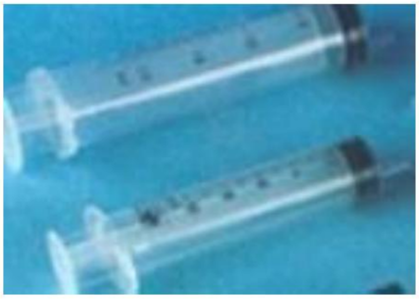

2. CARE PROTOCOL IN SAMPLE TAKING

To complete all informed consents, apply the 5 correct defined ones:

CORRECTOS PARA EL DILIGENCIAMIENTO DEL CONSENTIMIENTO INFORMADO

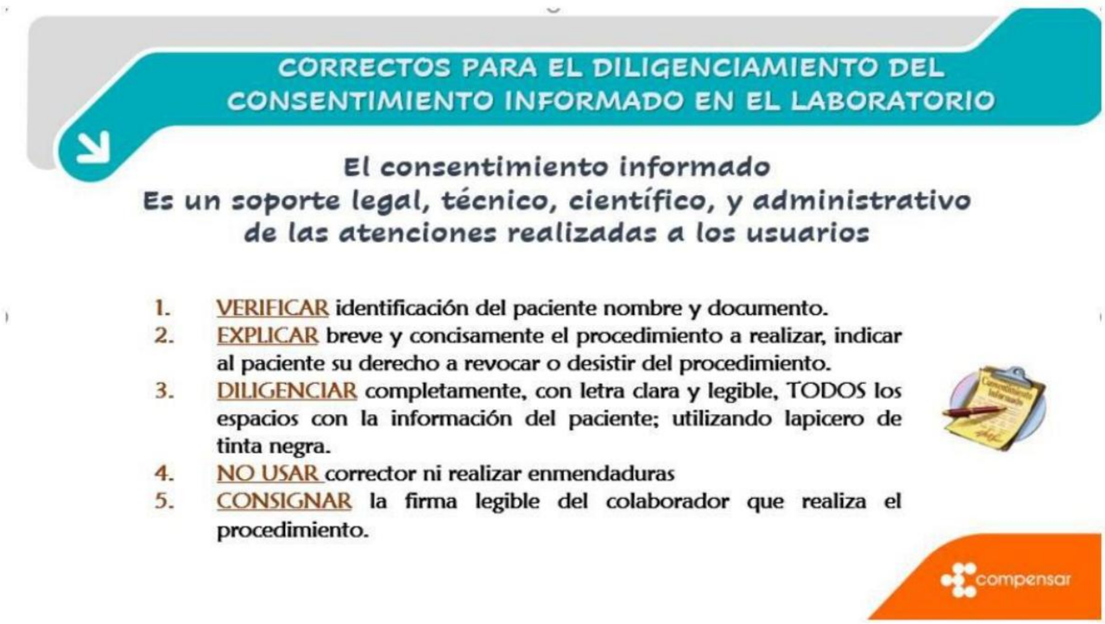

INFORMED CONSENT FOR TAKING CLINICAL LABORATORY SAMPLES

This procedure is defined for the care of outpatient patients and outpatient emergency patients. Observation and hospitalization patients, consent is immersed in the service. The format is found in the clinical portal with the code LC.01.MA.02.FT.01. The signing of this consent is given after the risks associated with sample collection, complications and events that may occur as a result of the procedure are explained to the patient.
Refusal is also included so that the patient has the option to refuse the procedure.

Machine Translated by Google

INFORMED CONSENT FOR ADMINISTRATION OF GLUCOSE LOADS

All patients with a pre- and post-load glucose order must give their consent using the LC.01.MA.02.FT.02 form, which is available on the clinical portal. Prior to signing, the procedure to be performed and the risks associated with ingesting glucose loads must be explained to the patient.

INFORMED CONSENT FOR HIV TESTING

Every patient with an HIV testing order must give consent. The Pre-test and post-test counseling process is in charge of the treating doctor who, after the counseling, will complete the DM.01.PR.01.FT.34 form. Before an HIV test is ordered, the laboratory assistant must verify the existence of this consent by verifying the signature of the patient or guardian who is aware of the test being performed. This must be done with orders for both external consultation and hospitalization and emergencies. For external consultation, only consents made at the institution will be received, which will guarantee subsequent attention for post-test counseling.

INFORMED CONSENT FOR RAPID TEST PROCESSING SARS CoV 2 (COVID-19) ANTIGEN DETECTION

Rapid test for diagnosis of COVID 19.

This test applies to:

* Person with symptoms for less than 11 days, treated in an emergency or hospital setting, where, due to territorial conditions, there is no capacity to carry out RT-PCR molecular tests.

*In outpatient or home services for symptomatic people and prioritized risk groups. To asymptomatic non-cohabiting contact with the confirmed case, within an epidemiological fence study.

*People who live in dispersed rural areas

APPLIES FOR OUTPATIENT OR HOME PATIENT:

*Person treated in the home or outpatient setting: In person with COVID-19 symptoms with less than 11 days of onset of symptoms. A person with one or more of the following symptoms is considered symptomatic of COVID-19: fever, cough, respiratory distress, odynophagia and/or fatigue/asthenia. These symptoms may or may not be accompanied by gastrointestinal symptoms such as diarrhea, vomiting, abdominal pain and others such as dysgeusia (loss of taste) or anosmia (loss of smell).

*Asymptomatic person who is a close contact of a confirmed case or deceased with COVID-19: RT-PCR test or antigen test will be taken on day 7 of exposure with the case.

Asymptomatic person with or without risk factors who is a close contact of a confirmed case or deceased with COVID-19:

* Take RT-PCR or antigen detection test on the seventh day of confirmed unprotected close contact or death.

Machine Translated by Google

This test does not apply to:

• Person whose onset of symptoms or exposure date is greater than 11 days.

It does not require strict fasting (unless the request is accompanied by exams that do require it).
Take a nasopharyngeal swab sample according to the protocol in Annex 1.

Apply *"SARS CoV 2 (COVID-19) RAPID TEST CLINICAL DATA SURVEY ANTIGEN DETECTION"* ANNEX 3

INFORMED CONSENT FOR THE PROCESSING OF SEROLOGICAL TESTS SARS Cov 2 (covid 19)

The qualitative detection test for IgG antibodies against SARS CoV-2 Virus applies to __________ people with probable mild symptoms of COVID-19, without risk factors, who are at least 11 days since the onset of symptoms. If the person has symptoms for less than 11 days, the RT-PCR molecular test should be applied.2.

This test does not apply to people treated in a home or outpatient setting who have the following risk factors:

1. Be a health worker who had contact with a probable or confirmed case of COVID-19.
2. Adult person over 70 years of age.
3. Person of any age who presents symptoms of COVID-19 with comorbidities (diabetes, cardiovascular diseases, high blood pressure, cerebrovascular disease, chronic respiratory disease, HIV or other immunodeficiency, cancer, autoimmune diseases, prolonged use of steroids, kidney failure, obesity , malnutrition) and smoking.
4. Asymptomatic person with or without risk factors, close contact of a confirmed case with COVID-19 or close contact of a person who died from confirmed COVID-19.
6. Active search in population groups: first evaluate the presence of symptoms suggestive of COVID-19. If the symptoms are of recent onset (equal to or less than 10 days).

It is defined as Close contact: less than 2 meters for 15 minutes with a confirmed COVID 19 case.

These tests should not be used for asymptomatic patients or for screening. It does not require strict fasting (unless the request is accompanied by tests that do require it). Take sample in dry tube.

Apply "Informed Consent for the processing of SARS CoV 2 (COVID 19) serological tests". ANNEX 4

CHAIN OF CUSTODY

The Chain of Custody is a system that guarantees the authenticity of physical elements and evidence to demonstrate and ensure that the conditions of identity, integrity, preservation, security, continuity and registration of the same are applied from the beginning until the ends the chain.

Machine Translated by Google

Chain of custody record: It is the exhaustive and documented history of each transfer and transfer of physical evidence material, during the development of the chain of custody process.
It allows you to verify the identity, state and original conditions of the physical evidence elements, as well as the modifications made to them, establishes the route followed by said elements, determines their place of permanence and the person responsible for their custody at each stage of the procedure. .

To determine the tests for drugs of abuse and alcohol, it is required to complete FOR PSS 474 Chain of Custody Clinical Laboratory for toxicological or microbiological samples.

In the case of urine toxicology, in the emergency and hospitalization services, the head of nursing must accompany the patient during the collection of the sample and deliver it to the laboratory staff with the chain of custody. For outpatient patients, the sample collection assistant will monitor and verify the sample collection and initial completion of the chain of custody.

EPIDEMIOLOGICAL SHEETS

For events requiring mandatory notification to SIVIGILA, the treating physician must complete the corresponding epidemiological notification form. These files are left in the epidemiology mailbox without requiring referral of samples to the Public Health Laboratory. If samples are sent to LSP, this form must be requested to be sent along with the samples.

Some notification events are:

- Acute respiratory infection ESI-SARI
- SARI
- Measles
- Rubella
- Whooping cough
- Bordetella
- Brucella

ADDITIONAL FORMATS

In some cases, according to the evidence requested, and according to the requirements mentioned in the compensation information sheet, additional formats must be requested. Some examples are genetic tests, metabolic errors, among others. It is essential to verify the conditions and requirements for processing special tests in the information sheet.

3.

For outpatient care, the schedule is defined from Monday to Friday from 6:00 am to 10:00 am and Saturdays from 6:30 to 10:00 am. There is an assistant for taking samples

Machine Translated by Google

and an assistant for the window service process prior to taking the sample. TO The activities to be carried out are described below:

ÿ The call is made digitally. Front line assistant performs the first service in which the data, invoice and order are verified and compliance with the conditions for taking the exams is verified. For this, it must be verified in the clinical laboratory information sheet according to the ordered tests, what conditions the patient requires. In case of non-compliance, the patient must be redirected to the access window where the patient return information is consolidated for indicator registration.

ÿ If the requirements are met, the entry is made through the information system and the patient is redirected to the sample collection module. The response time for the exams and the results delivery time are previously reported. It is important to inform the user of the possibility of sending results by email after completing authorization.

ÿ During the care process for sample collection, the assistant must comply with the correct sample collection procedures listed below and described in the clinical laboratory care model:

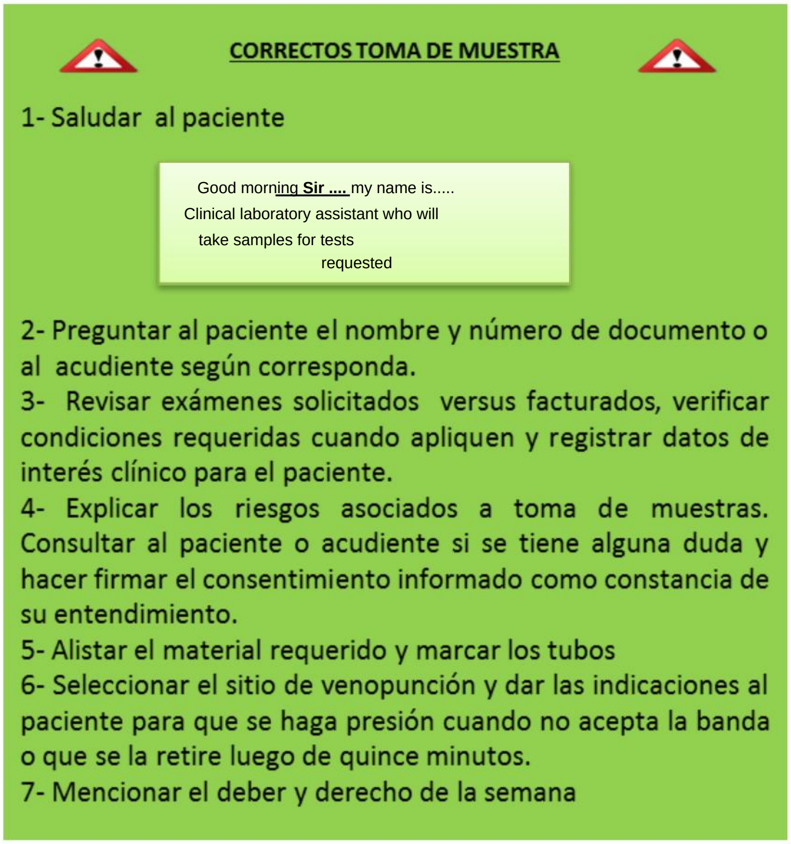

Outpatient Arterial Gas samples are taken by the Clinical Laboratory Assistant. If the puncture is difficult to access, the support of the Respiratory therapist is requested.

Machine Translated by Google

of availability.

Comply with hand washing as established:

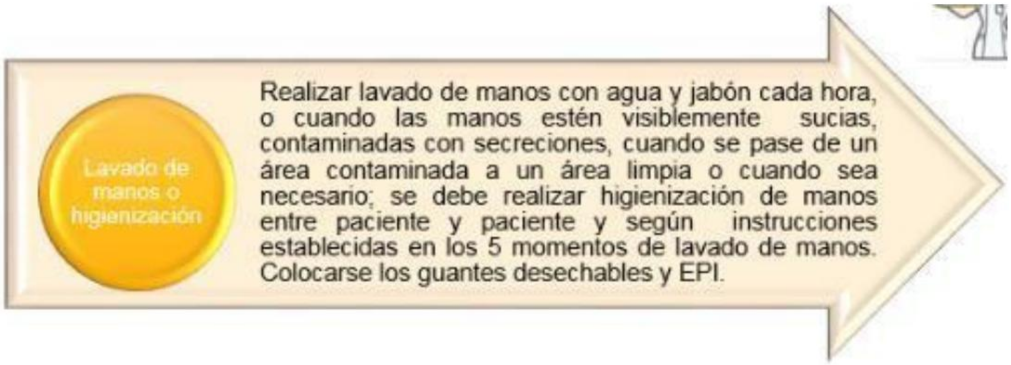

4. SAMPLE TAKING IN INPATIENTS AND EMERGENCIES

The clinical laboratory takes samples from patients in the emergency department. For this, there are two assistants on duty during the day and night. For patients who are in the resuscitation and pediatrics service, those who take the samples are the clinic's nursing staff, as well as taking gynecological and microbiological samples and accompaniment for collecting urine from toxicologists. For other users, samples must be taken by clinical laboratory assistants.

In the inpatient services, routine examinations by the clinical laboratory have been defined.

For intermediate ICU and adult ICU services, all samples will be taken by nursing staff. Likewise for pediatric patients in any service of the Clinic.

In all cases, the following must be taken into account:

- ÿ Verify requests in the Hosvital information system. In emergencies, search and call outpatients and direct them to the sample collection module.
- ÿ In the case of patients on a stretcher, chair or who cannot move, the mobile assistant must take the sample where the patient is located.
- ÿ Verify order in the system and define the tubes to take. Carry out the procedure taking into account the correct procedures for sample collection:

Machine Translated by Google

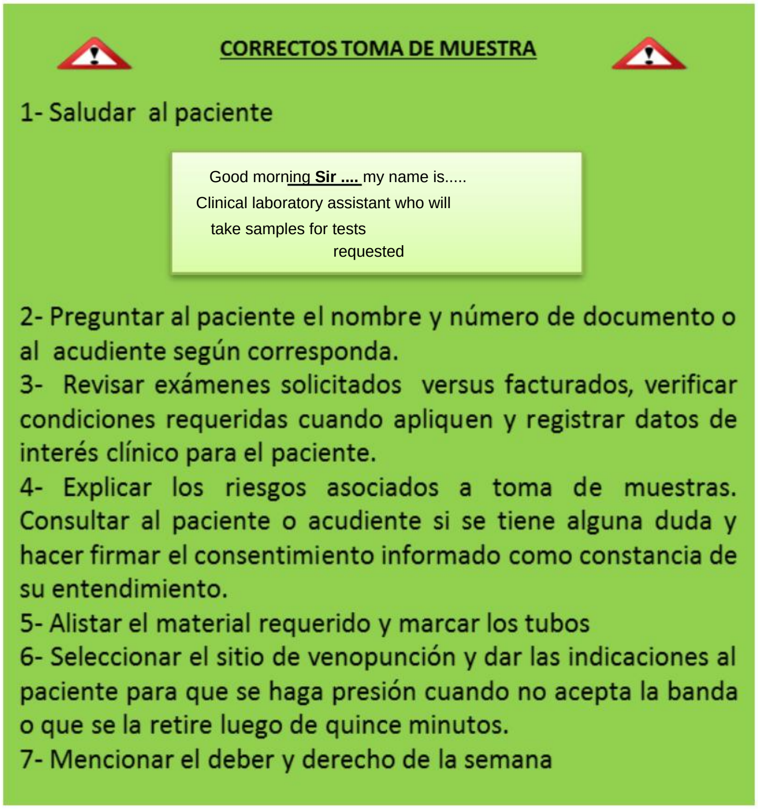

ÿ Always mark the tubes in front of the patient and after verifying the data.
ÿ Take into account the definitions of the laboratory care model.

5. SELECTION OF THE PUNCTURE SITE:

The veins most used for venipuncture are located in the anteulnar area. Among these we have:

- Cubital Vein: It is the longest and thickest of all and is preferred for bordering the muscles. from the arm.
- Cephalic Vein: It has the same characteristics as the previous one, but it is a little less thick.
- Basilic Vein: It is smaller than the previous ones. This vein is close to the brachial artery, so puncture is risky and its area is more sensitive and painful for the patient.

Machine Translated by Google

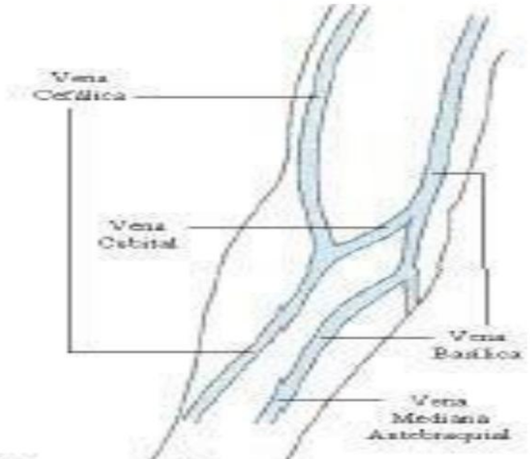

Venas del brazo

- ÿ Place the tourniquet 2 to 3 centimeters above the selected site, to better visualize them. Its use should not last longer than 1 minute.
- ÿ Adopt correct posture: the patient's arm must be placed in a straight line and rest firmly on the armrest or work surface, without bending at the elbow.
  To care for babies, they will lie down on the sample collection table using the mat, asking the mother or companion to hold the baby to prevent it from falling or moving during the sample taking.
- ÿ Areas with bruises, burns, ankles or feet should be avoided in diabetic patients and with circulatory disorders. Select opposite arm in case of post lymph node dissection surgery due to breast surgery.
- ÿ Once the vein has been chosen, carry out asepsis in the area with a cotton pad soaked in alcohol in a circular manner from the center to the periphery, letting the alcohol dry. Place the tip of the needle 1 cm below the selected vein.
- ÿ Puncture the vein with the bevel of the needle facing up in the direction of blood flow.
- ÿ Hold the needle at an angle of 30 to 40 degrees in relation to the skin and cellular tissue subcutaneous to take it to the vein.
- ÿ Once you have performed the puncture, insert the tubes into the holder and/or sleeve so that blood flows freely in the established order.
- ÿ If you use a syringe, tilt the needle until it is almost parallel to the surface of the skin and puncture the vein slowly, verifying penetration by the reflux of blood in the chamber.

Machine Translated by Google

6. COMPLICATIONS

COMPLICACIONES				
**DEFINICIÓN**	**COMO SE EVITA**	**QUE HACER SI SE COMPLICA**	**CAUSAS**	**CLASIFICACIÓN**
**INFECCIÓN O FLEBITIS:** Es la inflamación de la vena debido a una alteración del endotelio, con migración de plaquetas a la zona lesionada, lo que produce liberación de histamina, aumenta el flujo sanguíneo en la zona por vasodilatación, luego hay un aumento de la permeabilidad capilar, lo que permite la extravasación de la proteína y líquidos y se produce un acumulo de linfocitos en el lugar inflamado.La inflamación en la pared de una vena se produce generalmente como consecuencia de una mala circulación o por las malas prácticas clínicas durante los procedimientos de Venopunción y que puede llegar a ser potencialmente peligrosa en la medida de que de la zona afectada se desprenda un trombo.<br>Pueden ser de dos tipos: superficial y profunda siendo estos últimos los menos comunes pero los más graves y difíciles de manejar.<br>Se caracterizan por producir: inflamación local en el sitio de punción, dolor en la parte afectada del cuerpo, enrojecimiento de la piel, calor y sensibilidad sobre la vena.	* Mediante la aplicación de la técnica aséptica adecuada descrita en este instructivo; Cumplir con lo establecido en el protocolo de toma, transporte y conservacion de muestras. *Evitar realizar venopunciones en áreas cercanas a lesiones de piel y realizar en lo posible una sola venopunción<br><br>*.Verificar que antes de la puncion la solución alcoholica haya secado completamente.<br><br>Elija la vena de mejor calibre y acorde con la aguja a utilizar.Se debe elegir la aguja de menor calibre con respecto a la vena, que cumpla con el objetivo de la punción de modo que se evite trauma o irritación local. Evite utilizar venas que:<br>a)Se encuentren en zonas de flexión.<br>b)Sean muy visibles, ya que tienden a desplazarse apartándose de la aguja.<br>c)Estén dañadas por uso previo, flebitis, infiltración o esclerosis. d)Estén continuamente distendidas con sangre, o que se hayan vuelto nudosas o tortuosas.<br>e)Se encuentren en una extremidad lesionada o quirúrgicamente comprometida, debido a una posible alteración de la circulación y molestias para el paciente.<br>Verificar que la piel del sitio de punción debe estar intacta, sin rastros de eritemas, erritaciones y/o escoriaciones. I<br>De preferencia se deben utilizar las venas de las extremidades superiores (VER CAPITULO 3 DEL PRESENTE PROTOCOLO)	Solicitar a atención médica	• Manipulación: Barreras de seguridad ineficaces.<br>• La condición clínica del paciente.<br>• La condición de la vena.<br>* Aplicación de inadecuada Técnica de punción: Uso incorrecto de procedimiento de asepsia y ejecución de multipunciones.<br>• Fijación: Falla en fijación de la vena. (Recuerde, estabilice la vena manteniendo la piel tensa en sentido contrario a la dirección de la punción, con el fin de lograr la colocación atraumática de la aguja, coloque el bisel de la aguja sobre la vena y hacia arriba, en un ángulo de 30 a 45 grados y penetre en dirección del flujo venoso y mantenga fija la aguja sin realizar movimientos bruscos para minimizar el traumatismo de la pares del vaso, e inserte el dispositivo de vacío para la colección de la muestra.)	Presenta tres factores asociados:<br>• Infecciosa: se evidencia después de 72 horas. Inflamación originada por cualquier tipo de microorganismos causada por falta de aplicación de una adecuada técnica aséptica.<br>• Química: irritación de la vena por soluciones acidas, alcalinas o hipertónicas (no aplica en la práctica de toma de muestras).<br>• Mecánica: por mala manipulación, inadecuado sitio de inserción (zona de flexión, tortuosidad de la vena). Vena multipuncionada, fallas en la técnica de punción, condición de la vena.

COMPLICACIONES				
**DEFINICIÓN**	**COMO SE EVITA**	**QUE HACER SI SE COMPLICA**	**CAUSAS**	**CLASIFICACIÓN**
**HEMATOMA:** Acumulación de sangre que se forma cuando hay una lesión en los vasos sanguíneos pequeños que causa sangrado dentro de los tejidos.	• Técnica adecuada de venopunción donde se perfore la vena solo un sitio.<br>• Elegir una vena con buen tejido de soporte.<br>• Liberar rápidamente el tornique usar agujas con buen bisel, el cual debe colocarse hacia arriba para la punción y del mínimo o calibre posible.	• Suspender la punción.<br>• Presionar directamente sobre el sitio de la punción con un algodón seco, sin alcohol, ejerciendo presión firme y constante durante mínimo tres minutos.<br>• Si el hematoma continuo aumentando con estas medidas se llama al médico.	* Si el paciente presiona firmemente la zona de la extracción, en esas circunstancias factores como: Venas frágiles, Extracciones dificultosas por venas inaccesibles o muy finas<br><br>* Medicamentos como la aspirina o los anticoagulantes.<br>* En las siguientes patologias como hemofilia, enfermedad de von Willebrand, trombocitopenia.	NO APLICA

Machine Translated by Google

COMPLICACIONES				
**DEFINICIÓN**	**COMO SE EVITA**	**QUE HACER SI SE COMPLICA**	**CAUSAS**	**CLASIFICACIÓN**
**DOLOR:** La sensación de dolor es muy subjetiva y el paciente puede quejarse poco o mucho, aunque el trauma o el daño en la piel o tejido sea mínimo.	Para poder evitarlo o disminuirlo el laboratorio debe tener muy clara el protocolo de toma de muestras donde se indica que se le debe explicar muy claramente el procedimiento al paciente, verificar la buena calidad y estado de los elementos usados.	El dolor nunca debe subestimarse por su cronicidad y puede ser un motivo de consulta de urgencia	El laboratorio además debe contar con un proceso estandarizado, con personal suficientemente entrenado y hábil para realizar el procedimiento.	NO APLICA
**HEMORRAGIA O SANGRADO VAGINAL:** Es cualquier sangrado vaginal no relacionado con mestruación. Este tipo de sangrado puede incluir un manchado leve entre periodos. Puede proceder de cualquier sitio del tracto genital, incluyendo vulva, la vagina, el cuello uterino y el útero.	Capacitación en toma de muestras y retroalimentación constante al auxiliar de laboratorio o al personal hospitalario. Indagar correctamente al paciente, si es virgen o con sangrados previos	Se debe suspender la toma de muestra (puncion o toma de muestra especial). Presionar directamente sobre el sitio de punción con algodón seco. Solicitar cita mèdica recurriendo a colaborador o administrador designado si el caso lo amerita.	Se produce por falla en la técnica de la toma de la muestra de secreción vaginal	NO APLICA
**CORTADA:** Herida hecha con un cuchillo o un objeto cortante.	Utilizar los elementos de acuerdo las especificaiones del fabricante y a los implementos requeridos. (bisturi con su mango)	Suspender toma de muestra punción o toma de muestra especial. Presionar directamente sobre el sitio de la punción minimo tres segundos con algodón seco. Levantar la extremidad. Solicitar cita médica recurriendo a colaborador o administrador designado si el caso lo amerita	Toma de muestras de hongos con bisturí	NO APLICA

Machine Translated by Google

7. LABELING AND MARKING OF SAMPLES:

The Laboratory software automatically issues, at the time of entering the DataLab Enterprise application, a series of self-adhesive labels with barcode, which includes the demographic information of: patient name, assigned reference number and the number and name of the type. of container to drink.

When the user is in the sample collection module, the Laboratory assistant prepares the material according to the tests requested by the doctor, and manually marks the tubes with the correct ones established institutionally for sample marking: names and surnames. complete details, identification number and time of sample collection. Then adhere the label (bar code sticker) to the tubes vertically, first verifying the correct identification of the patient. Leaving the patient's name uncovered to be able to perform a double marking check before processing the sample but also leaving a visible window to verify the quality of the serum

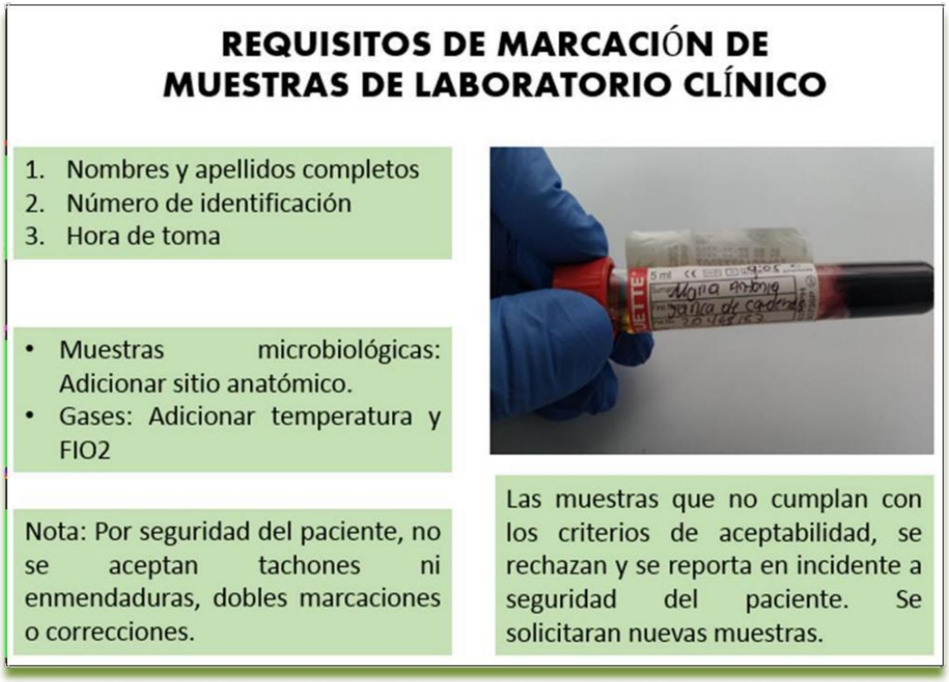

These marking criteria are mandatory for all clinic personnel who take samples for clinical laboratories. These criteria are standardized in order to minimize error, guaranteeing patient safety and the quality of the processes. The correct identification of the samples is led by the human talent of the institution who have the function of guaranteeing that the identification of the sample is a variable with zero errors.

When this fails and it is not possible to take a new sample, there is the possibility of filling out the **LC.01.MA.02.FT.03** Responsibility format for samples with labeling errors, which commits the doctor and the head nurse in charge to certify that the sample is from the patient from whom the tests were requested, and that they assume responsibility in the event that the sample does not meet any acceptance criteria for the requested test.

Machine Translated by Google

The use of this format is defined **only** for excisional biopsy samples and others from major procedures that involve sedation or greater risk for the patient. For other types of sample, they must always be rejected, a new sample requested and a report generated for patient safety.

This form must be completed in its entirety and delivered to the clinical laboratory service for entry and processing. In all cases, a patient safety report must be generated to perform analysis and define an improvement plan.

8. TECHNICAL SHEET FOR SAMPLE TAKING

FICHA TECNICA PARA TOMA DE MUESTRAS
LABORATORIO CLINICO COMPENSAR

compensar | salud

**TIPO DE MUESTRA:** Sanguínea	**ORIGEN:** Venosa, arterial, capilar
**POBLACION:** Lactantes, niños y adultos	**ROL:** Auxiliar de Laboratorio o Bacteriologo
**MATERIALES**	Ø Guantes desechables
	Ø Torundas de algodón
	Ø Alcohol de 70º
	Ø Curitas (según procedimiento)
	Ø Tubos (De acuerdo al requerimiento de la orden médica)
	Ø Torniquete (según procedimiento)
	Ø Aguja y camisa (según procedimiento)
	Ø Visualizador de venas: en las sedes de alto y mediano volumen de pacientes se utiliza el equipo en pacientes de difícil acceso venoso
	Ø Jeringas, equipo alado (según procedimiento)
	Ø Jeringas heparinizadas con heparina de litio (según procedimiento)
	Ø Lancetas retractil para tecnología POCT.
	Ø Lanceta para prueba rápida (según procedimiento)
	Ø Colchoneta pediátrica (pacientes pediátricos)
	Ø Etiquetas identificativas o rótulos con los datos de identificación del paciente
**ANTES DE REALIZAR LA TOMA**	
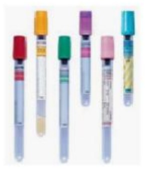	Aplicar los correctos de toma de muestra
	Verificar la identificación del paciente (preguntando al paciente competente o a su acompañante y confrontando la información recibida contra la orden médica), si existen discrepancias consultar el caso con administrador de la sede.
	Explicar a los padres y/o acudientes del paciente o al paciente si es mayor de edad competente, el procedimiento que se va a realizar.
	Alistar el material de acuerdo al requerimiento de los examenes solicitados en la orden, rotular los tubos de acuerdo a protocolo, mantener unicamente los elementos necesarios en el cubículo para la toma de muestras.
	Diligenciar documentación pertinente y/o consulta de instructivos. Indicar al paciente la fecha en que puede venir a reclamar los resultados.
	Realizar lavado de manos con agua y jabón cada hora, cuando las manos estén sucias, contaminadas con secreciones, cuando se pase de un área contaminada a un area limpia o cuando sea necesario; se debe realizar higienización de manos cuando se requiera según instrucciones establecidas en los 5 momentos de lavado de manos.
	Colocarse los guantes desechables y EPI.
	Indicar al paciente la posición que debe adoptar para la adecuada toma de muestra, si es paciente pediátrico ubicarlo en la colchoneta destindada para este fin solicitando la colaboaración de los padres o acudientes del menor.
**SELECCIÓN DEL SITIO DE PUNCIÓN**	
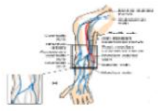 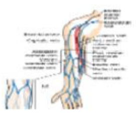	Solicitar al paciente que extienda el brazo y visualizar el sitio de punción.
	* Vena cubital * Vena cefalica * Vena basilica * Fosa antecubital * Antebrazo (vena radial) * Dorso de la mano
	El sitio ideal para realizar el procedimiento de venopunción es la fosa antecubital, que es el área de cualquiera de los brazos anterior (frente a) e inferior a la curva del codo, donde una serie de venas grandes se encuentran relativamente cerca de la superficie de la piel. Cuando las venas antecubitales no son de fácil venopuncion, es aceptable usar las venas ubicadas en el dorso de la mano (no ubicadas en la parte inferior de la muñeca). No se aconsejable usar sangre arterial si el requerimiento es sangre venosa.
	La vena a elección es: **La vena media y vena media cubital.** Como segunda y tercera opcion se pueden usar las venas cefalica y basilica. Estas pueden ubicarse en forma de H o M segun grafica anexa.
	Visualizar el sitio anatomico para realizar la venopunción.
	Idealmente el paciente debe mantener la mano cerrada constantemente ya que cuando se mantienen abiertas las manos se reduce la cantidad de presión venosa a medida que los musculos se relajan.
	Ubicar el torniquete con una presión adecuada, este se debe colocar sobre la superficie de la piel a unos 5 cm por encima de la punción con el fin de fijar la vena.
	El sitio de punción no se debe palpar luego de haber realizado proceso de desinfección según protocolo.
**DESINFECCION SITIO DE PUNCIÓN**	
	Se debe realizar con algodón impregnado al 70% realizando un movimiento de fricción del centro a la periferia (durante minimo 30 segundos), posterior a la realización de esta desinfeccion se debe dejar secar el acohol al aire libre.

Machine Translated by Google

	**USO DEL TORNIQUETE**
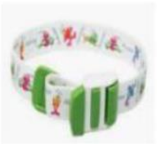	Los torniquetes se deben descartar cuando esten visiblemente contaminados con fluidos biológicos.
	Los torniquetes se deben segùn instructivo INS_PSS_168.
	El torniquete se debe colocar alrededor del brazo con una distancia de 7,5-10 cm sobre el sitio de la venopunción.
	El torniquete se debe retirar cuando se observe que la sangre fluye dentro del tubo, idealmente debe estar colocado sobre el brazo por un tiempo no superior a 10 segundos.
	**Torniquete Pronto**: En el caso del torniquete Pronto se debe cerrar y halar la cintilla para realizar presión con el torniquete, al obtener el retorno sanguíneo se debe desatar lentamente, pulsar y abrir para retirar del brazo. 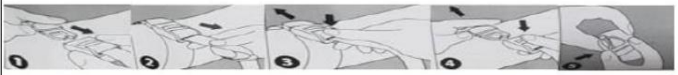
	**USO DE GUANTES**
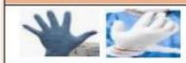	Los guantes se deben cambiar cada vez que se atienda un paciente.
	**USO TORUNDAS DE ALGODÓN**
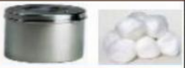	Se deben colocar en el sitio de la punción al retirar el sistema de extracción sanguínea ejerciendo una presión media sin que el paciente en ningún momento doble el brazo para reemplazar está presión. Las torundas de algodòn deben permanecer en las algodoneras.
	**USO DE BANDA PLASTICA ADHESIVA (CURAS REDONDAS)**
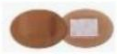	Se debe verificar que el flujo sanguineo haya cesado y proceder a colocar la banda plástica en el sitio de la venopunción de modo que la almohadilla de la cura quede sobre el sitio de la punción. Esta banda adhesiva debe ser retirada por el paciente 15 minutos después de colocada. No se deben usar en personas alergicas al latex.
	**NO OBTENCION DE MUESTRA TRAS PROCESO DE VENOPUNCION**
	Cambiar la posición de la aguja: Cuando la aguja ha penetrado lejos del interior de la vena, es necesario halar un poco hacia atrás la aguja, rotar la aguja para reubicarla lateralmente en intentar nuevamente extraer la muestra de la vena con un nuevo tubo que garantice el vacio. Este procedimiento puede generar dolor, hematoma e incluso perforación de arterias adyacentes por lo que solo se usa en casos de extrema necesidad. Este procedimiento no se debe realizar mas de dos veces, si esto ocurre se debe acudir a otra persona que tenga amplia experiencia en procesos de dificil venopunción.
	**CRITERIOS DE RECHAZO**
	Muestra insuficiente, muestra hemolizada, recolección en tubo inadecuado, muestra mal identificada, muestra coagulada en tubo que contenga anticoagulante (Tubo tapa azul, tapa lila, tapa verde) o en jeringa heparinizada.

9. ARTERIAL GASES SAMPLING

Before puncture, the Allen test is recommended to evaluate the perfusion and saturation of the artery. Ask the patient to clench his fist for 30 seconds, then press both arteries in the patient's forearm, the ulnar and the radial, with his fingers. Next release the pressure on the ulnar artery. Repeat the test releasing pressure on the radial artery. Capillary return should appear within 5 seconds. If after this time the hand is still pale, the test result is positive and indicates an alteration of the blood supply, in this case the artery of that extremity should not be punctured.

Machine Translated by Google

FICHA TÉCNICA PARA TOMA DE MUESTRAS LABORATORIO CLINICO COMPENSAR

compensar | salud

**TIPO DE MUESTRA:** Sanguínea	**ORIGEN:** Arterial
**POBLACION:** General	**ROL:** Auxiliar de Laboratorio y/o Enfermería, fisioterapeuta

**MATERIALES**	Ø Guantes desechables
	Ø Torundas de algodón
	Ø Alcohol de 70º
	Ø Curitas
	Ø Gasas
	Ø Jeringas heparinizadas con heparina de litio.
	Ø Etiquetas identificativas o rótulos con los datos de identificación del paciente

ANTES DE REALIZAR LA TOMA

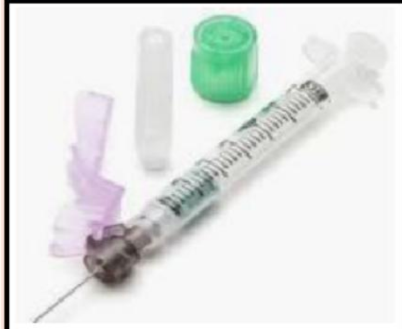

- Aplicar instrucciones descritas en la ficha Generalidades Toma de muestras sanguínea.
- Realizar lavado de manos con agua y jabón cada hora, cuando las manos estén sucias, contaminadas con secreciones, cuando se pase de un área contaminada a un área limpia o cuando sea necesario; se debe realizar higienización de manos cuando se requiera según instrucciones establecidas en los 5 momentos de lavado de manos.
- Colocarse los guantes desechables y EPI.
- Indicar al paciente la posición que debe adoptar para la adecuada toma de muestra.

SELECCIÓN DEL SITIO DE PUNCIÓN

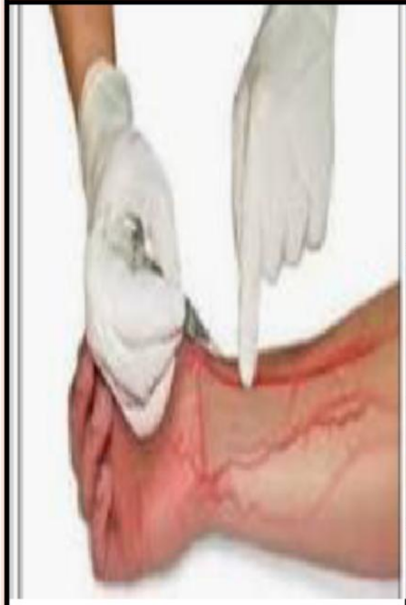

**1. Arteria Radial (Arteria de elecciòn ideal para la toma de muestra)**	Coloque el dedo indice y medio sobre la arteria radial a la altura de la muñeca presionando suavemente para sentir las pulsaciones en la arteria.
	Cerca del área de flexión de la muñeca, entre el apéndice estiloides del húmero y el tendón del flexor radial de la mano (preferiblemente la extremidad no dominante) Sujetar la arteria entre las yemas de los dedos.
**2. Arteria Femoral**	Por debajo del ligamento inguinal, generalmente en el pliegue inguinal (la arteria es lateral a la vena y medial al nervio). De está arteria **Únicamente se debe tomar esta muestra en ambiente hospitalario.**
**3. Arteria Braquial**	En el pliegue del codo (lugar no recomendado por riesgo de hematoma, que a su vez puede comprimir el nervio; se prefiere la extremidad no dominante). De está arteria **Únicamente se debe tomar esta muestra en ambiente hospitalario.**

Al identificar el sitio realice el procedimiento de asepsia de manera circular del centro a la periferia con alcohol isopropilico al 70%.

Machine Translated by Google

<table>
<tr><th colspan="2">TÉCNICA DE EXTRACCION</th></tr>
<tr><td rowspan="3">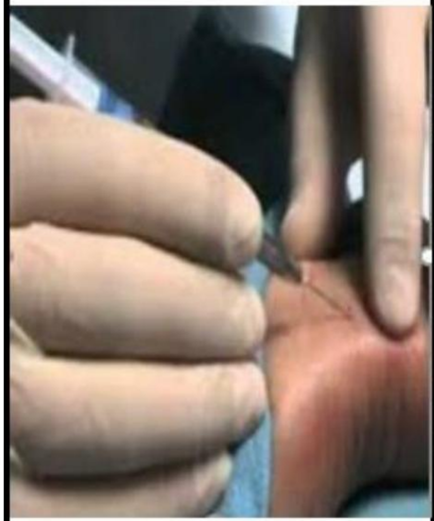</td><td>Se debe dejar secar el alcohol al aire.</td></tr>
<tr><td>Insertar la aguja bajo el ángulo de 45°. Tras la aparición del flujo de sangre pulsátil en la jeringa, extraer 1 ml de sangre, tirando delicada y lentamente del émbolo de la jeringa se debe tener cuidado para no succionar aire a la jeringa.</td></tr>
<tr><td>Después de extraer la sangre, en el mismo instante en que se retira la aguja, se coloca una torunda de algodón seco en el sitio de la punción realizando una presión constante, descartar la aguja según protocolo de bioseguridad, tapar la jeringa con el tapón de caucho, mezclar el contenido de la jeringa 5 veces por inversión, proceder a su procesamiento y/o transporte interno en la sede, (sedes que cuenten con analizador de gases arteriales y venosos), descartar los dispositivos usados según protocolo de bioseguridad. Para el caso de la jeringa, esta se debe descartar después del procesamiento de la muestra, retirar la torunda de algodón verificando que haya cesado el flujo de sangre, este procedimiento debe ser supervisado por el personal que toma la muestra, indicando al paciente que puede retirar la torunda cuando haya cesado el flujo sanguíneo.</td></tr>
</table>

10. CLINICAL LABORATORY SERVICES INFORMATION SHEET

Compensar Clinical Laboratory has two information sheets, the examination sheet for its own processing and for counter-reference. They are for free consumption by the compensation staff from the intranet. These sheets contain information related to the patient's conditions, types of samples, required tubes, storage conditions and stability, and reporting time. Whenever required, laboratory personnel will consult this information to provide it to patients or nursing.

11. GENERAL RULES ESTABLISHED FOR EACH TYPE OF SPECIMEN:

The specimens must be received by laboratory personnel in the shortest possible time once extracted or collected:

- Blood: During transport, agitation should be avoided (due to possible hemolysis) and they should be protected from direct exposure to light (due to the degradation of some constituents, such as bilirubin). For the determination of some parameters unstable (lactate, ammonium, plasma renin, acid phosphatase), the specimens should be kept refrigerated at 2 to 8°C, immediately after collection, and should be transported refrigerated. Blood tubes should be upright during its transport, with the cap facing up, which favors the complete formation of the clot and reduces the agitation of the contents of the tube.

- Secretions and Body Fluids: Collect samples in bottles or tubes with

Machine Translated by Google

anticoagulant for cell counting, except for CSF, for which a sterile glass tube is used, without accessories for cultures and biochemical examination. They should not be refrigerated or frozen before growing.

- Urine: Specimens for urinalysis are collected and transported in sterile, disposable plastic containers. Urine from pediatric patients is collected in flexible polyethylene bags, which can be sealed for transport.
- Feces: collected in special containers for stool samples.

Note:

- It is important to keep in mind that blood, urine and fecal matter samples should be transported as soon as possible (within 90 minutes, taking into account the recommendations for each of the tests according to test inserts).
- For arterial and venous gas samples, they must be transported to the laboratory within 15 minutes.
- For microbiology samples, transportation is recommended within the first fifteen minutes and a maximum of two hours.

12. CRITERIA FOR REQUESTING NEW SAMPLES/NEW PATIENTS

The establishment of acceptance-rejection criteria for specimens or samples that arrive at the laboratory must be one of the measures to be taken to establish an adequate quality system to guarantee patient safety.

The defined management is not to process if a new sample can be obtained or to process, recording at the time of validating the result, a comment on the quality of the sample in case of a patient with special mental abilities, with some degree of disability or difficult access. venous. In all cases we will try to facilitate the process so that the patient can access the service.

Patients who do not meet the test requirements are not suitable for sample collection since this directly affects the results of the exams:

- **Fasting:** the patient must have had his last meal between 8-10 hours before, after which he must not have ingested anything including chewing gum, cigarettes, water, red wine.
- **Diet:** When the test requires it, the patient must refrain from consuming certain foods and practice activities that directly influence the measurement of the analyte.
- **Medications:** If medication levels are controlled, the patient must NOT have taken/applied them, they will do so after taking the sample.
- **Inadequate container:** Fecal matter, isolated or 24-hour urine samples will be collected in the containers provided by the Laboratory for this purpose or can be purchased at a drugstore. For no reason will samples be received in containers of dairy products, preserves or similar.

When it is definitely not possible for the patient to be treated due to the aforementioned causes, the situation will be explained to them and it will be made clear that they can present themselves at the laboratory in the following business days (without exceeding one month) at the usual time. Patients must be redirected to access where the inattention indicator will be recorded and

Machine Translated by Google

They clarify the billing issue.

In addition to the causes of inattention already mentioned, the following will be taken into account for patients in inpatient services:

- **Samples without a medical order:** Samples that are difficult to obtain, deteriorate quickly or whose result depends on rapid decision making will be processed without order (gas, troponin, hematocrit, hemoglobin). The Clinic will undertake to comply with the administrative requirements subsequently. Samples that are not considered vitally urgent must always be accompanied by a medical order.

- **Use of inappropriate tube or input:** Depending on the type of test, the sample must Take in the indicated container.

- **Incorrect sample volume:** This sample rejection criterion is critical in analyzes that require samples taken in tubes with anticoagulants, such as the determination of coagulation tests or in the erythrocyte sedimentation rate and blood counts, since it must be maintained the exact proportion between the sample volume and the anticoagulant volume. The vacuum filling tubes are prepared so that the sample volume that enters is correct. When filling the tubes with a syringe, caution must be taken for determinations that require this proportion to be exact.

- **Hemolysis:** It occurs for different reasons that must be avoided: Difficult venipuncture, incorrect handling of the specimen obtained, consequence of a disease that produces in vivo destruction of erythrocytes. The degree of hemolysis interference depends on its intensity, the actual concentration of the analyte and the methodology used.
  Compensar Clinical Laboratory has a reagent that measures the hemolysis index present in the sample and depending on the analyte to be processed, the sample will be accepted or rejected taking into account the result of the hemolysis index and the degree of interference in the analyte.

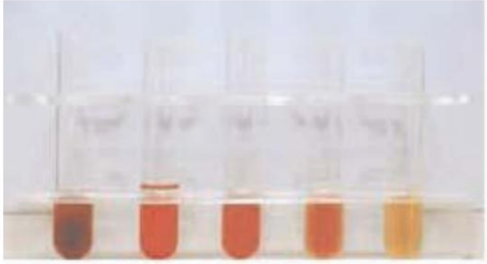

Imagen 12: muestras con diferentes grados de hemólisis.

- **Lipemic sample:** It is a plasma or serum sample with high fat content.
  It has a whitish appearance, and may be due to the extraction of a sample from a patient on parenteral nutrition or after a copious ingestion. There are determinations whose results are altered when this condition exists.

Machine Translated by Google

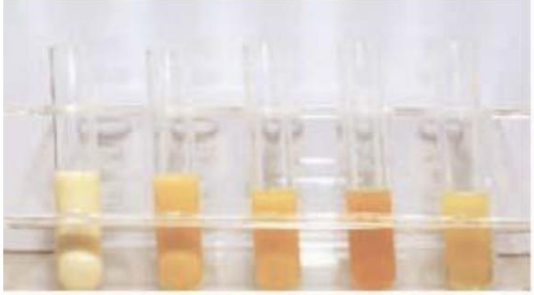

Imagen 15: muestras con diferentes grados de lipemia.

- **Clotted sample:** That sample that is partially or completely clotted and that was extracted with anticoagulant in the tube. Clotting may be due to slow extraction, improper mixing of the anticoagulant with the sample, or a defect in the anticoagulant itself.

- **Misidentified sample:** Samples must be identified with: Full names and surnames, identification number and time of collection in legible writing without erasures or amendments. Failure to comply with these criteria generates rejection of the samples.

- **Inadequate transport temperature:** There are determinations that can only be made under strict pre-analytical temperature conservation conditions. For example, lactic acid, gases, ammonium or homocysteine require refrigerated transport of the specimen, while cryoglobulins require that this transport seeks to maintain body temperature. In most analytes, temperature is a continuous variable and therefore does not affect abruptly, but rather gradually decreases the quality of the sample the further it moves away from the optimal transport or storage temperature.

- **Samples with retracted clot:** the samples must be sent to the laboratory in the shortest possible time once taken, to avoid this phenomenon, which is evidenced by the separation of erythrocytes from the serum, this could affect the determinations due to the degradation of the sample. .

- **Insufficient sample:** That sample to which not all the tests can be carried out. determinations requested when the specimen is exhausted.

- **Deteriorated sample in the Laboratory:** That sample that, coming correctly, deteriorates in the preparation process: fragmentation of the tube in the centrifuge, accidental spillage of the sample or fall of the tube.

Samples that have cause for rejection will not be processed, they will be retained in the laboratory and the respective service will be called to notify of such circumstance.

Requests for new samples for these reasons generate incidents that must be reported to patient safety through the curuba system. In addition, they must be recorded in the matrix defined for this purpose in order to record the monthly indicator.

Machine Translated by Google

13. GENERALITIES BY TYPE OF SAMPLE

13.1 SAMPLING FOR PRE AND POST GLUCOSE LOADING - GLUCOSE TOLERANCE CURVE - DEO'SULLIVAN TEST.

Tests taken on a blood sample, used for the possible diagnosis of diabetes mellitus, hypoglycemic states or gestational diabetes.

- The patient must present on an empty stomach before 9:00 am and have time for the completion of the exam.
- Take sample on an empty stomach, mark the tube with the sticker corresponding to the baseline.
- Inform the patient of the risks associated with venipuncture and ingestion of the glucose load, then request the completion and signing of the related consents.
- Perform glucometry: a drop of peripheral blood is placed on the glucometry strip and introduced into the glucometer until the blood glucose value is shown on the screen. (see user manual for handling the glucometer depending on the commercial company used.)
- If the glucometry is less than 130 mg/dl, give the glucose load according to the test ordered and type of patient. If the glucometry is greater than 130 mg/dl, instruct the patient to eat a normal breakfast and notify the Chemistry Bacteriologist to make the corresponding note to validate the result.
- Deliver in writing the schedule for taking the following samples to the patient and tell them that they cannot eat any drink or food, not smoke, not do any type of physical activity, not chew gum and remain in the laboratory waiting room.

To manage the glucose load, follow the following recommendations:

LABORATORIO CLINICO COMPENSAR — compensar

ADMINISTRACION CARGAS DE GLUCOSA

SOLICITUD	CARGA	GLUCOMETRIA	MUESTRA BASAL	MUESTRA POST 2 HORAS	MUESTRA 1/2 HORA	MUESTRA 1 HORA	MUESTRA 2 HORAS	MUESTRA 3 HORAS	MUESTRA 4 HORAS	MUESTRA 5 HORAS
Pre y post prandial	NA, Desayuno habitual	NA	X	X						
Pre y post carga	75 gr en 200 mL de Agua	X	X	X						
Curva de glicemia	75 gr en 200 mL de Agua	X	X		X	X	X	X		
Curva de glicemia 4 horas	75 gr en 200 mL de Agua	X	X		X	X	X	X	X	
Curva de glicemia 5 horas	75 gr en 200 mL de Agua	X	X		X	X	X	X	X	X

Machine Translated by Google

PROTOCOLO PREPARACION DE CARGAS DE GLUCOSA			
**POBLACIÓN**	**CANTIDAD DE GLUCOSA EN CC**	**CANTIDAD DE AGUA EN CC**	**VOLUMEN FINAL**
GESTANTES 75gr DE GLUCOSA CURVA DE 2 HORAS 3 MUESTRAS	150	150	300
ADULTOS NO GESTANTES 75gr DE GLUCOSA	150	150	300

PROTOCOLO PREPARACION DE CARGAS DE GLUCOSA			
Equivalencias en mililitros de glucosa para pacientes con curvas de glicemia; pre y post cargas de glucosa con peso inferior a 43 KG			
**PESO EN KILOGRAMOS (Kg)**	**CANTIDAD DE GLUCOSA EN CC**	**CANTIDAD DE AGUA EN CC**	**VOLUMEN FINAL**
10	35	35	70
11	38,5	38,5	77
12	42	42	84
13	45,5	45,5	91
14	49	49	98
15	52,5	52,5	105
16	56	56	112
17	59,5	59,5	119
18	63	63	126
19	66,5	66,5	133
20	70	70	140
21	73,5	73,5	147
22	77	77	154
23	80,5	80,5	161
24	84	84	168
25	87,5	87,5	175
26	91	91	182
27	94,5	94,5	189
28	98	98	196
29	101,5	101,5	203
30	105	105	210
31	108,5	108,5	217
32	112	112	224
33	115,5	115,5	231
34	119	119	238
35	122,5	122,5	245
36	126	126	252
37	129,5	129,5	259
38	133	133	266
39	136,5	136,5	273
40	140	140	280
41	143,5	143,5	287
42	147	147	294
43	150,5	150,5	301

Machine Translated by Google

For the use of glucose loading as a medicine, the following must be taken into account: correct related below:

13.2 SAMPLING FOR D-XYLOSE:

To take a sample for the D-Xylose test, take into account the following recommendations: At the time the patient requests information about the test and taking into account if they are a child and/or under 18 years of age, take the data on: Identification, age, size weight; Send the data by email to the bacteriologist in charge of referrals at the CPL or Salitre headquarters and/or call 4285088 ext. 11515,11502, for the reference laboratory to send the required load for the patient, which will be available on the day the patient comes to take their exam.
Samples are taken only Monday through Friday and must be presented to the laboratory at 6:30 am. Allow 1.5 hours if you are a minor or 5 hours if you are an adult.
Fasting for no more than 12 hours and having had your last meal before 11:00 pm the previous day.

Two (2) days prior to taking the exam, you must follow a diet free of: gelatins, fruits, jellies, desserts.
On the day of the exam, minor patients must bring 2 ounces of water measured in the container in which the child usually drinks liquids (bottle, glass, glass - straw), to dilute the load there and indicate its intake. complete within a maximum of 20 minutes.
Take a blood sample 1 hour after ingestion of the xylose load.

Patients over 18 years of age are administered a load of 5 grams of xylose in 500 ml of water. The patient must collect urine within five hours, counting immediately after ingesting the load. The collection is done in a clean plastic container. of one (1) liter supplied by the laboratory.

13.3 BIOLOGICAL LIQUIDS (See microbiology manual)

ÿ **Cerebrospinal fluid:** The correct diagnosis and treatment of a CNS disease may depend on the results of CSF examination in the laboratory; Because of this, this liquid has to be extracted and handled correctly.

CSF is normally sterile and can be obtained by lumbar puncture or, less commonly, by cisternal, cervical, or ventricular puncture; Each of these procedures must be performed aseptically by a doctor with experience in the same and the patient must be alerted about its indications and possible complications. The puncture area must be marked and disinfected. The amount collected will depend on the clinical situation; when looking for tumor cells, it is important to obtain as much cerebrospinal fluid as possible.

In order to avoid contamination of the sample, cerebrospinal fluid must be obtained and transported in closed tubes. The cerebrospinal fluid should be distributed, under aseptic conditions, into several transparent tubes with screw caps (sterile and without additives). It is very important after taking the sample to send it to the laboratory as soon as possible to avoid cellular deterioration.

Sometimes it is useful to collect samples in different portions indicating the sequence.

Machine Translated by Google

filling since it facilitates the determination of the origin of possible red blood cells.

The use of talc-powdered gloves is not advisable when collecting cerebrospinal fluid, as it could disturb the cytological examination of the cerebrospinal fluid.

ÿ **Serous fluids:** Pleural, pericardial and peritoneal fluids: Serous fluids are body fluids that derive from plasma and are found in the pleural, pericardial and peritoneal cavities. Serous fluids are ultrafiltrated plasma derived from the abundant capillary network of the serous membrane.

The fluid in the peritoneal cavity is usually called ascites fluid.
For biochemistry studies, the liquid must be collected in a sterile container with a screw cap. For cytology studies, the same procedure will be followed but using the tube with EDTA as an anticoagulant. If Adenosin Aminosine (ADA) is ordered, the tube must be taken. Sodium Heparin (Green Cap)

ÿ **Synovial fluid:** Disorders of the synovial membrane, alterations in the joint support elements and the presence of foreign bodies can cause the accumulation of large amounts of synovial fluid in the joints. Its subsequent analysis in the laboratory can be decisive for the diagnosis of the underlying pathology. Obtaining the liquid must be done with a syringe without anticoagulant. Once the sample has been collected, depending on the volume obtained, it must be distributed in the different containers necessary to carry out its study: Sterile tube for microbiological examination, Tube without additives for the study of crystals and biochemistry, Heparinized tube or with EDTA for cell counting. .

ÿ **Seminal fluid:** Instructions on sample collection and the pre-analytical conditions to be followed will be provided to the patient by both the doctor requesting the analysis and the laboratory: Abstinence period: Before collecting the semen sample to be analyzed , sexual abstinence must be maintained for a period between 3 and 5 days (and no more than 7 days), which implies not having any loss of semen due to intercourse, masturbation, nocturnal pollution or any other circumstance during these days. If the abstinence period is less than 48 hours, the sample should be considered invalid for study. Hygienic measures: It is important to avoid possible contamination of the sample. Wash your penis with soap and rinse thoroughly with water to avoid soap residue. No type of cream should be applied. Collect the sample in a sterile wide-mouth plastic bottle, closing it with its lid after obtaining the semen (make sure it is tightly closed). Avoid temperature changes that occur during transport.

Obtaining the sample: The sample must be obtained by masturbation or the use of a special collector that does not contain lubricants or spermicides used during intercourse in the case of spermograms. For cultures, the sample must be collected directly into a sterile bottle (urine sample containers). Ordinary condoms cannot be used because they contain lubricants and spermicides and “coitus interruptus” is unacceptable because the first fraction, rich in sperm, can be easily lost.

It is important to collect the entire content of the ejaculate. If any amount, no matter how small, is lost or spilled, the patient must notify the laboratory staff since the sample would not be valid for the fertility study.

Machine Translated by Google

etc.). It is also important to know if there have been significant clinical changes in the patient (fever, drug use, etc.) in the previous days or weeks.

Sample collection for Spermogram: To collect this sample, an office is available.

- It is recommended to have sexual abstinence of 3 to 5 days, which should never be less than 2 days nor more than 7
- Before obtaining the sample, the patient must wash their hands and genital region thoroughly with soap and water; rinse well and dry with a clean towel, you will need to urinate before collecting the semen sample
- To collect the sample, a special sterile collector must be used which allows the sample to be preserved in conditions suitable for processing.
- If you have had a fever or have undergone recent surgery, you should postpone the exam. approximately 30 days.
- Collect the sample using the collector by intercourse using the special collector avoiding spillage
- Seal the collector carefully, and place it inside another container or a bag. plastic.
- Write down the collection time
- Transport the sample, trying to keep it at a temperature of 35 to 37°C (body temperature).
- Send the sample to the laboratory as soon as possible

Note: The sample stored at 35 to 37°C can be kept in good condition for up to one hour after collection.

ÿ **Urine:** Urine samples are used by the laboratory to diagnose and monitor the treatment of kidney or urinary tract diseases and to detect metabolic or systemic diseases. Sample collection methods and times depend on the tests requested by the doctor.

Isolated urination urine sampling: First thing in the morning urine is preferred since it has a higher osmolarity, which reflects the kidney's ability to concentrate urine. In this first urine, elements such as leukocytes, bacteria, casts, red blood cells are more concentrated, thus optimizing the diagnostic performance of laboratory tests. Isolated urination urine obtained at random is accepted in special situations such as urgent analysis, or certain studies, such as, for example, in the study of bone metabolism, where the second urine in the morning is recommended. Urine should be collected from the middle portion of urination, since it is less contaminated by bacteria from the urinary meatus that are carried away by the first part of urination. It is recommended to first wash the external genitalia with soap and plenty of water, avoiding contamination of urine with soap because certain parameters such as pH may be affected, or even bacterial growth may be inhibited.

Machine Translated by Google

- **Technique for children:** In younger boys and girls (who do not yet control their sphincters), urine will be collected in collectors or sterile bags specially designed for them in the following way: Careful washing of the genitals and perineal area as in adults. .
  Place the plastic bag or sterile collector. Remove the bag as soon as the child has urinated.
  Every 20 minutes the bag must be changed and the process restarted. It is recommended to obtain a minimum urine volume of 8-12 ml for microchemical and sediment analysis. Smaller volumes may be accepted in samples from children or oligo-anuric patients.
- **Technique for adults:** The ideal sample is first in the morning, however, patients admitted for emergencies will have the sample collected at the time of request. In all cases, genital cleansing with soap and water must be carried out beforehand. The first portion of urine is discarded directly into the toilet and the middle part of the urine is subsequently collected. You should not have direct contact of your hands with the interior of the container.
- **Disabled patients or patients with neurogenic bladder:** The caregiver is the one who must take the sample, asepsis the cystophle, let a little urine come out and then let the urine fall into the sterile bottle.

For bacterial culture, a minimum volume of urine is needed (1-10 ml of urine).

ÿ **24 HOUR URINE COLLECTION:** One day must be available for this collection. The patient must have a new plastic container or, failing that, a container that has contained water with a capacity greater than 1000 c/c.

- The patient should not change their daily routine and should not drink more fluids than normal.
- To start the collection you must get up at 6:00 am and discard the first urine of the morning in the toilet; Then you must collect the entire production of urine from that moment, including that at 6:00 am the next day to complete the 24-hour urine. You should not discard any sample
- For women: It should not be collected during the menstrual period.
- If the requested test requires a blood sample, it must be taken on the same day that the 24-hour urine sample is provided.
- Plastic containers that have contained oils, detergents, soft drinks, etc. are not accepted. etc
- It is necessary to measure the volume of the collected urine, record the volume in the appropriate container, save a counter sample in case the sample is sent to CPL or a registered laboratory.
- For inpatient or emergency patients, sample collection can begin at any time of the day. Always keep a record of the start time which must be after vacating the vegija and calculate 24 hours for its completion.

13.4 TAKING BACTERIOLOGICAL SAMPLES

Through the bacteriological study, an etymological diagnosis can be established in a certain infectious process of bacterial origin.
When the clinical laboratory takes bacteriological samples for analysis, it can generally perform direct examination, cultures and antibiograms.
Take into account the causes that may generate inconsistency in the results, for example, contamination in the sampling process, insufficient sample, failures in the preparation of the sample.

Machine Translated by Google

patient, taking samples in places unrelated to the problem, lack of knowledge of information of interest for the diagnosis, patient under antibiotic therapy, inadequate transportation of the sample, delays in the processing of specimens.

Biosafety Standards and the use of personal protection elements must be complied with.

• **VAGINAL SAMPLES**

ÿ Sample taking in outpatient clinic by laboratory assistant. In emergencies and hospitalization in charge of nursing.
ÿ Verify the patient's reading and understanding of Risks in taking Special samples. Proceed to request a signed informed consent.
ÿ Record all related information in comments in Datalab Enterprise
Ask the patient about: Medications she is taking, vaginal suppositories or antiseptics she is applying, not having applied creams or vaginal douches three days before.
Confirm age and date of last menstruation, do not attend if you are menstruating (show up five days before or five days after your period).
Not having had sexual relations three days before taking the sample.
ÿ Direct the patient towards the bathroom or cubicle so that she can remove clothing from the waist to the down and put on the disposable or cloth gown intended for this purpose.
ÿ The sheet is changed to the stretcher, and a disposable field is placed at the bottom of it to position the patient.
ÿ Instruct the patient to lie on the stretcher face up in a gynecological position with her back adequately supported on the stretcher and her feet firmly supported in the stirrups in order to prevent falls.

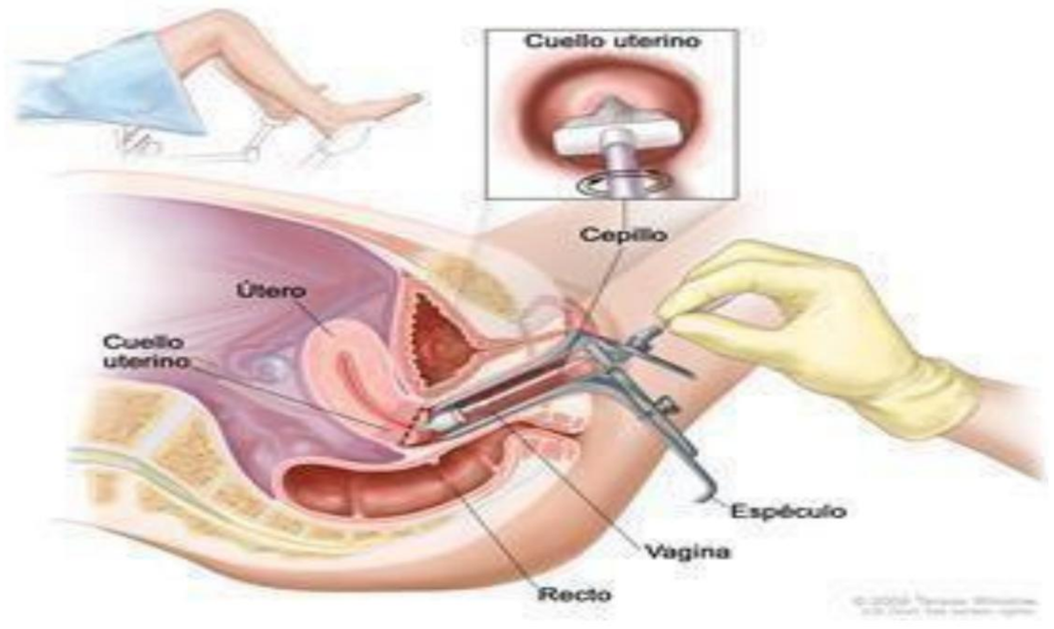

ÿ If it is a girl, explain the procedure to the companion; in this case, or virgin or pregnant patients, only use a toothbrush.
ÿ Do not use lubricants that facilitate the introduction of the speculum.
ÿ Initially take a sample of the endocervix and place it in a sterile tube for transport to the

Machine Translated by Google

laboratory.

ÿ With a second swab, make smears and smears for Gram staining, the slides must be previously marked with the reference number, patient's initials and examination to be performed, the endocervix sample will be located adjacent to the emery of the slide and the exocervix on the right (end of the plate)

ÿ After taking the vaginal sample, the field of disposable material is removed and placed in the red bin. In the same way, the sheet is removed and placed in the dirty material bin found inside the office.

ÿ The stretcher is disinfected using industrial gloves: with a disposable paper towel and soap in use in a sweeping manner, the excess is removed with another paper towel and water and disinfected with antiseptic alcohol. This procedure is done between patient and patient.

ÿ Wash hands and change disposable gloves for each patient.

• SPREAD OF PREPUCIAL BALANO SECRETION OR GENITAL LESION

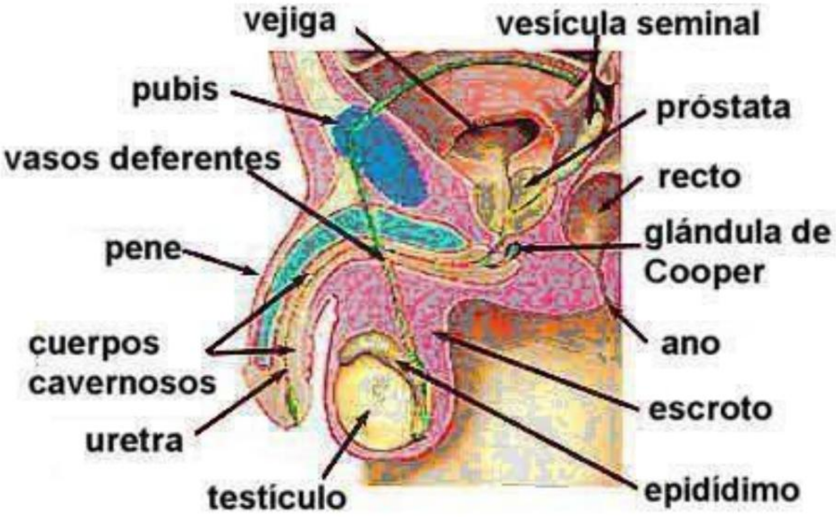

Verify with the patient that they are not using antibiotics or topical treatments, they should not have performed genital cleansing, nor have they had sexual relations 2 days before to optimize the quantity and quality of the sample to be taken.

The patient is asked to retract the foreskin and with two sterile swabs a sample of the lesions and/or secretions that can be observed is taken.
If you have a culture order, take a sterile swab.

ÿ With a second swab, make smears and smears for Gram staining. The slides must be previously marked with the reference number, patient's initials, and examination to be performed.

ÿ The swabs are placed in a sterile tube for transport to the laboratory.

• URETHRAL SECRETION SWEATER

ÿ The patient must attend the Laboratory with urinary retention of at least 2 hours and

Machine Translated by Google

sexual abstinence for 2 days.

ÿ The patient is asked to retract the foreskin and press so that the secretion flows freely; If there is no discharge, insert a sterile swab into the urethral canal and take the sample. Deposit in sterile tube for transport to the laboratory.

ÿ With a second swab, make a smear and smear for Gram staining. The slides must be previously marked with the reference number, patient's initials and examination to be performed.

• **PROSTATIC FLUID**

The sample is taken by the Urologist doctor who orders the examination.

ÿ For the sample, a prostate massage must be performed; the patient must have eliminated urine at least 2 hours before the exam and must have previously washed it with soap and water.

ÿ Description of the procedure to perform prostate massage: The patient is placed in a crawling position on the table, it is performed by introducing one or two fingers, sheathed in a latex glove and lubricated with mineral oil, through the anus, locating the prostate that is a tissue with a smooth and soft texture and the massage begins. Excessive pressure is not necessary. The procedure has to be carried out by rubbing gently at first with the index finger, massaging the sides of the prostate lobes, taking
Be careful not to press too vigorously on the central nerves.

ÿ A clear and transparent liquid should be obtained, in cases of prostatitis it is cloudy. The fluid must be free of seminal secretion to be a valid sample. Place in sterile tube to be transported to the laboratory.

• **PHARYNGAL SECRETION:**

ÿ It is used for the diagnosis of streptococcal pharyngitis and other types of pathogens.

ÿ Ask the patient if they are taking any type of antibiotic, if they have eaten any food or if they have rinsed their mouth.

ÿ Ask the patient to open his mouth and with the help of the tongue depressor keep the tongue downwards, with a sterile swab rub the tonsillar crypts and/or the posterior pharynx the sites with secretion, membranes or inflammation. If possible, do not touch the oral mucosa, tongue, uvula or teeth.

ÿ Place in sterile tube to be transported to the laboratory.

Machine Translated by Google

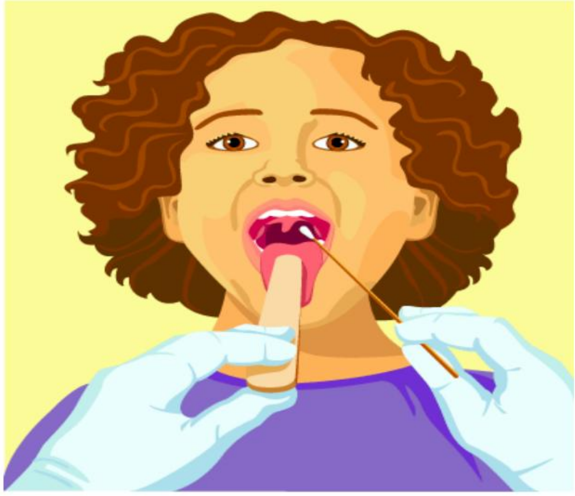

• **PHARYNGEAL SWAB FOR STREPTO A TEST.**

ÿ The sample is taken from a pharyngeal swab in patients with symptoms of fever, throat irritation and repeated pharyngitis.
ÿ The assistant must take the sample with a swab (provided by the commercial company) from the most irritated or exudative areas that are observed, avoiding contamination with oral flora, helped by a tongue depressor if necessary.
ÿ Put the swab in a sterile tube with the reagents provided by the commercial company (buffer A and buffer B in equal quantities) correctly marked with the patient's reference number and capped.
ÿ Deliver to the bacteriologist of the Immunology Unit.

13.5 SECRETIONS AND WOUNDS:

For the different culture samples to be taken, verify if you are taking antibiotics, the relevant information should be recorded in the comments in Datalab Enterprise

• **EYE SECRETION:**

ÿ Take the sample with a sterile swab from the inner part of the eyelids or from the secretion found on the inner edge of the eyelids, do not touch the adjacent skin to avoid contamination of the sample.
ÿ Collect the sample with a sterile swab and transport it to the laboratory in a glass tube. sterile.

• **EAR SECRETION:**

ÿ Take the sample with a sterile swab of the secretion present in the ear, avoiding contamination with the area surrounding the injury.
ÿ If the secretion is in the middle or inner ear, it is the ENT doctor who must take the sample, the material must be prepared for collection by the doctor.
ÿ Collect the sample with a sterile swab and transport it to the laboratory in a glass tube.

Machine Translated by Google

sterile.

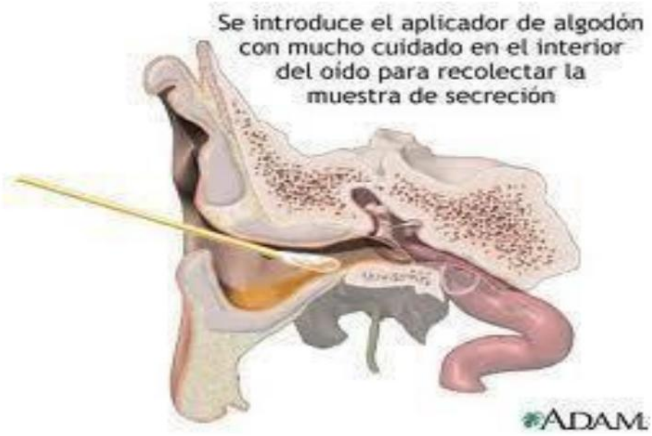

• **NASAL SECRETION:**

ÿ The patient should not apply nasal drops or antibiotics 24-48 hours before taking of the sample, nor should nasal baths be performed.

ÿ Take the sample with a sterile swab from the nasal passages, tilting the patient's head slightly back, insert the swab to the top of the nose, rotate it very gently, leaving it there for a few seconds and remove it.

ÿ Collect the sample with a sterile swab and transport it to the laboratory in a glass tube. sterile.

• **Eosinophils in Nasal Mucus:**

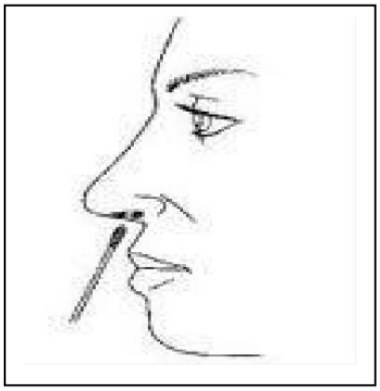

Ask the patient to try to gently expel nasal discharge if any. Take the sample with a sterile swab from the nasal passages, tilting the patient's head slightly back, insert the swab to the top of the nose, rotate it very gently, leaving it there for a few seconds and remove it. Take two plates for Wright's staining. Divide the sheet in half, mark the sheet indicating the collection site: Right fossa Left fossa

Machine Translated by Google

• **WOUND SECRETION:**

ÿ Clean the edge of the lesion with sterile saline solution
ÿ Take the sample with a sterile swab from the lesions observed. If the surface is healed, the surface should be lifted and pressed gently in order to obtain purulent material if there is any.

ÿ For abscesses, wounds, ulcers or furunculosis, the doctor must take the sample with a sterile syringe and needle.
ÿ Transport to the laboratory in a sterile container.

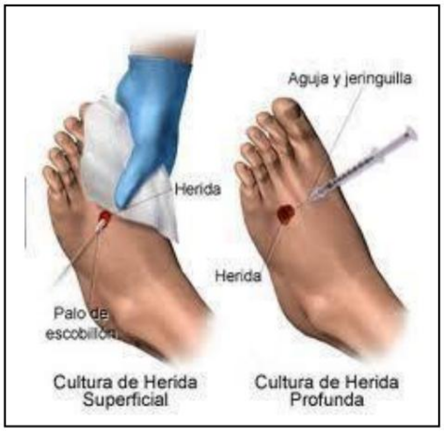

• **Whooping cough**

The international recommendation for the diagnosis of whooping cough *(Bordetella pertussis or parapertussis)* are three techniques:

- Positive direct immunofluorescence (DIF)
- Culture for *B. pertussis or B. parapertussis* on REGEN LOWE medium
- Positive PCR test for *B. pertussis or B. parapertussis*

It is important to guarantee the sample of the nasopharyngeal swab or the nasopharyngeal aspirate, depending on the case, even when the child or person has started antibiotic treatment, but the recommendation is to take the sample before starting treatment, as this would contribute to a better diagnosis. by the laboratory.

ÿ The sample must be taken by the Respiratory Therapist.
ÿ Samples for contact or case studies in field work must be taken by nasopharyngeal swab.
ÿ Sample collection in hospitalized patients should be done by aspiration nasopharyngeal with sterile tube.
ÿ Introduce the probe with the nasopharyngeal aspirate into a sterile tube and/or bottle and transport to the lab.
ÿ Send to the Secretary of Health of Cundinamarca for study and notification along with the epidemiological file and in accordance with the conditions required by this laboratory.

Machine Translated by Google

13.6 NASOPHARYNGEAL SWAB

**FICHA TÉCNICA PARA TOMA DE MUESTRAS LABORATORIO CLINICO COMPENSAR**		compensar | salud
**TIPO DE MUESTRA:** Hisopado Nasofaríngeo		**ORIGEN:** Nasofaringe
**POBLACION:** General		**ROL:** Auxiliar de Laboratorio
**TÉCNICA**	**ANTES DE REALIZAR LA TOMA**	
	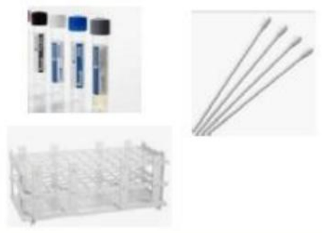	Aplicar instrucciones descritas en la ficha Generalidades de toma de muestras de microbiología.
		Realizar lavado de manos con agua y jabón cada hora, cuando las manos estén sucias, contaminadas con secreciones, cuando se pase de un área contaminada a un área limpia o cuando sea necesario, se debe realizar higienización de manos cuando se requiera según instrucciones establecidas en los 5 momentos de lavado de manos.
		Indicar al paciente la posición que debe adoptar para la adecuada toma de muestra.
	**TÉCNICA DE TOMA DE MUESTRA**	
	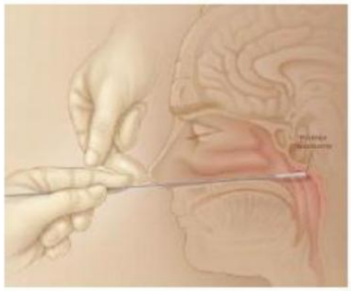	Con la mano libre, llevar hacia atrás la cabeza del paciente y con la otra mano, introducir el hisopo humedecido a través de los orificios nasales, paralelo al paladar (no hacía arriba), hasta que se encuentra resistencia o la distancia equivalente desde la fosa nasal hasta la oreja. En este punto se encuentra la punta en la nasofaringe.
		Rotar suavemente el hisopo por 5 segundos y luego retirar lentamente, permitiendo que se absorban las secreciones en el
		Retirar el hisopo de la fosa nasal y colocarlo inmediatamente en el tubo con el medio de cultivo.
		Repetir el procedimiento en la fosa nasal contra lateral.

SAMPLE TAKING KIT FOR COVID-19 (NASOPHARINGEOAL SWAB)

The samples requested for covid 19 by external consultation will be taken by the laboratory assistant staff in module 5 of external consultation sample collection. In inpatient services, samples will be taken by respiratory therapy staff.

For the procedure, the laboratory assistant must have:

11.1.1 Transport refrigerator with cooling cell.
11.1.2 1 Flexible Nylon, Rayon or Dacron brushes
11.1.3 MTV Viral Transport Medium
11.1.4 Urine bottle (secondary packaging). absorbent towel
11.1.5 A ziploc bag

Machine Translated by Google

PERSONAL PROTECTION ITEMS

In accordance with the provisions of the biosafety manual (LC.01.MA.18), the personal protection elements to be used are:

11.1.6 Disposable gown
11.1.7 Disposable cap
11.1.8 Gloves
11.1.9 N95 mask
11.1.10 Mask

Consult the biosafety protocol in the biosafety manual (LC.01.MA.18). See annex SAMPLING FOR PCR-COVID 19 IN OUTPATIENT.

13.7 TAKING BLOOD CULTURES IN HOSPITALITY

Aim

Provide general recommendations based on evidence and avoid false positives in blood culture collection that may compromise patient results and treatment.

Introduction

Blood cultures are essential for the diagnosis of bacteremia. The invasion of microorganisms in the blood is one of the most significant causes of increased morbidity and mortality in patients, representing one of the most prevalent causes of infection. The problem is of considerable magnitude, nearly 200,000 patients develop bacteremia or fungemia annually in the United States, with an attributable mortality of 20-50%. It has been estimated that a contaminated blood culture causes an increase of 4 to 5 days in hospitalization time and an added treatment cost of about €4000. Many of these episodes are nosocomial and in some institutions they represent the majority of cases; Likewise, increased bacterial resistance is associated with great morbidity from community-acquired episodes. Bacteremia is defined as the presence of bacteria in the blood, which is revealed by their isolation in blood cultures. The term fungemia is used to designate the presence of fungi in the blood. Septicemia and sepsis are expressions used to describe the clinical syndrome with which bacteremia or fungemia usually manifest, regardless of the results of blood cultures. Bacteremia and fungemia are serious complications of infections

bacterial and fungal, respectively, and have a very similar diagnostic methodology, so will describe together. Both occur when microorganisms invade the bloodstream and multiply at a rate that exceeds the ability of the reticuloendothelial system to eliminate them. This Invasion can occur from an extravascular infectious focus, through blood capillaries or lymphatic vessels, or from an intravascular focus (endocarditis, infection of intravenous or arterial catheters, etc.). The incidence of bacteremia depends on the type of population studied (5-30 cases per 1000 hospitalized patients) and can occur at any age, especially in patients with serious underlying diseases and in those undergoing maneuvers that alter local and general defense mechanisms against infection.

The most frequent sources of bacteremia identified are the genitourinary tract, abscesses, surgical wounds, biliary tract, and intravascular catheters; however, in up to 25% of cases, the original source is unknown. Most microorganisms are capable of invading the bloodstream.
Currently, gram-positive bacteria, especially staphylococci and enterococci, equal or exceed the frequency of gram-negative bacteria. This is due to multiple causes, among which the use of

Machine Translated by Google

broad-spectrum antibiotics, the widespread use of intravascular catheters and the use of invasive diagnostic methods. On the other hand, the increase in immunosuppressed patients with antineoplastic treatments or with HIV infection has led to the appearance of bacteremia due to agents that in the past were very rare causes of infection. The definitive diagnosis of bacteremia and fungemia is established when the causative microorganism is isolated from the patient's blood by blood culture. Isolation of the responsible agent is essential to determine its sensitivity to antimicrobials and establish the necessary treatment or modifications to the already established empiric therapy. Sometimes it can guide the diagnosis of diseases such as colon neoplasia (associated with bacteremia due to Streptococcus bovis), endocarditis (viridans group streptococci) and even HIV (Salmonella and enterococcus). On the other hand, it allows, in most cases, the differentiation of cases of true bacteremia from those in which the positivity is due to an inadequate extraction and processing procedure.

Definition

Blood culture is a diagnostic method that is performed to detect microorganisms in the blood and, subsequently, carry out the identification and determination of sensitivity.

Care and recommendations

- Confirm medical order in the system (Hosvital)
- Perform surgical hand washing
- Maintain aseptic technique throughout the procedure
- Use a sterile field to avoid contact with other areas to reduce the risk of contagion.
- Perform antisepsis on the area and do not palpate the vein without sterile gloves once the skin has been prepared.
- Change gloves after each antisepsis and puncture
- Ideally collect 8 to 10 ml per venipuncture for adults
- Ideally collect 0.5 to 4 ml per venipuncture for pediatrics
- Always inoculate the anaerobic bottle first and then the aerobic bottle.
- For cultures collected by peripheral veins, the following veins are recommended: middle ulnar, cephalic in upper limbs.
- Collecting blood cultures through veins in the lower limbs and arterial blood increases the risks of adverse contamination events, thus reducing the possibility of microbiological recovery.

- Blood collection through already channeled peripheral lines **IS NOT RECOMMENDED.**
- Taking blood through a central catheter is only indicated for infections associated with the central catheter itself, but it must be taken after taking blood through a peripheral vein.
- **IT IS NOT RECOMMENDED** to change the needle at the time of inoculating the blood into the vials since This generates an increase in accidents due to biological risks.
- Blood culture sampling from the arterial line **is NOT RECOMMENDED**
- Returning blood from the initial sample to the patient **is NOT RECOMMENDED .**

Equipment

- Chlorhexidine soap and solution
- Sterile fields
- Two pairs of gloves for each blood culture collection
- Sterile gauze
- Tourniquet

Machine Translated by Google

• Cap •
Surgical mask or face mask • Surgical
gown • Blood culture
bottles • Three sterile syringes

Technique for collecting blood cultures through peripheral accesses

1. Receive complete laboratory material 2.
Receive complete medical-surgical material from the pharmacy
3. Explain the procedure to the patient and family 4.
Nurse and assistant must put on a hat and mask 5. The patient's
mask position is suggested during the measurement of blood cultures 6. Select vein to puncture:
easy access, good caliber 7. Wash surgical hands according to
institutional protocol 8. Put on surgical gown 9. Put on sterile
gloves 10. Place sterile fields
around the puncture site 11.
Apply chlorhexidine soap in a sterile gauze and with grid
movements perform mechanical friction
from the puncture site
12. Perform the same procedure with chlorhexidine solution 13.
Let the skin dry spontaneously for 2 minutes 14. Change
gloves with aseptic technique 15. Place a tourniquet
(Accompanying assistant)
16. Receive sterile syringe and perform puncture in selected vein at a 35° angle 17. Obtain
8 to 10 ml of blood from each vial for adult patients 18. Obtain each sample
from three different anatomical sites with an interval of 15 minutes. Can
from the same anatomical site if it is an aerobic and an anaerobic sample.
19. Remove the tourniquet, extract the needle and apply pressure with gauze on the puncture
site 20. Remove the plastic cap from the bottle, reach for the sterile needle and open the alcohol swab (Auxiliary
companion) proceed to continue applying pressure.
21. Receive isopanin and disinfect the rubber stopper of the bottle 22.
Inject the sample through the rubber stopper of the bottle with aseptic technique 23. Discard
the needle in the sharps container.
24. Mark the bottles with the sticker that includes the full names and surnames, document number, write the order and time of taking the blood cultures along with the puncture site, this marking should NOT cover the barcode of the jars.
25. Wash your hands after finishing the procedure

Central catheter blood culture collection technique

1. Receive complete laboratory material 2.
Receive complete medical-surgical material from the pharmacy
3. Explain the procedure to the patient and family 4.
Nurse and assistant must put on a cap and mask 5. Wash
surgical hands according to institutional protocol 6. Put on
surgical gown 7. Put on sterile
gloves

Machine Translated by Google

8. Close the intravenous infusion port, for 3 – 5 minutes (depending on the condition of the patient).
9. Choose a nearby port, clean for 15 seconds using a 2% Chlorhexidine Gluconate solution in association with 70% isopropyl alcohol and allow it to dry.
10. In adults, extract 20 mL of blood from the line and divide the contents of the syringe as follows: 10 mL for the anaerobic bottle and 10 mL for the aerobic bottle without changing the needle. Always inoculate the anaerobic bottle first and then the aerobic bottle.
11. Remove the plastic cap from the bottle, reach for the sterile needle and open the alcohol isopanin (Auxiliary companion).
12. Receive isopañin and disinfect the rubber stopper of the bottle
13. Inject the sample through the rubber stopper of the bottle with aseptic technique
14. Discard the needle in the sharps container.
15. Mark the bottles with the barcode and write the order and time of taking the blood cultures
16. Wash your hands after finishing the procedure

blood volume

1. Aerobic and anaerobic adult bottles: 10 mL
2. Pediatric bottles: 0.5 to 4 mL. This volume will be according to the patient's weight:

Cuadro 1. Volúmenes de sangre a extraer por juego de hemocultivo según edad y peso en población pediátrica.

Población	Edad	Sitio	Volumen Minimo	Botellas
Neonatos	0-28 dias (o pacientes en URN)	vena periferica	<8 kg: 1 mL	Una botella pediatrica aerobica
Niños	1-3 meses	vena periferica	<8 kg: 1 mL	Una botella pediatrica aerobica
	3-36 meses	vena periferica	<8 kg: 1 mL 8-13 kg: 3 mL 13-27 kg: 5 mL	Botella pediatrica aerobica si el volumen es menor de 0,5 - 4 mL Botella aerobica de adulto si el volumen es mayor de 4,0 mL
	4-11 años	vena periferica	8-13 kg: 3 mL 13-27 kg: 5 mL 27-40 kg: 10 mL >40 kg: 10 mL	Botella pediatrica aerobica si el volumen es menor de 0,5 - 4 mL Botella aerobica de adulto si el volumen es mayor de 4,0 mL
	12-17 años	vena periferica; considerar dos venas de sitios separados para 2 cultivos	27-40 kg: 10 mL >40 kg: 10 mL	Botella pediatrica aerobica si el volumen es menor de 0,5 - 4 mL Botella aerobica de adulto si el volumen es mayor de 4,0 mL

Source: Sampling Manual for microbiological analysis LSP Bogotá

Bottle type

Green cap: Aerobic adult
Yellow cap: Pediatric aerobic
Orange cap: Anaerobic

Machine Translated by Google

Sample Identification

Each bottle must be identified with the following data:

- Full names and surnames of the patient
- ID number
- Sample type and anatomical site
- Number of blood cultures collected
- Collection date and time

Transportation

- It is recommended that it be transported within the first 15 minutes of collection at temperatures atmosphere.

Blood culture quality indicators

1. Pollution:

% contamination of blood cultures. Formula:

No. of contaminated blood cultures in the month (numerator) No. of bottles processed in the month x 100 (denominator)

2. Positivity:

% of positive blood cultures Formula:

No. of positive blood cultures in the month (numerator)
No. of bottles processed in the month x 100 (denominator)

3. Volume

% of bottles with adequate volume

Machine Translated by Google

Formula:

No. of bottles with adequate volume (numerator
No. of bottles taken in the month x 100 (denominator)

13.8 Blood cultures in outpatient clinic:

The patient must be feverish to take the sample; it can be taken at any time of the day or night, after having previously completed the billing process.

- Clean the counter very well with Surfanios. Use the lighter and take the sample near this to create a sterile field, likewise use a mask and hat.
- With a sterile glove, prepare the skin for venipuncture, disinfect from the elbow to the doll with antiseptic alcohol.
- Take the blood sample with normal venipuncture.
- Blood culture samples should be taken at 3 different sites, one bottle for each site; (for example: right arm center, left arm center and right side).
- Take 3-5 ml of blood in adults and 1-3 ml of blood in children, in the bottles. indicated according to the medical order.
- For adults you should take 3 bottles and 2 bottles for children.
- In children, the ideal is to take from two different anatomical sites, but taking into account that in pediatric patients venous access is often difficult, it could be taken from the same anatomical site.
- The blood culture bottle can be used as a normal tube since it has a vacuum and we fill the bottle with what you need
- It should be taken every 20 minutes, the patient should not take any antipyretic until not finish the outline.
- Mark the bottles with the barcode and write the order and time of taking the bottles. blood cultures.
- Write relevant clinical data, medication, time of onset of symptoms.
- Deliver to the bacteriologist responsible for Microbiology for incubation.

14. TAKING OF MYCOLOGICAL SAMPLES

To take a fungal sample with a request for KOH and/or mycosis culture from any anatomical site, verify that the patient is not taking antifungals. If they have received treatment, they must wait at least fifteen (15) days to take the fungal sample. No type of cream and/or ointment has been applied to the lesions. If the sample is fingernails, it should not have polish or cream; If it is from the toenails, you should not apply talcum powder or creams. The Laboratory assistant must verify the site of the injury to properly take the requested sample.

If you have any questions, you should confirm with the microbiology bacteriologist to guide the collection of the sample.

Machine Translated by Google

14.1 SCALP:

ÿ Choose the portion of the hair that interests us for the exam.
ÿ In case of a stone, examine the hair in the place where the stone is, cut with scissors and in case of ringworm, choose the portion close to the root, cutting the hair or pulling so that it comes out with the root. The sample should be 5 to 10 hairs. Place the sample in a sterile laboratory bottle.

ÿ Scrape the part of the lesion with a scalpel and leave the scalpel in the bottle.
ÿ Mark the samples with the barcode label, specifying whether it is only direct and/or with culture and the exact location of the sample.
ÿ Additionally, use the adhesive tape technique: It consists of placing a strip of adhesive tape 1.5 to 2 cm wide on the lesion, placing it once (1) on the skin, removing and placing it on a slide plate at the same time. to which 1 drop of 20% KOH has been added and stick the tape to the sheet; make 2 sheets;

identified with reference number and initials of the patient's first and last name and sample collection site.

.

14.2 SCALES - SKIN:

ÿ In the case of pityriasis versicolor, use the adhesive tape technique: It consists of placing a strip of adhesive tape 1.5 to 2 cm wide on the lesion, placing it once (1) on the skin, removing and placing it on a slide sheet to which 1 drop of 20% KOH has been added and stick the tape to the sheet; make 2 sheets; identified with reference number and initials of the patient's first and last name and sample collection site. sample.

ÿ Collect sample using a scalpel: scrape the edge of several lesions to obtain a greater amount of material, preferably selecting the areas in which raised, erythematous and peeling edges are observed, or on the periphery of the lesions and in those cases where that present blisters, the roof will be sectioned.
ÿ Place the sample in a sterile fungal bottle, place the scalpel inside the bottle, with the respective barcode identification and exact location of the sample.

14.3 NAILS

ÿ Take the sample from the nail lesion using the screw technique by rotating the tip of the scalpel over the nail lesion (use the technique only if the patient approves of taking it after explaining what it consists of).
ÿ Scrape under the nail with the tip of the scalpel from the proximal to the distal end and allowing the sample to fall into the sterile fungal bottle, place the scalpel inside the bottle

ÿ If the sample is from the toes (Artejos), they must be numbered.
And follow the previous procedure.
ÿ When taking mushrooms from throat and vaginal secretions, no other examination, if not included in the Gram stain.

14.4 PUS, EXUDATES, SECRETIONS, WOUNDS:

For mushroom samples of this sample type:

Machine Translated by Google

ÿ Take the samples with a sterile swab, place it in a sterile tube to transport to the laboratory. Label with the respective barcode identification and exact location of the sample.

14.5 URINE FOR KOH:

The patient must collect the first urine in the morning after genital cleansing. If the patient is hospitalized, the sample should preferably be taken with a sterile catheter. Process the sample immediately; The value of mycological studies with this sample is limited. However, in hospitalized patients, the doctor must take into account the clinical history and clinical status of the patient to determine whether Candida is pathogenic or not.

15. SAMPLES FOR DIAGNOSIS OF TUBERCULOSIS

15.1 SPUTUM

ÿ Clinical laboratory has a KIT that consists of three special wide-mouth, screw-top bottles for collecting these samples. The kit includes instructions to give the patient instructions for collecting the sample.

ÿ For outpatient patients, the ideal is to collect a sample every day. However, The delivery of the three samples must be ensured, making it easier for the patient.

ÿ For emergency patients, samples must be collected every hour, thus guaranteeing the completeness of the scheme.

ÿ Hospitalized patients can collect samples one each day.

15.2 URINE FOR TB CULTURE:

The patient must collect the total amount of first urination of the morning in a wide-mouth, plastic container (to facilitate incineration), with an airtight seal to prevent spillage and protected from direct light. Three serial samples should be taken on different days, preferably collected when the patient wakes up. The samples are
They must always be collected following the same conditions. The sample must be protected from light, recommend that the patient place the sample in a dark bag properly marked with the name.

15.3 GASTRIC ASPIRATE:

Gastric aspirate culture is essential for the diagnosis of tuberculosis or mycobacteriosis in adult patients and children who do not expectorate and swallow their sputum. Taking a sample requires work by the intrahospital team. It is essential to carry it out in the best conditions. The sample must be collected in an appropriate container: sterile screw-cap tubes to which 2 ml of 10% sodium triphosphate is added for every 10 ml of gastric aspirate. This addition is made to the sterile tubes upon request of the service where the procedure is to be performed. It is required to take serial samples for three consecutive days in order to increase the diagnostic possibility. The sample collection procedure is carried out by a head nurse of the requesting service as follows: The nasogastric tube is passed the night before; is fixed and the fixation point is marked. Before waking the patient, the gastric contents are aspirated with a syringe. The aspirate is placed in the sterile tube to allow the neutralization of the acidic pH of the gastric contents, as it affects the viability of the mycobacteria. 50 ml of sterile distilled water are injected and aspirated again; The aspirated is placed in the same sterile tube, the

Machine Translated by Google

Minimum amount recovered should be 10 to 20 ml. The gastric aspirate sample intended for culture should be sent to the laboratory protected from direct light and avoiding spillage. See Microbiology manual.

16. SAMPLES FOR DIAGNOSIS OF LEPROSY

HANSEN'S BACILLUS:

ÿ The sample is taken by the assistant

ÿ A total of 6 samples must be taken (new degreased sheets), six samples that are: (1) lymph from ear lobes, (1) lymph from the knee, (2) lymph from elbows and (2) lesions. The case that does not have lesions is replaced by lymph from the elbows or lymph from the ears. The collection of mucus samples is eliminated because false positives have occurred. They will be taken like this:

ÿ One (1) sample of earlobes (1) knee:

ÿ The site is cleaned with alcohol; To take a good sample, it is important to leave the site where the sample is to be taken free of blood, use Kelly-type clawless forceps or clamp.

ÿ With a lancet, 3 or 4 punctures are made close to each other in order to obtain a good sample. Take the sample when it is observed that the sample collection site is completely pale in order to guarantee the absence of blood.

ÿ Two (2) sample: elbows

ÿ One (2) sample of active lesions, if there are no lesions it is replaced by lymph from elbows or ears.

ÿ They must always describe on an additional sheet the exact site where all samples were taken, with a history of the disease and the answer to the following questions:

- Housing conditions (if the floor is made of cement or earth, if the walls are made of cement)
- If you frequently travel to endemic areas.
- Family history of leprosy.
- Loss of sensitivity. Describe if the patient does not feel pain when taking the Sample.
- Loss of eyebrows and/or eyelashes.
- Thickening of the skin of the nose and ear.
- If it is a control, how long have you been on treatment and if you are taking it as indicated.

The origin of each sample taken and the right or left side of the sheet must be identified on the sheet.

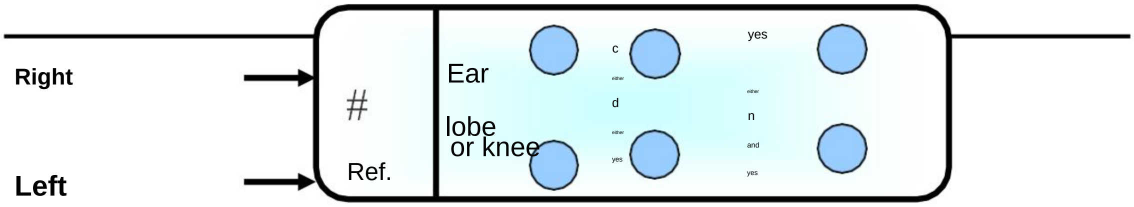

ÿ Take the samples to the Microbiology unit so that the bacteriologist can immediately process the slides. If it is not possible to carry out the procedure, preserve

Machine Translated by Google

samples at room temperature for a period of no more than 4 hours.

17. COLLECTION OF SPECIFIC AND FREE PROSTATE ANTIGEN

The patient should take into account the following:

- If you have had a prostate biopsy or prostatectomy, you must wait at least two (2) weeks to perform the exam.
- If you have had prostate massage, you should wait at least 4 or 5 days. If you have performed rectal examination there is no time restriction.
- Have sexual abstinence for 2 days.
- Not having ridden a bicycle or horse two days before.

18. PROLACTIN

- If the doctor requests it, prolactin pooling is performed, the patient must have at least one hour to remain in the laboratory. Sample collection: Basal, 20 and 40 minutes.
- You must report to the laboratory two (2) hours after waking up.
- Sexual abstinence for at least 1 day.
- Not having ingested dairy products the day before the exam.

19. MEDICATION DOSAGE AND HORMONAL LEVELS

- Do not take medications before taking the sample except by medical prescription.
- Inform the laboratory of medications and doses you are taking, as well as the time of administration. last dose.

20. SAMPLING WITH STIMULUS

Exams that require chemical or nervous stimulation are not performed at this location. The patient will be provided with information about the location or Laboratory to which they must attend for the tests and the Form for Submission of Samples and/or Patients to the Registered Laboratory will be delivered, so that they can present it at the time of attending. to the Reference Laboratory upon request for an appointment at the Reference Laboratory, the information related to the reference laboratory will be explained clearly for any questions that the patient may have, who will record the understanding by signing the copy of the referral form as support. at this headquarters.

Machine Translated by Google

21. SAMPLING FOR LEISHMANIA

FICHA TÉCNICA PARA TOMA DE MUESTRAS LABORATORIO CLINICO COMPENSAR

compensar | salud

**TIPO DE MUESTRA:** Frotis de lesión	**ORIGEN:** Lesión especificada en orden médica
**POBLACION:** General	**ROL:** Auxiliar de Laboratorio o Bacteriólogo

ASPECTOS GENERALES

TÉCNICA

ANTES DE REALIZAR LA TOMA

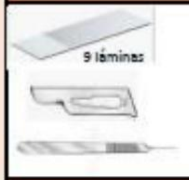

- Aplicar instrucciones descritas en la ficha Generalidades de toma de muestras de microbiología.
- Realizar lavado de manos con agua y jabón cada hora, cuando las manos estén sucias, contaminadas con secreciones, cuando se pase de un área contaminada a un área limpia o cuando sea necesario, se debe realizar higienización de manos cuando se requiera según instrucciones establecidas en los 5 momentos de lavado de manos.
- Indicar al paciente la posición que debe adoptar para la adecuada toma de muestra.

SELECCIÓN DEL SITIO DE TOMA DE MUESTRA

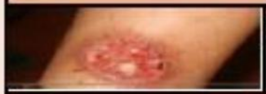

- Evaluar lesiones: tener presente si existen dos o más lesiones debe escogerse para el examen directo la que tenga un menor tiempo de evolución.

PREPARACIÓN DEL SITIO DE LA TOMA DE MUESTRA

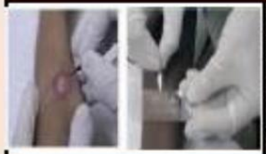

- Realizar una limpieza del sitio de la lesión, utilizando gasa impregnada ya sea de alcohol, solución salina y/o jabón quirúrgico. Si hay costra se debe remover cuidadosamente, colocando compresas de solución salina estéril por lo menos tres momentos antes de la toma de la muestra para ablandar la costra, dejar caer sobre la lesión solución salina a presión, al tiempo ir retirando la costra de arriba hacia abajo
- Elegir la lesión más reciente.

TÉCNICA DE TOMA DE MUESTRA

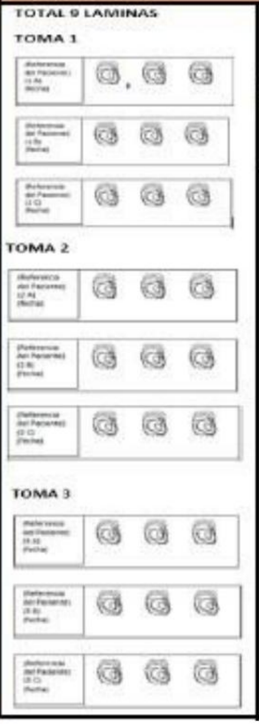

Método	Procedimiento
Frotis (raspado) del borde interno de la úlcera:	Tomar 3 láminas de la lesión de 3 aposiciones cada una.
	Obtener tejido con hoja de bisturí número 15 del borde activo de la lesión o del centro de la úlcera, realice un raspado del fondo de la úlcera.
	Hágalo de manera tal que no sangre mucho, presionando el sitio de la lesión hasta hacer isquemia.
	El material obtenido se extiende en forma suave sobre una lámina portaobjetos nueva, previamente limpia, desengrasada, y debidamente rotulada
Incisión y raspado del borde activo de la lesión:	Este método se recomienda para lesiones cerradas, no ulceradas.
	Realizar la limpieza del sitio de la lesión como se describió anteriormente.
	Tomar 3 láminas de la lesión de 3 aposiciones cada una.
	Si el paciente manifiesta dificultad en asistir en tres días diferentes para la toma de muestra, dividir la lesión en tres partes y de cada zona tomar 3 láminas con sus 3 aposiciones para un total de 9 láminas en las tomas del mismo día.
	Sobre el borde activo de la lesión, realice una pequeña incisión con una hoja de bisturí de 3 a 6 mm de longitud por 1 a 3 mm de profundidad. La isquemia se debe lograr haciendo presión en pinza con los dedos. Se debe ejercer presión en la lesión para hacer isquemia (torniquete para miembros inferiores y superiores).
	Con gasa estéril, limpie la sangre que emana de la incisión y con la misma gasa presione el borde de la lesión para hacer isquemia, la muestra debe ser linfa y debe estar lo menos contaminada con sangre posible.
	Con el borde romo de la hoja de bisturí levante la piel de la parte superior de la incisión y raspe tejido del interior de la incisión desde la profundidad hacia la superficie.
	El material así obtenido se extiende en forma suave sobre la lámina portaobjetos nueva, previamente limpia, desengrasada, y debidamente rotulada.
	Muestras nodulares y papulares no se toman por personal de laboratorio, es un procedimiento médico

Machine Translated by Google

22. BIBLIOGRAPHY

• Pre-analytical Quality Manual. Andalusian Health Service. 2001

• AEBM, AEFA and LABCAM. The Clinical Laboratory: Pre-analysis of urine samples. 2005 Andalusian Health Service. Carlos Haya Regional University Hospital.

• Clinical laboratory. Venous extraction protocol. Quality Manual. 2009. [accessed on September 1, 2010]. Available at: http://www.carloshaya.net/chchaya/ UGC/laboratories/Procedimientos/01_ Pre-analytical/02_Obtencion_Espeimenes/ PRO08A.pdf.

• BRAZILIAN SOCIETY OF CLINICAL PATHOLOGY/LABORATORIAL MEDICINE, Recommendations of the Brazilian Society of Clinical Pathology/Laboratory Medicine for Venous Blood Collection. 2005. Based on: CLINICAL AND LABORATORY STANDARDS INSTITUTE. NCCLS - H03A5 - Procedures for the collection of diagnostic blood specimens by venipuncture; Approved Standard 5ed.· Influenza-like illness protocol, Ministry of Social Protection, National Institute of Health, 2011

• Adapted: BRAZILIAN SOCIETY OF CLINICAL PATHOLOGY/ MEDICINE LABORATORIAL, Recommendations of the Brazilian Society of Clinical Pathology/Laboratory Medicine for Venous Blood Collection. 2005. Based on: CLINICAL AND LABORATORY STANDARDS INSTITUTE. NCCLS - H03A5 - Procedures for the collection of diagnostic blood specimens by venipuncture; Approved Standard *5ed.*

• Generalitat of Catalunya. Department of Health and Social Security. Requirements that must be met by the modules for obtaining samples, including the preservation and transportation after the laboratory. Barcelona, December 2001.

• National Committee for Clinical Laboratory Standards. *Procedure for handling and processing blood specimens; Approved guide.* NCCLS Document H18-A. Villanova: NCCLS, 1990.

• González-Oller C, Alsina MJ. *Database on stability of biological quantities.* National Committee for Clinical Laboratory Standards. *Urinalysis and collection, transportation and*

• Conservation of urine specimens; Approved guide. NCCLS Document GP16-A Villanova: NCCLS.1995.

• Guide to using BD Vacutainer Products. Taking order recommended by the Clinical and Laboratory Standards Institute. (CLSI)

• For the collection, transportation, conservation and submission of Clinical Laboratory samples. PRO-PSS-0080. Daruma, Compensate.

23. ANNEXES

CODE	QUALIFICATION	RESPONSIBLE
**LC.01.MA.02.FT.01**	Informed consent for taking of samples	Bacteriologist
**LC.01.MA.02.FT.02**	Informed consent for loads of Glucose	Bacteriologist

Machine Translated by Google

**LC.01.MA.02.FT.03**	Responsibility format samples with labeling errors	Bacteriologist
**LC.01.MA.02.FT.04**	Informed dissent for clinical laboratory sample collection	Bacteriologist
**APPENDIX 1**	Sample collection for pcr-covid 19 in an outpatient	Bacteriologist
**APPENDIX 2**	Table of personal protection elements	Bacteriologist
**ANNEX 3**	Clinical data survey rapid test SARS CoV 2 Informed	Bacteriologist
**ANNEX 4**	Consent for processing of Covid- 19 serological tests.	Bacteriologist
**ANNEX 5**	Sample Centrifugation	Bacteriologist

ELABORATED	REVISED	APPROVED
**Name:** Yury Andrea Quintero<br><br>**Position:** Bacteriologist Clinical laboratory<br><br>**Signature:**	**Name:** Luz Helena Baron<br><br>**Position:** Administrator Clinical laboratory<br><br>**Signature:**	**Name:** Hermencia Carolina Aponte Murcia<br><br>**Position:** Deputy Director of Quality, Education and Investigation<br><br>**Signature:**

Machine Translated by Google

APPENDIX 1

SAMPLE COLLECTION FOR COVID 19 IN AN OUTPATIENT

The Compensar Clínica Universidad de la Sabana headquarters adheres to the guidelines referred to in the "Instructions for the Prevention, Containment and Care of COVID 19 INS-PSS-576" and that document defines the following.

For the procedure, the laboratory assistant must have:

Yo. Transport refrigerator with cooling cell.
ii. 2 flexible Nylon, Rayon or Dacron brushes
iii. MTV viral transport medium
iv. Urine bottle (secondary packaging). absorbent towel
v. A ziploc bag.
saw. Personal protection items.
vii. Mask carrying bag.

According to the number of patients scheduled, there must be a sufficient number of elements.

Nasopharyngeal swab sample collection in outpatient clinic:

Sample collection is in charge of a laboratory assistant. Prior to taking the sample, the sample must be marked, recording the correct ones on the marking:

ÿ Full names
ÿ Identification number
ÿ Sample collection time
ÿ Sample type

1. The information must be verified by double checking with the patient and verifying a physical document.

2. Verify the correct completion of the data notification forms basics and 346.
3. Sign informed consent for sample collection. **LC.01.MA.02.FT.01**
4. Complete authorization to send clinical laboratory results to patients **LC.01.MA.01.FT.01.** when sending by email is requested by the patient.

5. In case of taking a sample for COVID-19 antigen, carry out the survey *"CLINICAL DATA SURVEY RAPID TEST SARS CoV 2 (COVID-19) ANTIGEN DETECTION"*

6. Verify medical order versus invoice.
7. After taking the scheduled patients, they will be transferred to the laboratory and admitted to Enterprise. Deliver barcode sticker and transport cooler to microbiology for verification, and referral if applicable.

PCR-COVID 19 SAMPLE TAKING

Nasopharyngeal swab will be taken according to the established technique:

Machine Translated by Google

FICHA TÉCNICA PARA TOMA DE MUESTRAS
LABORATORIO CLINICO COMPENSAR

compensar | salud

**TIPO DE MUESTRA:** Hisopado Nasofaríngeo		**ORIGEN:** Nasofaringe
**POBLACION:** General		**ROL:** Auxiliar de Laboratorio
**TÉCNICA**	**ANTES DE REALIZAR LA TOMA**	
	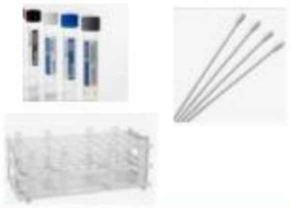	Aplicar instrucciones descritas en la ficha Generalidades de toma de muestras de microbiología.
		Realizar lavado de manos con agua y jabón cada hora, cuando las manos estén sucias, contaminadas con secreciones, cuando se pase de un área contaminada a un área limpia o cuando sea necesario, se debe realizar higienización de manos cuando se requiera según instrucciones establecidas en los 5 momentos de lavado de manos.
		Indicar al paciente la posición que debe adoptar para la adecuada toma de muestra.
	**TÉCNICA DE TOMA DE MUESTRA**	
	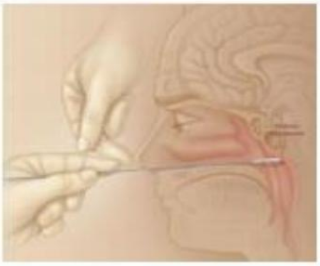	Con la mano libre, llevar hacia atrás la cabeza del paciente y con la otra mano, introducir el hisopo humedecido a través de los orificios nasales, paralelo al paladar (no hacía arriba), hasta que se encuentra resistencia o la distancia equivalente desde la fosa nasal hasta la oreja. En este punto se encuentra la punta en la nasofaringe.
		Rotar suavemente el hisopo por 5 segundos y luego retirar lentamente, permitiendo que se absorban las secreciones en el
		Retirar el hisopo de la fosa nasal y colocarlo inmediatamente en el tubo con el medio de cultivo.
		Repetir el procedimiento en la fosa nasal contra lateral.

NASOPHARYNGEAL SWAB SAMPLE COLLECTION FOR COVID 19 ANTIGEN

For the COVID 19 antigen test, the sample collection must be carried out in accordance with what is specified in the test insert:

Machine Translated by Google

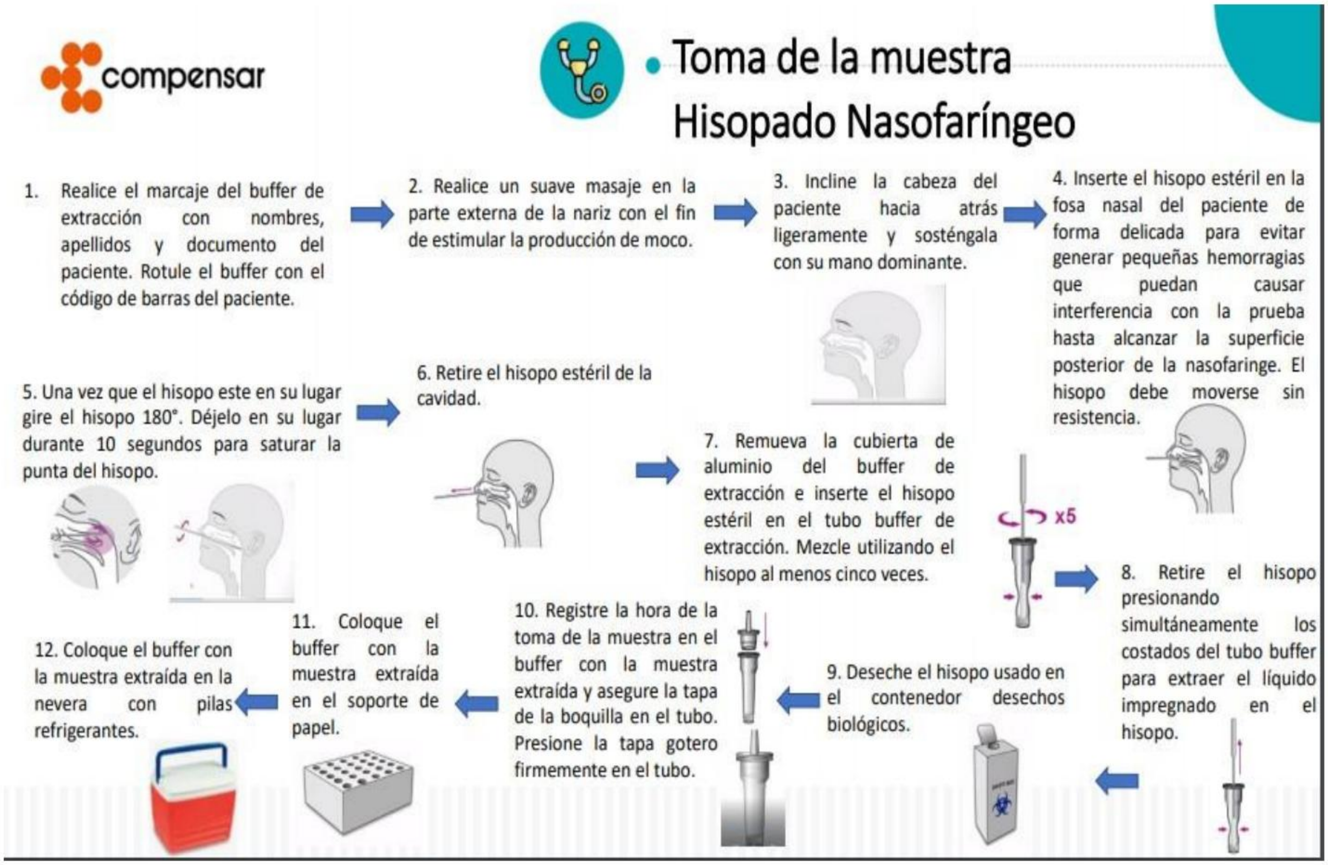

BIOSECURITY

For the procedure, the assistant will use the established personal protection elements: (See table of personal protection elements. Annex 4)

SUBPROCESO	PERFIL	ESPECIFICIDAD DE LA ACTIVIDAD											
LABORATORIO	BACTERIOLOGÍA	Procesamiento de muestras COVID19 fase de alistamiento y extracción	👍	👍	👎	👍	👍	👎	👍	👍	👎	👍	👎
		Procesamiento de muestras de rutina (hematología, bioquímica, estre otros)	👍	👍	👍	👎	👍	👎	👍	👍	👎	👎	👎
		Procesamiento de muestras para otros virus respiratorios	👍	👍	👎	👍	👍	👎	👍	👍	👎	👎	👎
		Procesamiento de baciloscopias montaje laminas	👍	👍	👎	👍	👍	👎	👍	👍	👎	👎	👎
		Procesamiento de muestras para micobacterias fase de siembra	👍	👍	👎	👍	👎	👎	👍	👍	👎	👍	👎
	AUXILIAR LABORATORIO	Toma de muestras sanguíneas en sede	👍	👍	👍	👎	👍	👎	👍	👍	👎	👎	👎
		Toma de muestras sanguíneas en domicilio	👍	👎	👍	👎	👍	👎	👍	👍	👎	👎	👎
		Toma de muestras sanguíneas a pacientes COVID19 (+)	👍	👍	👍	👎	👍	👎	👍	👍	👎	👎	👎
		Toma de hisopado nasofaríngeo	👍	👍	👎	👍	👍	👎	👍	👍	👎	👎	👎

Machine Translated by Google

- Wash hands with soap and water between each patient. Changing gloves between patient.
- The cap, gown and conventional mask are discarded at the end of the patient intake. scheduled.
- Spray the mask with 70% alcohol. Place it in a plastic bag to be transported to the laboratory where it is washed with detergents for disinfection.
- Dispose of PPE in a red bag.
- Terminal cleaning must be carried out at the end of taking the scheduled patients. Notify cleaning staff at the end.
- The patient must enter with a conventional mask and perform hand hygiene with antibacterial gel. During sample collection, only the nose should be uncovered.

Orden para colocación y retiro de los elementos de protección personal:

COLOCACIÓN	RETIRO
1. Lavado de las manos.<br>2. Bata clínica desechable.<br>3. Gorro<br>4. Guantes<br>5. Tapabocas quirúrgico o N95.<br>6. Gafas de protección o careta.	1. Guantes<br>2. Bata o delantal.<br>3. Gafas o escudo facial.<br>4. Lavado de manos<br>5. Tapabocas<br>6. Gorro

Machine Translated by Google

HANDWASHING

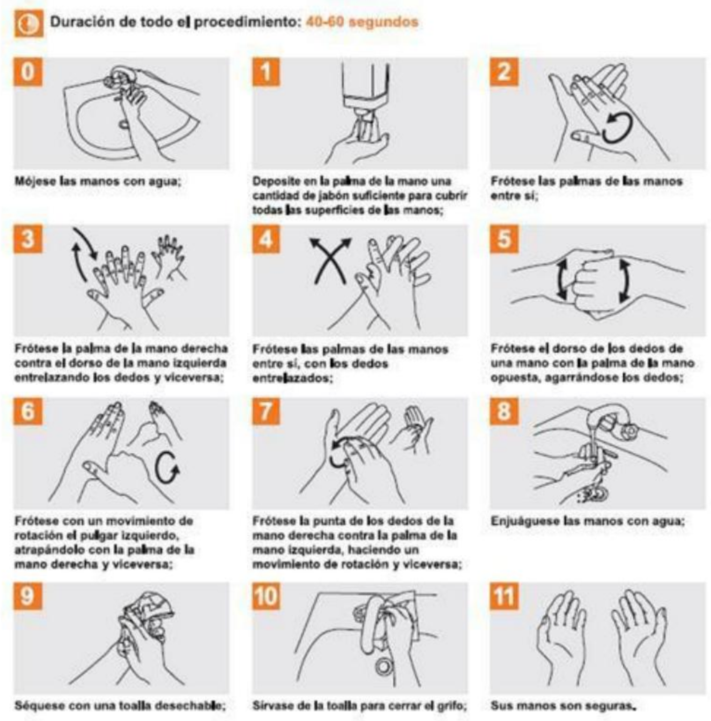

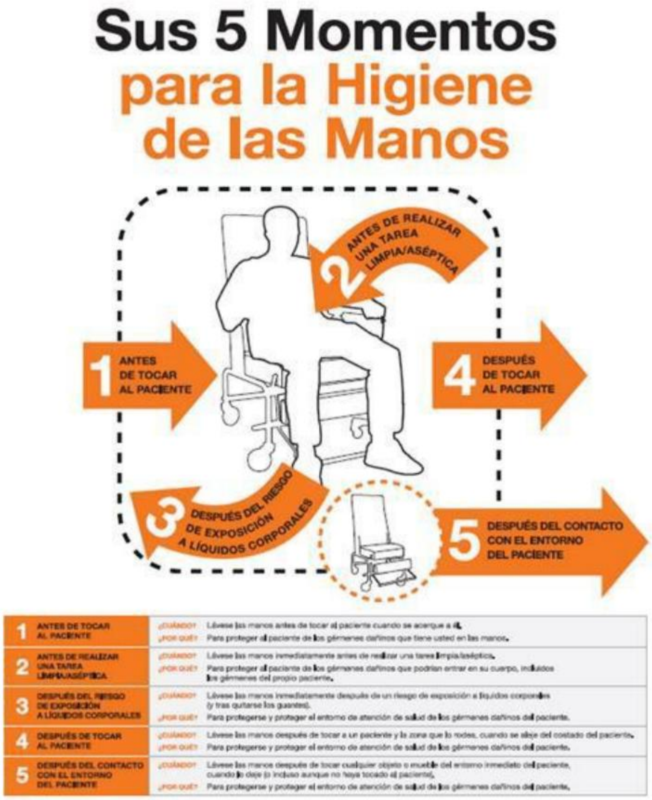

Machine Translated by Google

PROPER USE OF CONVENTIONAL MASKS

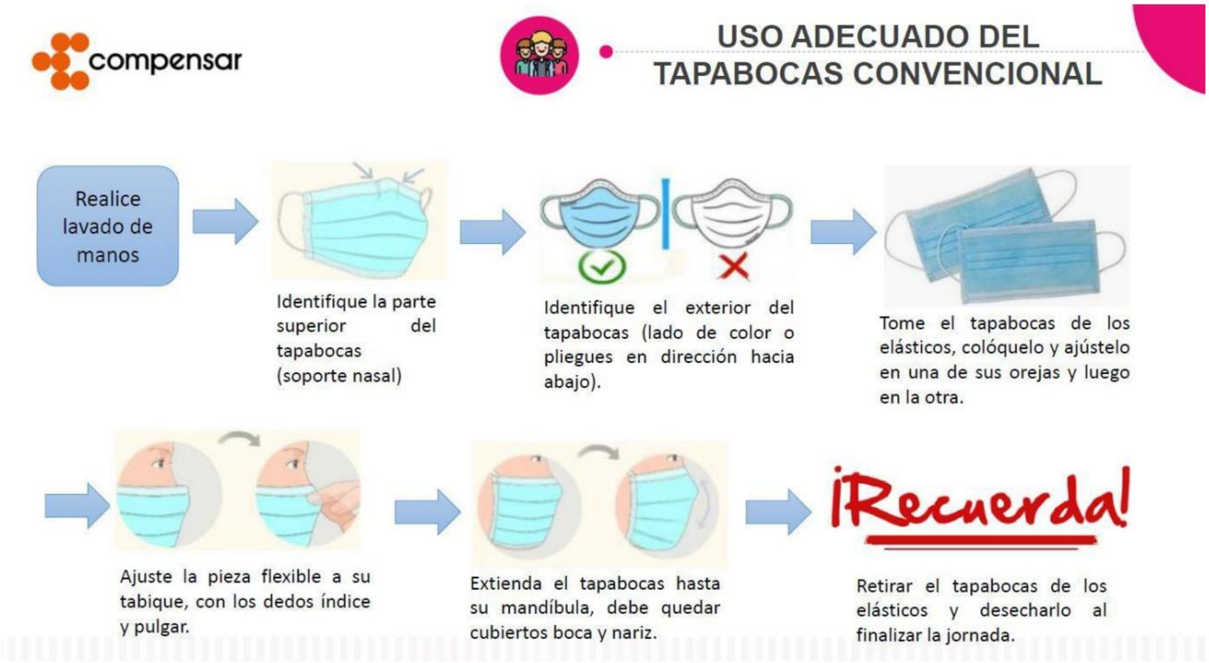

USE OF N95 MASK

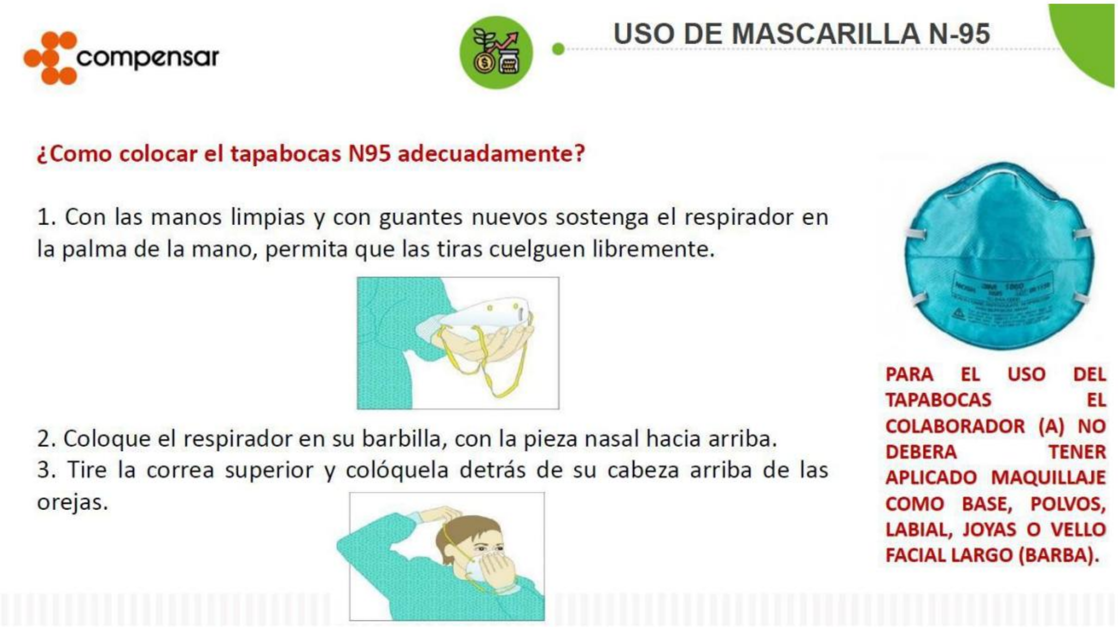

Machine Translated by Google

compensar

USO DE MASCARILLA N-95

4. Tire la correa inferior y colóquela detrás de su cabeza, debajo de las orejas y alrededor del cuello.

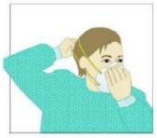

5. Coloque la punta de los dedos índice y corazón de cada mano, sobre la sobre la pieza de metal y ajuste a su nariz. Pellizcar la pieza con una sola mano es menos eficaz.
6. Cubra el frente de la mascarilla con ambas manos procurando no alterar su posición.

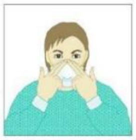

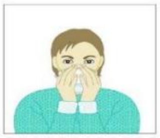

compensar

USO DE MASCARILLA N-95

7. Realice prueba de cierre positivo:

\+ Exhale bruscamente causando presión positiva dentro del respirador, si hay pérdida de aire ajuste posición y/o cintas de tensión. Repita la prueba hasta que no haya pérdida de aire.

8. Realice prueba de cierre negativo:

− Inhale profundamente, si no hay pérdidas la presión negativa hará que la mascarilla se adhiera a su rostro. Repita los pasos hasta que este correcto.

Once the intake of the scheduled patients is completed, remove the PPE according to the established order:

Machine Translated by Google

Orden para colocación y retiro de los elementos de protección personal:

COLOCACIÓN	RETIRO
1. Lavado de las manos.<br>2. Bata clínica desechable.<br>3. Gorro<br>4. Guantes<br>5. Tapabocas quirúrgico o N95.<br>6. Gafas de protección o careta.	1. Guantes<br>2. Bata o delantal.<br>3. Gafas o escudo facial.<br>4. Lavado de manos<br>5. Tapabocas<br>6. Gorro

Sample packaging and transportation:

As established in the biosafety manual, samples must be packed in triple packaging from the moment the sample is taken for subsequent transfer to the laboratory.

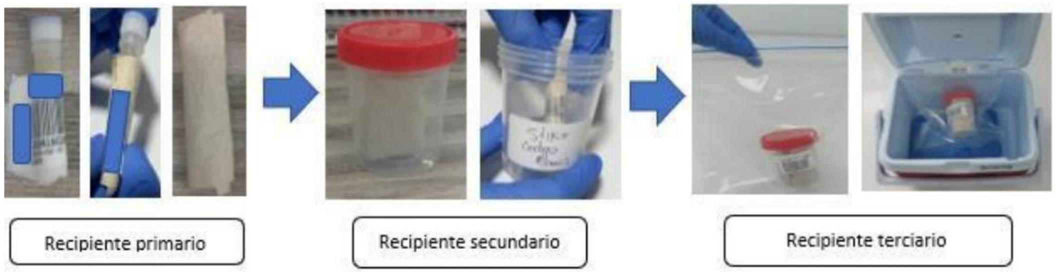

1. Cover the primary container with absorbent paper.
2. Deposit it inside the secondary container.
3. Place the secondary container in a ziploc bag and place it in the refrigerator with a battery. refrigerant. The refrigerator will be marked according to risk.
4. Upon completion of sample collection, transport to the clinical laboratory.

BIBLIOGRAPHY

- Instructions for the prevention, containment and care of covid 19. INS-PSS-576. Make up for
- Procedure for taking, transporting, preserving, and sending laboratory samples clinical. PRO-PSS-080. Make up for
- World Health Organization. Indications for hand hygiene. 5 moments to hand hygiene. May 2012
- IATA. Transport of infectious substances. 2019 version.

Machine Translated by Google

APPENDIX 2

ANNEX 2 OF LC.01.MA.02 TABLE OF PERSONAL PROTECTION ELEMENTS.xlsx

ANNEX 3

ANNEX 3 OF LC.01.MA.02 CLINICAL DATA SURVEY SARS CoV RAPID TEST 2.xlsx

ANNEX 4

ANNEX 4 OF LC.01.MA.02 INFORMED CONSENT FOR THE PROCESSING OF COVID-19 SEROLOGY TESTS.

ANNEX 5. CENTRIFUGATION OF SAMPLES

After taking the sample, transport it to the laboratory and identify the tubes to be centrifuged.
For tubes used to obtain serum, clot formation must be allowed for 30 minutes as established by the manufacturer. After this time, proceed to place the tubes in the centrifuge one in front of the other, verifying comparable volumes.
Use balance tubes if necessary.
Once the instrument balancing conditions have been validated, proceed to program the centrifuge according to the supplier's recommendations. To configure the centrifuge it is important to take into account the terms:

- RCF: Relative Centrifugal Force (Gravities)
- RPM: Revolutions per Minute (RPM)

These terms are relevant because centrifugation conditions vary by tube supplier and impact the performance of medical devices. The most used unit of measurement to express centrifugation conditions is RCF or gravities and the most common unit for configuring centrifuges is RPM, therefore it is necessary to know the procedure for the equivalence of RCF to RPM depending on the reference. of centrifuge being used.

The Rotina 380 centrifuge is available for centrifugation of blood samples:

Machine Translated by Google

**HETTICTH ROTINA 380**		
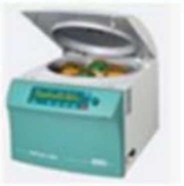	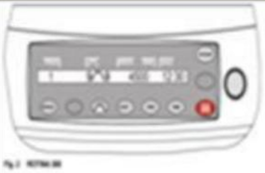	**RCF** : * Aceleración centrífuga relativa, parámtro RCF. El RCF se visualiza entre entre paréntesis)(. Se ilumina el LED en la tecla. Se puede ajustar un valor numérico del cuál resulta una velocidad de entre 50 TPM y la velocidad máxima del rotor (Nmáx). Ajustable en pasos de.
		*Radio de centrifugado, parámetro **RAD** . Ajustable de 10 mm-330mm, en pasos de 1 mm.
		* Consulta del Integral RCF. La consulta del Integral RCF sólo es posible si está activada la indicación del Integral RCF.

Rotofix 32 A centrifuge is used to centrifuge urine samples:

**HETTITCH ROTOFIX 32 A ROTOR ABATIBLE**		
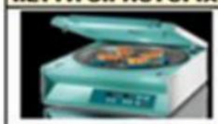	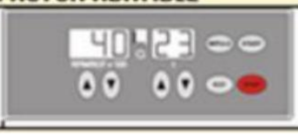	**RCF** : Indicación de la aceleración centrífuga relativa (RCF). La indicación de la aceleración centrífuga relativa (RCF) se produce mientras se mantenga pulsada la tecla **RCF** .

The insert and instructions according to the tube supplier must be verified in order to perform the centrifugation properly. Keep in mind that the tubes with gel should not be centrifuged as this may cause microparticle detachment and affect the results.

SPANISH VERSION (ORIGINAL)

1. INTRODUCCIÓN

El presente manual describe los procedimientos estandarizados de Toma de muestras de Laboratorio Clínico para pacientes de consulta externa hospitalizados, pacientes de cirugía y de urgencias con el objetivo de garantizar la calidad óptima en la fase pre-analítica del Proceso, lo cual es de vital importancia para generar un resultado seguro y confiable en las fases analíticas y post analítica.

El cumplimiento de los procedimientos que se incluyen en este Manual son responsabilidad de las colaboradoras Auxiliares de Enfermería de Laboratorio a cargo del área de Toma de muestras para pacientes de Consulta Externa, del personal asistencial: Auxiliares de Enfermería ó Jefes de Enfermería de la Clínica Universidad de La Sabana en los diferentes servicios y de Profesionales como Médicos y Terapeutas Respiratorios de la Clínica Universidad de La Sabana.

Las Bacteriólogas de Laboratorio brindarán apoyo al Auxiliar en la toma de muestras de consulta externa y supervisarán el proceso con reporte a la Administradora encargada de la sede.

Esta Manual se actualizará cada año por parte de las colaboradoras asignadas en la sede y con revisión de la Administradora.

Las principales razones para utilizar los servicios de Laboratorio son: recolectar muestras biológicas con fines terapéuticos, diagnósticos o profilácticos, descubrir enfermedades en etapas sub-clínicas, ratificar un diagnostico interrogado, precisar razones de riesgo, por lo cual es de suma importancia la eficiente recolección de las muestras para que en su posterior análisis se obtenga un resultado que aporte al personal de salud competente y ofrezca una solución de apoyo diagnóstico en pro de los pacientes.

2. OBJETIVO GENERAL

Brindar una herramienta de guía y consulta unificando los conceptos y procedimientos para la Toma de los diferentes especímenes clínicos, tanto a los colaboradores de Laboratorio como al personal asistencial de la Clínica responsable de toma de muestra en los diferentes servicios y que desee consultar.

3. OBJETIVOS ESPECÍFICOS

1. Definir el presente Manual como la Base de aprendizaje y entrenamiento para los colaboradores Auxiliares de Laboratorio de la sede Clínica Universidad de La Sabana en cuanto toma de muestras.

2. Estandarizar el procedimiento de toma de muestras en los diferentes servicios hospitalarios de la Clínica Universidad de La Sabana consolidando el trabajo en equipo.

3. Describir las condiciones de preparación que deben cumplir los pacientes para asistir al servicio de Toma de Muestras de Consulta Externa.
4. Establecer los criterios para actuar con la mayor habilidad, haciendo que el paciente no se incomode, transmitiéndole confianza, seguridad y profesionalismo durante el procedimiento demostrando buenas prácticas de Bioseguridad.

5. Garantizar una toma de muestra óptima que minimice el riesgo para el paciente.

6. Considerar al paciente como a un ser humano que en la mayoría de los casos presenta alguna enfermedad y acude al laboratorio solicitando ayuda.

7. Describir el proceso de toma de muestras extramural, contemplando la toma de muestras en empresas o instituciones y la toma de muestras a domicilio.

4. ALCANCE

Este Manual aplica para los procedimientos de Toma de Muestras de la Sede Laboratorio Clínico Compensar Clínica Universidad de La Sabana.

5. DEFINICIONES

- **SERVICIO DE TOMA DE MUESTRAS:**

Es aquél que cuenta con los recursos técnicos y humanos apropiados destinados exclusivamente a la toma de muestras y/o productos biológicos que serán procesados en el Laboratorio clínico de la sede Clínica Universidad de la Sabana con sus diferentes grados de complejidad, de los cuales dependan legal, técnica, científica y administrativamente con el fin de aumentar la accesibilidad y oportunidad al servicio por parte de los usuarios que requieren exámenes clínicos, cumpliendo de manera profesional y ética con las normas y procedimientos establecidos.

- **Muestras Biológicas:** Cualquier material humano o animal incluyendo excretas, sangre o sus componentes, tejidos y fluidos tisulares, colectados con el propósito de hacer un diagnóstico.
- **Bioseguridad:** Normas de comportamiento y manejo preventivo del personal de salud frente a microorganismos potencialmente patógenos.
- **Recipiente Primario:** Recipiente a prueba de derrames, elaborado con materiales que soportan cambios de temperaturas, presión o humedad a los cuales pueda exponerse durante el transporte, no se rompen fácilmente ni dejan escapar su contenido. Si el contenido de estos recipientes es líquido, se deben envolver en material absorbente suficiente para contener el líquido. En este recipiente está contenida la muestra. (tubo, frascos de orina, frascos hemocultivos etc)
- **Recipiente Secundario**: Segundo recipiente, a prueba de filtraciones que encierra y protege el o los recipientes primarios, frasco secundario). Debe tener material absorbente para proteger los recipientes primarios y evitar los choques entre ellos.
- **Recipiente terciario:** Elaborado de material rígido, soporta cambios de temperatura, humedad y presión a los que pueda exponerse durante el transporte. Debe ser a prueba de derrames. (Nevera de transporte)
- **Agente patógeno:** microorganismo (bacteria, virus, hongos o parásito) o microorganismo recombinado (híbrido o mutante), del que se sabe o se cree que provoca una enfermedad infecciosa en los animales o los seres humanos.
- **Estabilidad de la muestra:** capacidad de una muestra, cuando se mantiene en unas condiciones especificadas para mantener los valores de sus propiedades biológicas dentro de unos límites preestablecidos.
- **Sustancias infecciosas**: Para los fines de su transporte, se entiende por sustancias infecciosas las sustancias respecto de las cuales se sabe o se cree fundadamente que

contienen agentes patógenos. Los agentes patógenos son microorganismos (tales como bacterias, virus, rickettsias, parásitos y hongos) y otros agentes tales como priones, que pueden causar enfermedades en los animales o en los seres humanos. La definición se aplica a todas las muestras excepto a las excluidas explícitamente (véase lo indicado más adelante). Las sustancias infecciosas se dividen en dos categorías.

- **Ficha de Información**: La ficha contiene y describe las condiciones y requerimientos necesarios para las pruebas realizadas en el Laboratorio Clínico de Compensar o red de apoyo. El personal asistencial de la Clínica Universidad de La Sabana puede consultar la Ficha de Información de Servicios de Compensar a través de la carpeta compartida para tal fin desde la Jefatura de Enfermería de la Clínica.
- **Anticoagulantes:** Son sustancias que previenen la formación de coágulos. Existen diferentes tipos de ellos en polvo o líquidos. Debe seleccionarse siempre el anticoagulante apropiado según el estudio que se requiera realizar. Los anticoagulantes más comúnmente utilizados son: EDTA, Citrato de Sodio, Heparina, Oxalatos.
- **Citrato de Sodio:** Anticoagulante que se utiliza generalmente en concentraciones al 3.8% en estudios de coagulación. Funciona por quelación del calcio.
- **EDTA: (ETILEN-DIAMINO-TETRA-ACETATO)** Este tipo de anticoagulante es utilizado principalmente cuando se realizan estudios en donde se cuentan células. Funciona quelando el calcio.
- **Heparina:** Se utiliza tanto en algunos estudios de rutina como especializados. Su presentación puede incluir heparina con concentraciones de sodio o litio. En general, la heparina con litio es utilizada para estudios de química y la heparina sódica se utiliza para estudios de linfocitos. Actúa acelerando la inhibición del factor Xa por la antitrombina.
- **Exógeno:** Cualquier factor o mecanismo agregado en la muestra in vivo (es decir, un fármaco) o in vitro (es decir, un contaminante).
- **Factor de Influencia:** Influencia biológica (in vivo e in vitro) sobre el valor de una magnitud biológica en un sistema (ej. Sangre venosa)
- **Factor de Interferencia:** Componente de la matriz de una muestra que difiere del analito e interfiere con el procedimiento analítico para dar una señal de medida falsa.
- **COVID-19:** Enfermedad infecciosa causada por Coronavirus descubierto en el año 2019. La enfermedad Coronavirus 2019 es el nombre aasignado a la patología causada por la infección del coronavirus del Sindrome Agudo Respiratorio Agudo, Severo o Grave 2.

6. CAPÍTULOS

1. GENERALIDADES

1. TIEMPOS EN LA TOMA DE MUESTRA:

Existen dos tipos de tiempos en los exámenes. El primer tipo (tiempo simple), cuando se trata de un examen que solo requiere de una punción. El segundo tipo (tiempo múltiple), cuando el examen amerita y comprende diferentes muestras en diferentes tiempos. En cualquiera de los casos, debe tenerse en cuenta:

a. Las condiciones de preparación del paciente deben ser correctamente adoptadas.
b. Algunos pacientes toman medicamentos durante el día, que pueden interferir con los resultados; esto debe ser tenido en cuenta en el momento del análisis de los exámenes.
c. Si se le ordena al paciente regresar al laboratorio para tomar segundas muestras, deben

suministrarse las recomendaciones que debe seguir el paciente, mientras llega el momento para los estudios posteriores.

2. PRECAUCIONES DE BIOSEGURIDAD EN TOMA DE MUESTRA

La manipulación inapropiada puede convertirse en una fuente de riesgo biológico para las personas que están en contacto con las muestras o para el medio ambiente. Utilizar los elementos de protección personal necesarios para evitar exposición con riesgo biológico, de acuerdo con la fuente de la muestra.

En caso de accidente con riesgo biológico, avisar inmediatamente según las recomendaciones del protocolo de accidente de trabajo con riesgo biológico institucional. Ver Manual de Bioseguridad.
El personal auxiliar y/o bacteriólogas deben seguir todas las normas de bioseguridad para la atención de pacientes, Ver Manual de Bioseguridad.

CONTENEDORES DE BIOSEGURIDAD

- Contenedores para especímenes, a prueba de fugas y de fácil sellamiento.
- Cumplir con las recomendaciones de manejo de elementos cortopunzantes:
- No re enfundar agujas.
- Disponer y utilizar adecuadamente el contenedor para corto punzante.

ELEMENTOS DE PROTECCIÓN PERSONAL

- Protección ocular: gafas o mascarilla con visera.
- Mascarilla y/o tapabocas.
- Guantes desechables.
- Bata anti fluido o desechable.
- Gorro.

3. RECOLECCION DE MUESTRAS DE SANGRE.

La punción venosa es uno de los procedimientos más comunes a que son sometidos los pacientes en el Laboratorio Clínico, conlleva un riesgo para el paciente, ya que es abrir una Puerta peligrosa a un área totalmente aséptica como es la sangre, es por eso que toda veno punción lleva implícito el riesgo de la infección si no se tiene en cuenta todas las técnicas asépticas. La infección puede ser causada por: equipos contaminados, contaminación con microorganismos por vía aérea, por mala técnica aséptica durante la venopunción. Estos riegos pueden ser minimizados realizando el procedimiento con una estricta técnica aséptica y manteniendo cuidados en el sitio de la venopunción.

GENERALIDADES EN LA VENOPUNCION:

- El personal encargado de la toma y recolección de muestras de Laboratorio está constituido por Auxiliares de enfermería y/o Laboratorio certificados y entrenados en el área quienes asumen la responsabilidad de la correcta toma de muestras. Para el proceso de la toma de muestras se cuenta con la asesoría y soporte del coordinador del Laboratorio y del grupo de profesionales de Bacteriología. Para la toma de muestras de pacientes hospitalarios el personal de enfermería es el encargado de tomar las muestras en los servicios y de su

entrega al laboratorio.

- Para la toma de muestras de sangre se tienen asignados cubículos especiales para tal fin en el laboratorio clínico. El proceso es realizado por una auxiliar exclusiva para esta actividad, garantizando la confidencialidad en la atención para todos los pacientes, incluyendo los menores de edad.
- La auxiliar responsable de la toma de la muestra, verificará el riesgo de caídas de acuerdo al uso de botón de color rojo para adultos, manilla de color azul para menores en áreas ambulatoria y botón amarillo para el acompañante. De acuerdo con las metas de seguridad de paciente.
- Los exámenes son tomados según requerimiento medico en el horario de 6:00 am a 10:00 am por consulta externa y durante el todo el día son recepcionados desde los diferentes servicios de la clínica. (La toma de las muestras en el servicio de urgencias, pacientes de UCI que no tengan catéter central y la ronda matutina de hospitalización está a cargo del personal del laboratorio).
- Se debe verificar que los pacientes cumplan con las condiciones requeridas para la toma de la muestra antes de la venopunción.
- Se debe considerar que todas las muestras de especímenes biológicos deben ser tratadas como potencialmente infecciosas.
- Se debe Obtener suficiente y adecuada cantidad y calidad de sangre de acuerdo con el tipo de examen.
- Vigilar que los elementos de trabajo estén en perfectas condiciones físicas, fechas de vencimiento, limpieza, desinfección y/o esterilización.
- Mantener el Laboratorio limpio y ordenado, evitando la presencia de material y equipo que no tengan relación con el trabajo.

ELEMENTOS PARA LA TOMA DE MUESTRAS.

TUBOS PARA MUESTRAS DE SANGRE

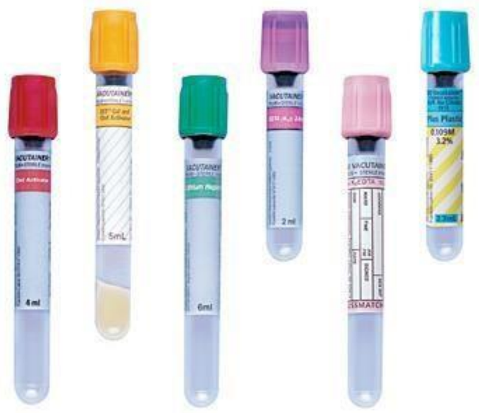

TAPA LILA: Contiene EDTA como anticoagulante, el cual se llena al vacío de acuerdo con el volumen que especifique el tubo teniendo en cuenta que existen tubos pediátricos y adulto que requieren volúmenes de sangre diferentes. Se utiliza para la determinación de pruebas de hematología y servicio transfusional.

En la toma de muestras con este tipo de tubo, es de vital importancia garantizar la relación adecuada entre sangre y anticoagulante. Para esto se debe siempre verificar en el tubo el volumen de sangre

que requiere. Si se toma la muestra con sistema de vacío este ayuda a mantener la relación, si es con jeringa se debe llenar hasta la marca indicada por el fabricante.

TAPA AMARILLA/ROJA: Es un tubo que contiene un gel que permite la separación de la muestra (suero – paquete globular), se debe llenar de acuerdo con el volumen que especifique el tubo teniendo en cuenta que existen tubos pediátricos y adulto que requieren volúmenes de sangre diferentes. Este tubo permite que, posterior al proceso de centrifugación, el paquete globular quede separado por el gel del suero en el que se realizan las determinaciones. Se utiliza para mediciones de química sanguínea, serologícas entre otras. Tubo contiene una sustancia procoagulante que acelera el proceso de coagulación de la sangre.

TUBO TAPA ROJA SIN GEL: Tubo que no contiene ninguna sustancia procoagulante ni gel. Este tubo se emplea para realización de pruebas que generan interferencias con los procoagulantes o el gel como es el caso del alcohol metílico.

TAPA GRIS: Este tubo posee como anticoagulante fluoruro de sodio. Se utiliza para medición de alcohol etílico y lactato semiautomatizado por algunas técnicas. En el laboratorio compensar no se utiliza rutinariamente.

TAPA AZUL: Este tubo contiene citrato de sodio como anticoagulante, el cual se utiliza para pruebas de coagulación. Para las pruebas de coagulación es de vital importancia la conservación de la realacón sangre-anticoagulante. Se debe llenar de acuerdo con el volumen que especifique el tubo teniendo en cuenta que existen tubos pediátricos y adulto que requieren volúmenes de sangre diferentes. se debe centrifugar lo más pronto posible para evitar el consumo de factores de coagulación, se puede guardar a temperatura ambiente o refrigerada hasta su traslado que de preferencia no exceda las 2 horas después de tomado.

TAPA VERDE: Contiene heparina de sodio como anticoagulante. Utilizado para determinación de gases venosos y marcadores cardíacos.

ORDEN EN EL LLENADO DE LOS TUBOS:

De acuerdo con las recomendaciones del fabricante se determina el orden de llenado de tubos:

Orden de Toma

Tapón	Contenido de tubo	Área de uso	Inversiones
	Hemocultivo	Microbiología	5 veces
	Citrato de sodio	Coagulación (Tiempos de coagulación fibrinógeno, agregación plaquetaria)	3 a 4 veces
	Gel separador	Química clínica	5 veces
	Sin anticoagulante, con activador de coagulación, con silicón	Química clínica, banco de sangre serología	8 a 10 veces
	Gel separador y trombina	Obtención de suero rápido	5 a 6 veces
	Gel separador y heparina de litio	Química clínica en plasma	5 veces
	Heparina de sodio/litio	Química clínica (urgencias) hematología (fragalidad osmótica)	8 a 10 veces
	EDTA K_2	Hematología, banco de sangre	8 a 10 veces
	Gel separador y EDTA K_2	Determinaciones de carga viral	8 a 10 veces
	Oxalato de Potasio/NaF	Química clínica, pruebas de lactato y glucosa	8 veces

1. Tabla Orden de Toma. Sistema BD Vacutainer Recolección de Sangre Venosa

MEZCLADO DE TUBOS: La adecuada homogenización de las muestras sanguíneas garantiza el óptimno desempeñode los dispositivos médicos, contribuyendo a la obtención de resultados confiables durante la fase analítica y post analítica. Recuerde homogeniza estos dispositivos como se ilustra a continuación:

INSTRUCTIVO PARA MEZCLA DE TUBOS LABORATORIO CLINICO COMPENSAR compensar

DISPOSITIVO MEDICO	MEZCLA POR INVERSION (De techo a piso)		IDENTIFICACION TAPA DE TUBO POR COLOR
TUBO TAPA AMARILLA	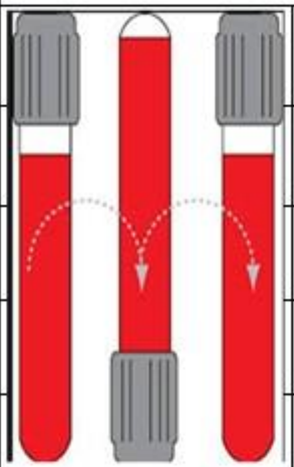	5 - 8 Veces	
TUBO TAPA AZUL		3-4 Veces	
TUBO TAPA VERDE (HEPARINA DE LITIO), LILA Y GRIS		8-10 Veces	
TUBO TAPA VERDE, LILA Y GRIS, PERLA		8-10 Veces	
TUBO TAPA VERDE (HEPARINA DE LITIO Y GEL SEPARADOR)		5 Veces	

- Después de la extracción de la muestra, los tubos se deben mantener siempre de forma vertical en una gradilla, propiciando el debido proceso de retracción del coágulo de la muestra, el cual ocurre generalmente después de 30 minutos posterior a la toma de la muestra.
- Almacenamiento y transporte: Las muestras deben ser centrifugadas, conservadas, almacenadas y transportadas según los requerimientos expuestos en la ficha de información la cual se encuentra disponible en la intranet de Compensar.

SISTEMA VACUTAINER

- HOLDER O CAMISA: Contenedor plástico de color amarillo o transparente en donde se ensambla la aguja y que en su parte inferior posee una capucha en donde se insertan los tubos para la toma de muestra.

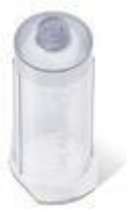

- AGUJAS: Están dentro de cápsulas verdes y tiene 2 partes una cubierta de goma en donde se insertan los tubos, y la aguja que es con la cual se punciona al paciente.

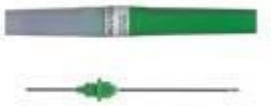

- ADAPTADOR MARIPOSA: Este permite adaptar el sistema Vacutainer para uso de mariposas, en pacientes de difícil acceso venoso o en lactantes y niños.

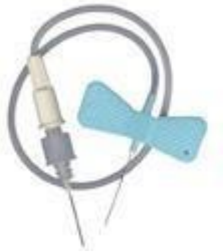

- **SISTEMA MICROTAINER:** Tubos utilizado para toma de muestras pediátricas o para pacientes con difícil acceso venoso. En la clínica se dispone de tubo microtainer tapa lila para hematología y tapa azul para coagulación y tapa amarilla para pruebas en suero.

JERINGAS SIN ADITIVOS
Se deben utilizar de manera mínima y sólo en casos de muy difícil acceso venoso. De acuerdo al volumen requerido de muestras existen jeringas de 3ml, 5ml y 10 ml.

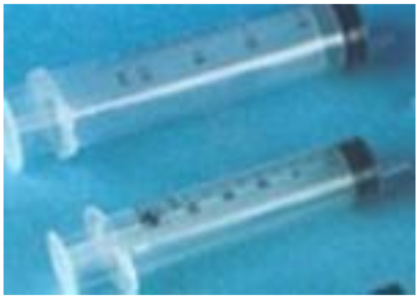

2. PROTOCOLO DE ATENCION EN TOMA DE MUESTRAS

Para el dilienciamiento de todos los consentimientos informados aplicar los 5 correctos definidos:

CORRECTOS PARA EL DILIGENCIAMIENTO DEL CONSENTIMIENTO INFORMADO

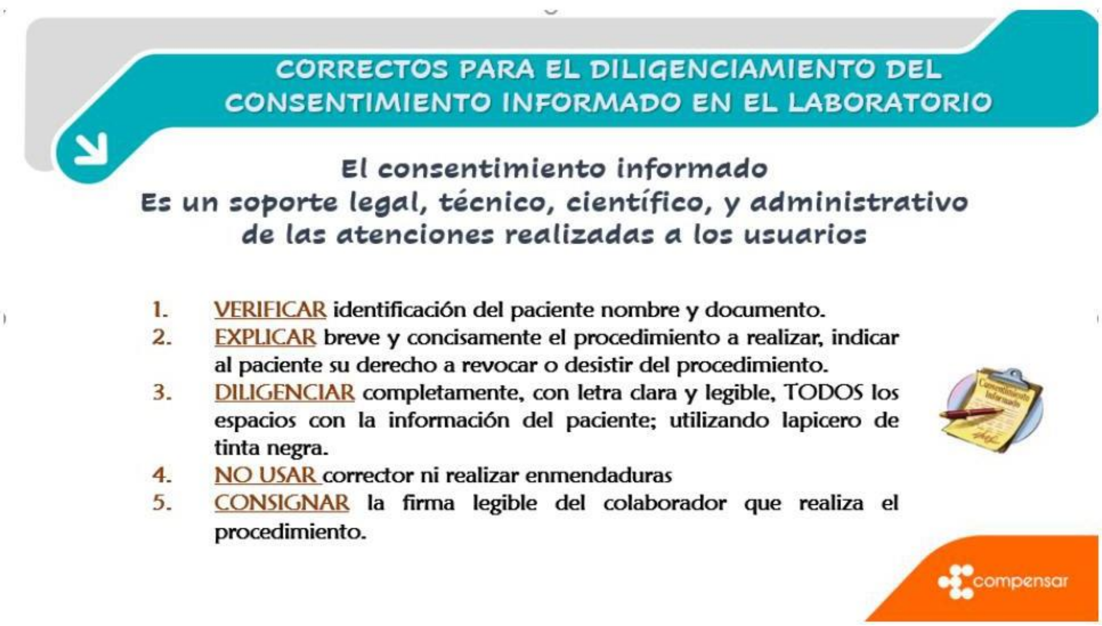

CONSENTIMIENTO INFORMADO PARA TOMA DE MUESTRAS DE LABORATORIO CLINICO

Este procedimiento está definido para la atención de pacientes de Consulta Externa, y pacientes de urgencias ambulatorios. Pacientes de observación e internación el consentimiento está inmerso en el del servicio. El formato se encuentra en el portal clínica con el código LC.01.MA.02.FT.01. La firma de este consentimiento se da luego de que se le explica al paciente los riesgos asociados a la toma de muestra, complicaciones y eventos que pueden producirse como efecto del procedimiento. También se incluye la denegación para que el paciente tenga la opción de negarse al procedimiento.

CONSENTIMIENTO INFORMADO PARA ADMINISTRACIÓN DE CARGAS DE GLUCOSA

Todo paciente con orden de glucosa pre y post carga, debe dar su consentimiento por medio del formato LC.01.MA.02.FT.02 el cual se encuentra disponible en portal clínica. Previo a la firma, se debe explicar al paciente el procedimiento que se va a realizar y los riesgos asociados a la ingesta de cargas de glucosa.

CONSENTIMIENTO INFORMADO PARA REALIZACIÓN DE PRUEBAS DE VIH

Todo paciente con orden de examen para VIH debe dar su consentimiento. El proceso de asesoría Pre test y post test está a cargo del médico tratante quien posterior a la asesoría realizara el dligenciamiento del formato DM.01.PR.01.FT.34. Ante orden de examen de VIH, auxiliar de laboratorio debe verificar la existencia de este consentimiento verificando la firma del paciente o acudiente que consciente la realización de la prueba. Esto se debe realizar con ordenes tante de consulta externa como de internación y urgencias. Para consulta externa solo se recibirán consentimientos realizados en la institución lo cual garantizará una atención posterior para la asesoría post test.

CONSENTIMIENTO INFORMADO PARA EL PROCESAMIENTO DE PRUEBA RÁPIDA DETECCIÓN DE ANTÍGENO SARS CoV 2 (COVID-19)

Prueba rápida para diagnóstico de COVID 19.

Esta prueba aplica para:
* Persona con síntomas de menos de 11 días, atendida en ámbito de urgencias u hospitalización, donde por las condiciones territoriales no se tenga la capacidad para realizar pruebas moleculares RT-PCR.
*En los servicios ambulatorios o domiciliarios a personas sintomáticas y grupos de riesgo priorizados. Al contacto asintomático no conviviente con el caso confirmado, dentro de un estudio de cerco epidemiológico.
*Personas que vivan en zonas rurales dispersas

APLICA PARA PACIENTE AMBULATORIO O DOMICILIARIO:
*Persona atendida en el ámbito domiciliario o ambulatorio: En persona con sintomatología de COVID-19 con menos de 11 días de inicio de síntomas. Se considera sintomático de COVID-19 una persona con uno o más de los siguientes síntomas: fiebre, tos, dificultad respiratoria, odinofagia y/o fatiga/astenia. Estos síntomas pueden acompañarse o no de síntomas gastrointestinales como diarrea, vómitos, dolor abdominal y otros como disgeusia (pérdida del gusto) o anosmia (pérdida de olfato).
*Persona asintomática que es contacto estrecho de un caso o fallecido confirmados con COVID-19: Se tomará prueba RT-PCR o prueba de antígeno al día 7 de la exposición con el caso.

Persona asintomática con o sin factores de riesgo que es contacto estrecho de un caso o fallecido confirmados con COVID-19:
***** Tomar RT- PCR o prueba de detección de antígeno al día séptimo del contacto estrecho no protegido o fallecido confirmados.

Esta prueba no aplica para:

- Persona que su inicio de síntomas o fecha de exposición sea mayor a 11 días.

No requiere ayuno estricto (A menos que la solicitud esté acompañada de exámenes que si lo requieran). Tomar muestra de hisopado nasofaríngeo según protocolo en anexo 1.

Aplicar "*ENCUESTA DE DATOS CLÍNICOS PRUEBA RÁPIDA SARS CoV 2 (COVID-19) DETECCIÓN DE ANTÍGENO"* ANEXO 3

CONSENTIMIENTO INFORMADO PARA EL PROCESAMIENTO DE PRUEBAS SEROLÓGICAS SARS Cov 2 (covid 19)

La prueba de detección cualitativa de anticuerpos IgG contra Virus SARS CoV-2 aplica para personas con síntomas leves probable de COVID-19, sin factores de riesgo, que tengan por lo menos 11 días desde el inicio de los síntomas. Si la persona presenta menos de 11 días de síntomas, se debe aplicar la prueba molecular RT-PCR.2.
Esta prueba no aplica para personas atendidas en ámbito domiciliario o ambulatorio que presente los siguientes factores de riesgo:
1. Ser trabajador de la salud que tuvo contacto con un caso probable o confirmado de COVID-19.
2. Persona adulta mayor de 70 años.
3. Persona de cualquier edad que presente sintomatología de COVID-19 con comorbilidades (diabetes, enfermedades cardiovasculares, hipertensión arterial, enfermedad cerebrovascular, enfermedad respiratoria crónica, VIH u otra inmunodeficiencia, cáncer, enfermedades autoinmunes, uso prolongado de esteroides,insuficiencia renal, obesidad, desnutrición) y tabaquismo.
4. Persona asintomática con o sin factores de riesgo, contacto estrecho de un caso confirmado con COVID-19 o contacto estrecho de persona fallecida por COVID-19 confirmado.
6. Búsqueda activa en grupos poblacionales: evaluar en primera instancia la presencia de síntomas sugestivos de COVID-19. Si los síntomas son de inicio reciente (igual o menor a 10 días).

Se define como Contacto estrecho: menos de 2 metros durante 15 minutos con un caso confirmado COVID 19.
No se deben utilizar estas pruebas para pacientes asintomaticos o para tamizajes. No requiere ayuno estricto (A menos que la solicitud este acompañada de exámenes que si lo requieran). Tomar muestra en tubo seco.
Aplicar "Consentimiento Informado para el procesamiento de pruebas serológicas SARS CoV 2 (COVID 19)". ANEXO 4

CADENA DE CUSTODIA

La Cadena de Custodia es un sistema que permite garantizar la autenticidad de los elementos físicos y materia de prueba para demostrar y asegurar que las condiciones de identidad, integridad, preservación, seguridad, continuidad y registro de los mismos, se aplican desde que inicia hasta que termina la cadena.

Registro de cadena de custodia: Es la historia exhaustiva y documentada de cada traspaso y traslado del material físico de prueba, durante el desarrollo del proceso de cadena de custodia. Permite verificar la identidad, el estado y condiciones originales de los elementos físicos de prueba, así como las modificaciones realizadas a ésta, establece la ruta seguida por dichos elementos, determina su lugar de permanencia y la persona responsable de la custodia en cada lapso del procedimiento.

Para la determinación de las pruebas de drogas de abuso y alcoholemia, se requiere diligenciamiento del FOR PSS 474 Cadena de custodia laboratorio Clinico para muestras toxicológicas o microbiológicas.

Para el caso de toxicología en orina, en los servicios de urgencias e internación, jefe de enfermería debe hacer el acompañamiento al paciente durante la recolección de la muestra y entregarla al personal de laboratorio con la cadena de custodia. Para pacientes de consulta externa, la auxiliar de toma de muestras realizará el acompañamiento y verificación de la recolección de la muestra y diligenciamiento inicial de la cadena de custodia.

FICHAS EPIDEMIOLÓGICAS

Para los eventos de notificación obligatoria a SIVIGILA, el médico tratante debe realizar el diligenciamiento de la ficha de notificación epidemiológica que corresponde. Estas fichas son dejadas en el buzon de epidemiología sin no requieren remisión de muestras a Laboratorio de Salud Publica. En caso de envío de muestras a LSP se debe solictar esta ficha para ser enviada junto con las muestras.
Algunos eventos de notificació son:

- Infección respiratoria aguda ESI-IRAG
- IRAG
- Sarampión
- Rubeola
- Tosferina
- Bordetella
- Brucella

FORMATOS ADICIONALES

En algunos casos, de acuerdo a las pruebas solicitadas, y segpun los requisitos mencionados en la ficha de información de compensar, se deben solictar formatos adicionales. Algunos ejemplos son exámenes de genética, errores del metabolismo entre otros. Es indispensable la verificación de condiciones y requisitos para procesamiento de pruebas especiales en la ficha de información.

3.

Para la atención a pacientes de consulta externa se tiene definido el horario de lunes a viernes de 6:00 am a 10 am y sabados de 6:30 a 10:00 am. Se cuenta con una auxiliar para la toma de muestras

y una auxiliar para el proceso de atención en ventanilla previo a la toma de la muestra. A continuación, se describen las actividades a realizar:

- Se realiza el llamado a través de de digiturno. Auxiliar de línea de frente realiza la primera atención en la cual se verifican los datos, la factura y orden y se verifica el cumplimiento de las condiciones para la toma de los exámenes. Para esto, se debe verificar en la ficha de información de laboratorio clínico de acuerdo con los exámenes ordenados, que condiciones requiere el paciente. En caso de incumplimientos, se debe redireccionar al paciente a la ventanilla de acceso donde consolidan la información de devolución de pacientes para registro de indicador.
- Si se da cumplimiento a los requisitos, se realiza el ingreso por el sistema de información y se redirecciona al paciente al módulo de toma de muestras. Previamente se informa el tiempo de respuesta de los exámenes y el horario de entrega de resultados. Es importante informar al usuario la posibilidad de envío de resultados por correo electrónico previo diligenciamiento de autorización.
- Durante el proceso de atención para la toma de muestra, auxiliar debe dar cumplimiento a los correctos de toma de muestra que se enumeran a continuación y están descritos en el modelo de atención del laboratorio clínico:

CORRECTOS TOMA DE MUESTRA

1- Saludar al paciente

> Buen día **Señor (a)**.... mi nombre es ..... Auxiliar de laboratorio clínico quien le va a tomar las muestras para los exámenes solicitados

2- Preguntar al paciente el nombre y número de documento o al acudiente según corresponda.
3- Revisar exámenes solicitados versus facturados, verificar condiciones requeridas cuando apliquen y registrar datos de interés clínico para el paciente.
4- Explicar los riesgos asociados a toma de muestras. Consultar al paciente o acudiente si se tiene alguna duda y hacer firmar el consentimiento informado como constancia de su entendimiento.
5- Alistar el material requerido y marcar los tubos
6- Seleccionar el sitio de venopunción y dar las indicaciones al paciente para que se haga presión cuando no acepta la banda o que se la retire luego de quince minutos.
7- Mencionar el deber y derecho de la semana

Las muestras de Gases Arteriales de Consulta Externa son tomadas por la Auxiliar del Laboratorio clínico. En caso de ser una punción de difícil acceso se solicita el apoyo de la terapeuta Respiratoria

de disponibilidad.

Dar cumplimiento en el lavado de manos según lo establecido:

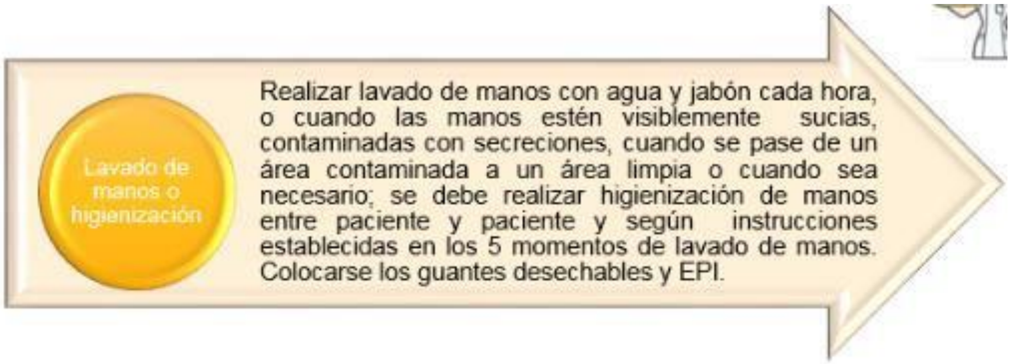

4. TOMA DE MUESTRAS EN PACIENTES DE INTERNACIÓN Y URGENCIAS

El laboratorio clínico realiza la toma de muestras a los pacientes del servicio de urgencias. Para esto, se dispone de dos auxiliares en el servicio durante el día la noche. Para los pacientes que se encuetren en el servicio de reanimación y pediatría, quienes realizan la toma de las muestras son el personal de enfermería de la clínica, así como la toma de muestras ginecológicas, microbiológicas y acompañamiento para recolección de orinas de toxicologicos. Para los demás usuarios, se debe realizar la toma de las muestras por parte de los auxiliares de laboratorio clínico.
En los servicios de internación, se tiene definido la toma de exámenes de rutina por parte del laboratorio clínico.
Para los servicios de UCI intermedia y UCI adultos todas las muestras serán tomadas por personal de enfermería. Igualmente para los pacientes pediátricos en cualquier servicio de la Clínica.

En todos los casos se debe tener en cuenta lo siguiente:

- Verificar las solicitudes en sistema de información Hosvital. En urgencias, realizar la busqueda y llamado a los pacientes ambulatorios y dirigirlos al módulo de toma de muestra.
- En caso de pacientes en camilla, silla o que no puedan desplazarse, el auxiliar mobil debe tomar la muestra donde se encuentre ubicado el paciente.
- Verificar orden en sistema y definir los tubos a tomar. Realizar el procedimiento teniendo en cuenta los correctos para toma de muestra:

CORRECTOS TOMA DE MUESTRA

1- Saludar al paciente

Buen día **Señor (a)**.... mi nombre es ..... Auxiliar de laboratorio clínico quien le va a tomar las muestras para los exámenes solicitados

2- Preguntar al paciente el nombre y número de documento o al acudiente según corresponda.
3- Revisar exámenes solicitados versus facturados, verificar condiciones requeridas cuando apliquen y registrar datos de interés clínico para el paciente.
4- Explicar los riesgos asociados a toma de muestras. Consultar al paciente o acudiente si se tiene alguna duda y hacer firmar el consentimiento informado como constancia de su entendimiento.
5- Alistar el material requerido y marcar los tubos
6- Seleccionar el sitio de venopunción y dar las indicaciones al paciente para que se haga presión cuando no acepta la banda o que se la retire luego de quince minutos.
7- Mencionar el deber y derecho de la semana

- Siempre realizar marcación de los tubos frente al paciente y previa verificación de datos.
- Tener en cuenta las definiciónes del modelo de atención del laboratorio.

5. SELECCIÓN DEL SITIO DE PUNCIÓN:

Las venas más utilizadas para la venopunción, están localizadas en el área ante cubital. Entre éstas tenemos:

- Vena Cubital: Es la más larga y gruesa de todas y es la preferida por bordear la musculatura del brazo.
- Vena Cefálica: Tiene iguales características de la anterior, pero es un poco menos gruesa.
- Vena Basílica: Es más pequeña que las anteriores. Esta vena está cerca de la arteria braquial, por lo que su punción es riesgosa y su área es más sensible y dolorosa para el paciente.

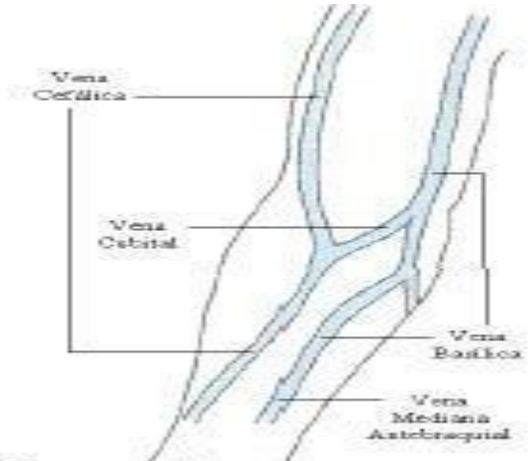

Venas del brazo

- Coloque el torniquete 2 a 3 centímetros por arriba del sitio seleccionado, para visualizarlas mejor. Su uso no debe externderse por mas de 1 minuto.
- Adoptar postura correcta: el brazo del paciente debe estar colocado en línea recta y apoyarse firmemente en apoyabrazos o superficie de trabajo, sin doblarse a nivel del codo. Para la atención de bebes se acostarán sobre la mesa de toma de muestras utilizando la colchoneta pidiéndole a la mamá o al acompañante sostener al bebe para evitar que se caiga o mueva durante la toma de la muestra.
- Debe evitarse zonas con hematomas, quemaduras, tobillos o pies en pacientes diabéticos y con trastornos circulatorios. Seleccionar brazo contrario en caso de post cirugía de vaciamiento ganglionar por cirugía de seno.
- Una vez escogida la vena realizar la asepsia en la zona con un algodón impregnado de alcohol en forma circular del centro a la periferia dejando secar el alcohol. Colocar la punta de la aguja a un 1 cm por debajo de la vena seleccionada.
- Puncionar la vena con el bisel de la aguja hacia arriba en dirección del flujo sanguíneo.
- Sostener la aguja en ángulo de 30 a 40 grados con relación a la piel y al tejido celular subcutáneo para llevarlo a la vena.
- Una vez haya realizado la punción inserte los tubos en el sostenedor y/o camisa para que la sangre fluya libremente en el orden establecido.
- Si utiliza jeringa incline la aguja hasta que esté casi paralelo a la superficie de la piel y puncione la vena lentamente verificando la penetración por el reflujo de sangre en la cámara.

6. COMPLICACIONES

COMPLICACIONES				
**DEFINICIÓN**	**COMO SE EVITA**	**QUE HACER SI SE COMPLICA**	**CAUSAS**	**CLASIFICACIÓN**
**INFECCIÓN O FLEBITIS:** Es la inflamación de la vena debido a una alteración del endotelio, con migración de plaquetas a la zona lesionada, lo que produce liberación de histamina, aumenta el flujo sanguíneo en la zona por vasodilatación, luego hay un aumento de la permeabilidad capilar, lo que permite la extravasación de la proteína y líquidos y se produce un acumulo de linfocitos en el lugar inflamado.La inflamación en la pared de una vena se produce generalmente como consecuencia de una mala circulación o por las malas prácticas clínicas durante los procedimientos de Venopunción y que puede llegar a ser potencialmente peligrosa en la medida de que de la zona afectada se desprenda un trombo.<br>Pueden ser de dos tipos: superficial y profunda siendo estos últimos los menos comunes pero los más graves y difíciles de manejar.<br>Se caracterizan por producir: inflamación local en el sitio de punción, dolor en la parte afectada del cuerpo, enrojecimiento de la piel, calor y sensibilidad sobre la vena.	* Mediante la aplicación de la técnica aséptica adecuada descrita en este instructivo; Cumplir con lo establecido en el protocolo de toma, transporte y conservacion de muestras. *Evitar realizar venopunciones en áreas cercanas a lesiones de piel y realizar en lo posible una sola venopunción<br><br>*.Verificar que antes de la puncion la solución alcoholica haya secado completamente.<br><br>Elija la vena de mejor calibre y acorde con la aguja a utilizar.Se debe elegir la aguja de menor calibre con respecto a la vena, que cumpla con el objetivo de la punción de modo que se evite trauma o irritación local. Evite utilizar venas que:<br>a)Se encuentren en zonas de flexión.<br>b)Sean muy visibles, ya que tienden a desplazarse apartándose de la aguja.<br>c)Estén dañadas por uso previo, flebitis, infiltración o esclerosis. d)Estén continuamente distendidas con sangre, o que se hayan vuelto nudosas o tortuosas.<br>e)Se encuentren en una extremidad lesionada o quirúrgicamente comprometida, debido a una posible alteración de la circulación y molestias para el paciente.<br>Verificar que la piel del sitio de punción debe estar intacta, sin rastros de eritemas, erritaciones y/o escoriaciones. I<br>De preferencia se deben utilizar las venas de las extremidades superiores (VER CAPITULO 3 DEL PRESENTE PROTOCOLO)	Solicitar a atención médica	• Manipulación: Barreras de seguridad ineficaces.<br>• La condición clínica del paciente.<br>• La condición de la vena.<br>* Aplicación de inadecuada Técnica de punción: Uso incorrecto de procedimiento de asepsia y ejecución de multipunciones.<br>• Fijación: Falla en fijación de la vena. (Recuerde, estabilice la vena manteniendo la piel tensa en sentido contrario a la dirección de la punción, con el fin de lograr la colocación atraumática de la aguja, coloque el bisel de la aguja sobre la vena y hacia arriba, en un ángulo de 30 a 45 grados y penetre en dirección del flujo venoso y mantenga fija la aguja sin realizar movimientos bruscos para minimizar el traumatismo de la pares del vaso, e inserte el dispositivo de vacío para la colección de la muestra.)	Presenta tres factores asociados:<br>• Infecciosa: se evidencia después de 72 horas. Inflamación originada por cualquier tipo de microorganismos causada por falta de aplicación de una adecuada técnica aséptica.<br>• Química: irritación de la vena por soluciones acidas, alcalinas o hipertónicas (no aplica en la práctica de toma de muestras).<br>• Mecánica: por mala manipulación, inadecuado sitio de inserción (zona de flexión, tortuosidad de la vena). Vena multipuncionada, fallas en la técnica de punción, condición de la vena.

COMPLICACIONES				
**DEFINICIÓN**	**COMO SE EVITA**	**QUE HACER SI SE COMPLICA**	**CAUSAS**	**CLASIFICACIÓN**
**HEMATOMA:** Acumulación de sangre que se forma cuando hay una lesión en los vasos sanguíneos pequeños que causa sangrado dentro de los tejidos.	• Técnica adecuada de venopunción donde se perfore la vena solo un sitio.<br>• Elegir una vena con buen tejido de soporte.<br>• Liberar rápidamente el tornique usar agujas con buen bisel, el cual debe colocarse hacia arriba para la punción y del mínimo o calibre posible.	• Suspender la punción.<br>• Presionar directamente sobre el sitio de la punción con un algodón seco, sin alcohol, ejerciendo presión firme y constante durante mínimo tres minutos.<br>• Si el hematoma continuo aumentando con estas medidas se llama al médico.	* Si el paciente presiona firmemente la zona de la extracción, en esas circunstancias factores como: Venas frágiles, Extracciones dificultosas por venas inaccesibles o muy finas<br><br>* Medicamentos como la aspirina o los anticoagulantes.<br>* En las siguientes patologías como hemofilia, enfermedad de von Willebrand, trombocitopenia.	NO APLICA

COMPLICACIONES				
DEFINICIÓN	COMO SE EVITA	QUE HACER SI SE COMPLICA	CAUSAS	CLASIFICACIÓN
**DOLOR:** La sensación de dolor es muy subjetiva y el paciente puede quejarse poco o mucho, aunque el trauma o el daño en la piel o tejido sea mínimo.	Para poder evitarlo o disminuirlo el laboratorio debe tener muy clara el protocolo de toma de muestras donde se indica que se le debe explicar muy claramente el procedimiento al paciente, verificar la buena calidad y estado de los elementos usados.	El dolor nunca debe subestimarse por su cronicidad y puede ser un motivo de consulta de urgencia	El laboratorio además debe contar con un proceso estandarizado, con personal suficientemente entrenado y hábil para realizar el procedimiento.	NO APLICA
**HEMORRAGIA O SANGRADO VAGINAL:** Es cualquier sangrado vaginal no relacionado con mestruación. Este tipo de sangrado puede incluir un manchado leve entre periodos. Puede proceder de cualquier sitio del tracto genital, incluyendo vulva, la vagina, el cuello uterino y el útero.	Capacitación en toma de muestras y retroalimentación constante al auxiliar de laboratorio o al personal hospitalario. Indagar correctamente al paciente, si es virgen o con sangrados previos	Se debe suspender la toma de muestra (puncion o toma de muestra especial). Presionar directamente sobre el sitio de punción con algodón seco. Solicitar cita mèdica recurriendo a colaborador o administrador designado si el caso lo amerita.	Se produce por falla en la técnica de la toma de la muestra de secreción vaginal	NO APLICA
**CORTADA:** Herida hecha con un cuchillo o un objeto cortante.	Utilizar los elementos de acuerdo las especificaiones del fabricante y a los implementos requeridos. (bisturi con su mango)	Suspender toma de muestra punción o toma de muestra especial. Presionar directamente sobre el sitio de la punción minimo tres segundos con algodón seco. Levantar la extremidad. Solicitar cita médica recurriendo a colaborador o administrador designado si el caso lo amerita	Toma de muestras de hongos con bisturí	NO APLICA

7. ROTULACION Y MARCACION DE MUESTRAS:

El software del Laboratorio emite de forma automática, en el momento del ingreso al aplicativo DataLab Enterprise una serie de rótulos autoadhesivos con código de barras, que incluye la información demográfica de: nombre del paciente, número de referencia asignado y el número y nombre del tipo de recipiente a tomar.

Cuando el usuario está en el módulo de toma de muestras, el auxiliar de Laboratorio alista el material de acuerdo con los exámenes solicitados por el médico, y marca de forma manual los tubos con los correctos establecidos institucionalmente para la marcación de muestra: nombres y apellidos completos, número de identificación y hora de la toma de muestra. Luego adhiere el rótulo (Sticker código de barras) a los tubos en forma vertical, verificando primero la correcta identificación del paciente. Dejando descubierto el nombre del paciente para poder realizar doble chequeo de marcación antes del procesamiento de la muestra pero también dejando ventana visible para verificar la calidad del suero

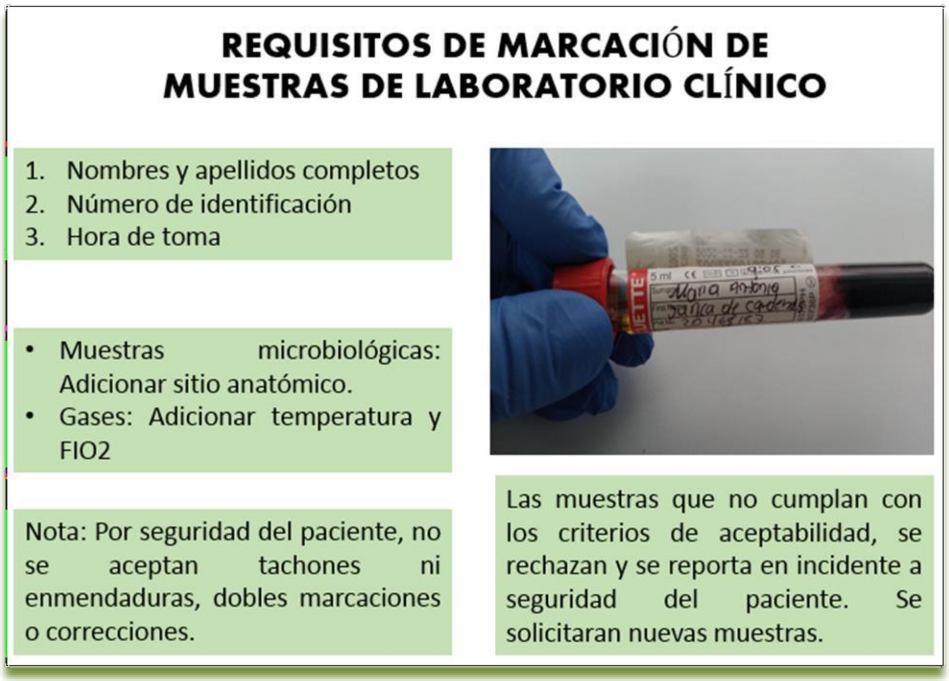

Estos criterios de marcación son de obligatorio cumplimiento por parte de todo el personal de la clínica que realice toma de muestras para laboratorio clínico. Estos criterios se estandarizan con el fín de minimizar el error, garantizando la seguridad del paciente y la calidad de los procesos. La identificación correcta de las muestras es liderada por el talento humano de la institución quienes tienen la función de garantizar que la identificación de la muestra sea una variable de cero errores. Cuando esto falla y no es posible tomar una nueva muestra, existe la posibilidad de llenar el **LC.01.MA.02.FT.03** Formato responsabilidad muestras con fallas en la rotulación, el cual compromete al médico y a la enfermera jefe encargada a certificar que la muestra si es del paciente al cual se le solicitaron los exámenes, y que asumen la responsabilidad en el caso de que la muestra no cumpla con algún criterio de aceptación para el examen solicitado.

Se define el uso de este formato **únicamente** para muestras de biopsia excisional y otras de procedimientos mayores que impliquen sedación o mayor riesgo para el paciente. Para los demás tipos de muestra, siempre se deberán rechazar, solicitar nueva muestra y generar reporte a seguridad del paciente.
Este formato debe ser diligenciado en su totalitdad y entregado en el servicio de laboratorio clínico para dar ingreso y procesamiento. En todos los casos se debe generar reporte a seguridad del paciente para realizar análisis y definir plan de mejora.

8. FICHA TÉCNICA PARA TOMA DE MUESTRAS

FICHA TECNICA PARA TOMA DE MUESTRAS
LABORATORIO CLINICO COMPENSAR
compensar | salud

**TIPO DE MUESTRA:** Sanguínea	**ORIGEN:** Venosa, arterial, capilar
**POBLACION:** Lactantes, niños y adultos	**ROL:** Auxiliar de Laboratorio o Bacteriologo
**MATERIALES**	Ø Guantes desechables
	Ø Torundas de algodón
	Ø Alcohol de 70º
	Ø Curitas (según procedimiento)
	Ø Tubos (De acuerdo al requerimiento de la orden médica)
	Ø Torniquete (según procedimiento)
	Ø Aguja y camisa (según procedimiento)
	Ø Visualizador de venas: en las sedes de alto y mediano volumen de pacientes se utiliza el equipo en pacientes de difícil acceso venoso
	Ø Jeringas, equipo alado (según procedimiento)
	Ø Jeringas heparinizadas con heparina de litio (según procedimiento)
	Ø Lancetas retractil para tecnología POCT.
	Ø Lanceta para prueba rápida (según procedimiento)
	Ø Colchoneta pediátrica (pacientes pediátricos)
	Ø Etiquetas identificativas o rótulos con los datos de identificación del paciente
**ANTES DE REALIZAR LA TOMA**	
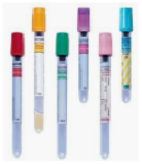	Aplicar los correctos de toma de muestra
	Verificar la identificación del paciente (preguntando al paciente competente o a su acompañante y confrontando la información recibida contra la orden médica), si existen discrepancias consultar el caso con administrador de la sede.
	Explicar a los padres y/o acudientes del paciente o al paciente si es mayor de edad competente, el procedimiento que se va a realizar.
	Alistar el material de acuerdo al requerimiento de los examenes solicitados en la orden, rotular los tubos de acuerdo a protocolo, mantener unicamente los elementos necesarios en el cubículo para la toma de muestras.
	Diligenciar documentación pertinente y/o consulta de instructivos. Indicar al paciente la fecha en que puede venir a reclamar los resultados.
	Realizar lavado de manos con agua y jabón cada hora, cuando las manos estén sucias, contaminadas con secreciones, cuando se pase de un área contaminada a un area limpia o cuando sea necesario; se debe realizar higienización de manos cuando se requiera según instrucciones establecidas en los 5 momentos de lavado de manos.
	Colocarse los guantes desechables y EPI.
	Indicar al paciente la posición que debe adoptar para la adecuada toma de muestra, si es paciente pediátrico ubicarlo en la colchoneta destindada para este fin solicitando la colaboaración de los padres o acudientes del menor.
**SELECCIÓN DEL SITIO DE PUNCIÓN**	
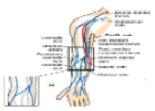 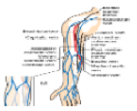	Solicitar al paciente que extienda el brazo y visualizar el sitio de punción.
	* Vena cubital * Vena cefalica * Vena basilica * Fosa antecubital * Antebrazo (vena radial) * Dorso de la mano
	El sitio ideal para realizar el procedimiento de venopunción es la fosa antecubital, que es el área de cualquiera de los brazos anterior (frente a) e inferior a la curva del codo, donde una serie de venas grandes se encuentran relativamente cerca de la superficie de la piel. Cuando las venas antecubitales no son de fácil venopuncion, es aceptable usar las venas ubicadas en el dorso de la mano (no ubicadas en la parte inferior de la muñeca). No se aconsejable usar sangre arterial si el requerimiento es sangre venosa.
	La vena a elección es: **La vena media y vena media cubital.** Como segunda y tercera opcion se pueden usar las venas cefalica y basilica. Estas pueden ubicarse en forma de H o M segun grafica anexa.
	Visualizar el sitio anatomico para realizar la venopunción.
	Idealmente el paciente debe mantener la mano cerrada constantemente ya que cuando se mantienen abiertas las manos se reduce la cantidad de presión venosa a medida que los musculos se relajan.
	Ubicar el torniquete con una presión adecuada, este se debe colocar sobre la superficie de la piel a unos 5 cm por encima de la punción con el fin de fijar la vena.
	El sitio de punción no se debe palpar luego de haber realizado proceso de desinfección según protocolo.
**DESINFECCION SITIO DE PUNCIÓN**	
	Se debe realizar con algodón impregnado al 70% realizando un movimiento de fricción del centro a la periferia (durante minimo 30 segundos), posterior a la realización de esta desinfeccion se debe dejar secar el acohol al aire libre.

	**USO DEL TORNIQUETE**
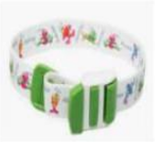	Los torniquetes se deben descartar cuando esten visiblemente contaminados con fluidos biológicos.
	Los torniquetes se deben segùn instructivo INS_PSS_168.
	El torniquete se debe colocar alrededor del brazo con una distancia de 7,5-10 cm sobre el sitio de la venopunción.
	El torniquete se debe retirar cuando se observe que la sangre fluye dentro del tubo, idealmente debe estar colocado sobre el brazo por un tiempo no superior a 10 segundos.
	**Torniquete Pronto**: En el caso del torniquete Pronto se debe cerrar y halar la cintilla para realizar presión con el torniquete, al obtener el retorno sanguíneo se debe desatar lentamente, pulsar y abrir para retirar del brazo. 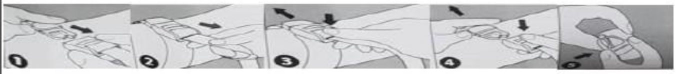
	**USO DE GUANTES**
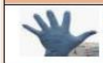 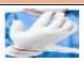	Los guantes se deben cambiar cada vez que se atienda un paciente.
	**USO TORUNDAS DE ALGODÓN**
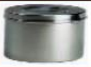 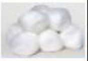	Se deben colocar en el sitio de la punción al retirar el sistema de extracción sanguínea ejerciendo una presión media sin que el paciente en ningún momento doble el brazo para reemplazar está presión. Las torundas de algodòn deben permanecer en las algodoneras.
	**USO DE BANDA PLASTICA ADHESIVA (CURAS REDONDAS)**
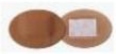	Se debe verificar que el flujo sanguineo haya cesado y proceder a colocar la banda plástica en el sitio de la venopunción de modo que la almohadilla de la cura quede sobre el sitio de la punción. Esta banda adhesiva debe ser retirada por el paciente 15 minutos después de colocada. No se deben usar en personas alergicas al latex.
	**NO OBTENCION DE MUESTRA TRAS PROCESO DE VENOPUNCION**
	Cambiar la posición de la aguja: Cuando la aguja ha penetrado lejos del interior de la vena, es necesario halar un poco hacia atrás la aguja, rotar la aguja para reubicarla lateralmente en intentar nuevamente extraer la muestra de la vena con un nuevo tubo que garantice el vacio. Este procedimiento puede generar dolor, hematoma e incluso perforación de arterias adyacentes por lo que solo se usa en casos de extrema necesidad. Este procedimiento no se debe realizar mas de dos veces, si esto ocurre se debe acudir a otra persona que tenga amplia experiencia en procesos de dificil venopunción.
	**CRITERIOS DE RECHAZO**
	Muestra insuficiente, muestra hemolizada, recolección en tubo inadecuado, muestra mal identificada, muestra coagulada en tubo que contenga anticoagulante (Tubo tapa azul, tapa lila, tapa verde) o en jeringa heparinizada.

9. TOMA DE MUESTRAS GASES ARTERIALES

Antes de la punción se recomienda la realización del test de Allen para evaluar la perfusión y saturación de la arteria. Pedir al paciente que apriete el puño durante 30 segundos, luego presionar con los dedos ambas arterias del antebrazo del paciente, la cubital y la radial. A continuación liberar la presión sobre la arteria cubital. Repetir la prueba liberando la presión sobre la arterua radial. El retorno capilar debe aparecer en 5 segundos. Se después de este tiempo la mano sigue palida, el resultado de la prueba es positivo e indica una alteración del suministro de sangre, en este caso no se debe puncionar la arteira de esa extremidad.

FICHA TÉCNICA PARA TOMA DE MUESTRAS LABORATORIO CLINICO COMPENSAR

compensar | salud

**TIPO DE MUESTRA:** Sanguínea	**ORIGEN:** Arterial
**POBLACION:** General	**ROL:** Auxiliar de Laboratorio y/o Enfermería, fisioterapeuta

**MATERIALES**	Ø Guantes desechables
	Ø Torundas de algodón
	Ø Alcohol de 70º
	Ø Curitas
	Ø Gasas
	Ø Jeringas heparinizadas con heparina de litio.
	Ø Etiquetas identificativas o rótulos con los datos de identificación del paciente

ANTES DE REALIZAR LA TOMA

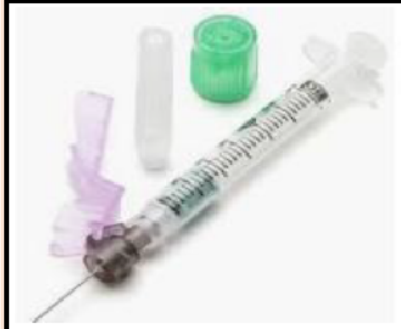

Aplicar instrucciones descritas en la ficha Generalidades Toma de muestras sanguínea.
Realizar lavado de manos con agua y jabón cada hora, cuando las manos estén sucias, contaminadas con secreciones, cuando se pase de un área contaminada a un área limpia o cuando sea necesario; se debe realizar higienización de manos cuando se requiera según instrucciones establecidas en los 5 momentos de lavado de manos.
Colocarse los guantes desechables y EPI.
Indicar al paciente la posición que debe adoptar para la adecuada toma de muestra.

SELECCIÓN DEL SITIO DE PUNCIÓN

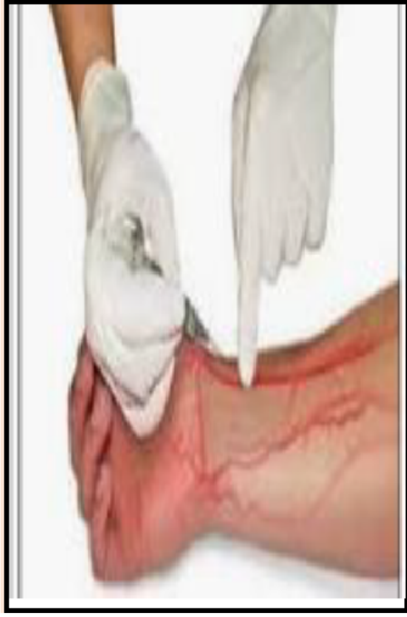

**1. Arteria Radial (Arteria de elecciòn ideal para la toma de muestra)**	Coloque el dedo indice y medio sobre la arteria radial a la altura de la muñeca presionando suavemente para sentir las pulsaciones en la arteria.
	Cerca del área de flexión de la muñeca, entre el apéndice estiloides del húmero y el tendón del flexor radial de la mano (preferiblemente la extremidad no dominante) Sujetar la arteria entre las yemas de los dedos.
**2. Arteria Femoral**	Por debajo del ligamento inguinal, generalmente en el pliegue inguinal (la arteria es lateral a la vena y medial al nervio). De está arteria **Únicamente se debe tomar esta muestra en ambiente hospitalario.**
**3. Arteria Braquial**	En el pliegue del codo (lugar no recomendado por riesgo de hematoma, que a su vez puede comprimir el nervio; se prefiere la extremidad no dominante). De está arteria **Únicamente se debe tomar esta muestra en ambiente hospitalario.**

Al identificar el sitio realice el procedimiento de asepsia de manera circular del centro a la periferia con alcohol isopropilico al 70%.

TÉCNICA DE EXTRACCION	
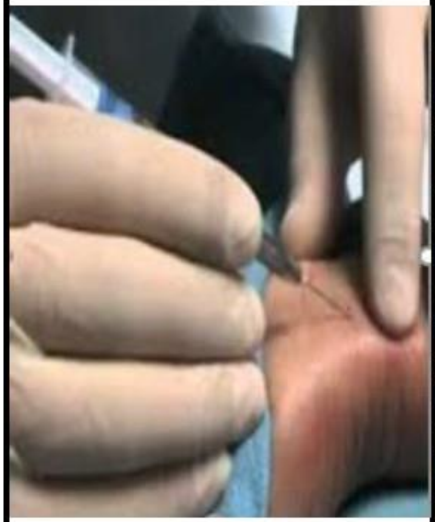	Se debe dejar secar el alcohol al aire.
	Insertar la aguja bajo el ángulo de 45°. Tras la aparición del flujo de sangre pulsátil en la jeringa, extraer 1 ml de sangre, tirando delicada y lentamente del émbolo de la jeringa se debe tener cuidado para no succionar aire a la jeringa.
	Después de extraer la sangre, en el mismo instante en que se retira la aguja, se coloca una torunda de algodón seco en el sitio de la punción realizando una presión constante, descartar la aguja según protocolo de bioseguridad, tapar la jeringa con el tapón de caucho, mezclar el contenido de la jeringa 5 veces por inversión, proceder a su procesamiento y/o transporte interno en la sede, (sedes que cuenten con analizador de gases arteriales y venosos), descartar los dispositivos usados según protocolo de bioseguridad. Para el caso de la jeringa, esta se debe descartar después del procesamiento de la muestra, retirar la torunda de algodón verificando que haya cesado el flujo de sangre, este procedimiento debe ser supervisado por el personal que toma la muestra, indicando al paciente que puede retirar la torunda cuando haya cesado el flujo sanguíneo.

10. FICHA DE INFORMACIÓN DE SERVICIOS DE LABORATORIO CLÍNICO

Laboratorio Clínico Compensar cuenta con dos fichas de información, la ficha de exámenes de procesamietno propio y a de contrareferencia. Son para consukta libre por parte del personal de compensar desde la intranet. En estas fichas se encuentra la información relacionada a las condiciones del paciente, tipos de muestras, tubos requeridos, condiciones de almacenamiento y estabilidad y tiempo de reporte. Siempre que se requiera, personal de laboratorio consultará esta información para brindarla a los pacientes o a enfermería.

11. NORMAS GENERALES ESTABLECIDAS PARA CADA TIPO DE ESPÉCIMEN:

Los especímenes deben ser recibidos por el personal del laboratorio en el menor tiempo posible una vez extraídos o recogidos:

- Sangre: Durante su transporte, debe evitarse la agitación (por la posible hemólisis) y se deben proteger de la exposición directa a la luz (debido a la degradación de algunos constituyentes, como la bilirrubina). Para la determinación de algunos parámetros inestables (lactato, amonio, renina plasmática, fosfatasa ácida) los especímenes deben mantenerse refrigerados de 2 a 8°C, inmediatamente después de la toma, y deben transportarse en refrigeración. Los tubos de sangre deben estar en posición vertical durante su transporte, con el tapón hacia arriba, lo que favorece la formación completa del coágulo y reduce la agitación del contenido del tubo.

- Secreciones y Líquidos corporales: Recolectar las muestras en frascos o tubos con

anticoagulante para recuento celular, exceptuando para LCR, para el cual se utiliza tubo de vidrio estéril, sin aditamentos para cultivos y examen bioquímico. No deben refrigerarse o congelarse antes de cultivar.

- Orina: los especímenes para análisis de orina se recogen y transportan en contenedores de plástico estériles y desechables. La orina de pacientes pediátricos se recoge en bolsas flexibles de polietileno, que pueden sellarse para el transporte.
- Heces: se recolectan en los recipientes especiales para muestras coprológicas.

Nota:

- Es importante tener en cuenta que las muestras sanguíneas, de orina y materia fecal deben transportarse lo más pronto posible (antes de 90 minutos, teniendo en cuenta, las recomendaciones para cada una de las pruebas de acuerdo a insertos de pruebas).
- Para muestras de gases arteriales y venosos deben transportarse al laboratorio antes de 15 minutos.
- Para las muestras de microbiología se recomienda el transporte en los primeros quince minutos y máximo dos horas.

12. CRITERIOS DE SOLICITUD DE NUEVAS MUESTRAS/INATENCION DE PACIENTES

El establecimiento de criterios de aceptación-rechazo de los especímenes o las muestras que llegan al laboratorio, debe ser una de las medidas a tomar para el establecimiento de un sistema de calidad adecuado para garantizar la seguridad del paciente.

El manejo definido es no procesar si puede obtenerse nueva muestra o procesar registrando en el momento de validar el resultado, un comentario sobre la calidad de la muestra en caso de ser un paciente con capacidades mentales especiales, con algún grado de discapacidad o de difícil acceso venoso. En todos los casos se tratará de facilitar el proceso para que el paciente pueda acceder al servicio.

Los pacientes que no cumplan con los requerimientos por prueba no son aptos para la toma de muestras ya que ello afecta directamente el resultado de los exámenes:

- **Ayuno:** el paciente debe haber realizado su última comida entre 8-10 horas antes, después de ello no haber ingerido nada incluyendo chicle o goma de mascar, cigarrillo, agua, tinto
- **Dieta:** Cuando la prueba así lo requiera el paciente debe abstenerse de consumir ciertos alimentos a practicar actividades que influyen directamente en la medición del analito.
- **Medicamentos:** En caso de realizar control a los niveles de medicamentos el paciente NO debe haber tomado/aplicado los mismos, lo hará después de la toma de la muestra
- **Recipiente inadecuado:** Las muestras de materia fecal, orina aislada o de 24 horas se recolectarán en los recipientes proporcionados por el Laboratorio para tal fin o pueden ser adquiridos en droguería. Por ningún motivo se recibirán muestras en envases de productos lácteos, conservas o similares

Cuando definitivamente no sea posible que el paciente sea atendido por presentar las causas anteriormente mencionadas, se le explicara la situación y se le dejara claro que puede presentarse en el laboratorio en los siguientes días hábiles (sin superar un mes) en el horario habitual. Los pacientes deben ser redireccionandos a acceso donde registraran el indicador de inatenciones y

aclarran el tema de facturación.
Además de las causas de inatención ya mencionadas se tendrán en cuenta las siguientes para los pacientes de los servicios de internación:

- **Muestras sin orden médica:** Las muestras que son de difícil obtención, rápido deterioro o que de su resultado depende la toma rápida de decisiones serán procesadas sin orden (gases, troponina, hematocrito, hemoglobina). La Clínica se comprometerá a cumplir con los requisitos administrativos posteriormente. Las muestras que no sean consideradas de urgencia vital deben estar acompañadas siempre de la orden médica.

- **Utilización de tubo o insumo inadecuado**: Dependiendo del tipo de prueba la muestra debe tomarse en el recipiente indicado.

- **Volumen de muestra incorrecto:** Este criterio de rechazo de muestras es crítico en los análisis que requieren de muestras tomadas en tubos con anticoagulantes como la determinación de las pruebas de coagulación o en la velocidad de sedimentación globular y cuadros hemáticos, ya que se debe mantener la proporción exacta entre el volumen de muestra y el de anticoagulante. Los tubos de llenado por vacío están preparados para que el volumen de muestra que entre sea el correcto. Cuando se llenan los tubos con jeringa debe tenerse precaución para las determinaciones que exigen que esta proporción sea exacta.

- **Hemólisis:** Se produce por diferentes motivos que deben ser evitados: Venopunción difícil, manejo incorrecto del espécimen obtenido, consecuencia de una enfermedad que produzca destrucción in vivo de los eritrocitos. El grado de interferencia de la hemólisis depende de la intensidad de la misma, de la concentración real del analito y de la metodología empleada. Laboratorio Clinico Compensar cuenta con un reactivo que mide el índice de hemolisis presente en la muestra y dependiendo el analito a procesar se acepatará o rechazará la muestra teniendo en cuenta el resultado del índice de hemólisis y el grado de interferencia en el analito.

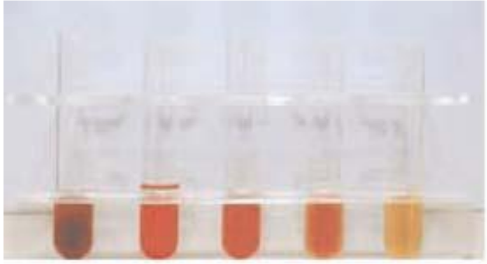

Imagen 12: muestras con diferentes grados de hemólisis.

- **Muestra lipémica:** Es aquella muestra de plasma o suero con alto contenido en grasa. Presenta un aspecto blanquecino, y puede deberse a la extracción de una muestra de un paciente con alimentación parenteral o tras una ingesta copiosa. Hay determinaciones cuyos resultados se alteran cuando existe esta condición.

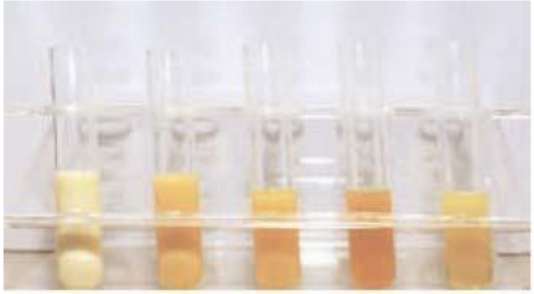

Imagen 15: muestras con diferentes grados de lipemia.

- **Muestra coagulada:** Aquella muestra que se presenta coagulada parcial o totalmente y que se extrajo con anticoagulante en el tubo. La coagulación puede deberse a una extracción lenta, a una mezcla incorrecta del anticoagulante con la muestra o a un defecto del propio anticoagulante.

- **Muestra mal identificada:** Las muestras deben identificarse con: Nombres y Apellidos completos, número de identificación y hora de la toma en letra legible sin tachones ni enmendaduras. El incumplimiento de estos criterios genera rechazo de las muestras.

- **Temperatura de transporte inadecuada**: Hay determinaciones que sólo se pueden realizar bajo estrictas condiciones pre analíticas de conservación de temperatura. Por ejemplo, ácido láctico, gases, amonio u homocisteína, exigen el transporte en refrigeración del espécimen, mientras que las crio globulinas exigen que este transporte procure mantener la temperatura corporal. En la mayoría de analitos la temperatura es una variable continua y que por tanto no afecta de una forma brusca, sino que disminuye gradualmente la calidad de la muestra en cuanto más se aleje de la temperatura óptima de transporte o de conservación.

- **Muestras con coagulo retraído:** las muestras deben ser enviadas al laboratorio en el menor tiempo posible una vez tomadas, para evitar este fenómeno, el cual se evidencia por la separación de eritrocitos del suero, esto podría afectar las determinaciones por la degradación de la muestra.

- **Muestra insuficiente:** Aquella muestra a la que no se le pueden realizar todas las determinaciones solicitadas al agotar el espécimen.

- **Muestra deteriorada en Laboratorio:** Aquella muestra que viniendo correctamente se deteriora en el proceso de preparación: fragmentación del tubo en la centrífuga, derrame accidental de la muestra o caída del tubo.

Las muestras que tengan causal de rechazo no se procesarán, serán retenidas en el laboratorio y se llamara al respectivo servicio para notificar de tal circunstancia.

Las solicitudes de nuevas muestras por estas causas generan incidentes que deben ser reportados a seguridad del paciente por el sistema curuba. Ademas, se deben registrar en la matriz definida para tal fin con el objetivo de registrar el indicador mensual.

13. GENERALIDADES POR TIPO DE MUESTRA

13.1 TOMA DE MUESTRAS PARA GLICEMIA PRE Y POST CARGA DE GLUCOSA - CURVA DE TOLERANCIA A LA GLUCOSA - TEST DEO´SULLIVAN.

Exámenes tomados en muestra de sangre, utilizados para el posible diagnóstico de diabetes mellitus, estados hipo glicémicos o diabetes gestacional.

- El paciente debe presentarse en ayunas antes de las 9:00 a.m. y disponer de tiempo para la realización del examen.
- Tomar muestra en ayunas, marcar el tubo con el sticker correspondiente a la basal.
- Informar al paciente los riesgos asociados a la venopunción y la ingesta de la carga de glucosa, posteriormente solicitar el diligenciamiento y firma de los consentimientos relacionados.
- Realizar glucometria: se coloca en la tira de glucometría una gota de sangre periférica la cual es introducida en el glucómetro hasta que el dato del valor de glicemia sea mostrado en la pantalla. (ver manual del usuario manejo del glucómetro dependiendo de la casa comercial utilizada.)
- Si la glucometria es inferior a 130 mg/dl dar la carga de glucosa según el examen ordenado y tipo de paciente. Si la glucometria es mayor a 130 mg/dl indicar al paciente tomar desayuno normal y avisar a la Bacterióloga de Química para hacer la anotación correspondiente en la validación del resultado.
- Entregar por escrito el horario de toma de las muestras siguientes al paciente e indicarle que no puede ingerir ninguna bebida o alimento, no fumar, no realizar ningún tipo de actividad física, no masticar chicle y permanecer en la sala de espera del laboratorio.

Para administrar la carga de glucosa seguir las siguientes recomendaciones:

LABORATORIO CLINICO COMPENSAR compensar

ADMINISTRACION CARGAS DE GLUCOSA

SOLICITUD	CARGA	GLUCOMETRIA	MUESTRA BASAL	MUESTRA POST 2 HORAS	MUESTRA 1/2 HORA	MUESTRA 1 HORA	MUESTRA 2 HORAS	MUESTRA 3 HORAS	MUESTRA 4 HORAS	MUESTRA 5 HORAS
Pre y post prandial	NA, Desayuno habitual	NA	X	X						
Pre y post carga	75 gr en 200 mL de Agua	X	X	X						
Curva de glicemia	75 gr en 200 mL de Agua	X	X		X	X	X	X		
Curva de glicemia 4 horas	75 gr en 200 mL de Agua	X	X		X	X	X	X	X	
Curva de glicemia 5 horas	75 gr en 200 mL de Agua	X	X		X	X	X	X	X	X

PROTOCOLO PREPARACION DE CARGAS DE GLUCOSA			
POBLACIÓN	CANTIDAD DE GLUCOSA EN CC	CANTIDAD DE AGUA EN CC	VOLUMEN FINAL
GESTANTES 75gr DE GLUCOSA CURVA DE 2 HORAS 3 MUESTRAS	150	150	300
ADULTOS NO GESTANTES 75gr DE GLUCOSA	150	150	300

PROTOCOLO PREPARACION DE CARGAS DE GLUCOSA			
Equivalencias en mililitros de glucosa para pacientes con curvas de glicemia; pre y post cargas de glucosa con peso inferior a 43 KG			
PESO EN KILOGRAMOS (Kg)	CANTIDAD DE GLUCOSA EN CC	CANTIDAD DE AGUA EN CC	VOLUMEN FINAL
10	35	35	70
11	38,5	38,5	77
12	42	42	84
13	45,5	45,5	91
14	49	49	98
15	52,5	52,5	105
16	56	56	112
17	59,5	59,5	119
18	63	63	126
19	66,5	66,5	133
20	70	70	140
21	73,5	73,5	147
22	77	77	154
23	80,5	80,5	161
24	84	84	168
25	87,5	87,5	175
26	91	91	182
27	94,5	94,5	189
28	98	98	196
29	101,5	101,5	203
30	105	105	210
31	108,5	108,5	217
32	112	112	224
33	115,5	115,5	231
34	119	119	238
35	122,5	122,5	245
36	126	126	252
37	129,5	129,5	259
38	133	133	266
39	136,5	136,5	273
40	140	140	280
41	143,5	143,5	287
42	147	147	294
43	150,5	150,5	301

Para el uso de la carga de glucosa como medicamento se deben tener en cuenta los siguientes correctos relacionados a continuación:

13.2 TOMA DE MUESTRA PARA D-XILOSA:

Para la toma de muestra del examen D- Xilosa tener en cuenta las siguientes recomendaciones: En el momento que el paciente solicite información sobre el examen y teniendo en cuenta si niño y/o menor de 18 años tomar los datos de: Identificación, edad, peso, talla; enviar los datos por correo electrónico de la bacterióloga encargada de remisiones en sede CPL ó Salitre y/o comunicarse al teléfono 4285088 ext. 11515,11502, para que el laboratorio de referencia envíe la carga requerida para el paciente, que estará disponible para el día que el paciente venga a tomarse su examen.
Las muestras se toman únicamente de lunes a viernes y debe presentarse en el laboratorio a las 6:30 am en el laboratorio. Disponer de 1.5 horas si es menor de edad o de 5 horas si es adulto.
Tener ayuno no mayor a 12 horas y que su última comida la haya hecho antes de las 11:00 pm del día anterior.
Dos (2) días anteriores a la toma del examen, debe realizar una dieta libre de: gelatinas, frutas, jaleas, postres.
Los pacientes menores de edad deben traer el día de la toma del examen 2 onzas de agua medida en el recipiente en el que el niño tome sus líquidos habitualmente (tetero, vaso, vaso - pitillo), para diluir allí la carga e indicar su toma completa dentro de máximo 20 minutos.
Tomar una muestra de sangre 1 hora después de la ingesta de la carga de xilosa.

A los pacientes mayores de 18 años se administra una carga 5 gramos de xilosa en 500 ml de agua, el paciente debe recolectar la orina en el intervalo de cinco horas contando inmediatamente después de ingerir la carga, la recolección se hace en un recipiente plástico limpio de un (1) litro suministrado por el laboratorio.

13.3 LÍQUIDOS BIOLÓGICOS (Ver manual de microbiología)

- **Líquido cefalorraquídeo:** El diagnóstico y tratamiento correcto de una enfermedad del SNC puede depender de los resultados del examen del LCR en el laboratorio; debido a esto, este líquido tiene que extraerse y manipularse correctamente.

El LCR es normalmente estéril y puede obtenerse mediante punción lumbar o, con menos frecuencia, por punción cisternal, cervical o ventricular; cada uno de estos procedimientos debe ser realizado asépticamente por un médico con experiencia en el mismo y alertado el paciente sobre sus indicaciones y posibles complicaciones. Se debe marcar y desinfectar la zona de punción. La cantidad que se recoja dependerá de la situación clínica, cuando se están buscando células tumorales, es importante obtener tanto líquido cefalorraquídeo como se pueda.

Con el fin de evitar la contaminación de la muestra, se debe obtener y transportar el líquido cefalorraquídeo en tubos cerrados. El líquido cefalorraquídeo deberá distribuirse, en condiciones asépticas, en varios tubos transparentes con tapa rosca (estériles y sin aditivos). Es muy importante después de tomada la muestra, enviarla al laboratorio lo antes posible para evitar deterioro celular.

En ocasiones es útil la recolección de muestras en diferentes porciones indicando la secuencia

de llenado ya que facilita la determinación del origen de los posibles hematíes.

No es aconsejable el uso de guantes empolvados con talco cuando se está extrayendo líquido cefalorraquídeo, ya que podría alterar el examen citológico del líquido cefalorraquídeo.

- **Líquidos serosos:** Líquidos pleural, pericárdico y peritoneal: Los líquidos serosos son líquidos corporales que derivan del plasma y se encuentran en la cavidad pleural, pericárdica y peritoneal. Los líquidos serosos son ultrafiltrado del plasma que derivan de la abundante red capilar de la membrana serosa.

El líquido de la cavidad peritoneal se denomina normalmente líquido ascítico.
Para los estudios de bioquímica el líquido debe recogerse sobre un recipiente estéril y tapón de rosca, para los estudios de citología se procederá igual pero utilizando el tubo con EDTA como anticoagulante, si ordenan Adenosin de aminasa (ADA) se debe tomar tubo. Heparina de sodio (Tapa verde)

- **Líquido sinovial:** Los trastornos de la membrana sinovial, la alteración en los elementos de sostén articular y la presencia de cuerpos extraños pueden producir la acumulación de grandes cantidades de líquido sinovial en las articulaciones. Su posterior análisis en el laboratorio puede ser decisivo para el diagnóstico de la patología subyacente. La obtención del líquido debe realizarse con una jeringa sin anticoagulante. Una vez recogida la muestra, en función del volumen obtenido, debe distribuirse en los diferentes recipientes necesarios para realizar su estudio: Tubo estéril para examen microbiológico, Tubo sin aditivos para el estudio de cristales y bioquímica, Tubo heparinizado o con EDTA para el recuento celular.
- **Líquido seminal:** Las instrucciones sobre la toma de muestra y las condiciones pre analíticas a seguir serán facilitadas al paciente tanto por el médico que solicita el análisis como por el laboratorio: Período de abstinencia: Antes de la recogida de la muestra de semen a analizar, debe guardarse abstinencia sexual durante un periodo entre 3 y 5 días (y no más de 7 días), lo que implica no tener ninguna pérdida de semen por coito, masturbación, polución nocturna o cualquier otra circunstancia durante estos días. Si el período de abstinencia es inferior a 48 horas, la muestra se debe considerar como no válida para su estudio. Medidas higiénicas: Es importante evitar una posible contaminación de la muestra. Lavarse el pene con jabón y aclararse abundantemente con agua para evitar restos de jabón. No se debe aplicar ningún tipo de crema. Recoger la muestra sobre un frasco de plástico de boca ancha estéril, cerrándolo con su tapa tras la obtención del semen (asegurarse de que queda bien cerrado). Evitar los cambios de temperatura que se producen en el transporte.

Obtención de la muestra: La muestra debe obtenerse por masturbación ó el uso de un colector especial que no contiene lubricantes ni espermicidas utilizado durante el coito para el caso de espermogramas. Para cultivos la muestra debe recogerse directamente en un frasco estéril (recipientes para muestras de orina). Los preservativos comunes no pueden usarse debido a que contienen lubricantes y espermicidas y el “coitus interruptus” es inaceptable debido a que la primera fracción, rica en espermatozoides, se puede perder fácilmente.

Es importante recoger el contenido total de la eyaculación. En caso de que se pierda o se vierta alguna cantidad, por pequeña que sea, el paciente deberá comunicarlo al personal de laboratorio ya que para el estudio de fertilidad la muestra no sería válida.

etc.). También es importante conocer si ha habido cambios clínicos significativos en el paciente (fiebre, toma de drogas, etc.) en los días o semanas previos.

Recolección de muestra para Espermograma: Para recolección de esta muestra, se dispone de un consultorio

- Se recomienda tener una abstinencia sexual de 3 a 5 días, que nunca debe ser menor de 2 días ni mayor a 7
- Previamente a la obtención de la muestra el paciente debe realizarse un buen lavado de las manos y de la región genital con agua y con jabón; enjuagarse bien y secarse con una toalla limpia, deberá orinar antes de recolectar la muestra de semen
- Para la recolección de la muestra de debe utilizar un colector especial estéril el cual permite la preservación de la muestra en condiciones adecuadas para su procesamiento.
- Si ha presentado fiebre o se sometió a una cirugía reciente debe aplazar el examen aproximadamente 30 días.
- Recoger la muestra utilizando el colector mediante coito usando el colector especial evitando que se derrame
- Sellar el colector cuidadosamente, y colocarlo dentro de otro recipiente o una bolsa plástica.
- Anotar la hora de recolección
- Transportar la muestra procurando mantenerla a una temperatura de 35 a 37°C (Temperatura corporal).
- Hacer llegar la muestra al laboratorio lo más pronto posible

Nota: La muestra conservada entre 35 a 37°C se puede mantener en buenas condiciones hasta una hora después de la recolección.

- **Orina**: Las muestras de orina son utilizadas por el laboratorio para diagnosticar y controlar el tratamiento de las enfermedades del riñón o del tracto urinario y en la detección de enfermedades metabólicas o sistémicas. Los métodos y horarios de recogida de las muestras dependen de las pruebas solicitadas por el médico.

Toma de muestra orina micción aislada: Se prefiere la orina de 1ª hora de la mañana ya que presenta una mayor osmolaridad, lo que refleja la capacidad que presenta el riñón para concentrar la orina. En esta primera orina se encuentran más concentrados elementos como leucocitos, bacterias, cilindros, hematíes, optimizándose así el rendimiento diagnóstico de las pruebas de laboratorio. Las orinas de micción aislada obtenidas de forma aleatoria se aceptan en situaciones especiales como analíticas urgentes, o determinados estudios, como por ejemplo, en el estudio del metabolismo óseo, que se recomienda la segunda orina de la mañana. Se debe recoger la orina de la porción media de la micción, ya que está menos contaminada por las bacterias del meato urinario que son arrastradas por la primera parte de la micción. Se recomienda lavado previo de genitales externos con jabón y abundante agua, evitar que la orina se contamine con jabón debido a que pueden verse afectados determinados parámetros como pH, o incluso el crecimiento bacteriano puede verse inhibido.

- **Técnica para niños:** En niños y niñas más pequeños (que no controlen esfínteres todavía), la orina se recogerá en colectores o bolsas estériles especialmente diseñadas para ellos de la siguiente forma: Lavado cuidadoso de los genitales y área perineal igual que en los adultos. Colocar la bolsa de plástico o el colector estéril. Retirar la bolsa en cuanto el niño haya orinado. Cada 20 minutos debe cambiarse la bolsa y reiniciar el proceso. Se recomienda obtener un volumen mínimo de orina de 8-12 ml para el análisis de micro químico y sedimento. Se pueden aceptar volúmenes menores en muestras procedentes de niños o pacientes oligo-anúricos.
- **Técnica para adultos:** La muestra ideal es la primera de la mañana, sin embargo, pacientes que ingresen por urgencias se recolectará la muestra en el momento de la solicitud. En todos los casos, se debe realizar un aseo genital con agua y jabon previamente. Se desecha la primera porción de la orina directamente en el sanitario y se recolecta posteriormente la parte media de la micción. No se debe tener contato directo de las manos con el interior del recipiente.
- **Pacientes discapacitados o con vejiga neurogenica**: El cuidador es quien debe tomar la muestra, Hacer asepsia del cistoflo, dejar salir un poco de orina y luego dejar caer la orina en el frasco estéril.

Para cultivo bacteriano se necesita un volumen mínimo de orina (1-10 ml de orina).

➢ **RECOLECCION ORINA DE 24 HORAS:** Para esta recolección se debe disponer de un día. El paciente debe contar con un recipiente plástico nuevo o en su defecto un recipiente que haya contenido agua con capacidad mayor a 1000 c/c.
  - El paciente no debe cambiar su rutina diaria y no ingerir más líquidos de lo normal.
  - Para iniciar la recolección debe levantarse a las 6:00 am y descartar la primera orina de la mañana en el sanitario; luego debe recoger el en recipiente plástico toda la producción de orina desde ese momento, incluyendo la de las 6:00 am del día siguiente para completar la orina de 24 horas. No debe descartar ninguna muestra
  - Para mujeres: No se debe recolectar durante el periodo menstrual.
  - Si el examen solicitado requiere muestra de sangre, debe tomarse el mismo día en el que se entrega la muestra de orina de 24 horas.
  - No se aceptan recipientes plásticos que hayan contenido aceites, detergentes, gaseosas, etc.
  - Es necesario medir el volumen de la orina recolectada, anotar el volumen en el recipiente adecuado, guardar una contra muestra en caso de que la muestra sea remitida a CPL o a un laboratorio inscrito.
  - Para pacientes de internación o urgencias, se puede iniciar la recolección de la muestra a cualquier hora del día. Siempre tener registro de la hora de inicio la cual debe ser posterior a desocupar vegija y calcular las 24 horas para su finalización.

13.4 TOMA DE MUESTRAS BACTERIOLOGICAS

Mediante el estudio bacteriológico se puede llegar a establecer un diagnóstico etimológico en un determinado proceso infeccioso de origen bacteriano.
Cuando el Laboratorio clínico toma muestras bacteriológicas para analizar, puede en general realizar examen directo, cultivos y antibiogramas.
Tener en cuenta las causas que puedan generar incongruencia en los resultados, por ejemplo, contaminación en el proceso de toma de muestras, muestra escasa, fallas en la preparación del

paciente, toma de muestras en sitios ajenos al problema, desconocimiento de información de interés para la orientación del diagnóstico, paciente bajo antibiótico-terapia, transporte inadecuado de la muestra, demoras en el proceso de especímenes.

Se deben cumplir con las Normas de Bioseguridad y el uso de elementos de protección personal.

- **MUESTRAS VAGINALES**

  - Toma de muestras en consulta externa a cargo de auxilair de laboraotrio. En urgencias e internación a cargo de enfermería.
  - Realizar al paciente la verificación de lectura y entendimiento de Riesgos en la toma de muestras Especiales. Proceder a solicitar firma de consentimiento informado.
  - Registrar toda la información relacionada en comentarios en Datalab Enterprise

  Preguntar a la paciente sobre: Medicamentos que esté tomando, óvulos o antisépticos vaginales que se esté aplicando, no haberse aplicado cremas ó duchas vaginales tres días antes.
  Confirmar edad y fecha de última menstruación, no asistir si está menstruando (presentarse cinco días antes o cinco días después del período).
  No haber tenido relaciones sexuales tres días antes de la toma de la muestra.

  - Orientar a la paciente hacia el baño o cubículo para que se retire la ropa de la cintura hacia abajo y se coloque la bata desechable o de tela destinada para tal fin.
  - Se realiza cambio de sabana a la camilla, y se coloca campo desechable en la parte inferior de la misma para ubicar a la paciente.
  - Indicar a la paciente acostarse en la camilla boca arriba en posición ginecológica con la espalda apoyada adecuadamente en la camilla y los pies en los estribos apoyados firmemente con el fin de prevenir caídas.

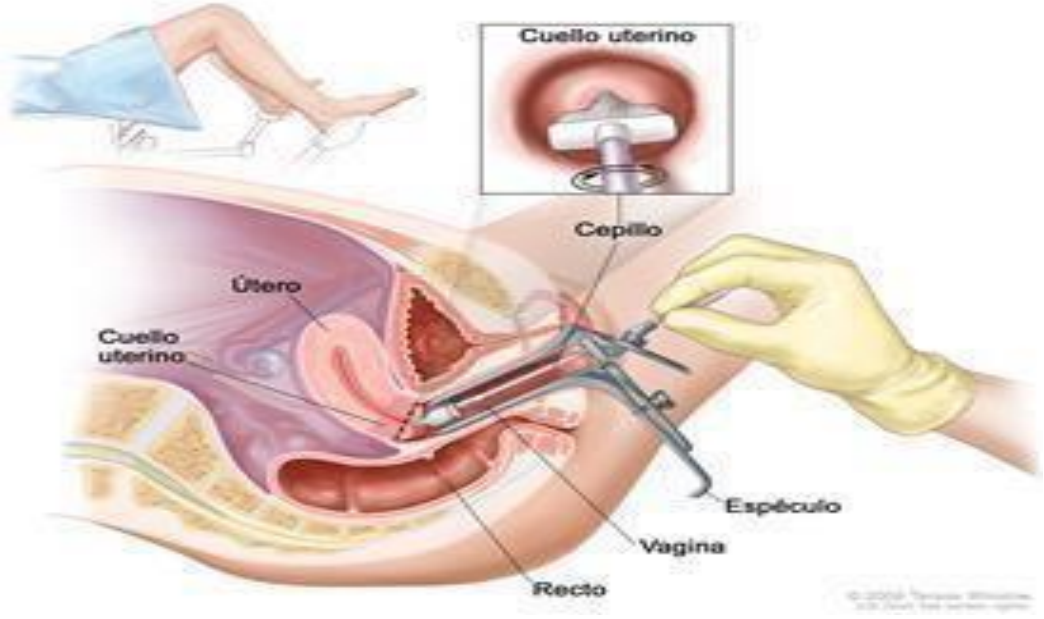

  - Si es una niña explicar al acompañante el procedimiento, en éste caso, ó pacientes vírgenes ó en embarazo, solo utilizar escobillón.
  - No utilizar lubricantes que facilitan la introducción del espéculo.
  - Tomar inicialmente muestra de endocervix y depositarla en tubo esteril para el transporte al

laboratorio.

- Con un segundo hisopo hacer frotis y extendidos para coloración de Gram, las láminas deben estar previamente marcadas con el número de referencia, iniciales del paciente y examen a realizar, la muestra de endocervix se ubicará contiguo al esmeril de la lámina y la muestra de exocérvix a su derecha (final de la lámina)
- Posterior a la toma de muestra vaginal, se retira el campo de material desechable, se deposita en la caneca roja, de igual forma se retira la sabana depositándola en la caneca de material sucio que se encuentra dentro del consultorio.
- Se hace desinfección de la camilla utilizando guantes industriales: con una toalla de papel desechable y jabón en uso en forma de barrido, se retira excedente con otra toalla de papel y agua y se desinfecta con alcohol antiséptico. Este procedimiento se hace entre paciente y paciente.
- Realizar lavado de manos y cambiar los guantes desechables por cada paciente.

- **FROTIS DE SECRECIÓN BALANO PREPUCIAL O LESION GENITAL**

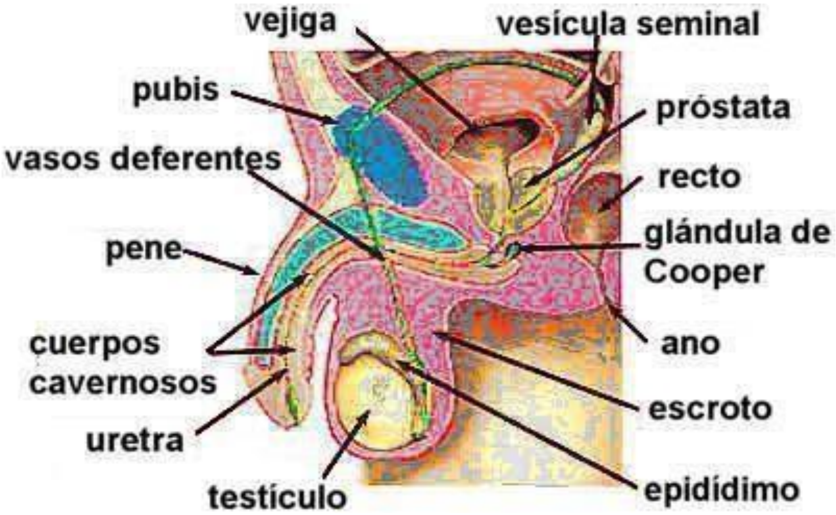

Verificar con el paciente que no esté utilizando antibióticos ni tratamientos tópicos, no debe haber realizado aseo genital, ni haber tenido relaciones sexuales 2 días antes para optimizar la cantidad y calidad de la muestra a tomar.

Se solicita al paciente que haga retracción del prepucio y con dos escobillones estériles se toma la muestra de las lesiones y-o secreciones que se puedan observar.
Si tiene orden de cultivo tomar un hisopo estéril.

- Con un segundo hisopo hacer frotis y extendidos para coloración de Gram, las láminas deben estar previamente marcadas con el número de referencia, iniciales del paciente y examen a realizar.
- Los hisopos se colocan en tubo esteril para su transporte al laboratorio.

- **FROTIS DE SECRECIÓN URETRAL**

- El paciente debe asistir al Laboratorio con retención urinaria mínima de 2 horas y

abstinencia sexual de 2 días.

- Se solicita al paciente que haga retracción del prepucio y presione para que la secreción fluya libremente; si no hay secreción introducir un hisopo estéril en el canal uretral y tomar la muestra. Depositar en tubo esteril para transporte al laboratorio.
- Con un segundo hisopo hacer frotis y extendido para coloración de Gram, las láminas deben estar previamente marcadas con el número de referencia, iniciales del paciente y examen a realizar.

- **LIQUIDO PROSTÁTICO**

La muestra es tomada por el médico Urólogo que ordena el examen.

- Para la muestra se debe realizar un masaje prostático, el paciente debe haber eliminado la orina mínimo 2 horas antes del examen y haberse realizado lavado previo con agua y jabón.
- Descripción del procedimiento para realizar Masaje prostático: Se coloca el paciente en posición de gateo sobre la camilla, se realiza introduciendo uno o dos dedos, enfundados en un guante de látex y lubricados con aceite mineral, por el ano, ubicando la próstata que es un tejido de contextura lisa y blanda y se inicia el masaje. No es necesaria una presión excesiva. El procedimiento tiene que realizarse frotando suavemente al principio con el dedo índice, masajeando los laterales de los lóbulos de la próstata, teniendo cuidado de no presionar demasiado vigorosamente sobre los nervios del centro.
- Se debe obtener un líquido claro y transparente, en casos de prostatitis se observa turbio. El líquido debe estar libre de secreción seminal para ser una muestra valida. Depositar en tubo esteril para ser transportada al laboratorio.

- **SECRECIÓN FARÍNGEA:**

- Se utiliza para el diagnóstico de faringitis estreptocóccica y otro tipo de patógenos.
- Preguntar al paciente si está tomando algún tipo de antibiótico, si ha ingerido algún alimento o se ha realizado enjuague bucal.
- Pedir al paciente que abra la boca y con la ayuda del baja lenguas mantener la lengua hacia abajo, con hisopo estéril frotar las criptas amigdalinas y/o la faringe posterior los sitios con secreción, membranas o inflamación. En lo posible no tocar la mucosa oral, lengua, úvula ni dientes.
- Depositar en tubo esteril para ser transportada al laboratorio.

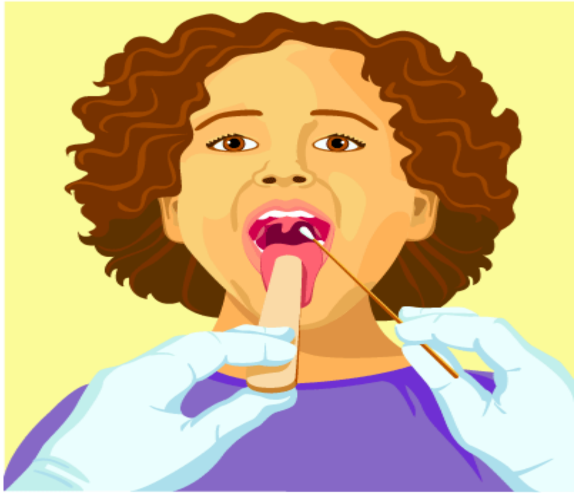

- **HISOPADO FARINGEO PARA STREPTO A TEST.**

  - La muestra es tomada de hisopado faríngeo en pacientes con sintomatología de fiebre, irritación en la garganta y faringitis a repetición.
  - La auxiliar debe tomar la muestra con un escobillón (provisto por la casa comercial) de las áreas más irritadas o exudativas que se observen, evitar la contaminación con flora oral, ayudados por un baja lenguas si es necesario.
  - Poner el escobillón en un tubo estéril con los reactivos provistos por la casa comercial (buffer A y buffer B en cantidades iguales) marcado correctamente con el número de referencia del paciente y tapado.
  - Entregar a la bacterióloga de la Unidad de Inmunología.

13.5 SECRECIONES Y HERIDAS:

Para las diferentes muestras de cultivo a tomar, verificar si está tomando antibióticos se debe registrar la información relevante en los comentarios en Datalab Enterprise

- **SECRECIÓN OJO:**

  - Tomar la muestra con un hisopo estéril de la parte interna de los párpados o de la secreción que se encuentre en el borde interno de los párpados, no tocar la piel adyacente para evitar la contaminación de la muestra.
  - Recolectar la muestra con escobillon esteril y transportar al laboatorio en tubo de vidrio esteril.

- **SECRECIÓN OIDO:**

  - Tomar la muestra con un hisopo estéril de la secreción presente en el oído evitando la contaminación con la zona aledaña a la lesión.
  - Si la secreción está en el oído medio o interno es el médico Otorrino quien debe tomar la muestra, se debe alistar el material para la toma por parte del medico.
  - Recolectar la muestra con escobillon esteril y transportar al laboatorio en tubo de vidrio

esteril.

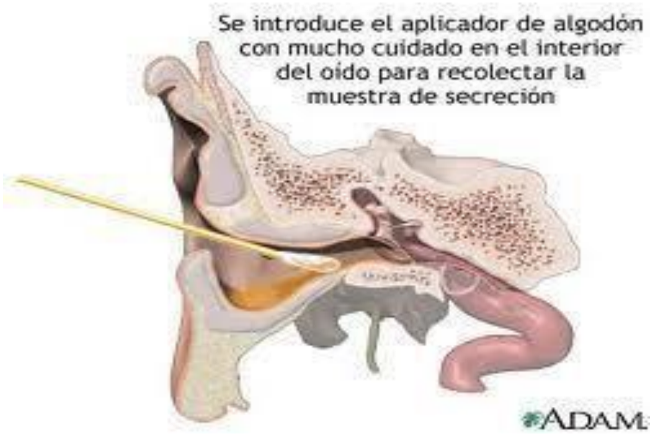

- **SECRECIÓN NASAL:**

  - El paciente no debe aplicarse gotas nasales ni antibiótico 24-48 horas antes de la toma de la muestra, ni debe realizarse baños nasales.
  - Tomar la muestra con un hisopo estéril de las fosas nasales inclinando la cabeza del paciente un poco hacia atrás, introducir el hisopo hasta la parte superior de la nariz rotar muy suavemente, dejando allí por unos segundos y se retira.
  - Recolectar la muestra con escobillon esteril y transportar al laboatorio en tubo de vidrio esteril.

- **Eosinofilos en Moco Nasal:**

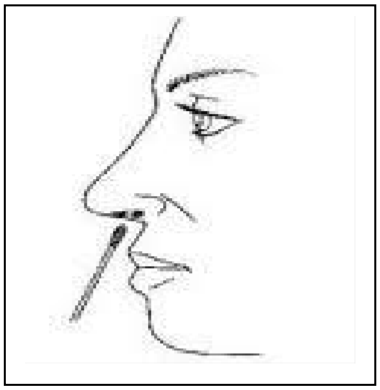

Pedir al paciente que trate de expulsar suavemente la secreción nasal si tiene. Tomar la muestra con un hisopo estéril de las fosas nasales inclinando la cabeza del paciente un poco hacia atrás, introducir el hisopo hasta la parte superior de la nariz rotar muy suavemente, dejando allí por unos segundos y se retira. Tomar dos láminas para coloración de Wright. Dividir la lámina por mitad, marcar la lámina indicando el sitio de toma: Fosa derecha Fosa izquierda

- **SECRECIÓN HERIDA:**

  - Limpiar el borde de la lesión con solución salina estéril
  - Tomar la muestra con un hisopo estéril de las lesiones observadas, si tiene la superficie cicatrizada, se debe levantar la superficie y oprimir suavemente con el fin de obtener material purulento si lo hay.
  - Para los procesos de abscesos, heridas, úlceras o forunculosis el médico debe tomar la muestra con jeringa y aguja estéril.
  - Transportar al laboratorio en recipiente estéril.

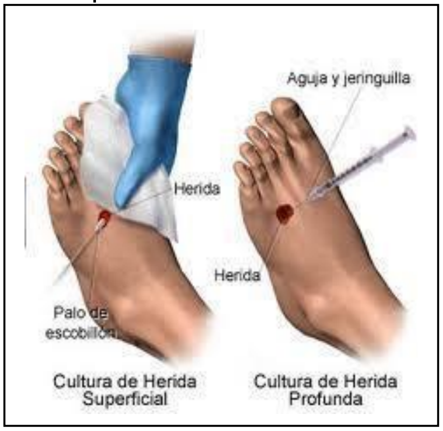

- **Tos ferina**

La recomendación internacional para el diagnóstico de la tos ferina (*Bordetella pertussis o parapertussis)* son tres técnicas:

• Inmunofluorescencia directa positiva (IFD)
• Cultivo para *B. pertussis o B. parapertussis* en medio de REGEN LOWE
• Prueba de PCR positiva para *B. pertussis o B. parapertussis*

Es importante garantizar la muestra del frotis nasofaríngeo o el aspirado nasofaríngeo según el caso, aun cuando el niño o la persona hayan iniciado el tratamiento con antibiótico, pero la recomendación es tomar la muestra antes del inicio del tratamiento, pues esto contribuiría a un mejor diagnóstico por parte del laboratorio.

- La muestra debe ser tomada por la Terapeuta Respiratoria.
- Las muestras para el estudio de contactos o de casos en trabajo de campo se deben tomar mediante frotis nasofaríngeo.
- Las tomas de muestras en pacientes hospitalizados se deben hacer mediante aspirado nasofaríngeo con sonda estéril.
- Introducir la sonda con el aspirado nasofaríngeo en un tubo y/o frasco estéril y transportar al laboratorio.
- Enviar a la Secretaria de Salud de Cundinamarca para su estudio y notificación junto con la ficha epidemiológica y de acuerdo con las condiciones requeridas por este laboratorio.

13.6 HISOPADO NASOFARINGEO

**FICHA TÉCNICA PARA TOMA DE MUESTRAS LABORATORIO CLINICO COMPENSAR**		
**TIPO DE MUESTRA:** Hisopado Nasofaríngeo		**ORIGEN:** Nasofarínge
**POBLACION:** General		**ROL:** Auxiliar de Laboratorio
**TÉCNICA**	**ANTES DE REALIZAR LA TOMA**	
	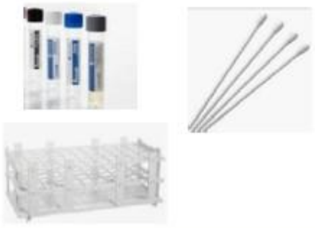	Aplicar instrucciones descritas en la ficha Generalidades de toma de muestras de microbiología.
		Realizar lavado de manos con agua y jabón cada hora, cuando las manos estén sucias, contaminadas con secreciones, cuando se pase de un área contaminada a un área limpia o cuando sea necesario, se debe realizar higienización de manos cuando se requiera según instrucciones establecidas en los 5 momentos de lavado de manos.
		Indicar al paciente la posición que debe adoptar para la adecuada toma de muestra.
	**TÉCNICA DE TOMA DE MUESTRA**	
	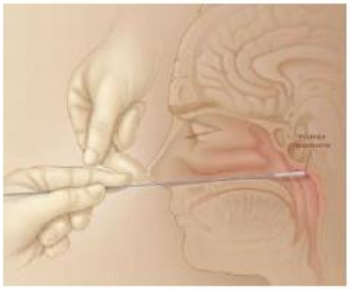	Con la mano libre, llevar hacia atrás la cabeza del paciente y con la otra mano, introducir el hisopo humedecido a través de los orificios nasales, paralelo al paladar (no hacía arriba), hasta que se encuentra resistencia o la distancia equivalente desde la fosa nasal hasta la oreja. En este punto se encuentra la punta en la nasofarínge.
		Rotar suavemente el hisopo por 5 segundos y luego retirar lentamente, permitiendo que se absorban las secreciones en el
		Retirar el hisopo de la fosa nasal y colocarlo inmediatamente en el tubo con el medio de cultivo.
		Repetir el procedimiento en la fosa nasal contra lateral.

KIT TOMA DE MUESTRAS PARA COVID – 19 (HISOPADO NASOFARINGEO)

Las muestras solicitadas para covid 19 por consulta externa serán tomadas por el personal de auxiliar de laboratorio en el módulo 5 de toma de muestras de consulta externa. En los servicios de internación, las muestras serán tomadas por el personal de terapia respiratoria.

Para el procedimiento, el auxiliar de laboratorio debe contar con:

11.1.1 Nevera de transporte con pila refrigerante.
11.1.2 1 Escobillones flexibles de Nylon, Rayon o Dacron
11.1.3 Medio de transporte viral MTV
11.1.4 Frasco de orina (embalaje secundario). Toalla absorvente
11.1.5 Una bolsa ziploc

ELEMENTOS DE PROTECCIÓN PERSONAL

De acuerdo con lo establecido en el manual de bioseguridad (LC.01.MA.18) los elementos de protección personal a utilizar son:

11.1.6 Bata desechable
11.1.7 Gorro desechable
11.1.8 Guantes
11.1.9 Mascarilla N95
11.1.10 Careta

Consultar protocolo de bioseguridad en manual de bioseguridad (LC.01.MA.18). Ver anexo TOMA DE MUESTRA PARA PCR-COVID 19 EN PACIENTE AMBULATORIO.

13.7 TOMA DE HEMOCULTIVOS EN INTERNACIÓN

Objetivo
Proporcionar recomendaciones generales basadas en la evidencia y evitar falsos positivos en la toma de hemocultivos que puedan comprometer los resultados y el tratamiento del paciente.
Introducción
Los hemocultivos son fundamentales para el diagnóstico de las bacteriemias. La invasión de microorganismos en la sangre es una de las causas más significativas en el aumento de la morbi-mortalidad en pacientes, representando una de las causas más prevalentes de infección. El problema es de considerable magnitud, cerca de 200.000 pacientes desarrollan bacteriemias o fungemias anualmente en los Estados Unidos, con una mortalidad atribuible del 20-50%. Se ha calculado que un hemocultivo contaminado causa un incremento de 4 a 5 días en el tiempo de hospitalización y un coste añadido de tratamiento de unos 4000 €. Muchos de estos episodios son nosocomiales y en algunas instituciones representan la mayoría de los casos; igualmente el aumento de la resistencia bacteriana está asociado con una gran morbilidad de los episodios adquiridos en la comunidad. Se define como bacteriemia la presencia de bacterias en la sangre, que se pone de manifiesto por el aislamiento de éstas en los hemocultivos. El término fungemia se utiliza para designar la presencia de hongos en la sangre. Septicemia y sepsis son expresiones que se emplean para denominar el síndrome clínico con el que habitualmente se manifiestan las bacteriemias o las fungemias, independientemente del resultado de los hemocultivos. La bacteriemia y la fungemia son complicaciones graves de las infecciones bacterianas y fúngicas, respectivamente, y tienen una metodología diagnóstica muy similar, por lo que se describirán de forma conjunta. Ambas se producen cuando los microorganismos invaden el torrente sanguíneo y se multiplican a un ritmo que supera la capacidad del sistema reticuloendotelial para eliminarlos. Esta invasión puede producirse desde un foco infeccioso extravascular, a través de los capilares sanguíneos o de los vasos linfáticos, o desde un foco intravascular (endocarditis, infección de catéteres intravenosos o arteriales, etc.). La incidencia de la bacteriemia depende del tipo de población estudiada (5- 30 casos por 1000 pacientes hospitalizados) y puede presentarse a cualquier edad, sobre todo en pacientes con graves enfermedades de base y en los sometidos a maniobras que alteran los mecanismos locales y generales de defensa frente a la infección.
Los focos más frecuentes de bacteriemia identificados son el tracto genitourinario, abscesos, heridas quirúrgicas, tracto biliar y catéteres intravasculares, no obstante, hasta en un 25% de los casos su foco originario es desconocido. La mayoría de los microorganismos son capaces de invadir el torrente circulatorio. En la actualidad, las bacterias grampositivas, especialmente estafilococos y enterococos, igualan o superan en frecuencia a las gramnegativas. Ello es debido a múltiples causas, entre las que destacan la utilización de

antibióticos de amplio espectro, el uso generalizado de catéteres intravasculares y el empleo de métodos invasivos de diagnóstico. Por otra parte, el aumento de pacientes inmunodeprimidos con tratamientos antineoplásicos o con infección por el VIH ha propiciado la aparición de bacteriemias por agentes que en el pasado eran causas muy raras de infección. El diagnóstico definitivo de la bacteriemia y de la fungemia se establece cuando se aísla el microorganismo causal en la sangre del enfermo mediante hemocultivo. El aislamiento del agente responsable es trascendente para conocer su sensibilidad a los antimicrobianos e instaurar el tratamiento o las modificaciones necesarias a la terapia empírica ya establecida. En ocasiones puede orientar el diagnóstico de enfermedades como la neoplasia de colon (asociada a bacteriemia por Streptococcus bovis), endocarditis (estreptococos del grupo viridans) e incluso por el VIH (Salmonella y enterococo). Por otra parte, permite, en la mayoría de las ocasiones, la diferenciación de los casos de verdadera bacteriemia de aquellos en los que la positividad es debida a un inadecuado procedimiento de extracción y procesamiento.

Definición

El hemocultivo es un método diagnóstico que se realiza para la detección de microorganismos en la sangre y así, posteriormente, realizar la identificación y determinación de sensibilidad.

Cuidados y recomendaciones

- Confirmar orden medica en el sistema (Hosvital)
- Realizar lavado de manos quirúrgico
- Mantener técnica aséptica durante todo el procedimiento
- Utilizar campo estéril para evitar contacto con otras áreas para disminuir el riesgo de contagio
- Realizar antisepsia de la zona y no palpar la vena sin guantes estériles una vez preparada la piel
- Cambiar guantes después de cada antisepsia y punción
- Recolectar idealmente de 8 a 10 ml por cada venopunción para adultos
- Recolectar idealmente de 0.5 a 4 ml por cada venopunción para pediatría
- Siempre inocular primero la botella anaeróbica y luego la aeróbica.
- Para los cultivos recolectados por venas periféricas, están recomendadas las siguientes venas: media cubital, cefálicas en miembros superiores.
- La realización de recolección de los hemocultivos por medio de venas en miembros inferiores y por sangre arterial aumentan los riesgos de eventos adversos de contaminación disminuyendo así la posibilidad de recuperación microbiológica.
- La recolección de sangre por vías periféricas ya canalizadas **NO ESTA RECOMENDADA**.
- La toma de sangre por catéter central solo esta indicada para las infecciones asociadas al mismo catéter central, pero debe tomarse posterior a una toma de sangre por vena periférica.
- **NO SE RECOMIENDA** cambiar la aguja en el momento de inocular la sangre en los frascos dado a que esto genera un aumento en accidentes por riesgos biológicos.
- **NO SE RECOMIENDA** la toma de muestras de hemocultivos de la línea arterial
- **No SE RECOMIENDA** la devolución de la sangre de la muestra inicial al paciente**.**

Equipo

- Clorhexidina jabón y solución
- Campos estériles
- Dos pares de guantes por cada toma de hemocultivo
- Gasas estériles
- Torniquete

- Gorro
- Mascarilla quirúrgica o tapabocas
- Bata quirúrgica
- Frascos para hemocultivos
- Tres jeringas estériles

Técnica de recolección de hemocultivos por accesos periféricos

1. Recibir material completo de laboratorio
2. Recibir material médico-quirúrgico completo de farmacia
3. Explicar el procedimiento al paciente y familiar
4. Enfermera y auxiliar deben colocarse gorro y tapabocas
5. Se sugiere la postura de tapabocas para el paciente durante la toma de los hemocultivos
6. Seleccionar vena a puncionar: acceso fácil, buen calibre
7. Lavado de manos quirúrgico según protocolo institucional
8. Colocarse bata quirúrgica
9. Colocarse guantes estériles
10. Colocar los campos estériles alrededor del sitio de punción
11. Aplicar clorhexidina jabón en una gasa estéril y con movimientos de rejilla realizar fricción mecánica del sitio de punción
12. Realizar el mismo procedimiento con clorhexidina solución
13. Dejar secar espontáneamente la piel durante 2 minutos
14. Realizar cambio de guantes con técnica aséptica
15. Colocar torniquete (Auxiliar acompañante)
16. Recibir jeringa estéril y realizar punción en vena seleccionada en un ángulo de 35°
17. Obtener de 8 a 10 ml de sangre de cada frasco para pacientes adultos
18. Obtener cada muestra de tres sitios anatómicos diferentes con un intervalo de 15 minutos. Se puede del mismo sitio anatómico si es una muestra aerobia y otra anaerobia.
19. Retirar torniquete, extraer aguja y realizar presión con gasa en sitio de punción
20. Retirar tapa plástica de la botella, alcanzar aguja estéril y abrir isopañin de alcohol (Auxiliar acompañante) proceder a continuar haciendo presión.
21. Recibir isopañin y desinfectar el tapón de caucho de la botella
22. Inyectar la muestra a través del tapón de caucho de la botella con técnica aséptica
23. Desechar la aguja en el contenedor para cortopunzantes.
24. Marcar los frascos con el sticker que incluye los nombres y apellidos completos, número de documento, escribir el orden y la hora de la toma de los hemocultivos junto con el sitio de punción, esta marcación NO debe tapar el código de barras de los frascos.
25. Lavarse las manos luego de finalizado el procedimiento

Técnica de recolección de hemocultivos por catéter central

1. Recibir material completo de laboratorio
2. Recibir material médico-quirúrgico completo de farmacia
3. Explicar el procedimiento al paciente y familiar
4. Enfermera y auxiliar deben colocarse gorro y tapabocas
5. Lavado de manos quirúrgico según protocolo institucional
6. Colocarse bata quirúrgica
7. Colocarse guantes estériles

8. Cerrar el paso de infusión intravenosa, durante 3 – 5 minutos (dependiendo de la condición del paciente).
9. Elegir un puerto próximo, realizar limpieza por 15 segundos utilizando una solución de Gluconato de Clorhexidina al 2% en asociación con alcohol isopropílico al 70% y permitir que seque.
10. En adultos extraer 20 mL de sangre de la vía y dividir el contenido de la jeringa así: 10 mL para la botella anaeróbica y 10 mL para la botella aeróbica sin cambiar de aguja. Siempre inocular primero la botella anaeróbica y luego la aeróbica.
11. Retirar tapa plástica de la botella, alcanzar aguja estéril y abrir isopañin de alcohol (Auxiliar acompañante).
12. Recibir isopañin y desinfectar el tapón de caucho de la botella
13. Inyectar la muestra a través del tapón de caucho de la botella con técnica aséptica
14. Desechar la aguja en el contenedor para cortopunzantes.
15. Marcar con el código de barras las botellas y escribir el orden y la hora de la toma de los hemocultivos
16. Lavarse las manos luego de finalizado el procedimiento

Volumen de sangre

1. Botellas adultas aerobias y anaerobias: 10 mL
2. Botellas pediátricas: 0.5 a 4 mL. Este volumen de ser de acuerdo al peso del paciente:

Cuadro 1. Volúmenes de sangre a extraer por juego de hemocultivo según edad y peso en población pediátrica.

Población	Edad	Sitio	Volumen Minimo	Botellas
Neonatos	0-28 dias (o pacientes en URN)	vena periferica	<8 kg: 1 mL	Una botella pediatrica aerobica
Niños	1-3 meses	vena periferica	<8 kg: 1 mL	Una botella pediatrica aerobica
	3-36 meses	vena periferica	<8 kg: 1 mL 8-13 kg: 3 mL 13-27 kg: 5 mL	Botella pediatrica aerobica si el volumen es menor de 0,5 - 4 mL Botella aerobica de adulto si el volumen es mayor de 4,0 mL
	4-11 años	vena periferica	8-13 kg: 3 mL 13-27 kg: 5 mL 27-40 kg: 10 mL >40 kg: 10 mL	Botella pediatrica aerobica si el volumen es menor de 0,5 - 4 mL Botella aerobica de adulto si el volumen es mayor de 4,0 mL
	12-17 años	vena periferica; considerar dos venas de sitios separados para 2 cultivos	27-40 kg: 10 mL >40 kg: 10 mL	Botella pediatrica aerobica si el volumen es menor de 0,5 - 4 mL Botella aerobica de adulto si el volumen es mayor de 4,0 mL

Fuente: Manual de Toma de Muestra para análisis microbiológico LSP Bogotá

Tipo de botella

Tapa verde: Adulto aerobia
Tapa amarilla: Pediátrica aerobia
Tapa naranja: Anaerobia

Identificación de la muestra
Cada frasco debe ser identificado con los siguientes datos:

- Nombres y apellidos completos del paciente
- Numero de documento de identidad
- Tipo de muestra y sitio anatómico
- Numero de recolección de hemocultivos
- Fecha y hora de recolección

Trasporte

- Se recomienda que sea trasportada en los primeros 15 minutos de la recolección a temperatura ambiente.

Indicadores de calidad de hemocultivos

1. Contaminación:

   % de contaminación de
   hemocultivos. Formula:
   No. de hemocultivos contaminados en el mes (numerador) No.
   de botellas procesadas en el mes x 100 (denominador)

2. Positividad:

   % de hemocultivos positivos
   Formula:
   No. de hemocultivos positivos en el mes (numerador)
   No. de botellas procesadas en el mes x 100 (denominador)

3. Volumen
   % de botellas con volumen adecuado

Formula:

No. De botellas con volumen adecuado (numerador
No. De botellas tomadas en el mes x 100 (denominador)

13.8 Hemocultivos en consulta externa:

El paciente debe estar con pico febril para la toma de la muestra, se puede tomar en cualquier momento del día o de la noche, previamente haber realizado el proceso de facturación.

- Limpiar el mesón muy bien con Surfanios.Utilizar el mechero y tomar la muestra cerca de éste para crear un campo estéril, así mismo utilizar tapabocas y gorro.
- Con guante estéril preparar la piel para la venopunción, desinfectar desde el codo hasta la muñeca con alcohol antiséptico.
- Tomar la muestra de sangre con venopunción normal.
- Las muestras de hemocultivos se deben tomar en 3 sitios diferentes, una botella por cada sitio; (por ejemplo: central brazo derecho, central brazo izquierdo y lateral derecho).
- Tomar de 3-5 ml de sangre en adultos y de 1-3 ml de sangre en niños, en las botellas indicadas según la orden médica.
- Para adultos se deben tomar 3 botellas y 2 botellas para niños.
- En niños lo ideal es tomar de dos sitios anatómicos diferentes, pero teniendo en cuenta que en pacientes pediátricos el acceso venoso muchas veces es difícil se podría tomar del mismo sitio anatómico.
- Se puede usar la botella de hemocultivo como un tubo normal ya que ésta tiene vacío y nos llena lo que necesita la botella
- Se debe tomar cada 20 minutos, el paciente no debe tomar ningún antipirético hasta tanto no terminar el esquema.
- Marcar con el código de barras las botellas y escribir el orden y la hora de la toma de los hemocultivos.
- Escribir datos clínicos relevantes, medicación, tiempo de inicio de los síntomas.
- Entregar a la bacterióloga responsable de Microbiología para su incubación.

14. TOMA DE MUESTRAS MICOLOGICAS

Para la toma de muestra de hongos con solicitud de KOH y/o cultivo de micosis de cualquier sitio anatómico verificar que el paciente no esté tomando antimicóticos, si ha recibido tratamiento debe esperar al menos quince (15) días para tomar la muestra para hongos. No se haya aplicado ningún tipo de crema y/o ungüento en la a lesiones. Si la muestra es de uñas de las manos no debe tener esmalte, crema; si es de las uñas de los pies no debe aplicarse talco, ni cremas. El auxiliar de Laboratorio debe verificar el sitio de la lesión para realizar la toma adecuada de la muestra solicitada. Ante cualquier duda debe confirmar con la bacterióloga de microbiología para orientar la toma de la muestra.

14.1 CUERO CABELLUDO:

- Escoger la porción del pelo que nos interese para el examen.
- En caso de una piedra examinar el pelo en el sitio donde está la piedra, cortar con tijeras y en caso de una tiña escoger la porción cercana a la raíz, cortando el pelo o halando para que salga con raíz. La muestra debe ser 5a 10 pelos. Depositar la muestra en un frasco estéril para Laboratorio,
- Realizar un raspado con bisturí de la parte de la lesión y dejar el bisturí en el frasco.
- Marcar las muestras con el rotulo de código de barras especificando si es sólo directo y/o con cultivo y la ubicación exacta de la muestra.
- Además, utilizar la técnica de la cinta Pegante: Consiste en colocar una tira de cinta pegante de 1.5 a 2 cms de ancho sobre la lesión, colocarla una (1) sola vez sobre la piel, retirar y colocar sobre una lámina portaobjetos a la cual se la ha adicionado 1 gota de KOH al 20 % y pegar la cinta a la lámina; hacer 2 láminas;

  identificadas con número de referencia e iniciales del nombre y apellido del paciente y sitio de toma de la muestra.

.

14.2 ESCAMAS - PIEL:

- En caso de la pitiriasis versicolor utilizar la técnica de la cinta Pegante: Consiste en colocar una tira de cinta pegante de 1.5 a 2 cms de ancho sobre la lesión, colocarla una (1) sola vez sobre la piel, retirar y colocar sobre una lámina portaobjetos a la cual se la ha adicionado 1 gota de KOH al 20 % y pegar la cinta a la lámina; hacer 2 láminas; identificadas con número de referencia e iniciales del nombre y apellido del paciente y sitio de toma de la muestra. muestra.
- Recolectar muestra empleando un bisturí: raspar el borde de varias lesiones para obtener mayor cantidad de material, seleccionar preferentemente las zonas en la que se observen bordes sobre elevados, eritematosos y descamantes, o en la periferia de las lesiones y en aquellos casos en los que presenten ampollas se seccionará el techo de la misma.
- Depositar la muestra en un frasco estéril para hongos, colocar el bisturí dentro del frasco, con la respectiva identificación de código de barras y ubicación exacta de la muestra.

14.3 UÑAS

- Tomar la muestra de la lesión de la uña utilizando la técnica de tornillo haciendo girar la punta del bisturí sobre la lesión de la uña, (utilizar la técnica solo si el paciente aprueba la toma luego de explicarle en qué consiste).
- Hacer un raspado por debajo de la uña con la punta del bisturí desde el extremo proximal al distal y permitiendo que la muestra caiga en el frasco estéril para hongos, colocar el bisturí dentro del frasco
- En caso que la muestra sea de los dedos de los pies (Artejos) se deben enumerar. Y seguir el procedimiento anterior.
- Para toma de hongos en secreciones de garganta y vaginales, no se genera otro examen, sino se incluye en la coloración de Gram.

14.4 PUS, EXUDADOS, SECRECIONES, HERIDAS:

Para las muestras de hongos de este tipo de muestra:

- Tomar las muestras con un hisopo estéril, depositarlo en tubo esteril para transportar al laboratorio. Rotular con la respectiva identificación de código de barras y ubicación exacta de la muestra.

14.5 ORINA PARA KOH:

El paciente debe recoger la primera orina de la mañana previo aseo genital. Si el paciente está hospitalizado la muestra debe ser tomada preferiblemente con catéter estéril. Procesar la muestra inmediatamente; el valor de los estudios micológicos con esta muestra es limitada. Sin embargo, en pacientes hospitalizados el médico debe tener en cuenta la historia clínica, el estado clínico del paciente para determinar si la Cándida es patógena o no.

15. MUESTRAS PARA DIAGNOSTICO DE TUBERCULOSIS

15.1 ESPUTO

- Laboratorio clínico dispone de un KIT que consta de tres frascos boca ancha, tapa rosca especiales para la recolección de estas muestras. Dentro del kit se incluye un instructivo para dar la indicación al paciente para la recolección de la muestra.
- Para pacientes de consulta externa lo ideal es recolectar una muestra cada día. Sin embargo, se debe procurar la entrega de las tres muestras dando facilidad al paciente.
- Para pacientes de urgencias, se deben recolectar las muestras cada hora garantizando así la completitud del esquema.
- Pacientes hospitalizados pueden recolectar las muestras una cada día.

15.2 ORINA PARA CULTIVO TBC:

El paciente debe recoger el total de la primera micción de la mañana en un recipiente de boca ancha, plástico (para facilitar su incineración), con cierre hermético para evitar que se derrame y protegida de la luz directa. Se debe tomar tres muestras seriadas en días diferentes, recolectadas preferiblemente en el momento en que el paciente se despierta. Las muestras se deben recoger siguiendo siempre las mismas condiciones. La muestra debe estar protegida de la luz, recomendar al paciente colocar la muestra en bolsa oscura y debidamente marcada con el nombre.

15.3 ASPIRADO GASTRICO:

El cultivo de aspirado gástrico es primordial para el diagnóstico de tuberculosis o de micobacteriosis en pacientes adultos y niños que no expectoran y degluten sus esputos. La toma de muestra requiere de un trabajo del equipo intrahospitalario. Es fundamental llevarla a cabo en las mejores condiciones, Se debe recolectar la muestra en un envase adecuado: tubos tapa rosca estériles a los que se le adiciona 2 ml de Trifosfato sódico al 10% para cada 10 ml de aspirado gástrico. Esta adición se realiza a los tubos estériles previa solicitud del servicio donde se va a realizar el procedimiento. Se requiere tomar muestras seriadas durante tres días consecutivos con el fin de aumentar la posibilidad diagnóstica. El procedimiento de toma de muestra lo realiza una enfermera jefa del servicio solicitante de la siguiente manera: Se pasa la sonda naso gástrica la noche anterior; se fija y se marca el punto de fijación. Antes de despertar al paciente, se aspira con jeringa el contenido gástrico. Se deposita el aspirado en el tubo estéril para permitir la neutralización del pH ácido del contenido gástrico, pues afecta la viabilidad de las micobacterias. Se Inyectan 50 ml de agua destilada estéril y se aspira nuevamente; se coloca lo aspirado en el mismo tubo estéril, la

cantidad mínima recuperada debe ser de 10 a 20 ml. La muestra de aspirado gástrico destinada para cultivo, se debe enviar al laboratorio protegida de la luz directa y evitando que se derrame. Ver manual de Microbiología.

16. MUESTRAS PARA DIAGNOSTICO DE LEPRA

BACILO DE HANSEN:

- La muestra es tomada por el auxiliar
- Se debe tomar en total 6 muestras (láminas nuevas desengrasadas), seis muestras que son: (1) linfa de lóbulos de la oreja, (1) linfa de la rodilla, (2) linfas de codos y (2) lesiones, En el caso que no tenga lesiones se reemplaza por linfa de codos o linfa de orejas. Se elimina la toma de muestras de moco por que se han presentado falsos positivos. Se tomarán así:
- Una (1) muestra de lóbulos de las orejas (1) rodilla:
- Se efectúa limpieza del sitio con alcohol; para tomar una buena muestra es importante dejar libre de sangre el sitio donde se va a tomar la muestra, usar pinzas sin garra tipo Kelly o clamp.
- Con una lanceta se hacen 3 o 4 punciones cercanas entre si con el fin de obtener una buena muestra, tomar la muestra cuando se observe que el sitio de toma de muestra este completamente pálido con el fin de garantizar la ausencia de sangre.
- Dos (2) muestra: de codos
- Una (2) muestra de lesiones activas, si no hay lesiones se reemplaza por linfa de codos u orejas.
- Siempre deben describir en una hoja adicional el sitio de toma exacto de todas las muestras, con antecedentes de la enfermedad y la respuesta a los siguientes interrogantes:

- Condiciones de vivienda (si el piso es en cemento o tierra, si las paredes son en cemento)
- Si viaja con frecuentemente a zonas endémicas.
- Antecedentes familiares de lepra.
- Pérdida de sensibilidad. Describir si el paciente no siente dolor al momento de tomar la Muestra.
- Caída de cejas y/o pestañas.
- Engrosamiento de la piel de nariz y pabellón auricular.
- Si es control, cuánto tiempo lleva con tratamiento y si lo está tomando en forma indicada.

Debe identificarse en la lámina la procedencia de cada muestra que se tome y el lado derecho o izquierdo de la lámina.

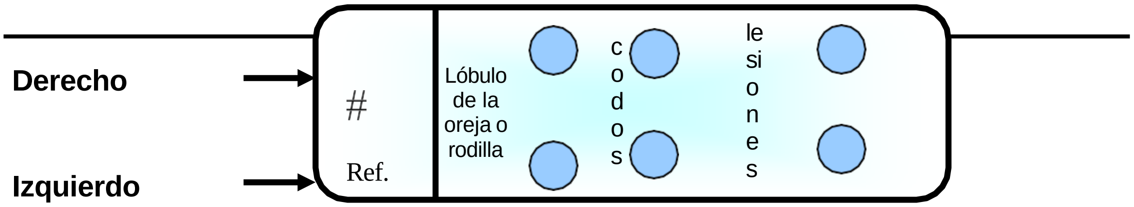

- Llevar las muestras a la unidad de Microbiología para que la bacterióloga procese de manera inmediata las láminas, Si no es posible llevar a cabo el procedimiento conservar

las muestras a temperatura ambiente por un periodo no mayor a 4 horas.

17. TOMA DE ANTIGENO PROSTATICO ESPECÍFICO Y LIBRE

El paciente debe tener en cuenta lo siguiente:

- Si le han realizado biopsias prostáticas o prostactestomia debe esperar al menos dos (2) semanas para la realización del examen.
- Si le han practicado masaje prostático debe esperar por lo menos 4 o 5 días. Si le han realizado tacto rectal no hay restricción de tiempo.
- Tener una abstinencia sexual de 2 días.
- No haber montado bicicleta ni caballo dos días antes.

18. PROLACTINA

- Si el médico lo solicita se realiza pool de prolactina, el paciente debe disponer de al menos una hora para permanecer en el laboratorio. Toma de muestra: Basal, 20 y 40 minutos.
- Debe presentarse en el laboratorio dos (2) horas después de levantarse.
- Abstinencia sexual por lo menos de 1 día.
- No haber ingerido lácteos el día anterior al examen.

19. DOSIFICACION DE MEDICAMENTOS Y NIVELES HORMONALES

- No tomar medicamentos antes de la toma de la muestra excepto por prescripción médica.
- Informar al laboratorio medicamentos y dosis que esté tomando, así como la hora de la última dosis.

20. TOMA DE MUESTRAS CON ESTIMULOS

No se practican en esta sede examenes que requieren estimulación quimica o nerviosa. Se le brindara al paciente la información de la sede o el Laboratorio al que debe asistir para la(s) pruebas y se hara entrega del Formato De Remision De Muestras Y/O Pacientes A Laboratorio Inscrito, para que lo presente en el momento de asistir al Laboratorio de Referencia previa solicitud de cita en el Laboratorio de Referencia, se le explicara de manera clara la informacion relacionada con el laboratorio de referencia por cualquier duda que tenga el paciente quien dejara constancia del entendimiento firmando la copia del formato de remisión queda como soporte en esta sede.

21. TOMA DE MUESTRAS PARA LEISHMANIA

FICHA TÉCNICA PARA TOMA DE MUESTRAS
LABORATORIO CLINICO COMPENSAR

compensar | salud

**TIPO DE MUESTRA:** Frotis de lesión	**ORIGEN:** Lesión especificada en orden médica
**POBLACION:** General	**ROL:** Auxiliar de Laboratorio o Bacteriólogo

ASPECTOS GENERALES

TÉCNICA

ANTES DE REALIZAR LA TOMA

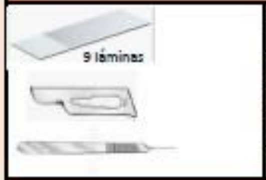

	Aplicar instrucciones descritas en la ficha Generalidades de toma de muestras de microbiología.
	Realizar lavado de manos con agua y jabón cada hora, cuando las manos estén sucias, contaminadas con secreciones, cuando se pase de un área contaminada a un área limpia o cuando sea necesario, se debe realizar higienización de manos cuando se requiera según instrucciones establecidas en los 5 momentos de lavado de manos.
	Indicar al paciente la posición que debe adoptar para la adecuada toma de muestra.

SELECCIÓN DEL SITIO DE TOMA DE MUESTRA

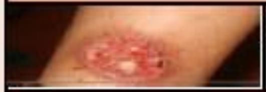

Evaluar lesiones: tener presente si existen dos o más lesiones debe escogerse para el examen directo la que tenga un menor tiempo de evolución.

PREPARACIÓN DEL SITIO DE LA TOMA DE MUESTRA

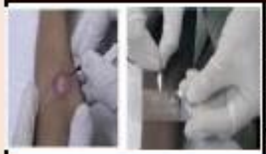

	Realizar una limpieza del sitio de la lesión, utilizando gasa impregnada ya sea de alcohol, solución salina y/o jabón quirúrgico. Si hay costra se debe remover cuidadosamente, colocando compresas de solución salina estéril por lo menos tres momentos antes de la toma de la muestra para ablandar la costra, dejar caer sobre la lesión solución salina a presión, al tiempo ir retirando la costra de arriba hacia abajo
	Elegir la lesión más reciente.

TÉCNICA DE TOMA DE MUESTRA

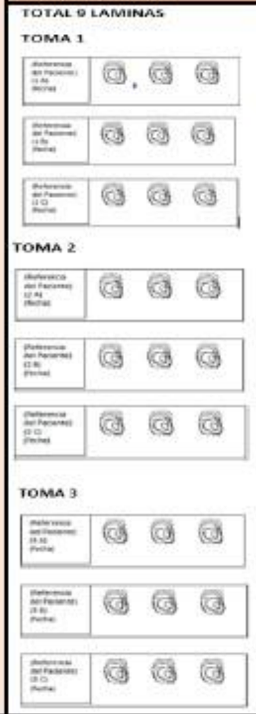

Frotis (raspado) del borde interno de la úlcera:	Tomar 3 láminas de la lesión de 3 aposiciones cada una.
	Obtener tejido con hoja de bisturí número 15 del borde activo de la lesión o del centro de la úlcera, realice un raspado del fondo de la úlcera.
	Hágalo de manera tal que no sangre mucho, presionando el sitio de la lesión hasta hacer isquemia.
	El material obtenido se extiende en forma suave sobre una lámina portaobjetos nueva, previamente limpia, desengrasada, y debidamente rotulada
Incisión y raspado del borde activo de la lesión:	Este método se recomienda para lesiones cerradas, no ulceradas.
	Realizar la limpieza del sitio de la lesión como se describió anteriormente.
	Tomar 3 láminas de la lesión de 3 aposiciones cada una.
	Si el paciente manifiesta dificultad en asistir en tres días diferentes para la toma de muestra, dividir la lesión en tres partes y de cada zona tomar 3 láminas con sus 3 aposiciones para un total de 9 láminas en las tomas del mismo día.
	Sobre el borde activo de la lesión, realice una pequeña incisión con una hoja de bisturí de 3 a 6 mm de longitud por 1 a 3 mm de profundidad. La isquemia se debe lograr haciendo presión en pinza con los dedos. Se debe ejercer presión en la lesión para hacer isquemia (torniquete para miembros inferiores y superiores).
	Con gasa estéril, limpie la sangre que emana de la incisión y con la misma gasa presione el borde de la lesión para hacer isquemia, la muestra debe ser linfa y debe estar lo menos contaminada con sangre posible.
	Con el borde romo de la hoja de bisturí levante la piel de la parte superior de la incisión y raspe tejido del interior de la incisión desde la profundidad hacia la superficie.
	El material así obtenido se extiende en forma suave sobre la lámina portaobjetos nueva, previamente limpia, desengrasada, y debidamente rotulada.
	Muestras nodulares y papulares no se toman por personal de laboratorio, es un procedimiento médico

22. BIBLIOGRAFÍA

- Manual de Calidad Pre analítica. Servicio Andaluz de Salud. 2001
- AEBM, AEFA y LABCAM. El Laboratorio Clínico: Pre analítica de muestras de Orina.2005 Servicio Andaluz de Salud. Hospital Regional Universitario Carlos Haya.
- Laboratorio Clínico. Protocolo de extracción venosa. Manual de Calidad. 2009. [consultado el 1 de septiembre de 2010]. Disponible en: http://www.carloshaya.net/chchaya/ UGC/laboratorios/Procedimientos/01_ Pre analíticos/02_Obtencion_Especimenes/ PRO08A.pdf.
- SOCIEDADE BRASILEIRA DE PATOLOGIA CLINICA/ MEDICINA LABORATORIAL, Recomendações da Sociedade Brasileira de Patologia Clínica/ Medicina Laboratorial para Coleta de Sangue Venoso. 2005. Basado en: CLINICAL AND LABORATORY STANDARDS INSTITUTE. NCCLS - H03A5 - Procedures for the collection of diagnostic blood specimens by venipuncture; Approved Standard 5ed.· Protocolo de enfermedad similar a la influenza, Ministerio de La Protección Social, Instituto Nacional de Salud, 2011
- Adaptado: SOCIEDADE BRASILEIRA DE PATOLOGIA CLINICA/ MEDICINA LABORATORIAL, Recomendações da Sociedade Brasileira de Patologia Clínica/ Medicina Laboratorial para Coleta de Sangue Venoso. 2005. Basado en: CLINICAL AND LABORATORY STANDARDS INSTITUTE. NCCLS - H03A5 - Procedures for the collection of diagnostic blood specimens by venipuncture; Approved Standard *5ed*.
- Generalitat de Catalunya. Departament de Sanitat i Seguretat Social. Requisits que han de complir els mòduls d'obtenció de mostres, així com la seva conservació i transport posterior al laboratori. Barcelona, diciembre de 2001.
- National Committee for Clinical Laboratory Standards. *Procedimiento para la manipulación y procesamiento de los especímenes de sangre; Guía aprobada*. NCCLS Documento H18-A. Villanova: NCCLS, 1990.
- González-Oller C, Alsina MJ. *Base de datos sobre estabilidad de las magnitudes biológicas*. National Committee for Clinical Laboratory Standards. *Análisis de orina y recogida, transporte y*
- Conservación de los especímenes de orina; Guía aprobada. NCCLS Documento GP16-A Villanova: NCCLS.1995.
- Guía de uso de Productos BD Vacutainer. Orden de toma recomendada por Clinical and Laboratory Standards Institute. (CLSI)
- Para la toma, transporte, conservación y remisión de muestras de Laboratorio Clínico. PRO-PSS-0080. Daruma, Compensar.

23. ANEXOS

CÓDIGO	TÍTULO	RESPONSABLE
**LC.01.MA.02.FT.01**	Consentimiento informado para toma de muestras	Bacterióloga
**LC.01.MA.02.FT.02**	Consentimiento informado para cargas de Glucosa	Bacterióloga

**LC.01.MA.02.FT.03**	Formato responsabilidad muestras con fallas en la rotulación	Bacterióloga
**LC.01.MA.02.FT.04**	Disentimiento informado para toma de muestras de laboratorio clínico	Bacterióloga
**ANEXO 1**	Toma de muestra para pcr-covid 19 en paciente ambulatorio	Bacterióloga
**ANEXO 2**	Tabla de elementos de protección personal	Bacterióloga
**ANEXO 3**	Encuesta de datos clínicos prueba rápida SARS CoV 2	Bacterióloga
**ANEXO 4**	Consentimiento Informado para el procesamiento de pruebas serológicas Covid-19.	Bacterióloga
**ANEXO 5**	Centrifugación de muestras	Bacterióloga

ELABORÓ	REVISÓ	APROBÓ
**Nombre:** Yury Andrea Quintero<br><br>**Cargo:** Bacterióloga Laboratorio Clínico<br><br>**Firma:**	**Nombre:** Luz Helena barón<br><br>**Cargo:** Administradora Laboratorio Clínico<br><br>**Firma:**	**Nombre:** Hermencia Carolina Aponte Murcia<br><br>**Cargo:** Subdirectora de Calidad, Educación e Investigación<br><br>**Firma:**

ANEXO 1
TOMA DE MUESTRA PARA COVID 19 EN PACIENTE AMBULATORIO

La sede Compensar Clínica Universidad de la Sabana esta adherida a las directrices referidas en el "Instructivo para la Prevención, contención y atención de COVID 19 INS-PSS-576" y de ese documento se define lo siguiente.
Para el procedimiento, el auxiliar de laboratorio debe contar con:

i. Nevera de transporte con pila refrigerante.
ii. 2 Escobillones flexibles de Nylon, Rayon o Dacron
iii. Medio de transporte viral MTV
iv. Frasco de orina (embalaje secundario). Toalla absorbente
v. Una bolsa ziploc.
vi. Elementos de protección personal.
vii. Bolsa para transporte de careta.

De acuerdo con el número de pacientes agendados, debe contarse con la cantidad suficiente de elementos.

Toma de muestra de hisopado nasofaríngeo en consulta externa:

La toma de muestra está a cargo de auxiliar de laboratorio. Previo a la toma de muestra, se debe realizar el marcado de la muestra registrando los correctos en la marcación:

- Nombres completos
- Número de identificación
- Hora de toma de muestra
- Tipo de muestra

1. La información debe ser verificada con doble chequeo con el paciente y verificando un documento en físico.
2. Realizar la verificación del diligenciamiento correcto de las fichas de notificación de datos básicos y 346.
3. Realizar firma de consentimiento informado para toma de muestras. **LC.01.MA.02.FT.01**
4. Realizar diligenciamiento de autorización de envío de resultados de laboratorio clínico a pacientes **LC.01.MA.01.FT.01.** cuando sea solicitado el envío por correo electrónico por el paciente.
5. En caso de toma de muestra para antígeno de COVID – 19, realizar la encuesta "*ENCUESTA DE DATOS CLÍNICOS PRUEBA RÁPIDA SARS CoV 2 (COVID-19) DETECCIÓN DE ANTÍGENO"*
6. Verificar orden médica versus factura.
7. Posterior a la toma de pacientes agendados, se trasladará al laboratorio y se realizará el ingreso a Enterprise. Entregar stiker de código de barras y nevera de transporte en microbiología para su verificación, y remisión si corresponde.

TOMA DE MUESTRA PCR-COVID 19

Se tomará hisopado nasofaríngeo de acuerdo con la técnica establecida:

FICHA TÉCNICA PARA TOMA DE MUESTRAS LABORATORIO CLINICO COMPENSAR		compensar salud
**TIPO DE MUESTRA:** Hisopado Nasofaríngeo		**ORIGEN:** Nasofaringe
**POBLACION:** General		**ROL:** Auxiliar de Laboratorio
**TÉCNICA**	**ANTES DE REALIZAR LA TOMA**	
	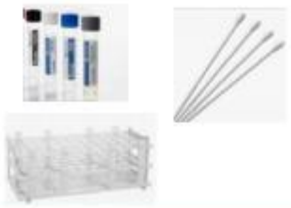	Aplicar instrucciones descritas en la ficha Generalidades de toma de muestras de microbiología.
		Realizar lavado de manos con agua y jabón cada hora, cuando las manos estén sucias, contaminadas con secreciones, cuando se pase de un área contaminada a un área limpia o cuando sea necesario, se debe realizar higienización de manos cuando se requiera según instrucciones establecidas en los 5 momentos de lavado de manos.
		Indicar al paciente la posición que debe adoptar para la adecuada toma de muestra.
	**TÉCNICA DE TOMA DE MUESTRA**	
	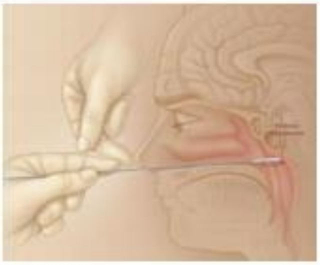	Con la mano libre, llevar hacia atrás la cabeza del paciente y con la otra mano, introducir el hisopo humedecido a través de los orificios nasales, paralelo al paladar (no hacía arriba), hasta que se encuentra resistencia o la distancia equivalente desde la fosa nasal hasta la oreja. En este punto se encuentra la punta en la nasofaringe.
		Rotar suavemente el hisopo por 5 segundos y luego retirar lentamente, permitiendo que se absorban las secreciones en el
		Retirar el hisopo de la fosa nasal y colocarlo inmediatamente en el tubo con el medio de cultivo.
		Repetir el procedimiento en la fosa nasal contra lateral.

TOMA DE MUESTRA DE HISOPADO NASOFARINGEO PARA ANTIGENO DE COVID 19

Para la prueba de antigeno de COVID 19, la toma de muestra se debe realizar de acuerdo con lo especificado en el inserto de la prueba:

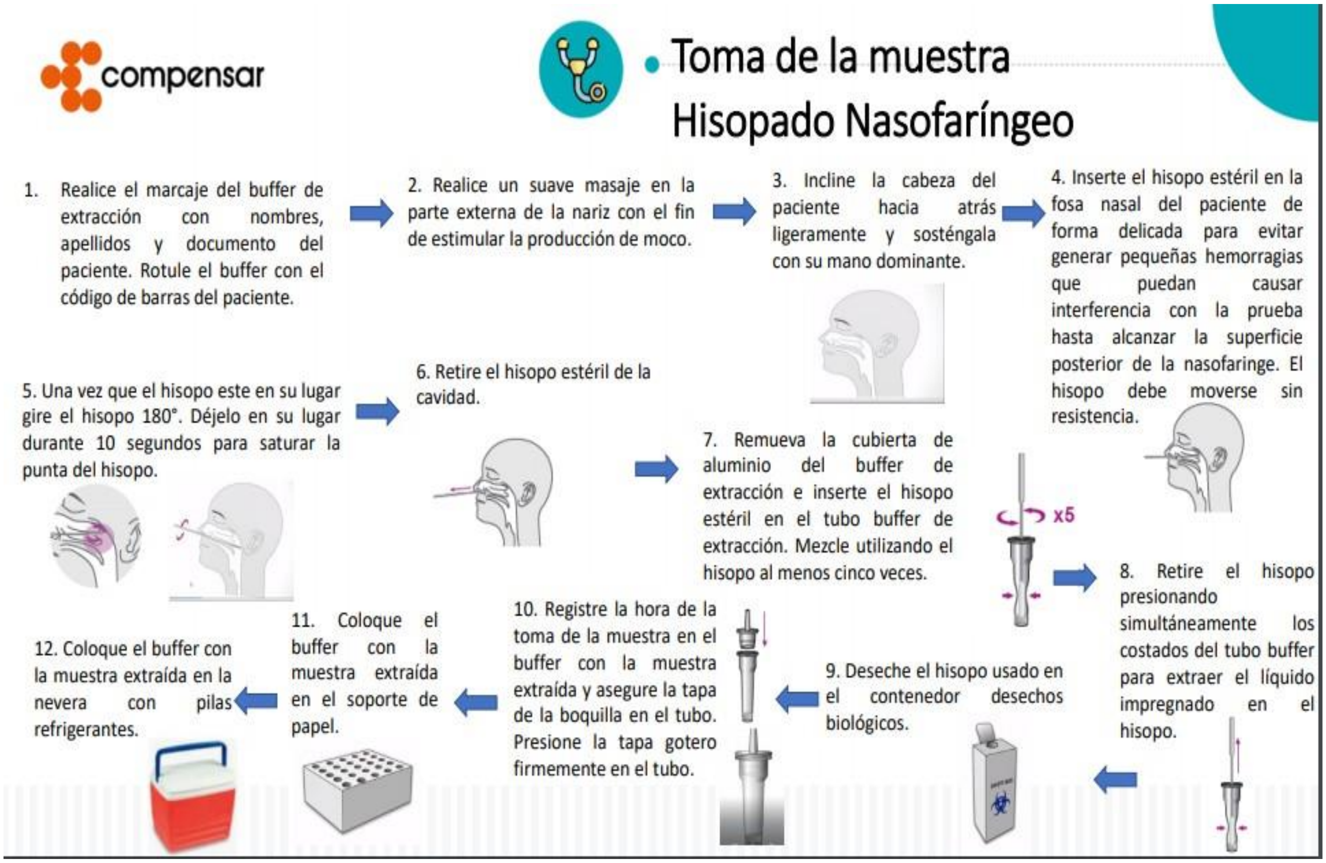

BIOSEGURIDAD

Para el procedimiento, la auxiliar utilizará los elementos de protección personal establecidos: (Ver tabla de elementos de protección personal. Anexo 4)

SUBPROCESO	PERFIL	ESPECIFICIDAD DE LA ACTIVIDAD											
LABORATORIO	BACTERIOLOGÍA	Procesamiento de muestras COVID19 fase de alistamiento y extracción	👍	👍	👎	👍	👍	👎	👍	👍	👎	👍	👎
		Procesamiento de muestras de rutina (hematología, bioquímica, estre otros)	👍	👍	👍	👎	👍	👎	👍	👍	👎	👎	👎
		Procesamiento de muestras para otros virus respiratorios	👍	👍	👎	👍	👍	👎	👍	👍	👎	👎	👎
		Procesamiento de baciloscopias montaje laminas	👍	👍	👎	👍	👍	👎	👍	👍	👎	👎	👎
		Procesamiento de muestras para micobacterias fase de siembra	👍	👍	👎	👍	👎	👎	👍	👍	👎	👍	👎
	AUXILIAR LABORATORIO	Toma de muestras sanguíneas en sede	👍	👍	👍	👎	👍	👎	👍	👍	👎	👎	👎
		Toma de muestras sanguíneas en domicilio	👍	👎	👍	👎	👍	👎	👍	👍	👎	👎	👎
		Toma de muestras sanguíneas a pacientes COVID19 (+)	👍	👍	👍	👎	👍	👎	👍	👍	👎	👎	👎
		Toma de hisopado nasofaríngeo	👍	👍	👎	👍	👍	👎	👍	👍	👎	👎	👎

- Realizar lavado de manos con agua y jabón entre cada paciente. Cambio de guantes entre paciente.
- El gorro, bata y mascarilla convencional se descartan al finalizar la toma de los pacientes agendados.
- Realizar aspersión con alcohol al 70% a la careta. Depositarla en bolsa plástica para ser transportada al laboratorio donde se realiza el lavado con deterganios para su desinfección.
- Descarte de EPP en bolsa roja.
- Se debe realizar limpieza terminal al finalizar la toma de los pacientes agendados. Avisar a personal de aseo al finalizar.
- El paciente debe ingresar con mascarilla convencional y realizar higiene de manos con gel antibacterial. Durante la toma de la muestra, solo debe descubrirse la nariz.

Orden para colocación y retiro de los elementos de protección personal:

COLOCACIÓN	RETIRO
1. Lavado de las manos.<br>2. Bata clínica desechable.<br>3. Gorro<br>4. Guantes<br>5. Tapabocas quirúrgico o N95.<br>6. Gafas de protección o careta.	1. Guantes<br>2. Bata o delantal.<br>3. Gafas o escudo facial.<br>4. Lavado de manos<br>5. Tapabocas<br>6. Gorro

LAVADO DE MANOS

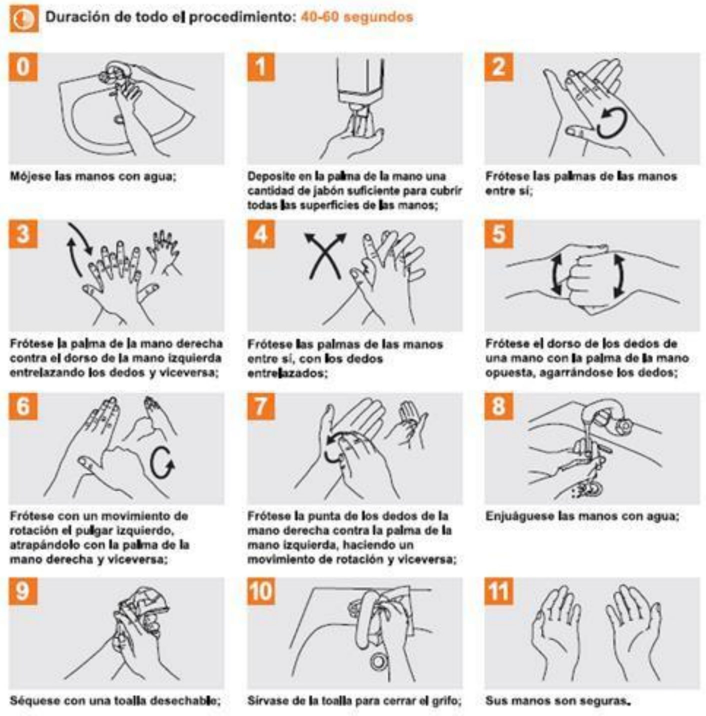

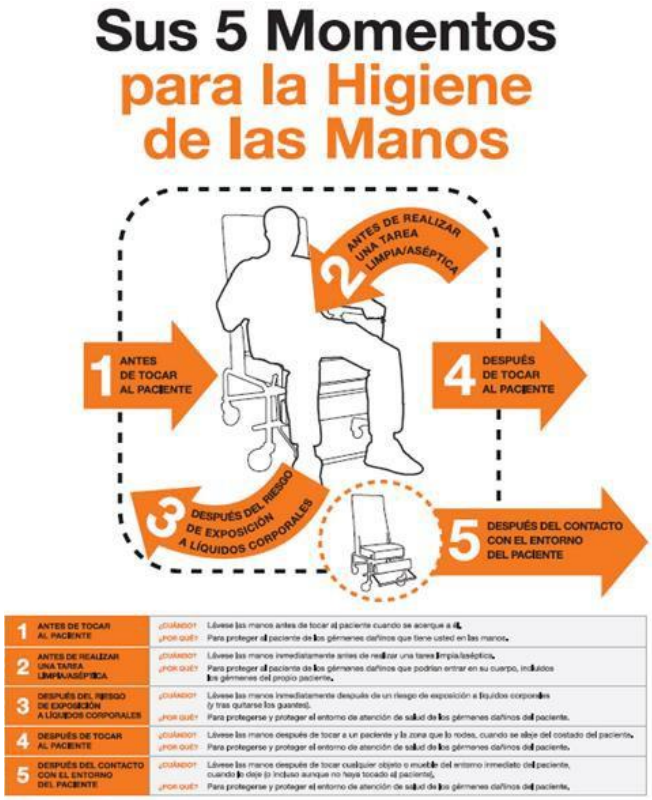

USO ADECUADO DE TAPABOCAS CONVENCIONAL

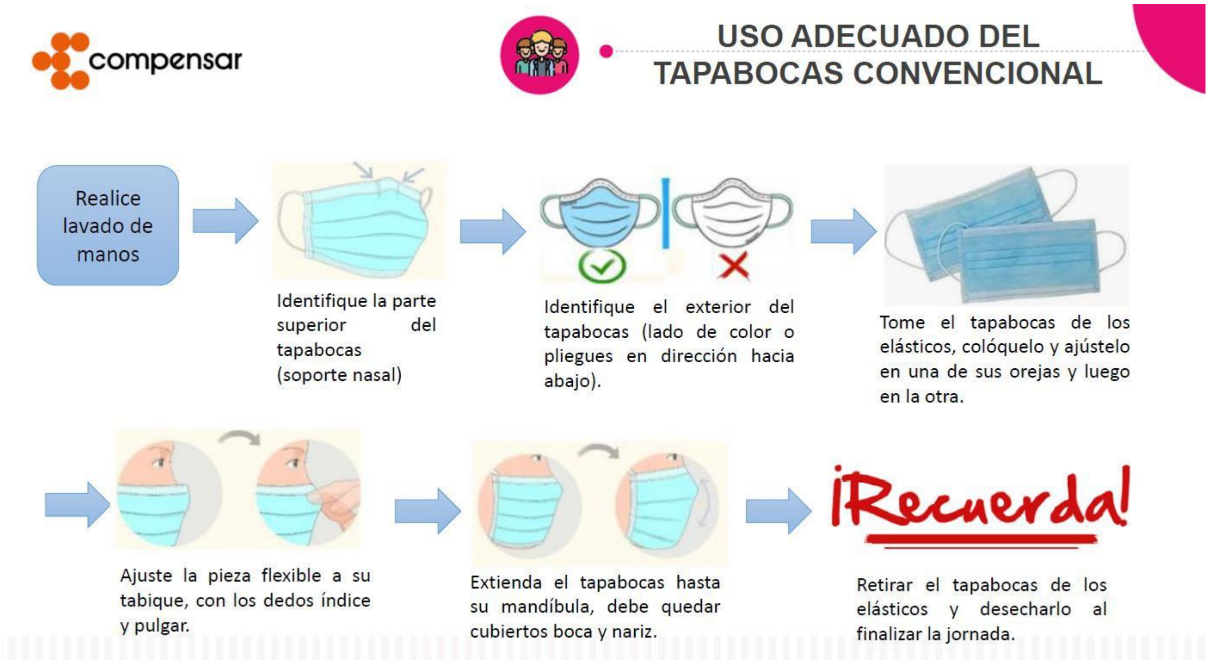

USO DE MASCARILLA N95

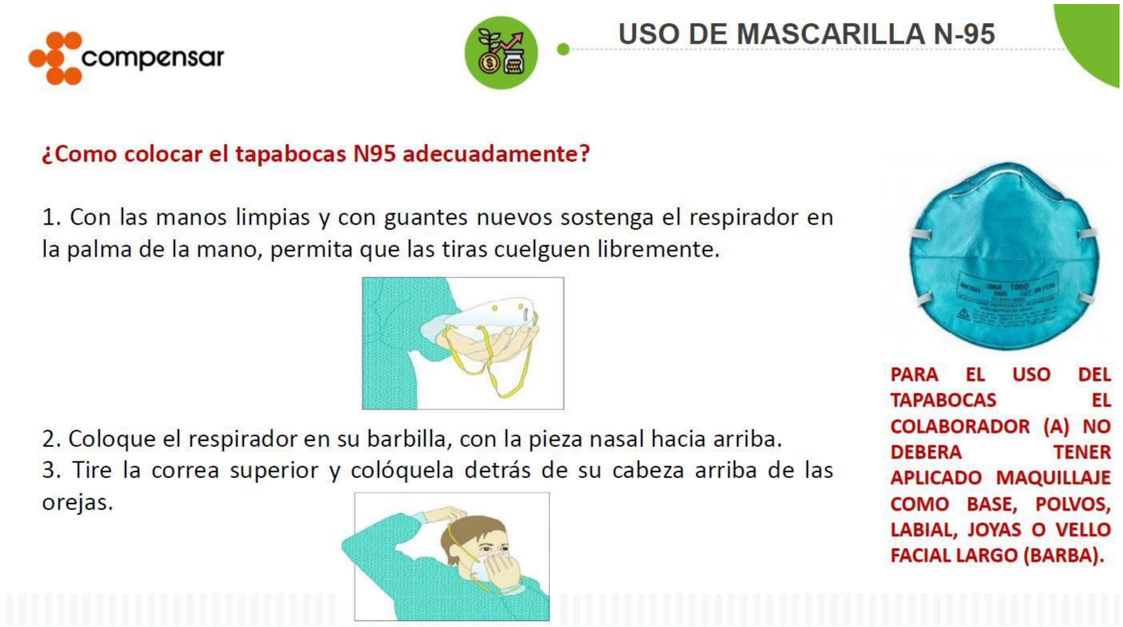

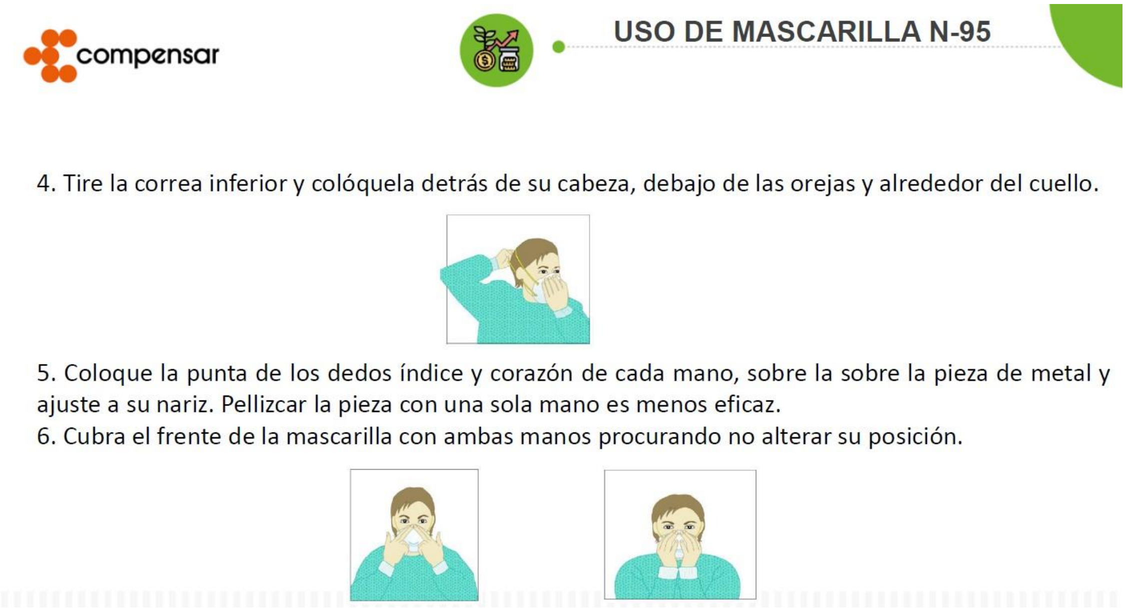

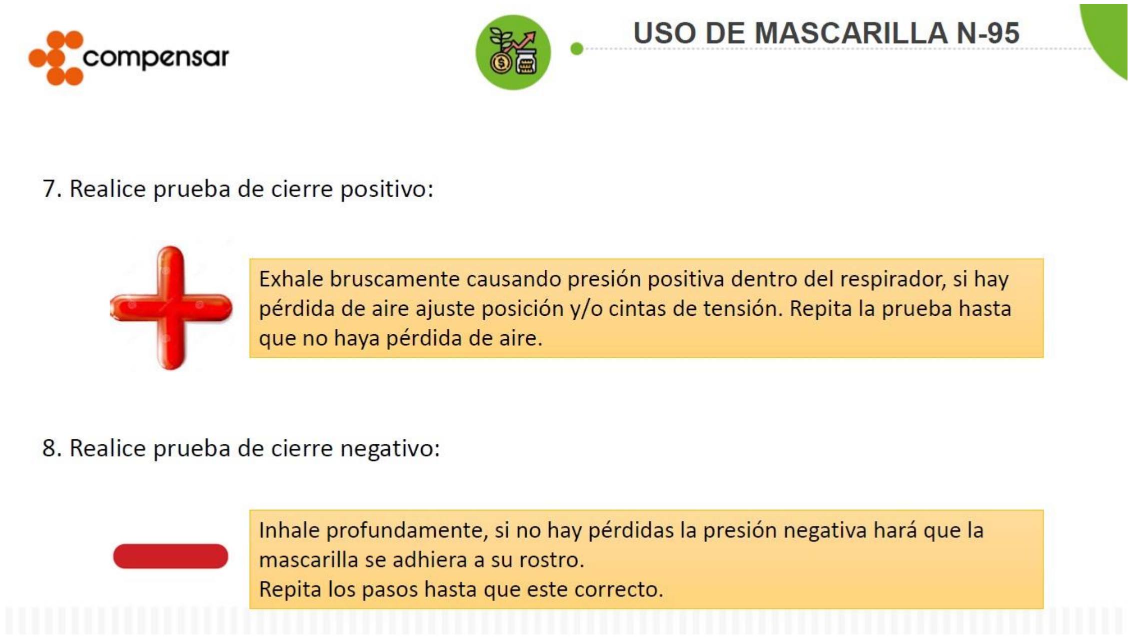

Una vez terminada la toma de los pacientes agendados, retirar los EPP según el orden establecido:

Orden para colocación y retiro de los elementos de protección personal:

COLOCACIÓN	RETIRO
1. Lavado de las manos.<br>2. Bata clínica desechable.<br>3. Gorro<br>4. Guantes<br>5. Tapabocas quirúrgico o N95.<br>6. Gafas de protección o careta.	1. Guantes<br>2. Bata o delantal.<br>3. Gafas o escudo facial.<br>4. Lavado de manos<br>5. Tapabocas<br>6. Gorro

Embalaje y transporte de muestras:

Según lo establecido en el manual de bioseguridad, las muestras deben ser embaladas en triple embalaje desde la toma de la muestra para su posterior traslado al laboratorio.

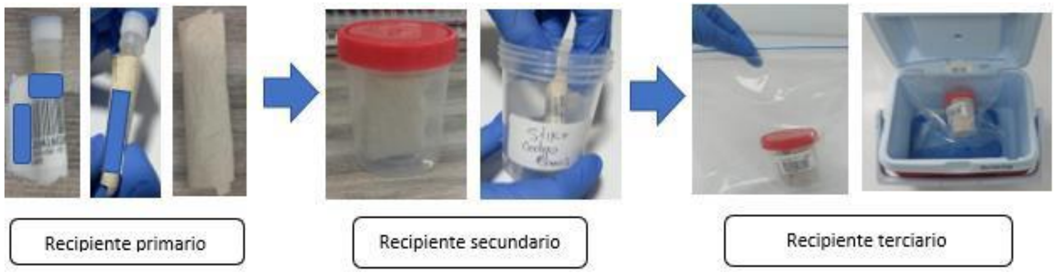

1. Cubrir el recipiente primario con papel absorbente.
2. Depositarlo dentro del recipiente secundario.
3. Depositar el recipiente secundario en bolsa ziploc y poner dentro de la nevera con pila refrigerante. La nevera estará marcada según el riesgo.
4. Al finalizar la toma de muestra, transportar al laboratorio clínico.

BIBLIOGRAFÍA

- Instructivo para la prevención, contención y atención del covid 19. INS-PSS-576. Compensar
- Procedimiento para la toma, transporte, conservación, y remisión de muestras de laboratorio clínico. PRO-PSS-080. Compensar
- Organización Mundial de la Salud. Indicaciones para la higiene de manos. 5 momentos para la higiene de manos. Mayo 2012
- IATA. Transporte de sustancias infecciosas. Versión 2019.

ANEXO 2

ANEXO 2 DE LC.01.MA.02 TABLA DE ELEMENTOS DE PROTECCIÓN PERSONAL.xlsx

ANEXO 3

ANEXO 3 DE LC.01.MA.02 ENCUESTA DE DATOS CLÍNICOS PRUEBA RÁPIDA SARS CoV 2.xlsx

ANEXO 4

ANEXO 4 DEL LC.01.MA.02 CONSENTIMIENTO INFORMADO PARA EL PROCESAMIENTO DE PRUEBAS SEROLOGICAS COVID-19.

ANEXO 5. CENTRIFUGACIÓN DE MUESTRAS

Posterior a la toma de muestra trasportar al laboratorio, identificar los tubos a centrifugar. Para los tubos utilizados para obtención de suero, se debe permitir la formación de coagulo durante 30 minutos según lo establecido por el fabricante. Posterior a este tiempo proceder a ubicar los tubos en la centrifuga uno en frente de otro verificando volúmenes equiparables. Utilizar tubos de equilibrio en caso de ser necesario.
Una vez validadas las condiciones de valanceo del instrumento proceda a programar la centrifuga de acuerdo con las recomendaciones del proveedor. Para la configuración de la centrifuga es importante tener en cuenta los términos:

- RCF: Fuerza centrífuga Relativa (Gravedades)
- RPM: Revoluciones por Minuto (RPM)

Estos términos son relevantes debido a que las condiciones de centrifugación varian de acuerdo al proveedor de los tubos e impactan enel desempeño de los dispositivos médicos. La unidad de medida mas usada para expresar las condiciones de centrifugación son las RCF o gravedades y las mas frecuente para configuración de las centrifugas son las RPM, por lo tanto es necesario que se conozca el procedimiento para la equivalencia de RCF a RPM dependiendo la referencia de centrífuga que se esté usando.
Para la centrifugación de muestras sanguíneas se tiene disponible la centrífuga Rotina 380:

HETTICTH ROTINA 380		
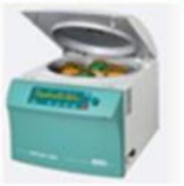	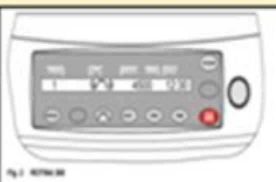	**RCF** : * Aceleración centrífuga relativa, parámtro RCF. El RCF se visualiza entre entre paréntesis)(. Se ilumina el LED en la tecla. Se puede ajustar un valor numérico del cuál resulta una velocidad de entre 50 TPM y la velocidad máxima del rotor (Nmáx). Ajustable en pasos de.
		*Radio de centrifugado, parámetro **RAD**. Ajustable de 10 mm-330mm, en pasos de 1 mm.
		* Consulta del Integral RCF. La consulta del Integral RCF sólo es posible si está activada la indicación del Integral RCF.

Para centrifugación de muestras de orina se utiliza centrifuga Rotofix 32 A:

HETTITCH ROTOFIX 32 A ROTOR ABATIBLE		
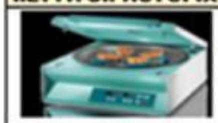	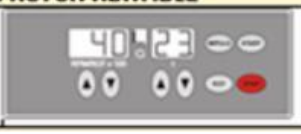	**RCF** : Indicación de la aceleración centrífuga relativa (RCF). La indicación de la aceleración centrífuga relativa (RCF) se produce mientras se mantenga pulsada la tecla **RCF** .

Se debe verificar el inserto e indicaciones según proveedor del tubo para así realizar la centrifugación de manera adecuada. Tenga presente que no se debe recentrifugar los tubos con gel ya que se puede generar desprendimiento de microparticulas y afectar los resultados.
