# Supplementary material for: Molecular characterization and descriptive analysis of carbapenemase-producing Gram-negative rod infections in Bogota, Colombia
Source: Microbiol Spectr. 2024 Apr 17;12(6):e01714-23. doi: 10.1128/spectrum.01714-23 (PMC11237484; doi:10.1128/spectrum.01714-23)
Supplement: Supplemental material — Supplemental text, tables, and figures. [file spectrum.01714-23-s0003.docx]

**Supplemental Text and Tables**

Characterization and Descriptive Analysis of Carbapenemase-Producing Gram-Negative Rod Infections in Bogota, Colombia.

Elsa D. Ibáñez-Prada^a, b¶^, Ingrid G. Bustos^a, b¶^, Enrique Gamboa-Silva^a^, Diego F. Josa^a, c^, Lina Mendez^b^, Yuli V. Fuentes^a, b^, Cristian C. Serrano-Mayorga^a, b^, Oscar Baron^a^, Alejandra Ruiz-Cuartas^a^, Edwin Silva^a, c^, Louise M. Judd^d^, Taylor Harshegyi^d^, Hector F. Africano^b^, Juan Urrego-Reyes^e^, Claudia C. Beltran^e^, Sebastian Medina^e^, Rafael Leal^c^, Andrew J. Stewardson^d^, Kelly L. Wyres^d^, Jane Hawkey^d^, and Luis Felipe Reyes^a, b, f #^.

^a^ Universidad de la Sabana, Chía, Colombia;

^b^ Clínica Universidad de La Sabana, Chía, Colombia;

^c^ Fundación Clínica Shaio, Bogotá, Colombia;

^d^ Department of Infectious Diseases, Central Clinical School, Monash University, Melbourne, Australia;

^e^ MSD Colombia, Bogotá, Colombia;

^f^ Pandemic Sciences Institute, University of Oxford, Oxford, United Kingdom.

**Running Head:** Molecular Characterization of Carbapenem Resistance.

**# Address for Correspondence:** Luis Felipe Reyes, M.D., MSc., Ph.D.; Universidad de La Sabana, Campus Puente del Común, KM 7.5 Autopista Norte de Bogotá, Chía, Colombia. Phone: (571)-861-5555 ext. 23342; Email: luis.reyes5@unisabana.edu.co

METHODS

*Study definitions*

Colonization: Colonization is the presence of bacteria on a body surface (like on the skin, mouth, intestines, or airway) without causing disease in the person (1).

Infection: Infectious disease—also called communicable disease—is any illness caused by an infective agent—a germ, microbe, or parasite. Infective agents include bacteria, viruses, fungi, parasitic protozoa, and worms; causing signs and symptoms (1).

Nosocomial-acquired infection: A nosocomial infection or healthcare-associated infection is defined by the World Health Organization as an infection occurring in a patient during the care process in a hospital or other healthcare facility, which was not present or incubating at the time of admission. This includes infections acquired in the health care facility, appearing after discharge, and occupational infections among health care workers (HCWs). Moroever, they usually develop after 48 hours of admission (2, 3). For the porpuse of this study, those that were documented within 48 hours of admission and had one of the following, were classified as healthcare-acquired: (1) hospitalization within 30 days prior to admission, (2) previous residency in a long-term care facility within 30 days prior to admission, (3) chronic kidney disease stage G4, G5, or in dialysis, (4) chronic liver disease Child-Pug B, Chuld-Pub C, or chirrosis, (5) actively immunocompromised (i.e. chronic use of oral corticosteroids, chemotherapy, acquired immunodeficiency syndrome [AIDS]), (6) active cancer, (7) pregnancy, or (8) permanent use of a medical device (i.e. permanent bladder catheter, tracheostomy, nasogastric tube, subclavian catheter).

Urinary tract infection: Was defined as the presence of micro-organisms in the urine or tissues of the normally sterile genitourinary tract (4). The European Association of Urology can classify it as complicated, uncomplicated, recurrent, catheter-associated, and urosepsis (5).

Bloodstream Infections: According to the Centers for Disease Control and Prevention, is defined by positive blood cultures in a patient with systemic signs of infection and may be either primary or secondary to a documented source (6). IDSA guidelines suggest that for most etiologic agents, conventional blood culture methods provide positive results within 48 hours (7).

Peritonitis: is inflammation of the peritoneum; IDSA guidelines classify it as Spontaneous Bacterial Peritonitis (ascitic fluid infection without an evident intra-abdominal focus), secondary peritonitis (is dependent upon identifying a source for invading microorganisms—usually genitourinary or gastrointestinal microbiota, for example, iatrogenic or accidental trauma, intestinal perforation, typhlitis, or intra-abdominal abscess), tertiary peritonitis (persistent or recurrent peritonitis following unsuccessful treatment of secondary peritonitis, it might also indicate the presence of an intra-abdominal abscess or organisms that are refractory to broad-spectrum antimicrobial therapy), and peritoneal dialysis associated-peritonitis (7).

Skin and soft tissue infection: occur when the skin’s protective mechanisms fail, especially following trauma, inflammation, maceration from excessive moisture, poor blood perfusion, or other factors that disrupt the stratum corneum that provides a point of entry for a myriad of exogenous and endogenous microbiota that can produce a variety of infections. Infections of the skin and soft tissue are often classified as primary or secondary, infections associated with underlying conditions of the skin, and necrotizing infections (7).

Community-acquired pneumonia: Was defined by ATS/IDSA guidelines as suggestive clinical features and a chest X-ray or other imaging assessment documenting alveolar infiltrates. Moreover, patients with at least three minor criteria or one major criterion of the ATS/IDSA severity criteria were diagnosed with severe CAP. Being minor criteria: respiratory rate ≥ 30 breaths/min, PaO_2_/FiO_2_ ratio ≤ 250, multilobar infiltrates, confusion/disorientation, uremia (blood urea nitrogen level, 20 mg/dL), leukopenia (white blood cell count, <4000 cells/mm^3^), thrombocytopenia (platelet count, <100,000 cells/mm^3^), hypothermia (core temperature, 36ºC), hypotension requiring aggressive fluid resuscitation. The major criteria were invasive mechanical ventilation or septic shock with the need for vasopressors (8).

Hospital-acquired pneumonia is defined by ATS/IDSA guidelines as pneumonia not incubating at the time of hospital admission and occurring 48 hours or more after admission (9, 10).

Ventilator-Associated pneumonia: is defined by the European Respiratory Society (ERS), European Society of Intensive Care Medicine (ESICM), European Society of Clinical Microbiology and Infectious Diseases (ESCMID), and Asociación Latinoamericana del Tórax (ALAT) guidelines as pneumonia that arises more than 48 hours after endotracheal intubation (9, 10).

Surgical site infections: may be caused by endogenous microbiota or originate from exogenous sources such as healthcare providers, the environment, or materials manipulated during an “incisional” or “organ/space” surgical procedure. Incisional infections are further divided into superficial (skin and subcutaneous tissue) and deep (tissue, muscle, fascia) (7).

Osteomyelitis: according to IDSA definition, is the infection of bones and can occur following hematogenous spread, after a contaminated open fracture, or in those with diabetes mellitus or vascular insufficiency (7).

*Microbiological resistance tests*

Carbapenemase-producing isolates that presented negative results by boronic acid and/or EDTA synergy tests, a modification of the method was performed, placing boronic acid (300 mg) and meropenem (10 mg) disks closer at different distances (10 mm, 5 mm, and 0 mm edge-to-edge). In the case of EDTA synergy, assays were performed with disks of EDTA (372 mg), meropenem (10 mg), and imipenem (10 mg) closer at different distances (10 mm, 5 mm, and 0 mm edge-to-edge). Moreover, for quality control, *Klebsiella pneumoniae* ATCC BAA 1705 (KPC-2), *Klebsiella pneumoniae* ATCC BAA 2146 (NDM-1), *Klebsiella pneumoniae* ATCC BAA1706, *Escherichia coli* ATCC 25922, and *Pseudomonas aeruginosa* ATCC 27853 were used as controls.

MHT was implemented using the indicator strain E. coli ATCC 25922. A 0.5 McFarland suspension was prepared in saline and diluted 1:10. Then, a Mueller-Hinton agar plate was inoculated for the routine disk diffusion procedure. The plated dried for 3 to 10 min, and a 10 mg disk of meropenem was placed in the center. Using a 10-mL loop, three to five colonies of each culture of Enterobacterales were picked to inoculate a straight line out from the edge of the disk. Plates were incubated at 36 ± 1 °C in ambient air for 16 h to 20 h.

Boronic acid sensi-discs synergy test (Britania) was carried out, a 0.5 McFarland suspension was prepared and inoculated on Mueller–Hinton agar. Then, disks of boronic acid 300 mg meropenem (10 mg) and ceftazidime (30 mg) were placed, leaving 15 mm edge-to-edge distance following the recommendations of the Colombian National Institute of Health (INS, for their Spanish acronym). Plates were incubated at 36 ± 1 °C in ambient air for 16 h to 20 h.

EDTA discs synergy test (Britania) was performed with a 0.5 McFarland suspension was prepared and inoculated on Mueller–Hinton agar. Then, disks of EDTA (372 mg), meropenem (10 mg), and imipenem (10 mg) were placed, leaving 15 mm edge-to-edge distance following the Colombian INS recommendations. Plates were incubated at 36 ± 1 °C in ambient air for 16 h to 20 h.

RESIST-4 – O.K.N.V lateral flow immunoassays (Coris BioConcept, Gembloux, Belgium) was also performed with twelve drops of the buffer solution were added to a plastic tube. Then, using a loop, three colonies of each isolate were emulsified, and the preparation was shaken for homogenization. Three drops of this suspension were dispensed into the sample well of the cassette, and the test was left to run for a maximum of 15 min. Reading was performed according to the manufacturer’s pack- age insert.

For NG-Test CARBA 5 lateral flow immunoassay (NG Biotech, Guipry, France) five drops of the buffer solution contained in the kit were added to a plastic tube were used. Subsequently, three colonies were emulsified with a loop, and the preparation was shaken for homogenization. Then, with a pipette, 100 mL of this suspension was dispensed into the cassette sample well. The test was left to run for a maximum of 15 min and read as per the manufacturer’s package insert.

*Genome analysis*

Extracted DNA was stored at room temperature, while all bacterial isolates were frozen at -80 until their analysis at Monash University, Australia. Samples were recovered through a slow thawing process. Quality control was performed for all isolates, to assess the genome analysis All genomes were assembled using Unicycler v0.5.0 with default parameters (11). Genomes with insufficient read data (<85x coverage) were excluded, and genome assemblies larger than 9 Mbp were considered mixed samples and were also excluded. Pathogenwatch (https://pathogen.watch) was used to determine the species of each genome and mlst v2.19.0 (<https://github.com/tseemann/mlst>) to determine the sequence type (ST) for those genomes representing species with an established multi-locus sequence typing (MLST) scheme. Kleborate v2.0.4 was used to identify all AMR genes (12).

To generate a multi-species phylogenetic tree, we used mash v2.1 (13) to create a distance matrix using one representative genome for each species and passed the resulting matrix to the function *fastme. bal* in the R package *ape* v5.6-2 (14). Visualizations were performed using the R packages *ggtree* v3.4.0 (15) and *ggplot2* v3.3.6 (<http://www.ievbras.ru/ecostat/Kiril/R/Biblio_N/R_Eng/Wickham2016.pdf>).

STs with 10 or more genomes were selected for additional, in-depth phylogenetic analysis. We downloaded publicly available assemblies for *P. aeuriginosa* ST111, ST235 and *K. pneumoniae* ST258 from Pathogenwatch (https://pathogen.watch/). All 365 ST111 genomes that were available were used for downstream analysis. However, due to the large number of ST235 and ST258 genomes we subsampled by selecting, at random, five genomes per country per year. This resulted in 377 ST235 genomes and 462 ST258 genomes available for downstream analysis. A full list of the public genomes used here, and their relevant metadata, can be found in **Table S7**.

For each ST, we used SKA v2 (16) to index all assemblies (both public and the ones from this study) with a kmer size of 31. We generated SNP alignments from the resulting indices using the following parameters: min-freq=1, filter, and no-const. Final alignments comprised 9996, 7875 and 7879 variable sites for *P. aeruginosa* ST111, ST235 and *K. pneumoniae* ST258, respectively. We generated maximum-likelihood phylogenies from these SNP alignments using IQTree v2 (17) with the TEST parameter to select the best substitution model. For the public genomes, we used AMRFinderPlus (18)] to detect carbapenemase genes. The resulting tree figures were plotted in R with ggtree v3.4.4 (19).

Following preliminary analyses, it was noted that a subset of genomes that had passed quality control did not contain any detectable carbapenemase gene; antibiotic susceptibility testing was repeated on the corresponding isolates received by the sequencing lab (using the VITEK 2 platform).

**TABLES**

**Table S1.** Identified bacteria through Vitek, Maldi-TOF, and WGS.

| **Microorganisms** | **Vitek**  **n=248 (%)** | **WGS**  **n=228 (%)** |
| --- | --- | --- |
| *Acinetobacter baumannii* | 2 (0.8) | 0 (0.0) |
| *Acinetobacter gyllenbergii* | 1 (0.4) | 0 (0.0) |
| *Acinetobacter spp. ANC 3929* | 0 (0.0) | 1 (0.4) |
| *Atlantibacter subterranea* | 0 (0.0) | 1 (0.4) |
| *Citrobacter braakii* | 1 (0.4) | 1 (0.4) |
| *Citrobacter freundii* | 3 (1.2) | 1 (0.4) |
| *Citrobacter koseri* | 0 (0.0) | 1 (0.4) |
| *Citrobacter portucalensis* | 0 (0.0) | 1 (0.4) |
| *Comamonas testosteroni* | 1 (0.4) | 0 (0.0) |
| *Comamonas thiooxydans* | 0 (0.0) | 1 (0.4) |
| *Enterobacter aerogenes* | 8 (3.2) | 0 (0.0) |
| *Enterobacter asburiae* | 0 (0.0) | 2 (0.9) |
| *Enterobacter cloacae* | 23 (9.3) | 2 (0.9) |
| *Enterobacter hormaechei* | 0 (0.0) | 17 (7.5) |
| *Enterobacter kobei* | 0 (0.0) | 2 (0.9) |
| *Escherichia coli* | 11 (4.4) | 7 (3.1) |
| *Klebsiella aerogenes* | 0 (0.0) | 5 (2.2) |
| *Klebsiella africana* | 0 (0.0) | 1 (0.4) |
| *Klebsiella grimontii* | 0 (0.0) | 3 (1.3) |
| *Klebsiella oxytoca* | 7 (2.8) | 3 (1.3) |
| *Klebsiella pasteurii* | 0 (0.0) | 1 (0.4) |
| *Klebsiella pneumoniae* | 90 (36.3) | 65 (28.5) |
| *Klebsiella quasipneumoniae subspp. quasipneumoniae* | 0 (0.0) | 1 (0.4) |
| *Klebsiella quasipneumoniae subspp. similipneumoniae* | 0 (0.0) | 4 (1.8) |
| *Klebsiella variicola subspp. variicola* | 0 (0.0) | 4 (1.8) |
| *Pandoraea pnomenusa* | 1 (0.4) | 1 (0.4) |
| *Proteus mirabilis* | 0 (0.0) | 1 (0.4) |
| *Providencia rettgeri* | 1 (0.4) | 1 (0.4) |
| *Pseudomonas aeruginosa* | 87 (35.1) | 87 (38.2) |
| *Pseudomonas fluorescens* | 3 (1.2) | 0 (0.0) |
| *Pseudomonas monteilii* | 0 (0.0) | 1 (0.4) |
| *Pseudomonas protegens* | 0 (0.0) | 2 (0.9) |
| *Pseudomonas putida* | 6 (2.4) | 1 (0.4) |
| *Pseudomonas spp. NBRC 111131* | 0 (0.0) | 1 (0.4) |
| *Serratia marcescens* | 3 (1.2) | 4 (1.8) |
| *Shigella dysenteriae* | 0 (0.0) | 1 (0.4) |
| *Shigella sonnei* | 0 (0.0) | 2 (0.9) |
| *Shigella spp. PAMC 28760* | 0 (0.0) | 1 (0.4) |
| *Stenotrophomonas maltophilia* | 0 (0.0) | 1 (0.4) |

**Table S3**. Microorganism identified by WGS according to their number of carbapenemase genes.

| **Microorganism, n= (%)** | **WGS, n= 228** | | | |
| --- | --- | --- | --- | --- |
|  | **1 carbapenem resistance gene,**  **n= 175 (%)** | **2 carbapenem resistance genes,**  **n= 20 (%)** | **3 carbapenem resistance genes,**  **n= 2 (%)** | **Non-identified,**  **n= 31 (%)** |
| *Acinetobacter spp. ANC 3929* | 1 (0.6) | 0 (0.0) | 0 (0.0) | 0 (0.0) |
| *Atlantibacter subterranea* | 1 (0.6) | 0 (0.0) | 0 (0.0) | 0 (0.0) |
| *Citrobacter braakii* | 1 (0.6) | 0 (0.0) | 0 (0.0) | 0 (0.0) |
| *Citrobacter freundii* | 1 (0.6) | 0 (0.0) | 0 (0.0) | 0 (0.0) |
| *Citrobacter koseri* | 1 (0.6) | 0 (0.0) | 0 (0.0) | 0 (0.0) |
| *Citrobacter portucalensis* | 1 (0.6) | 0 (0.0) | 0 (0.0) | 0 (0.0) |
| *Comamonas thiooxydans* | 1 (0.6) | 0 (0.0) | 0 (0.0) | 0 (0.0) |
| *Enterobacter asburiae* | 1 (0.6) | 0 (0.0) | 0 (0.0) | 1 (3.2) |
| *Enterobacter cloacae* | 1 (0.6) | 0 (0.0) | 0 (0.0) | 1 (3.2) |
| *Enterobacter hormaechei* | 12 (6.9) | 3 (15.0) | 0 (0.0) | 2 (6.5) |
| *Enterobacter kobei* | 2 (1.1) | 0 (0.0) | 0 (0.0) | 0 (0.0) |
| *Escherichia coli* | 6 (3.4) | 0 (0.0) | 0 (0.0) | 1 (3.2) |
| *Klebsiella aerogenes* | 4 (2.3) | 0 (0.0) | 0 (0.0) | 1 (3.2) |
| *Klebsiella africana* | 3 (1.7) | 0 (0.0) | 0 (0.0) | 1 (3.2) |
| *Klebsiella grimontii* | 0 (0.0) | 0 (0.0) | 0 (0.0) | 0 (0.0) |
| *Klebsiella oxytoca* | 3 (1.7) | 0 (0.0) | 0 (0.0) | 0 (0.0) |
| *Klebsiella pasteurii* | 1 (0.6) | 0 (0.0) | 0 (0.0) | 0 (0.0) |
| *Klebsiella pneumoniae* | 57 (32.6) | 4 (20.0) | 2 (100.0) | 2 (6.5) |
| *Klebsiella quasipneumoniae subspp. quasipneumoniae* | 1 (0.6) | 0 (0.0) | 0 (0.0) | 0 (0.0) |
| *Klebsiella quasipneumoniae subspp. similipneumoniae* | 4 (2.3) | 0 (0.0) | 0 (0.0) | 0 (0.0) |
| *Klebsiella variicola subspp. variicola* | 3 (1.7) | 1 (5.0) | 0 (0.0) | 0 (0.0) |
| *Microbacterium spp. oral taxon 186* | 0 (0.0) | 0 (0.0) | 0 (0.0) | 0 (0.0) |
| *Pandoraea pnomenusa* | 1 (0.6) | 0 (0.0) | 0 (0.0) | 0 (0.0) |
| *Proteus mirabilis* | 0 (0.0) | 0 (0.0) | 0 (0.0) | 1 (3.2) |
| *Providencia rettgeri* | 0 (0.0) | 1 (5.0) | 0 (0.0) | 0 (0.0) |
| *Providencia stuartii* | 0 (0.0) | 0 (0.0) | 0 (0.0) | 0 (0.0) |
| *Pseudomonas aeruginosa* | 58 (33.1) | 11 (55.0) | 0 (0.0) | 18 (58.1) |
| *Pseudomonas monteilii* | 1 (0.6) | 0 (0.0) | 0 (0.0) | 0 (0.0) |
| *Pseudomonas protegens* | 2 (1.1) | 0 (0.0) | 0 (0.0) | 0 (0.0) |
| *Pseudomonas putida* | 1 (0.6) | 0 (0.0) | 0 (0.0) | 0 (0.0) |
| *Pseudomonas spp. NBRC 111131* | 1 (0.6) | 0 (0.0) | 0 (0.0) | 0 (0.0) |
| *Serratia marcescens* | 4 (2.3) | 0 (0.0) | 0 (0.0) | 0 (0.0) |
| *Shigella dysenteriae* | 0 (0.0) | 0 (0.0) | 0 (0.0) | 1 (3.2) |
| *Shigella sonnei* | 1 (0.6) | 0 (0.0) | 0 (0.0) | 1 (3.2) |
| *Shigella spp. PAMC 28760* | 1 (0.6) | 0 (0.0) | 0 (0.0) | 0 (0.0) |
| *Stenotrophomonas maltophilia* | 0 (0.0) | 0 (0.0) | 0 (0.0) | 1 (3.2) |

**Table S4.** Distribution of community and hospital-acquired microorganisms with more than one identified carbapenem resistance gene through WGS.

| Microorganism, n= (%) | WGS | | | |
| --- | --- | --- | --- | --- |
|  | *bla*KPC-2 *+ bla*NDM-1, n= 9 | *bla*KPC-3 + *bla*NDM-1, n= 1 | *bla*KPC-3 + *bla*VIM-2, n= 10 | *bla*KPC-2 + *bla*NDM-1 + *bla*VIM-24, n= 2 |
| *Enterobacter hormaechei* | 3 (33.3%) |  |  |  |
| *Klebsiella pneumoniae* | 4 (44.4) |  |  | 2 (100.0) |
| *Klebsiella variicola subspp. variicola* | 1 (11.1) |  |  |  |
| *Providencia rettgeri* | 1 (11.1) |  |  |  |
| *Pseudomonas aeruginosa* |  | 1 (100.0) | 10 (100.0) |  |

**Table S5.** Bivariate analysis between alleles identified by WGS.

| **Outcome** | ***bla*KPC-2, n= 88** | ***bla*KPC-3, n= 83** | ***bla*OXA-152, n= 1** | ***bla*OXA-244, n= 1** | ***bla*VIM-2, n= 29** | ***bla*VIM-24, n= 3** | ***bla*GES-5, n= 1** | ***bla*NDM-1, n= 16** | ***P-value*** |
| --- | --- | --- | --- | --- | --- | --- | --- | --- | --- |
| In-hospital mortality,  n (%) | 30 (34.1) | 26 (31.3) | 0 (0.0) | 0 (0.0) | 11 (37.9) | 1 (33.3) | 1 (100.0) | 6 (37.5) | 0.83 |
| ICU admission,  n (%) | 66 (75.0) | 66 (79.5) | 1 (100.0) | 0 (0.0) | 24 (82.8) | 2 (66.7) | 1 (100.0) | 14 (87.5) | 0.52 |
| Vasopressor support,  n (%) | 55 (62.5) | 52 (62.7) | 1 (100.0) | 0 (0.0) | 20 (69.0) | 2 (66.7) | 1 (100.0) | 13 (81.3) | 0.61 |
| Inotropic support,  n (%) | 27 (30.7) | 30 (36.1) | 1 (100.0) | 0 (0.0) | 10 (34.5) | 1 (33.3) | 1 (100.0) | 5 (31.3) | 0.65 |
| Mechanical ventilation,  n (%) | 51 (58.0) | 52 (62.7) | 1 (100.0) | 0 (0.0) | 21 (72.4) | 2 (66.7) | 1 (100.0) | 14 (87.5) | 0.25 |

**Table S6.** Total number of genomes available for molecular analysis.

| **microorganism** | **n =248** | **Failed Quality Control n=7** | **Failed Genomic Analysis n=13** | **Total WGS n=228** |
| --- | --- | --- | --- | --- |
| *Acinetobacter spp. ANC 3929* | 1 (0.4) |  |  | 1 (0.4) |
| *Atlantibacter subterranea* | 1 (0.4) |  |  | 1 (0.4) |
| *Citrobacter braakii* | 1 (0.4) |  |  | 1 (0.4) |
| *Citrobacter freundii* | 2 (0.8) | 1 |  | 1 (0.4) |
| *Citrobacter koseri* | 1 (0.4) |  |  | 1 (0.4) |
| *Citrobacter portucalensis* | 1 (0.4) |  |  | 1 (0.4) |
| *Comamonas thiooxydans* | 1 (0.4) |  |  | 1 (0.4) |
| *Enterobacter asburiae* | 2 (0.8) |  |  | 2 (0.9) |
| *Enterobacter cloacae* | 2 (0.8) |  |  | 2 (0.9) |
| *Enterobacter hormaechei* | 20 (8.1) |  | 3 | 17 (7.5) |
| *Enterobacter kobei* | 2 (0.8) |  |  | 2 (0.9) |
| *Escherichia coli* | 8 (3.2) |  | 1 | 7 (3.1) |
| *Klebsiella aerogenes* | 6 (2.4) |  | 1 | 5 (2.2) |
| *Klebsiella africana* | 1 (0.4) |  |  | 1 (0.4) |
| *Klebsiella grimontii* | 3 (1.2) |  |  | 3 (1.3) |
| *Klebsiella oxytoca* | 3 (1.2) |  |  | 3 (1.3) |
| *Klebsiella pasteurii* | 1 (0.4) |  |  | 1 (0.4) |
| *Klebsiella pneumoniae* | 66 (26.6) |  | 1 | 65 (28.5) |
| *Klebsiella quasipneumoniae subspp. quasipneumoniae* | 2 (0.8) |  | 1 | 1 (0.4) |
| *Klebsiella quasipneumoniae subspp. similipneumoniae* | 4 (1.6) |  |  | 4 (1.8) |
| *Klebsiella variicola subspp. variicola* | 4 (1.6) |  |  | 4 (1.8) |
| *Microbacterium spp. oral taxon 186* | 1 (0.4) |  | 1 | 0 (0.0) |
| *Pandoraea pnomenusa* | 1 (0.4) |  |  | 1 (0.4) |
| *Proteus mirabilis* | 2 (0.8) | 1 |  | 1 (0.4) |
| *Providencia rettgeri* | 1 (0.4) |  |  | 1 (0.4) |
| *Providencia stuartii* | 1 (0.4) |  | 1 | 0 (0.0) |
| *Pseudomonas aeruginosa* | 91 (36.7) | 1 | 3 | 87 (38.2) |
| *Pseudomonas monteilii* | 1 (0.4) |  |  | 1 (0.4) |
| *Pseudomonas protegens* | 2 (0.8) |  |  | 2 (0.9) |
| *Pseudomonas putida* | 1 (0.4) |  |  | 1 (0.4) |
| *Pseudomonas spp. NBRC 111131* | 1 (0.4) |  |  | 1 (0.4) |
| *Serratia marcescens* | 5 (2.0) |  | 1 | 4 (1.8) |
| *Shigella dysenteriae* | 1 (0.4) |  |  | 1 (0.4) |
| *Shigella sonnei* | 2 (0.8) |  |  | 2 (0.9) |
| *Shigella spp. PAMC 28760* | 1 (0.4) |  |  | 1 (0.4) |
| *Stenotrophomonas maltophilia* | 1 (0.4) |  |  | 1 (0.4) |
| *NA* | 4 (1.6) | 4 |  | 1 (0.4) |

**
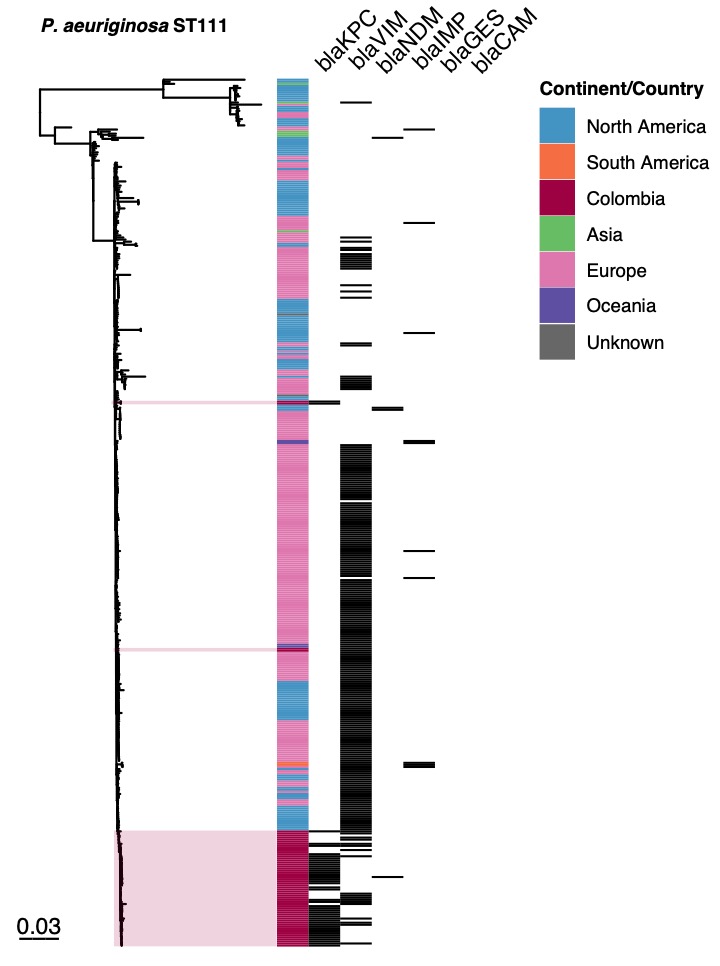
**

**Figure S1: Maximum liklhood phylogeny for 422 *Pseudmonas aeruginosa* ST111.** Geographical regsions of origina are shown as marked in the legend. Isolates from this study are further marked by red shading on the tree. The presence of carbapenemase genes is indicated by black bars.


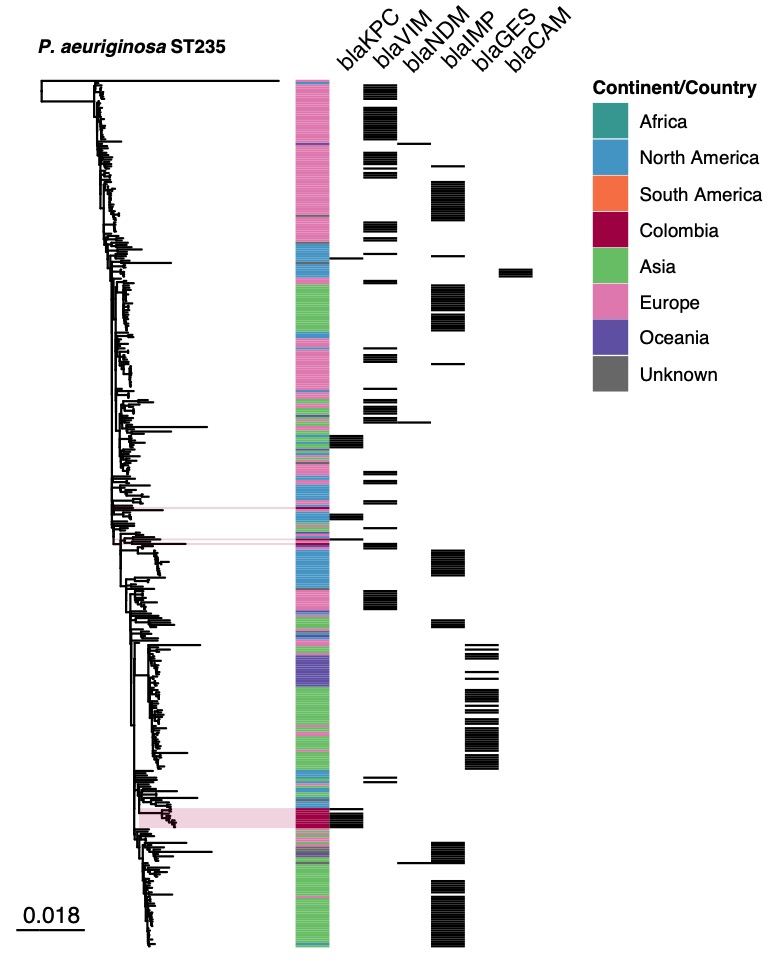


**Figure S2: Maximum liklhood phylogeny for 389 *Pseudmonas aeruginosa* ST235.** Geographical regsions of origina are shown as marked in the legend. Isolates from this study are further marked by red shading on the tree. The presence of carbapenemase genes is indicated by black bars.


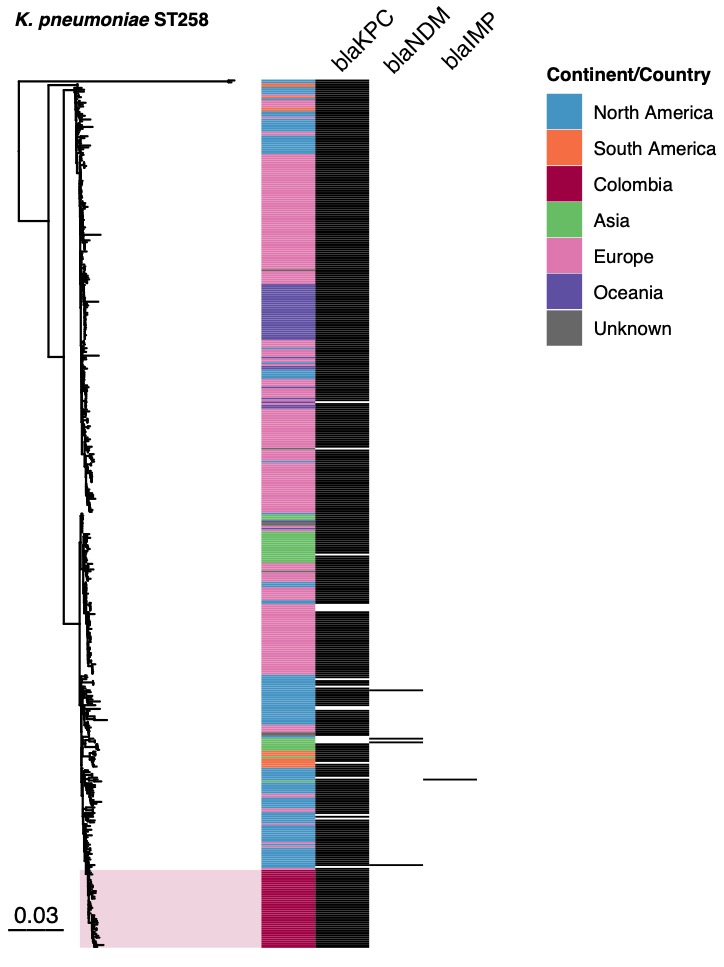


**Figure S3: Maximum liklhood phylogeny for 472 *Klebsiella pneumoniae* ST258.** Geographical regsions of origina are shown as marked in the legend. Isolates from this study are further marked by red shading on the tree. The presence of carbapenemase genes is indicated by black bars.

**REFERENCES**

1. The Gale Group I. Gale Encyclopedia of Medicine. Gale Encyclopedia of Medicine, 5th ed; 2015.

2. Sikora A, Zahra F. Nosocomial Infections. StatPearls. Treasure Island (FL); 2022.

3. Andrew Webb (ed.) DAe, Simon Finfer (ed.), Luciano Gattinoni (ed.), Mervyn Singer (ed.). Oxford Textbook of Critical Care (2 edn). In: Andrew Webb (ed.) DAe, Simon Finfer (ed.), Luciano Gattinoni (ed.), Mervyn Singer (ed.), editor: Oxford University Press; April 2016.

4. Fund IM. A Global Strategy to Manage the Long-Term Risks of COVID-19. *IMF WORKING PAPERS* 2022.

5. G. Bonkat (Chair) RB, F. Bruyère, T. Cai, S.E. Geerlings, B. Köves, S. Schubert, A. Pilatz, R. Veeratterapillay, F. Wagenlehner Guidelines Associates: W. Devlies, J. Horváth, G. Mantica, T. Mezei, B. Pradere, Guidelines Office: E.J. Smith. EAU Guidelines on Urological Infections. [serial online] 2022. Available from: <https://d56bochluxqnz.cloudfront.net/documents/full-guideline/EAU-Guidelines-on-Urological-Infections-2022.pdf>

6. Timsit JF, Ruppe E, Barbier F, Tabah A, Bassetti M. Bloodstream infections in critically ill patients: an expert statement. *Intensive Care Med* 2020; 46: 266-284.

7. Miller JM, Binnicker MJ, Campbell S, Carroll KC, Chapin KC, Gilligan PH, Gonzalez MD, Jerris RC, Kehl SC, Patel R, Pritt BS, Richter SS, Robinson-Dunn B, Schwartzman JD, Snyder JW, Telford S, 3rd, Theel ES, Thomson RB, Jr., Weinstein MP, Yao JD. A Guide to Utilization of the Microbiology Laboratory for Diagnosis of Infectious Diseases: 2018 Update by the Infectious Diseases Society of America and the American Society for Microbiology. *Clin Infect Dis* 2018; 67: e1-e94.

8. Mandell LA, Wunderink RG, Anzueto A, Bartlett JG, Campbell GD, Dean NC, Dowell SF, File TM, Jr., Musher DM, Niederman MS, Torres A, Whitney CG, Infectious Diseases Society of A, American Thoracic S. Infectious Diseases Society of America/American Thoracic Society consensus guidelines on the management of community-acquired pneumonia in adults. *Clin Infect Dis* 2007; 44 Suppl 2: S27-72.

9. Torres A, Niederman MS, Chastre J, Ewig S, Fernandez-Vandellos P, Hanberger H, Kollef M, Li Bassi G, Luna CM, Martin-Loeches I, Paiva JA, Read RC, Rigau D, Timsit JF, Welte T, Wunderink R. International ERS/ESICM/ESCMID/ALAT guidelines for the management of hospital-acquired pneumonia and ventilator-associated pneumonia: Guidelines for the management of hospital-acquired pneumonia (HAP)/ventilator-associated pneumonia (VAP) of the European Respiratory Society (ERS), European Society of Intensive Care Medicine (ESICM), European Society of Clinical Microbiology and Infectious Diseases (ESCMID) and Asociacion Latinoamericana del Torax (ALAT). *Eur Respir J* 2017; 50.

10. Kalil AC, Metersky ML, Klompas M, Muscedere J, Sweeney DA, Palmer LB, Napolitano LM, O'Grady NP, Bartlett JG, Carratala J, El Solh AA, Ewig S, Fey PD, File TM, Jr., Restrepo MI, Roberts JA, Waterer GW, Cruse P, Knight SL, Brozek JL. Management of Adults With Hospital-acquired and Ventilator-associated Pneumonia: 2016 Clinical Practice Guidelines by the Infectious Diseases Society of America and the American Thoracic Society. *Clin Infect Dis* 2016; 63: e61-e111.

11. Wick RR, Judd LM, Gorrie CL, Holt KE. Unicycler: Resolving bacterial genome assemblies from short and long sequencing reads. *PLoS Comput Biol* 2017; 13: e1005595.

12. Lam MMC, Wick RR, Watts SC, Cerdeira LT, Wyres KL, Holt KE. A genomic surveillance framework and genotyping tool for Klebsiella pneumoniae and its related species complex. *Nat Commun* 2021; 12: 4188.

13. Ondov BD, Treangen TJ, Melsted P, Mallonee AB, Bergman NH, Koren S, Phillippy AM. Mash: fast genome and metagenome distance estimation using MinHash. *Genome Biol* 2016; 17: 132.

14. Paradis E, Schliep K. ape 5.0: an environment for modern phylogenetics and evolutionary analyses in R. *Bioinformatics* 2019; 35: 526-528.

15. Yu G. Using ggtree to Visualize Data on Tree-Like Structures. *Curr Protoc Bioinformatics* 2020; 69: e96.

16. Harris SR. SKA: Split Kmer Analysis Toolkit for Bacterial Genomic Epidemiology. *bioRxiv* 2018: 453142.

17. Minh BQ, Schmidt HA, Chernomor O, Schrempf D, Woodhams MD, von Haeseler A, Lanfear R. Corrigendum to: IQ-TREE 2: New Models and Efficient Methods for Phylogenetic Inference in the Genomic Era. *Mol Biol Evol* 2020; 37: 2461.

18. Feldgarden M, Brover V, Gonzalez-Escalona N, Frye JG, Haendiges J, Haft DH, Hoffmann M, Pettengill JB, Prasad AB, Tillman GE, Tyson GH, Klimke W. AMRFinderPlus and the Reference Gene Catalog facilitate examination of the genomic links among antimicrobial resistance, stress response, and virulence. *Sci Rep* 2021; 11: 12728.

19. Yu G, Smith DK, Zhu H, Guan Y, Lam TT-Y. ggtree: an r package for visualization and annotation of phylogenetic trees with their covariates and other associated data. *Methods in Ecology and Evolution* 2017; 8: 28-36.
